# Supplementary material for: Human monocytotropic ehrlichiosis—A systematic review and analysis of the literature
Source: PLoS Negl Trop Dis. 2024 Aug 2;18(8):e0012377. doi: 10.1371/journal.pntd.0012377 (PMC11324158; doi:10.1371/journal.pntd.0012377)
Supplement: S1 Text — (DOCX) [file pntd.0012377.s001.docx]

**List of databases with search term used**

- **Pubmed**

((“Ehrlichia” [Mesh] OR “Ehrlichiosis” [Mesh] AND “Monocytes” [Mesh])) OR (“Ehrlichi*” [tiab] OR “Neoehrlichi*” [tiab] OR “HME Agent” [tiab] OR “chaffeensis” [tiab] OR “ruminantium” [tiab] OR “muris eauclairensis” [tiab] OR “muris” [tiab] OR “sennetsu” [tiab] OR “ewingii” [tiab] OR “Ehrlichia canis” [tiab]) NOT (“Animals” [Mesh] NOT “Humans” [Mesh]))

- **Embase**

(('ehrlichia'/syn OR 'ehrlichiosis'/syn OR 'human monocytic ehrlichiosis'/syn) AND 'monocyte'/syn) OR (ehrlichi*:ti,ab OR neoehrlichi*:ti,ab OR 'hme agent':ti,ab) OR (chaffeensis:ti,ab OR ruminantium:ti,ab OR 'muris eauclairensis':ti,ab OR muris:ti,ab OR sennetsu:ti,ab OR ewingii:ti,ab OR “Ehrlichia canis”:ti,ab) NOT ('animal'/exp NOT 'humans'/exp)

- **Cochrane**

(([mh Ehrlichia] OR [mh Ehrlichiosis] AND [mh Monocytes])) OR (Ehrlichi*:ti,ab OR Neoehrlichi*:ti,ab OR "HME Agent":ti,ab OR chaffeensis:ti,ab OR ruminantium:ti,ab OR "muris eauclairensis":ti,ab OR muris:ti,ab OR sennetsu:ti,ab OR ewingii:ti,ab OR “Ehrlichia canis”:ti,ab) NOT ([mh Animals] NOT [mh Humans])

- **Web of Science**

TS=(Ehrlichia OR Ehrlichiosis OR Neoehrlichia OR Neoehrlichiosis OR “HME Agent” OR “human monocytic ehrlichiosis” OR ewingii OR sennetsu OR “Ehrlichia canis” OR “muris eauclairensis” OR chaffeensis OR ruminantium OR muris) AND TS=(Humans OR Human)

- **Scopus**

(TITLE-ABS (Ehrlichia) OR TITLE-ABS (Ehrlichiosis) OR TITLE-ABS (“HME Agent”) OR TITLE-ABS (Neoehrlichia) OR TITLE-ABS (Neoehrlichiosis)) OR (TITLE-ABS (chaffensis) OR TITLE-ABS (ewingii) OR TITLE-ABS (ruminantium) OR TITLE-ABS (sennetsu) OR TITLE-ABS (muris) OR TITLE-ABS (“muris eauclairensis”) OR TITLE-ABS (“Ehrlichia canis”)) OR (AUTHKEY (ehrlichia) OR AUTHKEY (ehrlichiosis) OR AUTHKEY (“HME Agent”) OR AUTHKEY (Neoehrlichia) OR AUTHKEY (Neoehrlichiosis)) OR (AUTHKEY (chaffensis) OR AUTHKEY (ewingii) OR AUTHKEY (ruminantium) OR AUTHKEY (sennetsu) OR AUTHKEY (muris) OR AUTHKEY (“muris eauclairensis”) OR AUTHKEY (“Ehrlichia canis”)) AND (TITLE-ABS (human) OR TITLE-ABS (humans) OR AUTHKEY (human) OR AUTHKEY (humans))

- **CINAHL**

(TI Ehrlichia OR AB Ehrlichia) OR (TI Ehrlichiosis OR AB Ehrlichiosis) OR (TI Neoehrlichia OR AB Neoehrlichia) OR (TI Neoehrlichiosis OR AB Neoehrlichiosis) OR (TI "HME Agent" OR AB "HME Agent") AND (TI monocyt* OR AB monocyt*)) OR (TI chaffensis OR AB chaffensis) OR (TI ewingii OR AB ewingii) OR (TI sennetsu OR AB sennetsu) OR (TI ruminantium OR AB ruminantium) OR (TI muris OR AB muris) OR (TI “muris eauclairensis” OR AB “muris eauclairensis”) OR (TI “Ehrlichia canis” OR AB “Ehrlichia canis”) AND (TI monocyt* OR AB monocyt*))
