# Supplementary material for: Human monocytotropic ehrlichiosis—A systematic review and analysis of the literature
Source: PLoS Negl Trop Dis. 2024 Aug 2;18(8):e0012377. doi: 10.1371/journal.pntd.0012377 (PMC11324158; doi:10.1371/journal.pntd.0012377)
Supplement: S2 Text — (DOCX) [file pntd.0012377.s002.docx]

Systematic Review Protocol

Inhaltsverzeichnis

[Review Title 2](#_Toc122010492)

[Timescale 2](#_Toc122010493)

[Review team details 2](#_Toc122010494)

[Review methods 4](#_Toc122010495)

[Review questions: 4](#_Toc122010496)

[Searches 4](#_Toc122010497)

[Condition/ Domain being studies 4](#_Toc122010498)

[Participants/Population 4](#_Toc122010499)

[Intervention(s), Exposure(s) 4](#_Toc122010500)

[Comparators/Control 4](#_Toc122010501)

[Types of study to be included initially 4](#_Toc122010502)

[Primary outcome 5](#_Toc122010503)

[Measures of effect 5](#_Toc122010504)

[Secondary outcomes 5](#_Toc122010505)

[Selection strategy 5](#_Toc122010506)

[Data extraction 5](#_Toc122010507)

[Risk of bias (quality) assessment 8](#_Toc122010508)

[Strategy for data synthesis 9](#_Toc122010509)

[Analysis of subgroups or subsets 9](#_Toc122010510)

[Additional Information 10](#_Toc122010511)

# Review Title

Human Ehrlichiosis: A systematic review of the literature

# Timescale

Anticipated Start date: 14 November 2022

Anticipated Completion date: 27 October 2023

Stage of review at time of submission to PROSPERO:

- Preliminary searches: completed
- Piloting of study selection process: completed

# Review team details

Named Contact: Larissa Gygax

Named contact email: [larissa.gygax@swisstph.ch](mailto:larissa.gygax@swisstph.ch)

Organisational affiliation of the review:

- Swiss Tropical and Public Health Institute, Basel, Switzerland.
- University of Basel, Basel, Switzerland.

Team Members and their Organisational affiliation

- Larissa Gygax:
- Swiss Tropical and Public Health Institute, Basel, Switzerland.
- University of Basel, Basel, Switzerland.
- Sophie Schudel:
- Swiss Tropical and Public Health Institute, Basel, Switzerland.
- University of Basel, Basel, Switzerland.
- Esther Kuenzli:
- Swiss Tropical and Public Health Institute, Basel, Switzerland.
- University of Basel, Basel, Switzerland.
- Christian Kositz
- Swiss Tropical and Public Health Institute, Basel, Switzerland.
- Clinical Research Department, Faculty of Infectious and Tropical Diseases, London School of Hygiene & Tropical Medicine, London, United Kingdom.
- Andreas Neumayr:
- Swiss Tropical and Public Health Institute, Basel, Switzerland.
- University of Basel, Basel, Switzerland.
- Department of Public Health and Tropical Medicine, College of Public Health, Medical and Veterinary Sciences, James Cook University, Queensland, Australia.

Reviewer roles

- Primary reviewers: Larissa Gygax
  Secondary reviewers: Andreas Neumayr, Sophie Schudel, Christian Kositz, Esther Kuenzli
  Quality assessors: Esther Kuenzli, Christian Kositz, Andreas Neumayr

Funding: Self financed, no external funding

Conflicts of interest/ Competing interest: The authors declare that they have no known conflicts of interest.

Type and method of review: Systematic Review, Health area: Infections and infestations

Language: English

Country: Switzerland

Keywords: systematic review, *Ehrlichia*, human ehrlichiosis, *Ehrlichia chaffeensis*, human monocytotropic ehrlichiosis

Current review status: completed

# Review methods

## Review questions:

- What is the epidemiology and geographical range of human ehrlichiosis?
- What are risk factors associated with acquiring ehrlichiosis and for sequelae and mortality?
- What is the clinical spectrum (signs and symptoms) and the laboratory findings of human ehrlichiosis?
- What antibiotic treatment regimens are used for human ehrlichiosis?

## Searches

We will search the following bibliographic databases: PubMed, Embase Elsevier, CINAHL, Scopus, Web of Science Core Collection.

The search strategy will include terms relating to Human Ehrlichiosis. The search strategy for PubMed will be the following:

((“Ehrlichia” [Mesh] OR “Ehrlichiosis” [Mesh] AND “Monocytes” [Mesh])) OR (“Ehrlichi*” [tiab] OR “Neoehrlichi*” [tiab] OR “HME Agent” [tiab] OR “chaffeensis” [tiab] OR “ruminantium” [tiab] OR “muris eauclairensis” [tiab] OR “muris” [tiab] OR “sennetsu” [tiab] OR “ewingii” [tiab] OR “Ehrlichia canis” [tiab]) NOT (“Animals” [Mesh] NOT “Humans” [Mesh]))

The search term will be adapted for use with other bibliographic databases.

The search will be restricted to papers in English, German, French, Spanish or Italian. There will be no geographical restriction nor date limitation.

The searches will be re-run just before the final analyses and further studies retrieved for inclusion.

## Condition/ Domain being studies

Ehrlichiosis is a zoonotic bacterial infection transmitted to humans by Ione star ticks (*Amblyomma americanum*) causing primarily an undifferentiated febrile illness.

## Participants/Population

Human ehrlichiosis cases

## Intervention(s), Exposure(s)

Not applicable

## Comparators/Control

Not applicable

## Types of study to be included initially

There are no restrictions on the types of study to be included. Since the search strategy is rather broad and the estimated amount of high quality studies rather low, this review will consider all types of published study designs, not only limited to RCTs. Quality assessment will be crucial before definite inclusion. In doubt, two independent reviewers will be consulted for further assessment. What types of studies eventually will be included, will become apparent after completing the search and quality assessment.

## Primary outcome

Of interest to the review are epidemiological, clinical and diagnostic findings as well as data on therapy regimens and clinical outcome of human ehrlichiosis, as specified in the data extraction list.

## Measures of effect

The frequency of the signs/symptoms, laboratory abnormalities, complications/sequelae and the outcome of human ehrlichiosis cases will be descriptively summarized (using percentages, medians, ranges). The obtained geographic data will be reported as maps.

## Secondary outcomes

Not applicable

## Selection strategy

Identified material will be de-duplicated both by automatic search for duplicates by Endnote software and manual search for duplicates, following this review. Titles and abstracts of material identified via searches will be screened and reviewed manually. Screening will be conducted by two reviewers. Secondly, the reference lists of identified relevant articles will be manually searched for additional studies or articles. Further identified material will be again screened and reference lists will be searched. Full text papers of potentially eligible articles will be obtained.
Inclusion criteria will be applied and full text papers selected for the review. Studies that didn’t fulfill the criteria for inclusion will be excluded and their bibliographic details will be listed in an Appendix.
Results will be reported using a PRIMSA diagram. The bibliographic software ENDNOTE will be used for storage and processing.

## Data extraction

A standardized, pre-piloted form will be used to extract data from included studies for assessment of study quality and evidence synthesis. Extracted information will include: study characteristics, patient characteristics, epidemiological, clinical and laboratory findings, diagnostic measures, therapy regimen and outcome data. A detailed list of extracted parameters is available below:

Study characteristics:

- Reference No. according to our search list
- Year
- First author
- Title
- Journal
- Country of study
- Type of study
- Study Period
- Inclusion / exclusion of article; If exclusion, reason for exclusion
- Number of Human ehrlichiosis cases reported in the reference
- Number of cases already reported
- Cohort study: (Human ehrlichiosis out of how many cases of what)
- Patient specifity of data

Epidemiology

- Clinical Patient's age (years)
- Patient's sex
- Most likely country of acquisition
- Most likely province of acquisition
- If imported: time between end of trip and symptoms
- Country of diagnosis
- Province of diagnosis
- Autochthonous or imported case
- Occupational/ recreational risk factor for tick bite
- Year of Acquisition
- Pre-existing medical conditions
- Immunocompromised yes/ no
- Pregnancy or breast feeding

Presentation

- Symptomatic / asymptomatic
- Hospital admission
- Duration of symptoms (fever)
- Fever
  - highest temperature measured
- Chills
- Malaise/ fatigue
- Rash
- Presence of eschar, erythema migrans
- Headache
- Myalgia
- Arthralgia
- Lymphadenopathy
- Nausea
- Vomiting
- Abdominal pain
- Diarrhea
- Anorexia
- Hepatosplenomegaly
- Cough
- Dyspnea
- Confusion
- Meningeal symptoms
- Neck stiffness
- Neck pain
- Photophobia
- Altered mental status / lethargy
- Seizure
- Other neurological signs and symptoms
- Conjunctivitis
- Dizziness
- Vertigo
- Sore throat / pharyngitis
- Chest pain
- Weakness
- Tachycardia
- Hypotension
- Other cardiovascular signs and symptoms
- Other symptoms
- Duration between onset of fever and appropriate treatment
- Specify presumed vector of disease
  - Tick bite remembered
  - Tick species
  - Duration between bite and symptoms

Diagnostics

- Serology
  - Days between acute and convalescent sample
- PCR
- Blood smear or buffy coat microscopy
  - Percentage of infected monocytes
  - Morulae in which cell line(s)
- Culture
- Biopsy
- Level of diagnostic certainty
- Time of first specific diagnostic test
- Additional diagnostics
- Ehrlichia species
- Coinfections
- Diagnosis given initially
- Leukopenia acc. author
  - Leucopenia: exact value
  - Specify leucopenia
- Thrombocytopenia acc. author
  - Thrombocytopenia: exact value
- Anemia acc. author
  - Hemoglobin
  - Hematocrit
- Elevated liver enzymes (at least one)
  - (AST) Aspartate aminotransferase elevated
    - AST: exact value
  - (ALT) Alanine aminotransferase elevated
    - ALT: exact value
  - (AP) Alkaline phosphatase elevated
    - AP: Exact Value
  - GGT: exact value
  - Total bilirubin elevated
    - Bilirubin: exact value
- CRP elevated
  - CRP highest
- D-Dimer elevated
  - D-Dimer: exact value
- ESR elevated
  - ESR: exact value
- Procalcitonin
- LDH: exact value
- Blood urea nitrogen elevated
- Blood urea nitrogen: exact value
- Creatinine elevated
  - Creatinine: exact value
- Ferritin: exact value
- Sodium: exact value
- Albumin: exact value
- CSF findings
- Other laboratory findings

Treatment

- Received antibiotics
  - If no antibiotics specify why
- Appropriate antibiotics given
- Time of appropriate antibiotics
- Compound
- Empirical antibiotic therapy
- Time between presentation to hospital and specific therapy
- Time until afebrile after start of appropriate antibiotic therapy
- Duration of antibiotic therapy
- Dosage of antibiotic therapy
- Side effects of treatment
- Other treatment

Outcome

- Complications
- Outcome
  - Specify cause of death
  - Time from symptom onset to death
  - Sequelae

## Risk of bias (quality) assessment

The selected full text papers will be assessed by two reviewers for methodological validity prior to inclusion into the review. Any disagreements will be resolved through discussion between the reviewers, further disagreement will be intended to be resolved by a third independent reviewer. Due to the low expected number of high-quality study designs especially questions about completeness of data and selective reporting are thought to be relevant as the great majority of studies will not be randomised or blinded.

It will be considered and discussed how the quality assessment results might have an impact on the conclusions and recommendations of the review. Discussion will be presented in the “Discussion” section of the review.

The review itself will be checked using a systematic review quality assessment tool, the PRISMA checklist.

## Strategy for data synthesis

The data will be visually presented in summary tables and synthesized narratively in a sense of an observational analysis. As far as the available data allows, associations and conclusions will be drawn. Where we can, we will attempt to group similar data.

At this stage, the decision will be made whether the data will only be synthesized narratively or if the data is sufficient for a meta-analysis. Four aspects will be assessed whether it is appropriate to combine the results in a meta-analysis.

1: Studies should be similar in terms of the patients (inclusion criteria, patient characteristics)
2: Interventions/Exposures and Comparators should be the same
3: The same outcomes should be reported (primary or secondary, as well as time frames)
4: The results should show that the effects/impacts are generally going into the same direction (visualized by forest plot using a statistical software)

If all four criteria are sufficiently fulfilled by the data from reviewed studies, a meta-analysis will be performed – due to rather expectable lack of sufficient homogeneous data, further planning in this direction is not appropriate now. In case of only some studies meeting all the criteria, it may be considered to perform a meta-analysis only using those studies. In this case, a sensitivity analysis will be carried out, using the remaining studies to test the robustness of the results. Any decisions will be justified in the text of the review, clearly setting out the reasons of why a meta-analysis was performed or not.
As the scoping search showed a relatively limited number of published studies, we may expect limitations due to a lack of data. It is likely in this review, that we will have to deal with a variety of different study designs with different study aims, resulting in a rather large heterogeneity. Furthermore, the review question isn’t just comparing two different interventions on the outcome, we want to get a further picture of human ehrlichiosis. Examining the epidemiology, the clinical impact and laboratory findings, evaluating different therapy plans and outcomes, we will most likely have to deal with a large variety of data or possibly a lack of data. Since one of the aims of our review is to show the current state of knowledge, discovering possible knowledge gaps, this will be considered and discussed in the “discussion” and “conclusions” section.

## Analysis of subgroups or subsets

If the necessary data are available, subgroup analyses will be done for cases from different world regions, age groups, ehrlichia species as well as possible risk factors for poor outcome. As the scope and quality of data is still unclear at this point it is not possible to specify the groups in advance.

## Additional Information

This systematic review will be conducted in line and with the same intention as the below listed systematic reviews previously conducted by our team:

https://PubMed.ncbi.nlm.nih.gov/35171908/

https://PubMed.ncbi.nlm.nih.gov/33705384/

https://PubMed.ncbi.nlm.nih.gov/33705387/
