# Supplementary material for: Human monocytotropic ehrlichiosis—A systematic review and analysis of the literature
Source: PLoS Negl Trop Dis. 2024 Aug 2;18(8):e0012377. doi: 10.1371/journal.pntd.0012377 (PMC11324158; doi:10.1371/journal.pntd.0012377)
Supplement: S3 Text — (DOCX) [file pntd.0012377.s003.docx]

Reference Table Laboratory Data

| D-Dimere | < 0.50 | µg/ml |
| --- | --- | --- |
| ESR | F: 0-28  M: 0-19 | mm/h |
| Procalcitonin | < 0.1 | ng/ml |
| Albumin in Plasma | < 1 year: 33-42  < 1-7 years: 35 -49  < 7-18 years: 35-53  > 18 years: 35-52 | g/L |
| Ferritin | < 1 year: 11-327  1-4 years: 7-61  M:  4-7 years: 4-64  7-13 years: 14-124  13-18 years: 14-152  > 18 years: 30-300  F:  4-7 years: 13-55  7-13 years: 8-79  13-18 years: 12-68  > 18 years: 10-200 | µg/L |
| Hemoglobin | M:  14-18  F:  12-16 | g/dl |
| Hematocrit | M:  43-49  F:  36-46 | % |
| WBC | 4-10 | 10^3/µl |
| Platelets | 150-350 | 10^3/µl |
| CRP | 0.01-5 | mg/l |
| AST | 8-38 | U/I |
| ALT | 4-44 | U/I |
| GGT | 16-73 | U/I |
| Total bilirubin | 2-21 | µmol/l |
| AP | 38-126 | U/I |
| Creatinine | <15 days: 28-81  15 days - 2 years: 9-35  2-5 years: 18-38  5-12 years: 27-54  12-15 years: 40-72  M:  15-18 years: 55-95  >18 years: 59-104  W:  15-18 years: 43-72  >18 years: 45-84 | µmol/l |
| Sodium | 136-149 | mmol/l |
| CK | 40-200 | U/I |
| LDH | 106-211 | U/I |
| Blood urea nitrogen | M:  18-50 years: 8.8-20.2  > 50 years: 9.4-24.0  F:  18-50 years: 7.2-18.5  > 50 years: 8.3-21.5 | mg/dl |
