# Supplementary material for: Human monocytotropic ehrlichiosis—A systematic review and analysis of the literature
Source: PLoS Negl Trop Dis. 2024 Aug 2;18(8):e0012377. doi: 10.1371/journal.pntd.0012377 (PMC11324158; doi:10.1371/journal.pntd.0012377)
Supplement: S4 Text — (DOCX) [file pntd.0012377.s004.docx]

| **Screening and Selecting Tool – Data extraction Sheet** | | | | | | | |
| --- | --- | --- | --- | --- | --- | --- | --- |
| **Reviewer name:** | | | | **Date:** | | | |
| **Author name/type of study/country:** | | | | **Year:** | | | |
| **Title:** | | | | **Journal:** | | | |
| **Studies excluded because:** | | | | | | | |
| Not related to human ehrlichiosis | Language | Unsuitable study type (e.g. Review) | Duplicate | Insufficient data | Seroprevalence only, no clinical cases | Related to veterinary ehrlichiosis | Not retrievable |
|  | **Number of HE cases reported in the reference** | | | | | | |
|  | **Number of cases already reported** | | | | | | |
|  | **Cohort study: (HE out of how many cases of what)** | | | | | | |
|  | **Running patient No.** | | | | | | |
|  | **Patient specificity of data** | | | | | | |
|  | **No data on epidemiology** | | | | | | |
|  | **Patient's age** | | | | | | |
|  | **Patient's sex** | | | | | | |
|  | **Most likely country of acquisition** | | | | | | |
|  | **Most likely province of acquisition** | | | | | | |
|  | **If imported: Time between end of trip and symptoms** | | | | | | |
|  | **Country of diagnosis** | | | | | | |
|  | **Province of diagnosis** | | | | | | |
|  | **Autochthonous or imported case** | | | | | | |
|  | **Occupational / recreational risk factor for tick bite** | | | | | | |
|  | **If risk factor for tick bite specify** | | | | | | |
|  | **Year of acquisition** | | | | | | |
|  | **Pre-existing medical conditions** | | | | | | |
|  | **Immunocompromised** | | | | | | |
|  | **If immunocompromised specify** | | | | | | |
|  | **Pregnancy** | | | | | | |
|  | **If pregnancy: which week** | | | | | | |
|  | **No data on symptoms** | | | | | | |
|  | **Symptomatic / asymptomatic** | | | | | | |
|  | **Data on individual symptoms available** | | | | | | |
|  | **Hospital admission** | | | | | | |
|  | **Time between first symptoms and presentation to hospital/physician** | | | | | | |
|  | **Duration of hospital stay** | | | | | | |
|  | **Duration of symptoms (fever)** | | | | | | |
|  | **Fever** | | | | | | |
|  | **Highest temperature measured** | | | | | | |
|  | **Chills/rigor** | | | | | | |
|  | **Sweats** | | | | | | |
|  | **Malaise / fatigue** | | | | | | |
|  | **Rash** | | | | | | |
|  | **Presence of eschar, erythema migrans** | | | | | | |
|  | **Headache** | | | | | | |
|  | **Myalgia** | | | | | | |
|  | **Arthralgia** | | | | | | |
|  | **Lymphadenopathy** | | | | | | |
|  | **Nausea** | | | | | | |
|  | **Vomiting** | | | | | | |
|  | **Abdominal pain** | | | | | | |
|  | **Diarrhea** | | | | | | |
|  | **Anorexia** | | | | | | |
|  | **Hepatosplenomegaly** | | | | | | |
|  | **Cough** | | | | | | |
|  | **Dyspnea** | | | | | | |
|  | **Confusion** | | | | | | |
|  | **Meningeal symptoms** | | | | | | |
|  | **Neck stiffness** | | | | | | |
|  | **Neck pain** | | | | | | |
|  | **Photophobia** | | | | | | |
|  | **Altered mental status / lethargy** | | | | | | |
|  | **Seizure** | | | | | | |
|  | **Other neurological signs and symptoms** | | | | | | |
|  | **Conjunctivitis** | | | | | | |
|  | **Dizziness** | | | | | | |
|  | **Vertigo** | | | | | | |
|  | **Sore throat / pharyngitis** | | | | | | |
|  | **Chest pain** | | | | | | |
|  | **Weakness** | | | | | | |
|  | **Tachycardia** | | | | | | |
|  | **Hypotension** | | | | | | |
|  | **Other cardiovascular signs and symptoms** | | | | | | |
|  | **Other symptoms** | | | | | | |
|  | **Duration between onset of fever and appropriate treatment** | | | | | | |
|  | **Specify presumed vector of disease** | | | | | | |
|  | **Tick bite remembered** | | | | | | |
|  | **Tick species** | | | | | | |
|  | **Duration between bite and symptoms** | | | | | | |
|  | **No data on diagnostics** | | | | | | |
|  | **Serology** | | | | | | |
|  | **Days between acute and convalescent sample** | | | | | | |
|  | **PCR** | | | | | | |
|  | **Blood smear or buffy coat microscopy** | | | | | | |
|  | **Percentage of infected monocytes** | | | | | | |
|  | **Morulae in which cell line(s)** | | | | | | |
|  | **Culture** | | | | | | |
|  | **Biopsy** | | | | | | |
|  | **Level of diagnostic certainty** | | | | | | |
|  | **Time of first specific diagnostic test** | | | | | | |
|  | **Additional diagnostics** | | | | | | |
|  | **Ehrlichia species** | | | | | | |
|  | **Coinfections** | | | | | | |
|  | **If coinfection(s) specify** | | | | | | |
|  | **Diagnosis given initially** | | | | | | |
|  | **Data on individual laboratory data available** | | | | | | |
|  | **Leukopenia acc. author** | | | | | | |
|  | **Leucopenia: exact value** | | | | | | |
|  | **Specify leucopenia** | | | | | | |
|  | **Thrombocytopenia acc. author** | | | | | | |
|  | **Thrombocytopenia: exact value** | | | | | | |
|  | **Anemia acc. author** | | | | | | |
|  | **Hemoglobin** | | | | | | |
|  | **Hematocrit** | | | | | | |
|  | **Elevated liver enzymes (at least one)** | | | | | | |
|  | **(AST) Aspartate aminotransferase elevated** | | | | | | |
|  | **AST: exact value** | | | | | | |
|  | **(ALT) Alanine aminotransferase elevated** | | | | | | |
|  | **ALT: exact value** | | | | | | |
|  | **(AP) Alkaline phosphatase elevated** | | | | | | |
|  | **AP: exact Value** | | | | | | |
|  | **GGT: exact value** | | | | | | |
|  | **Total Bilirubin elevated** | | | | | | |
|  | **Bilirubin: exact value** | | | | | | |
|  | **CRP elevated** | | | | | | |
|  | **CRP highest** | | | | | | |
|  | **D-Dimer elevated** | | | | | | |
|  | **D-Dimer: exact value** | | | | | | |
|  | **ESR elevated** | | | | | | |
|  | **ESR: exact value** | | | | | | |
|  | **Procalcitonin** | | | | | | |
|  | **LDH: exact value** | | | | | | |
|  | **Blood urea nitrogen elevated** | | | | | | |
|  | **Blood urea nitrogen: exact value** | | | | | | |
|  | **Creatinine elevated** | | | | | | |
|  | **Creatinine: exact value** | | | | | | |
|  | **Ferritin: exact value** | | | | | | |
|  | **Sodium: exact value** | | | | | | |
|  | **Albumin: exact value** | | | | | | |
|  | **CSF findings** | | | | | | |
|  | **Other laboratory findings** | | | | | | |
|  | **No data on treatment** | | | | | | |
|  | **Received antibiotics** | | | | | | |
|  | **If no antibiotics specify why** | | | | | | |
|  | **Appropriate antibiotics given** | | | | | | |
|  | **Time of appropriate antibiotics** | | | | | | |
|  | **Compound** | | | | | | |
|  | **Empirical antibiotic therapy** | | | | | | |
|  | **Time between presentation to hospital and specific therapy** | | | | | | |
|  | **Time until afebrile after start of appropriate antibiotic therapy** | | | | | | |
|  | **Duration of antibiotic therapy** | | | | | | |
|  | **Dosage of antibiotic therapy** | | | | | | |
|  | **Side effects of treatment** | | | | | | |
|  | **Other treatment** | | | | | | |
|  | **No data on outcome** | | | | | | |
|  | **Complications** | | | | | | |
|  | **Specify complications** | | | | | | |
|  | **Outcome** | | | | | | |
|  | **Specify cause of death** | | | | | | |
|  | **Time from symptom onset to death** | | | | | | |
|  | **Specify sequelae** | | | | | | |
|  | **Comment on relevant / interesting aspects** | | | | | | |
