# Supplementary material for: Human monocytotropic ehrlichiosis—A systematic review and analysis of the literature
Source: PLoS Negl Trop Dis. 2024 Aug 2;18(8):e0012377. doi: 10.1371/journal.pntd.0012377 (PMC11324158; doi:10.1371/journal.pntd.0012377)
Supplement: S5 Text — (DOCX) [file pntd.0012377.s005.docx]

**Included publications**

1. Fishbein DB, Sawyer LA, Holland CJ. Unexplained febrile illnesses after exposure to ticks. Infection with an Ehrlichia? Journal of the American Medical Association. 1987;257(22):3100-4. doi: 10.1001/jama.257.22.3100.

2. Maeda K, Markowitz N, Hawley RC, Ristic M, Cox D, McDade JE. HUMAN INFECTION WITH EHRLICHIA-CANIS, A LEUKOCYTIC RICKETTSIA. New England Journal of Medicine. 1987;316(14):853-6. doi: 10.1056/nejm198704023161406. PubMed PMID: WOS:A1987G606200006.

3. Edwards MS, Jones JE, Leass DL, Whitmore JW, Dawson JE, Fishbein DB. Childhood infection caused by Ehrlichia canis or a closely related organism. Pediatr Infect Dis J. 1988;7(9):651-4. Epub 1988/09/01. doi: 10.1097/00006454-198809000-00010. PubMed PMID: 3050859.

4. Pearce CJ, Conrad ME, Nolan PE, Fishbein DB, Dawson JE. EHRLICHIOSIS - A CAUSE OF BONE-MARROW HYPOPLASIA IN HUMANS. American Journal of Hematology. 1988;28(1):53-5. doi: 10.1002/ajh.2830280111. PubMed PMID: WOS:A1988N344300010.

5. Taylor JP, Betz TG, Fishbein DB, Roberts MA, Dawson J, Ristic M. Serological evidence of possible human infection with Ehrlichia in Texas. J Infect Dis. 1988;158(1):217-20. Epub 1988/07/01. doi: 10.1093/infdis/158.1.217. PubMed PMID: 3392415.

6. Barton LL, Foy TM. Ehrlichia canis infection in a child. Pediatrics. 1989;84(3):580-2. Epub 1989/09/01. PubMed PMID: 2771561.

7. Dimmitt DC, Fishbein DB, Dawson JE. HUMAN EHRLICHIOSIS ASSOCIATED WITH CEREBROSPINAL-FLUID PLEOCYTOSIS - A CASE-REPORT. American Journal of Medicine. 1989;87(6):677-8. PubMed PMID: WOS:A1989CC93400014.

8. Doran TI, Parmley RT, Logas PC, Chamblin S. Infection with Ehrlichia canis in a child. J Pediatr. 1989;114(5):809-12. Epub 1989/05/01. doi: 10.1016/s0022-3476(89)80143-x. PubMed PMID: 2715894.

9. Fishbein DB, Kemp A, Dawson JE, Greene NR, Redus MA, Fields DH. HUMAN EHRLICHIOSIS - PROSPECTIVE ACTIVE SURVEILLANCE IN FEBRILE HOSPITALIZED-PATIENTS. Journal of Infectious Diseases. 1989;160(5):803-9. doi: 10.1093/infdis/160.5.803. PubMed PMID: WOS:A1989AY21800010.

10. Golden SE. Aseptic meningitis associated with Ehrlichia canis infection. Pediatr Infect Dis J. 1989;8(5):335-7. Epub 1989/05/01. PubMed PMID: 2726324.

11. Harkess JR. EHRLICHIOSIS - A CAUSE OF BONE-MARROW HYPOPLASIA IN HUMANS. American Journal of Hematology. 1989;30(4):265-6. doi: 10.1002/ajh.2830300417. PubMed PMID: WOS:A1989T677500016.

12. Manian FA, Weidner J, Costello J, Fishbein DB, Dawson JE. Human ehrlichiosis. Mo Med. 1989;86(10):691-5. Epub 1989/10/01. PubMed PMID: 2677657.

13. Petersen LR, Sawyer LA, Fishbein DB, Kelley PW, Thomas RJ, Magnarelli LA, et al. An outbreak of ehrlichiosis in members of an army reserve unit exposed to ticks. Journal of Infectious Diseases. 1989;159(3):562-8.

14. Raad I, Singh V, Quan TJ. Concurrent positive serology for ehrlichiosis and Lyme disease. J Infect Dis. 1989;160(4):727-8. Epub 1989/10/01. doi: 10.1093/infdis/160.4.727-a. PubMed PMID: 2794568.

15. Simmons BP, Hughey JR. Ehrlichia in Tennessee. South Med J. 1989;82(5):669. Epub 1989/05/01. doi: 10.1097/00007611-198905000-00038. PubMed PMID: 2717999.

16. Barton L, Dawson JE, Letson GW, Luisiri A, Scalzo AJ. Simultaneous ehrlichiosis and Lyme disease. Pediatric Infectious Disease Journal. 1990;9(2):127-9.

17. Ende M, O'Donnal PM, Ende FI, Ende M. Ehrlichiosis in Virginia: case reports. Va Med. 1990;117(4):160-1. Epub 1990/04/01. PubMed PMID: 2349832.

18. Eng TR, Harkess JR, Fishbein DB, Dawson JE, Greene CN, Redus MA, et al. EPIDEMIOLOGIC, CLINICAL, AND LABORATORY FINDINGS OF HUMAN EHRLICHIOSIS IN THE UNITED-STATES, 1988. Jama-Journal of the American Medical Association. 1990;264(17):2251-8. doi: 10.1001/jama.264.17.2251. PubMed PMID: WOS:A1990EF48800031.

19. Harkess JR, Stucky D, Ewing SA. Neurologic abnormalities in a patient with human ehrlichiosis. South Med J. 1990;83(11):1341-3. Epub 1990/11/01. doi: 10.1097/00007611-199011000-00031. PubMed PMID: 2237570.

20. Abbott KC, Vukelja SJ, Smith CE, McAllister CK, Konkol KA, Orourke TJ, et al. HEMOPHAGOCYTIC SYNDROME - A CAUSE OF PANCYTOPENIA IN HUMAN EHRLICHIOSIS. American Journal of Hematology. 1991;38(3):230-4. doi: 10.1002/ajh.2830380315. PubMed PMID: WOS:A1991GL86300014.

21. Dumler JS, Brouqui P, Aronson J, Taylor JP, Walker DH. Identification of Ehrlichia in human tissue [6]. New England Journal of Medicine. 1991;325(15):1109-10.

22. Ghorbel A, Kennou MF, Benhamed S, Benjemaa M, Vidor E. HUMAN EHRLICHIOSIS IN TUNISIA - PRELIMINARY-STUDY. Medecine Et Maladies Infectieuses. 1991;21(12):725-31. doi: 10.1016/s0399-077x(05)81422-2. PubMed PMID: WOS:A1991HB42600002.

23. Gilson I, Jones P. Human ehrlichiosis diagnosed in Wisconsin. Wis Med J. 1991;90(5):219-21. Epub 1991/05/01. PubMed PMID: 1897237.

24. Harkess JR, Ewing SA, Brumit T, Mettry CR. Ehrlichiosis in children. Pediatrics. 1991;87(2):199-203. Epub 1991/02/01. PubMed PMID: 1987531.

25. Malpass DG, Heiman HS, Sumaya CV. Childhood Ehrlichiosis: A case report and review of the literature. International Pediatrics. 1991;6(4):354-8.

26. Morais JD, Dawson JE, Greene C, Filipe AR, Galhardas LC, Bacellar F. First European case of ehrlichiosis. Lancet. 1991;338(8767):633-4. Epub 1991/09/07. doi: 10.1016/0140-6736(91)90644-5. PubMed PMID: 1679172.

27. Moskovitz M, Fadden R, Min T. HUMAN EHRLICHIOSIS - A RICKETTSIAL DISEASE ASSOCIATED WITH SEVERE CHOLESTASIS AND MULTISYSTEMIC DISEASE. Journal of Clinical Gastroenterology. 1991;13(1):86-90. doi: 10.1097/00004836-199102000-00019. PubMed PMID: WOS:A1991EV98200019.

28. Armstrong RW. Ehrlichiosis in a visitor to Virginia. West J Med. 1992;157(2):182-4. Epub 1992/08/01. PubMed PMID: 1441482; PubMed Central PMCID: PMCPMC1011252.

29. Dunn BE, Monson TP, Dumler JS, Morris CC, Westbrook AB, Duncan JL, et al. IDENTIFICATION OF EHRLICHIA-CHAFFEENSIS MORULAE IN CEREBROSPINAL-FLUID MONONUCLEAR-CELLS. Journal of Clinical Microbiology. 1992;30(8):2207-10. doi: 10.1128/jcm.30.8.2207-2210.1992. PubMed PMID: WOS:A1992JD59400061.

30. Hammill WW, Wilson MB, Reigart JR, Flick JT, Laver J. Ehrlichia canis infection in a child in South Carolina. Clin Pediatr (Phila). 1992;31(7):432-4. Epub 1992/07/01. doi: 10.1177/000992289203100710. PubMed PMID: 1319878.

31. Rathore MH. INFECTION DUE TO EHRLICHIA-CANIS IN CHILDREN. Southern Medical Journal. 1992;85(7):703-5. doi: 10.1097/00007611-199207000-00008. PubMed PMID: WOS:A1992JD78600008.

32. Uhaa IJ, Maclean JD, Greene CR, Fishbein DB. A CASE OF HUMAN EHRLICHIOSIS ACQUIRED IN MALI - CLINICAL AND LABORATORY FINDINGS. American Journal of Tropical Medicine and Hygiene. 1992;46(2):161-4. doi: 10.4269/ajtmh.1992.46.161. PubMed PMID: WOS:A1992HH01400010.

33. Dumler JS, Sutker WL, Walker DH. PERSISTENT INFECTION WITH EHRLICHIA-CHAFFEENSIS. Clinical Infectious Diseases. 1993;17(5):903-5. doi: 10.1093/clinids/17.5.903. PubMed PMID: WOS:A1993ME52300018.

34. Fichtenbaum CJ, Peterson LR, Weil GJ. Ehrlichiosis presenting as a life-threatening illness with features of the toxic shock syndrome. Am J Med. 1993;95(4):351-7. Epub 1993/10/01. doi: 10.1016/0002-9343(93)90302-6. PubMed PMID: 8213865.

35. Mathisen GE, Weiss PJ, Kennedy CA. PNEUMONIA, ASEPTIC-MENINGITIS, AND LEUKOPENIA IN A 28-YEAR-OLD MAN. Clinical Infectious Diseases. 1993;16(6):809-15. doi: 10.1093/clind/16.6.809. PubMed PMID: WOS:A1993LE42300013.

36. Paddock CD, Suchard DP, Grumbach KL, Hadley WK, Kerschmann RL, Abbey NW, et al. Brief report: Fatal seronegative ehrlichiosis in a patient with HIV infection. New England Journal of Medicine. 1993;329(16):1164-7. doi: 10.1056/NEJM199310143291605.

37. Rathore MH, Meyer K. Human ehrlichiosis in Florida. J Fla Med Assoc. 1993;80(5):327-9. Epub 1993/05/01. PubMed PMID: 8315405.

38. Everett ED, Evans KA, Henry RB, McDonald G. HUMAN EHRLICHIOSIS IN ADULTS AFTER TICK EXPOSURE - DIAGNOSIS USING POLYMERASE CHAIN-REACTION. Annals of Internal Medicine. 1994;120(9):730-5. doi: 10.7326/0003-4819-120-9-199405010-00002. PubMed PMID: WOS:A1994NH25100002.

39. Fishbein DB, Dawson JE, Robinson LE. HUMAN EHRLICHIOSIS IN THE UNITED-STATES, 1985 TO 1990. Annals of Internal Medicine. 1994;120(9):736-43. doi: 10.7326/0003-4819-120-9-199405010-00003. PubMed PMID: WOS:A1994NH25100003.

40. Paparone PW, Glenn WB. Lyme disease with concurrent ehrlichiosis. J Am Osteopath Assoc. 1994;94(7):568-70, 73, 77. Epub 1994/07/01. PubMed PMID: 8083066.

41. Rynkiewicz DL, Liu LX. Human ehrlichiosis in New England [8]. New England Journal of Medicine. 1994;330(4):292-3. doi: 10.1056/NEJM199401273300417.

42. Tisdale JF, Veldkamp P. Ehrlichiosis. J Tenn Med Assoc. 1994;87(11):480-1. Epub 1994/11/01. PubMed PMID: 7983865.

43. Antony SJ, Dummer JS, Hunter E. HUMAN EHRLICHIOSIS IN A LIVER-TRANSPLANT RECIPIENT. Transplantation. 1995;60(8):879-81. doi: 10.1097/00007890-199510270-00021. PubMed PMID: WOS:A1995TC02400021.

44. Hawkins MM. Human ehrlichiosis: a case report from the South Carolina lowcountry. J S C Med Assoc. 1995;91(5):228-9. Epub 1995/05/01. PubMed PMID: 7776625.

45. Marty AM, Dumler JS, Imes G, Brusman HP, Smrkovski LL, Frisman DM. EHRLICHIOSIS MIMICKING THROMBOTIC THROMBOCYTOPENIC PURPURA - CASE-REPORT AND PATHOLOGICAL CORRELATION. Human Pathology. 1995;26(8):920-5. doi: 10.1016/0046-8177(95)90017-9. PubMed PMID: WOS:A1995RN51900018.

46. Paparone PW, Ljubich P, Rosman GA, Nazha NT. Ehrlichiosis with pancytopenia and ARDS. N J Med. 1995;92(6):381-5. Epub 1995/06/01. PubMed PMID: 7617311.

47. Pierard D, Levtchenko E, Dawson JE, Lauwers S. Ehrlichiosis in Belgium. Lancet. 1995;346(8984):1233-4. Epub 1995/11/04. doi: 10.1016/s0140-6736(95)92943-6. PubMed PMID: 7475691.

48. Roland WE, McDonald G, Caldwell CW, Everett ED. Ehrlichiosis - A cause of prolonged fever. Clinical Infectious Diseases. 1995;20(4):821-5.

49. Shea KW, Calio AJ, Klein NC, Cunha BA. Rhabdomyolysis associated with Ehrlichia chaffeensis infection. Clin Infect Dis. 1995;21(4):1056-7. Epub 1995/10/01. doi: 10.1093/clinids/21.4.1056. PubMed PMID: 8645815.

50. Standaert SM, Dawson JE, Schaffner W, Childs JE, Biggie KL, Singleton BS, et al. EHRLICHIOSIS IN A GOLF-ORIENTED RETIREMENT COMMUNITY. New England Journal of Medicine. 1995;333(7):420-5. doi: 10.1056/nejm199508173330704. PubMed PMID: WOS:A1995RP24300004.

51. Tal A, Shannahan D, Fichtenbaum CJ, Weil GJ. Ehrlichiosis presenting as a life-threatening illness. American Journal of Medicine. 1995;98(3):318-9. doi: 10.1016/S0002-9343(99)80388-X.

52. Williams JD, Snow RM, Arciniegas JG. Myocardial involvement in a patient with human ehrlichiosis. Am J Med. 1995;98(4):414-5. Epub 1995/04/01. doi: 10.1016/s0002-9343(99)80324-6. PubMed PMID: 7709955.

53. Barenfanger J, Patel PG, Dumler JS, Walker DH. Pathology Rounds: Identifying Human Ehrlichiosis. Laboratory Medicine. 1996;27(6):372-4. doi: 10.1093/labmed/27.6.372.

54. Cunha BA. Ehrlichiosis. Emergency Medicine (00136654). 1996;28(4):87-91. PubMed PMID: 107347422. Language: English. Entry Date: 19971201. Revision Date: 20150711. Publication Type: Journal Article.

55. Naqvi SH, Barry RC, Friedman AD, St. Louis F. Ehrlichiosis: An infectious cause of severe leukopenia. International Journal of Pediatric Hematology/Oncology. 1996;3(1):1-4.

56. Silvers LE, Watkins S, Strickland GT, Clothier M, Grant J, Hall E, et al. Human ehrlichiosis - Maryland, 1994 (Reprinted from MMWR, vol 45, pg 798-802, 1996). Jama-Journal of the American Medical Association. 1996;276(15):1212-3. PubMed PMID: WOS:A1996VL69100012.

57. Stith DM, Telford Iii SR, Dawson JE. Diagnosing ehrlichiosis [1]. Annals of Internal Medicine. 1996;124(9):854-5. doi: 10.7326/0003-4819-124-9-199605010-00012.

58. Vanek NN, Kazi S, Cepero NM, Tang S, Rex JH. Human ehrlichiosis causing left ventricular dilatation and dysfunction. Clin Infect Dis. 1996;22(2):386-7. Epub 1996/02/01. doi: 10.1093/clinids/22.2.386. PubMed PMID: 8838212.

59. Vugia DJ, Holmberg E, Steffe EM, Ascher MS, Gallo D. A human case of monocytic ehrlichiosis with adult respiratory distress syndrome in northern California. West J Med. 1996;164(6):525-8. Epub 1996/06/01. PubMed PMID: 8764635; PubMed Central PMCID: PMCPMC1303637.

60. Carter N, Miller NR. Fourth nerve palsy caused by Ehrlichia chaffeensis. J Neuroophthalmol. 1997;17(1):47-50. Epub 1997/03/01. PubMed PMID: 9093962.

61. Devereaux CE. Human monocytic ehrlichiosis presenting as febrile diarrhea. J Clin Gastroenterol. 1997;25(3):544-5. Epub 1997/12/31. doi: 10.1097/00004836-199710000-00014. PubMed PMID: 9412975.

62. Friedman AD, Daniel GK, Qureshi WA. Systemic ehrlichiosis presenting as progressive hepatosplenomegaly. South Med J. 1997;90(6):656-60. Epub 1997/06/01. doi: 10.1097/00007611-199706000-00017. PubMed PMID: 9191748.

63. Grant AC, Hunter S, Partin WC. A case of acute monocytic ehrlichiosis with prominent neurologic signs. Neurology. 1997;48(6):1619-23. Epub 1997/06/01. doi: 10.1212/wnl.48.6.1619. PubMed PMID: 9191777.

64. Jackson RT, Jackson JW. Ehrlichiosis with systemic sepsis syndrome. Tenn Med. 1997;90(5):185-6. Epub 1997/05/01. PubMed PMID: 9130874.

65. Laudicina RJ, Hilger AE. Focus: tick-borne diseases. Human Ehrlichiosis: a case review. Clinical Laboratory Science. 1997;10(3):149-69. PubMed PMID: 107238109. Language: English. Entry Date: 19980201. Revision Date: 20150820. Publication Type: Journal Article.

66. Paddock CD, Sumner JW, Shore GM, Bartley DC, Elie RC, McQuade JG, et al. Isolation and characterization of Ehrlichia chaffeensis strains from patients with fatal ehrlichiosis. J Clin Microbiol. 1997;35(10):2496-502. Epub 1997/10/08. doi: 10.1128/jcm.35.10.2496-2502.1997. PubMed PMID: 9316896; PubMed Central PMCID: PMCPMC229999.

67. Trotta RF, Hospenthal DR, Bennett SP, Daniels AM, Fishbain JT. Human monocytic ehrlichiosis with concurrent Lyme antibody seroconversion. Infectious Diseases in Clinical Practice. 1997;6(6):401-5. doi: 10.1097/00019048-199706060-00010.

68. Fordham LA, Chung CJ, Specter BB, Merten DF, Ingram DL. Ehrlichiosis: findings on chest radiographs in three pediatric patients. AJR Am J Roentgenol. 1998;171(5):1421-4. Epub 1998/11/03. doi: 10.2214/ajr.171.5.9798890. PubMed PMID: 9798890.

69. Heilpern KL. Update: Human ehrlichiosis - Maryland and Wisconsin, 1994 - Commentary. Annals of Emergency Medicine. 1998;32(1):109-10. PubMed PMID: WOS:000074489600022.

70. Sexton DJ, Walker DH. Dual infection with Ehrlichia chaffeensis and a spotted fever group rickettsia: A case report - Reply to Dr. Sulzer. Emerging Infectious Diseases. 1998;4(4):706-. doi: 10.3201/eid0404.980431. PubMed PMID: WOS:000077532500031.

71. Standaert SM, Clough LA, Schaffner W, Adams JS, Neuzil KM. Neurologic manifestations of human monocytic ehrlichiosis. Infectious Diseases in Clinical Practice. 1998;7(7):358-62. doi: 10.1097/00019048-199809003-00014.

72. Sulkowski MS, Dumler JS. Clinical spectrum of disease due to Ehrlichia chaffeensis: Case reports and review. Infectious Diseases in Clinical Practice. 1998;7(6):252-6. doi: 10.1097/00019048-199808000-00002.

73. Berry DS, Miller RS, Hooke JA, Massung RF, Bennett J, Ottolini MG. Ehrlichial meningitis with cerebrospinal fluid morulae. Pediatr Infect Dis J. 1999;18(6):552-5. Epub 1999/07/03. doi: 10.1097/00006454-199906000-00016. PubMed PMID: 10391189.

74. Buller RS, Arens M, Hmiel SP, Paddock CD, Sumner JW, Rikihisa Y, et al. Ehrlichia ewingii, a newly recognized agent of human ehrlichiosis. New England Journal of Medicine. 1999;341(3):148-55. doi: 10.1056/NEJM199907153410303.

75. Carpenter CF, Gandhi TK, Kong LK, Corey GR, Chen SM, Walker DH, et al. The incidence of ehrlichial and rickettsial infection in patients with unexplained fever and recent history of tick bite in central North Carolina. J Infect Dis. 1999;180(3):900-3. Epub 1999/08/07. doi: 10.1086/314954. PubMed PMID: 10438390.

76. Gongora-Biachi RA, Zavala-Velazquez J, Castro-Sansores CJ, Gonzalez- Martinez P. First case of human Ehrlichiosis in Mexico [1]. Emerging Infectious Diseases. 1999;5(3):481. doi: 10.3201/eid0503.990327.

77. Jerrard D. Ehrlichiosis. J Emerg Med. 1999;17(1):27-30. Epub 1999/02/09. doi: 10.1016/s0736-4679(98)00117-6. PubMed PMID: 9950382.

78. Martin GS, Christman BW, Standaert SM. Rapidly fatal infection with Ehrlichia chaffeensis [2]. New England Journal of Medicine. 1999;341(10):763-4. doi: 10.1056/NEJM199909023411014.

79. Nutt AK, Raufman JP. Gastrointestinal and hepatic manifestations of human ehrlichiosis: 8 Cases and a review of the literature. Digestive Diseases. 1999;17(1):37-43. doi: 10.1159/000016901.

80. Patel RG, Byrd MA, editors. Near fatal acute respiratory distress syndrome in a patient with human ehrlichiosis. Southern Medical Journal; 1999.

81. Roundtree SE, Nixon GA. Human monocytic ehrlichiosis in a thirteen year old: a case report. J S C Med Assoc. 1999;95(8):300-2. Epub 1999/09/09. PubMed PMID: 10478517.

82. Sadikot R, Shaver MJ, Reeves WB. Ehrlichia chaffeensis in a renal transplant recipient. Am J Nephrol. 1999;19(6):674-6. Epub 1999/12/11. doi: 10.1159/000013540. PubMed PMID: 10592362.

83. Scaglia F, Vogler LB, Hymes LC, Maki Jr A. Minimal change nephrotic syndrome: A possible complication of ehrlichiosis. Pediatric Nephrology. 1999;13(7):600-1. doi: 10.1007/s004670050666.

84. Weaver RAR, Virella G, Weaver A. Ehrlichiosis with severe pulmonary manifestations despite early treatment. Southern Medical Journal. 1999;92(3):336-9. doi: 10.1097/00007611-199903000-00018.

85. Whitt SP, Everett ED, Roland W, Dolan S. Ehrlichia chaffeensis-Associated cardiomyopathy in a patient with AIDS. Clinical Infectious Diseases. 1999;28(1):140. doi: 10.1086/515092.

86. Arav-Boger R, Knepp JH, Walls JJ, Dumler JS. Human monocytic ehrlichiosis in a child with leukemia. Pediatr Infect Dis J. 2000;19(2):173-5. Epub 2000/02/29. doi: 10.1097/00006454-200002000-00023. PubMed PMID: 10694014.

87. Bicknell S, Mason A. Acute respiratory failure due to ehrlichiosis - CT findings: Case report. Canadian Association of Radiologists Journal. 2000;51(5):300-1.

88. Donohue T, Cawley JF. Case study: ehrlichiosis: a new and emerging infectious disease. Physician Assistant. 2000;24(8):17-26. PubMed PMID: 107136341. Language: English. Entry Date: 20001001. Revision Date: 20150818. Publication Type: Journal Article.

89. Dumler JS, Dey C, Meier F, Lewis LL. Human monocytic ehrlichiosis: a potentially severe disease in children. Arch Pediatr Adolesc Med. 2000;154(8):847-9. Epub 2000/08/02. doi: 10.1001/archpedi.154.8.847. PubMed PMID: 10922287.

90. Keysary A, Waner T. Serologic evidence of human monocytic and granulocytic ehrlichiosis in Israel. Emerging Infectious Diseases. 2000;6(3):315-. doi: 10.3201/eid0603.000317. PubMed PMID: WOS:000087321300017.

91. Peters TR, Edwards KM, Standaert SM. Severe ehrlichiosis in an adolescent taking trimethoprim-sulfamethoxazole. Pediatr Infect Dis J. 2000;19(2):170-2. Epub 2000/02/29. doi: 10.1097/00006454-200002000-00021. PubMed PMID: 10694012.

92. Pick N, Potasman I, Strenger C, Keysary A, Schwartz I. Ehrlichiosis associated vasculitis. J Intern Med. 2000;247(6):674-8. Epub 2000/07/08. doi: 10.1046/j.1365-2796.2000.00680.x. PubMed PMID: 10886489.

93. Standaert SM, Yu T, Scott MA, Childs JE, Paddock CD, Nicholson WL, et al. Primary isolation of Ehrlichia chaffeensis from patients with febrile illnesses: Clinical and molecular characteristics. Journal of Infectious Diseases. 2000;181(3):1082-8. doi: 10.1086/315346.

94. Weeks MG. Ehrlichiosis ... in Hawaii? An unusual presentation. Hawaii Med J. 2000;59(5):182-3. Epub 2000/06/01. PubMed PMID: 10832209.

95. Brantley RK. Trimethoprim-sulfamethoxazole and fulminant ehrlichiosis [1]. Pediatric Infectious Disease Journal. 2001;20(2):231. doi: 10.1097/00006454-200102000-00028.

96. Dawson JE, Paddock CD, Warner CK, Greer PW, Bartlett JH, Ewing SA, et al. Tissue diagnosis of Ehrlichia chaffeensis in patients with fatal ehrlichiosis by use of immunohistochemistry, in situ hybridization, and polymerase chain reaction. Am J Trop Med Hyg. 2001;65(5):603-9. Epub 2001/11/22. doi: 10.4269/ajtmh.2001.65.603. PubMed PMID: 11716122.

97. Javed MZ, Srivastava M, Zhang S, Kandathil M. Concurrent babesiosis and ehrlichiosis in an elderly host. Mayo Clin Proc. 2001;76(5):563-5. Epub 2001/05/19. doi: 10.4065/76.5.563. PubMed PMID: 11357805.

98. Rooney TB, McGue TE, Delahanty KC, Rooney TB, McGue TE, Delahanty KC. A Naval Academy midshipman with ehrlichiosis after summer field exercises in Quantico, Virginia. Military Medicine. 2001;166(2):191-3. PubMed PMID: 107029216. Language: English. Entry Date: 20010608. Revision Date: 20171209. Publication Type: journal article.

99. Tan HP, Dumler JS, Maley WR, Klein AS, Burdick JF, Poordad FF, et al. Human monocytic ehrlichiosis: An emerging pathogen in transplantation. Transplantation. 2001;71(11):1678-80. doi: 10.1097/00007890-200106150-00030. PubMed PMID: WOS:000169420900030.

100. Liddell AM, Sumner JW, Paddock CD, Rikihisa Y, Unver A, Buller RS, et al. Reinfection with Ehrlichia chaffeensis in a liver transplant recipient. Clin Infect Dis. 2002;34(12):1644-7. Epub 2002/05/29. doi: 10.1086/340523. PubMed PMID: 12032902.

101. Safdar N, Love RB, Maki DG. Severe Ehrlichia chaffeensis infection in a lung transplant recipient: a review of ehrlichiosis in the immunocompromised patient. Emerg Infect Dis. 2002;8(3):320-3. Epub 2002/04/03. doi: 10.3201/eid0803.010249. PubMed PMID: 11927032; PubMed Central PMCID: PMCPMC2732464.

102. Saunders A. Diagnosing human ehrlichiosis. Physician Assistant. 2002;26(6):44-50. PubMed PMID: 106958717. Language: English. Entry Date: 20020913. Revision Date: 20150818. Publication Type: Journal Article.

103. Sehdev AES, Sehdev PS, Jacobs R, Dumler JS. Human monocytic ehrlichiosis presenting as acute appendicitis during pregnancy. Clinical Infectious Diseases. 2002;35(9):E99-E102. doi: 10.1086/342887. PubMed PMID: WOS:000178641200025.

104. Westerman EL. Chills, fever, and myalgia in a farmer. Infections in medicine. 2002;19(6):252.

105. Olano JP, Hogrefe W, Seaton B, Walker DH. Clinical manifestations, epidemiology, and laboratory diagnosis of human monocytotropic ehrlichiosis in a commercial laboratory setting. Clin Diagn Lab Immunol. 2003;10(5):891-6. Epub 2003/09/11. doi: 10.1128/cdli.10.5.891-896.2003. PubMed PMID: 12965923; PubMed Central PMCID: PMCPMC193882.

106. Olano JR, Masters E, Hogrefe W, Walker DH. Human monocytotropic ehrlichiosis, Missouri. Emerging Infectious Diseases. 2003;9(12):1579-86. doi: 10.3201/eid0912.020733. PubMed PMID: WOS:000187247600012.

107. Talbot TR, Comer JA, Bloch KC. Ehrlichia chaffeensis infections among HIV-infected patients in a human monocytic ehrlichiosis-endemic area. Emerg Infect Dis. 2003;9(9):1123-7. Epub 2003/10/02. doi: 10.3201/eid0909.020560. PubMed PMID: 14519250; PubMed Central PMCID: PMCPMC3016774.

108. Calic SB, Galvão MA, Bacellar F, Rocha CM, Mafra CL, Leite RC, et al. Human ehrlichioses in Brazil: first suspect cases. Braz J Infect Dis. 2004;8(3):259-62. Epub 2004/10/12. doi: 10.1590/s1413-86702004000300011. PubMed PMID: 15476059.

109. Hamilton KS, Standaert SM, Kinney MC. Characteristic peripheral blood findings in human ehrlichiosis. Mod Pathol. 2004;17(5):512-7. Epub 2004/02/21. doi: 10.1038/modpathol.3800075. PubMed PMID: 14976527.

110. Rowe SM, Wille KM. Severe acute human monocytic ehrlichiosis associated with trimethoprim-sulfamethoxazole therapy. Chest. 2004;126(4):966S-7S. doi: 10.1378/chest.126.4_MeetingAbstracts.966S. PubMed PMID: WOS:000224731400745.

111. Stone JH, Dierberg K, Aram G, Dumler JS, Stone JH, Dierberg K, et al. Human monocytic ehrlichiosis. JAMA: Journal of the American Medical Association. 2004;292(18):2263-70. doi: 10.1001/jama.292.18.2263. PubMed PMID: 106540679. Language: English. Entry Date: 20051118. Revision Date: 20200708. Publication Type: journal article.

112. Fritz CL, Bronson LR, Smith CR, Crawford-Miksza L, Yeh E, Schnurr D. Clinical, epidemiologic, and environmental surveillance for ehrlichiosis and anaplasmosis in an endemic area of northern California. J Vector Ecol. 2005;30(1):4-10. Epub 2005/07/13. PubMed PMID: 16007950.

113. Louw M, Allsopp M, Meyer EC. Ehrlichia ruminantium, an emerging human pathogen a further report. Samj South African Medical Journal. 2005;95(12):948-. PubMed PMID: WOS:000234362800020.

114. Malani A, Weigand R, Gupta V, Hertzberg L, Rangineni G. Ehrlichiosis Mimicking T-Cell Lymphoma/Leukemia. Blood. 2005;106(11):4343-. doi: 10.1182/blood.V106.11.4343.4343.

115. Ochoa WG, Wedro BC, Firary SA. Images in emergency medicine. Human ehrlichiosis. Ann Emerg Med. 2005;46(5):470, 8. Epub 2005/11/08. doi: 10.1016/j.annemergmed.2005.06.008. PubMed PMID: 16271680.

116. Chapman AS. Diagnosis and management of tickborne rickettsial diseases: Rocky Mountain spotted fever, Ehrlichioses, and anaplasmosis -- United States: a practical guide for physicians and other health-care and public health professionals. MMWR: Morbidity & Mortality Weekly Report. 2006;55(RR-4):1-29. PubMed PMID: 106434258. Corporate Author: Tickborne Rickettsial Diseases Working Group. Language: English. Entry Date: 20060505. Revision Date: 20151019. Publication Type: Journal Article.

117. Cotant C, Okulicz J, Brezina B, Riley D, Conger N. Human monocytic ehrlichiosis in a renal transplant patient. Scandinavian Journal of Infectious Diseases. 2006;38(8):699-702. doi: 10.1080/00365540500444694.

118. da Costa PSG, de Carvalho Valle LM, Brigatte ME, Greco DB. More about human monocytotropic ehrlichiosis in Brazil: Serological evidence of nine new cases. Brazilian Journal of Infectious Diseases. 2006;10(1):7-10. doi: 10.1590/S1413-86702006000100002.

119. Pendse S, Bilyk JR, Lee MS. The ticking time bomb. Surv Ophthalmol. 2006;51(3):274-9. Epub 2006/04/29. doi: 10.1016/j.survophthal.2006.02.004. PubMed PMID: 16644367.

120. Perez M, Bodor M, Zhang C, Xiong Q, Rikihisa Y, editors. Human infection with Ehrlichia canis accompanied by clinical signs in Venezuela. Annals of the New York Academy of Sciences; 2006.

121. Schutze GE. Ehrlichiosis. Pediatr Infect Dis J. 2006;25(1):71-2. Epub 2006/01/06. doi: 10.1097/01.inf.0000196921.97085.b0. PubMed PMID: 16395107.

122. Prince LK, Shah AA, Martinez LJ, Moran KA. Ehrlichiosis: making the diagnosis in the acute setting. South Med J. 2007;100(8):825-8. Epub 2007/08/24. doi: 10.1097/SMJ.0b013e31804aa1ad. PubMed PMID: 17713310.

123. Thomas LD, Hongo I, Bloch KC, Tang YW, Dummer S. Human ehrlichiosis in transplant recipients. Am J Transplant. 2007;7(6):1641-7. Epub 2007/05/22. doi: 10.1111/j.1600-6143.2007.01821.x. PubMed PMID: 17511689.

124. Young NP, Klein CJ. Encephalopathy with seizures having PCR-positive Anaplasma phagocytophilum and Ehrlichia chaffeensis [2]. European Journal of Neurology. 2007;14(2):e3-e4. doi: 10.1111/j.1468-1331.2006.01582.x.

125. Bariola JR, Bradsher RW. Severe Ehrlichia chaffeensis infection in a patient receiving etanercept, a tumor necrosis factor inhibitor. Infectious Diseases in Clinical Practice. 2008;16(2):124-6. doi: 10.1097/IPC.0b013e3181151217.

126. Martínez MC, Gutiérrez CN, Monger F, Ruiz J, Watts A, Mijares VM, et al. Ehrlichia chaffeensis in child, Venezuela. Emerg Infect Dis. 2008;14(3):519-20. Epub 2008/03/08. doi: 10.3201/eid1403.061304. PubMed PMID: 18325283; PubMed Central PMCID: PMCPMC2570823.

127. Reeves WK, Loftis AD, Nicholson WL, Czarkowski AG. The first report of human illness associated with the Panola Mountain Ehrlichia species: a case report. J Med Case Rep. 2008;2:139. Epub 2008/05/02. doi: 10.1186/1752-1947-2-139. PubMed PMID: 18447934; PubMed Central PMCID: PMCPMC2396651.

128. Heffner A, Nestor J. Severe sepsis and multi-system organ failure due to human monocytic ehrlichiosis. Critical Care Medicine. 2009;37(12):A514. doi: 10.1097/01.ccm.0000365439.11849.a2.

129. Lawrence KL, Morrell MR, Storch GA, Hachem RR, Trulock EP. Clinical outcomes of solid organ transplant recipients with ehrlichiosis. Transpl Infect Dis. 2009;11(3):203-10. Epub 2009/02/21. doi: 10.1111/j.1399-3062.2009.00373.x. PubMed PMID: 19228344.

130. Masters EJ, Storch GA, Sumner JW. Ehrlichia ewingii in an immunocompetent adult. Mo Med. 2009;106(4):301-3. Epub 2009/09/17. PubMed PMID: 19753925.

131. Ndip LM, Labruna M, Ndip RN, Walker DH, McBride JW. Molecular and clinical evidence of Ehrlichia chaffeensis infection in Cameroonian patients with undifferentiated febrile illness. Ann Trop Med Parasitol. 2009;103(8):719-25. Epub 2009/12/25. doi: 10.1179/000349809x12554106963753. PubMed PMID: 20030996; PubMed Central PMCID: PMCPMC2913319.

132. Schneider JG. Human ehrlichiosis: a case study. Clin Lab Sci. 2009;22(1):3-8. Epub 2009/04/10. PubMed PMID: 19354021.

133. Stoecker WV, Calcara DA, Malters JM, Clonts M, Everett ED. Tick-borne febrile illnesses lacking specific symptoms. Mo Med. 2009;106(4):304-8. Epub 2009/09/17. PubMed PMID: 19753926.

134. Villavicencio R, Munro A. HUMAN MONOCYTOTROPIC EHRLICHIOSIS: AN EVASIVE PATHOGEN OF WOODLAND AMERICA. Journal of General Internal Medicine. 2009;24:319-. PubMed PMID: WOS:000265382000843.

135. Burns S, Saylors R, Mian A. Hemophagocytic lymphohistiocytosis secondary to Ehrlichia chaffeensis infection: a case report. J Pediatr Hematol Oncol. 2010;32(4):e142-3. Epub 2010/03/13. doi: 10.1097/MPH.0b013e3181c80ab9. PubMed PMID: 20224442.

136. Esbenshade A, Esbenshade J, Domm J, Williams J, Frangoul H. Severe ehrlichia infection in pediatric oncology and stem cell transplant patients. Pediatr Blood Cancer. 2010;54(5):776-8. Epub 2010/01/07. doi: 10.1002/pbc.22392. PubMed PMID: 20052776.

137. Hanson D, Walter A, Powell J. Ehrlichia-induced hemophagocytic lymphohistiocytosis in two children. Pediatric Blood and Cancer. 2010;54(6):829-30. doi: 10.1002/pbc.22516.

138. Hughes JM, Wille KM. When macrophages attack. American Journal of Respiratory and Critical Care Medicine. 2010;181(1).

139. Kalra A, Gajera MJ, Shah N, Fraimow H. Cardiac tamponade as manifestation of rickettsial infection. Chest. 2010;138(4). doi: 10.1378/chest.9979.

140. Kendra PE, Vazquez-Kendra P. A CASE HISTORY OF CONCURRENT ROCKY MOUNTAIN SPOTTED FEVER AND HUMAN MONOCYTIC EHRLICHIOSIS IN FLORIDA. Florida Entomologist. 2010;93(3):457-9. doi: 10.1653/024.093.0324. PubMed PMID: WOS:000281612500023.

141. Yachoui R, Sabbah M, Subhi A. Multiorgan failure related to human monocytic ehrlichiosis. Journal of Hospital Medicine. 2010;5:197. doi: 10.1002/jhm.709.

142. Hanson D, Walter AW, Powell J. Ehrlichia-induced hemophagocytic lymphohistiocytosis in two children. Pediatr Blood Cancer. 2011;56(4):661-3. Epub 2011/02/08. doi: 10.1002/pbc.22814. PubMed PMID: 21298756.

143. Jose A. ARDS as a potentially avoidable complication of ehrlichiosis infection. Journal of General Internal Medicine. 2011;26:S524-S5. doi: 10.1007/s11606-011-1730-9.

144. Pavelites JJ, Prahlow JA. Fatal human monocytic ehrlichiosis: a case study. Forensic Sci Med Pathol. 2011;7(3):287-93. Epub 2011/02/01. doi: 10.1007/s12024-010-9219-0. PubMed PMID: 21279705.

145. Pritt BS, Sloan LM, Johnson DKH, Munderloh UG, Paskewitz SM, McElroy KM, et al. Emergence of a New Pathogenic Ehrlichia Species, Wisconsin and Minnesota, 2009. New England Journal of Medicine. 2011;365(5):422-9. doi: 10.1056/NEJMoa1010493. PubMed PMID: WOS:000293447800006.

146. Stanojevic D, De Nazareth AG, Wichman T. A man with generalized fatigue and malaise. Journal of General Internal Medicine. 2011;26:S551. doi: 10.1007/s11606-011-1730-9.

147. Watson ME, Jr., Storch GA, Dunne WM, Jr., Burnham CA. A 2-year-old female with Fever and rash. J Clin Microbiol. 2011;49(7):2389, 784. Epub 2011/07/02. doi: 10.1128/jcm.00081-11. PubMed PMID: 21719732; PubMed Central PMCID: PMCPMC3147883.

148. Badireddi S, Joshi M. Hemophagocytic lymphohistiocytosis: An unreported complication of ehrlichiosis in adults. Chest. 2012;142(4). doi: 10.1378/chest.1390907.

149. Chug L, Mahayni T, Hamblin M. Human ehrlichiosis: Severe pulmonary manifestations in a patient with human immunodeficiency virus. Critical Care Medicine. 2012;40(12):152. doi: 10.1097/01.ccm.0000425605.04623.4b.

150. Cunha BA, Chandrankunnel JG, Hage JE. Ehrlichia chaffeensis human monocytic ehrlichiosis with pancytopenia. Scand J Infect Dis. 2012;44(6):473-4. Epub 2012/04/14. doi: 10.3109/00365548.2011.633550. PubMed PMID: 22497260.

151. Cunha BA, Petelin A, Hage JE. Ehrlichia chaffeensis presenting with bilateral anterior thigh pain (Louria's sign). Travel Med Infect Dis. 2012;10(5-6):267-9. Epub 2012/04/24. doi: 10.1016/j.tmaid.2012.03.010. PubMed PMID: 22520448.

152. Duthuluru S, Saad A, Crosser M. Myocarditis associated with human monocytic ehrlichiosis in a 40-year old woman. American Journal of Respiratory and Critical Care Medicine. 2012;185.

153. Havens NS, Kinnear BR, Mató S. Fatal ehrlichial myocarditis in a healthy adolescent: a case report and review of the literature. Clin Infect Dis. 2012;54(8):e113-4. Epub 2012/02/23. doi: 10.1093/cid/cis032. PubMed PMID: 22354925.

154. Ismail N, Walker DH, Ghose P, Tang YW. Immune mediators of protective and pathogenic immune responses in patients with mild and fatal human monocytotropic ehrlichiosis. BMC Immunol. 2012;13:26. Epub 2012/05/23. doi: 10.1186/1471-2172-13-26. PubMed PMID: 22607204; PubMed Central PMCID: PMCPMC3517396.

155. Kane ID, Fish FA, Edwards KM. Ehrlichia myocarditis in an adolescent. Infectious Diseases in Clinical Practice. 2012;20(3):226-8. doi: 10.1097/IPC.0b013e318248f168.

156. Mancuso AM, Bertagnolli JF, Shadiack E, De Antonio SM. An Unusual Case of Transient Psychosis from Ehrlichia Infection. Osteopathic Family Physician. 2012;4(4):124-7. doi: 10.1016/j.osfp.2011.07.005.

157. Montes-Farah J, De la Vega-del Risco F, Bello-Espinosa A, Fortich-Salvador AS. COINFECCIÓN DE BABESIOSIS Y EHRLICHIOSIS:UN CASO EN CARTAGENA DE INDIAS, COLOMBIA. Revista ciencias biomédicas. 2012;3(2):339-45.

158. Zamanifekri B, Riel-Romero RMS, Schwendimann R, Davis DE. Neurologic manifestation of ehrlichiosis: Case report and review of literature. Journal of Child Neurology. 2012;27(7):963. doi: 10.1177/0883073812448439.

159. Kumar N, Goyal J, Goel A, Shakoory B, Chatham W. Macrophage Activation Syndrome Secondary to Human Monocytic Ehrlichiosis. Indian Journal of Hematology and Blood Transfusion. 2013;30(1):145-7. doi: 10.1007/s12288-013-0299-3.

160. Nayak SU, Simon GL. Myocarditis after trimethoprim/sulfamethoxazole treatment for ehrlichiosis. Emerg Infect Dis. 2013;19(12):1975-7. Epub 2013/11/28. doi: 10.3201/eid1912.121459. PubMed PMID: 24274783; PubMed Central PMCID: PMCPMC3840877.

161. Regan J, Matthias J, Green-Murphy A, Stanek D, Bertholf M, Pritt BS, et al. A confirmed Ehrlichia ewingii infection likely acquired through platelet transfusion. Clin Infect Dis. 2013;56(12):e105-7. Epub 2013/03/21. doi: 10.1093/cid/cit177. PubMed PMID: 23511303.

162. Slack D, Emmons W. A case of fulminant ehrlichiosis in the setting of recent trimethoprim-sulfamethoxazole use. Infectious Diseases in Clinical Practice. 2013;21(2):141-4. doi: 10.1097/IPC.0b013e3182699194.

163. Uppalapati A, Gogineni S, Kamel G. Ehrlichiosis-An unusua l cau se of shock and acute inflammatory demyelinating polyneuropathy (AIDP). Critical Care Medicine. 2013;41(12):A316. doi: 10.1097/01.ccm.0000440465.01475.82.

164. Xuefei D, Qin H, Xiaodi G, Zhen G, Wei L, Xuexia H, et al. Epidemiological and clinical features of three clustered cases co-infected with Lyme disease and rickettsioses. Zoonoses Public Health. 2013;60(7):487-93. Epub 2013/01/03. doi: 10.1111/zph.12025. PubMed PMID: 23280084.

165. Allen MB, Pritt BS, Sloan LM, Paddock CD, Musham CK, Ramos JM, et al. First reported case of Ehrlichia ewingii involving human bone marrow. J Clin Microbiol. 2014;52(11):4102-4. Epub 2014/09/05. doi: 10.1128/jcm.01670-14. PubMed PMID: 25187638; PubMed Central PMCID: PMCPMC4313272.

166. Arsić B, Gligić A, Ristanović E, Lako B, Potkonjak A, Perunicić M, et al. A case of human monocytic ehrlichiosis in Serbia. Srp Arh Celok Lek. 2014;142(1-2):79-82. Epub 2014/04/02. doi: 10.2298/sarh1402079a. PubMed PMID: 24684037.

167. Beavers C, Rickard K, W. Snyder J, C. Hollensead S. Two fatal Ehrlichia cases with complete autopsies. JMM Case Reports. 2014;1(1). doi: https://doi.org/10.1099/jmmcr.0.000349.

168. Caster DJ, Summersgill JT, Paueksakon P, Massung RF, Shieh WJ, McLeish KR. Mixed cryoglobulinemia and secondary membranoproliferative glomerulonephritis associated with ehrlichiosis. CEN Case Rep. 2014;3(2):178-82. Epub 2014/11/01. doi: 10.1007/s13730-014-0113-6. PubMed PMID: 28509195; PubMed Central PMCID: PMCPMC5413663.

169. Hidrón Botero A, Muñoz Ramirez F, Vega Miranda J. Primer caso de ehrlichiosis monocítica humana reportado en Colombia. Infectio. 2014;18(4):162-6. doi: 10.1016/j.infect.2014.04.001.

170. Otrock ZK, Eby CS. Ehrlichia-induced hemophagocytic lymphohistiocytosis: A case series. Blood. 2014;124(21).

171. Sachdev SH, Joshi V, Cox ER, Amoroso A, Palekar S. Severe life-threatening Ehrlichia chaffeensis infections transmitted through solid organ transplantation. Transpl Infect Dis. 2014;16(1):119-24. Epub 2013/12/18. doi: 10.1111/tid.12172. PubMed PMID: 24330198.

172. Scott MC, Holtzman NG, Afshar M, Hines SE. Ehrlichiosis-induced hemophagocytic lymphohistiocytosis presenting as septic shock-an immune response gone haywire and its lessons for the ICU setting. American Journal of Respiratory and Critical Care Medicine. 2014;189.

173. Stollings JL, Chadha SN, Paul AM, Shaver CM, Hagaman D. Doxycycline desensitization for a suspected case of ehrlichiosis. J Allergy Clin Immunol Pract. 2014;2(1):103-4. Epub 2014/02/26. doi: 10.1016/j.jaip.2013.08.002. PubMed PMID: 24565779.

174. Broussard JR, Johansen K. Autoimmune primary adrenal insufficiency associated with aseptic meningitis from tick-borne infection. Endocrine Reviews. 2015;36.

175. Burke A, Graves SR, Anstey NM. Fever and rash from Timor: where have you been and when? Medical Journal of Australia. 2015;203(8):338-. doi: 10.5694/mja15.00936. PubMed PMID: 110420046. Language: English. Entry Date: 20160228. Revision Date: 20191111. Publication Type: journal article.

176. Hilal T, Snapp WK. The perils of country life: human monocytic ehrlichiosis. Am J Med. 2015;128(8):831-3. Epub 2015/04/29. doi: 10.1016/j.amjmed.2015.04.003. PubMed PMID: 25912197.

177. Nathan K, Agarwal A, Park D, Agrawal A. Hepatitis and lipase elevation in human monocytic ehrlichiosis in a critically ill patient. Chest. 2015;148(4). doi: 10.1378/chest.2281660.

178. Statler VA, Marshall GS. Hemophagocytic lymphohistiocytosis induced by monocytic ehrlichiosis. J Pediatr. 2015;166(2):499-99.e1. Epub 2014/12/03. doi: 10.1016/j.jpeds.2014.09.053. PubMed PMID: 25449215.

179. Vijayan V, Thambundit A, Sukumaran S. Hemophagocytic lymphohistiocytosis secondary to ehrlichiosis in a child. Clin Pediatr (Phila). 2015;54(1):84-6. Epub 2014/04/03. doi: 10.1177/0009922814529183. PubMed PMID: 24691079.

180. Williams F, Cheng A, Fortenberry J, Salinas S, Preissig C, Kamat P. Extracorporeal membrane oxygenation for hemophagocytic lymphohistiocytosis secondary to ehrlichiosis. Critical Care Medicine. 2015;43(12):306. doi: 10.1097/01.ccm.0000475048.82994.7b.

181. Abusaada K, Ajmal S, Hughes L. Successful Treatment of Human Monocytic Ehrlichiosis with Rifampin. Cureus. 2016;8(1):e444. Epub 2016/02/27. doi: 10.7759/cureus.444. PubMed PMID: 26918212; PubMed Central PMCID: PMCPMC4744069.

182. Chikeka I, Matute AJ, Dumler JS, Woods CW, Mayorga O, Reller ME. Use of Peptide-Based Enzyme-Linked Immunosorbent Assay followed by Immunofluorescence Assay To Document Ehrlichia chaffeensis as a Cause of Febrile Illness in Nicaragua. J Clin Microbiol. 2016;54(6):1581-5. Epub 2016/04/08. doi: 10.1128/jcm.03331-15. PubMed PMID: 27053675; PubMed Central PMCID: PMCPMC4879277.

183. Dredla B, Freeman WD. Ehrlichia Meningitis Mimicking Aneurysmal Subarachnoid Hemorrhage: A Case Study for Medical Decision-Making Heuristics. Neurohospitalist. 2016;6(2):76-9. Epub 2016/04/08. doi: 10.1177/1941874415596743. PubMed PMID: 27053985; PubMed Central PMCID: PMCPMC4802771.

184. Geier C, Davis J, Siegel M. Severe human monocytic ehrlichiosis presenting with altered mental status and seizures. BMJ Case Rep. 2016;2016. Epub 2016/10/21. doi: 10.1136/bcr-2016-215967. PubMed PMID: 27758794; PubMed Central PMCID: PMCPMC5073573.

185. Halac E. [Ehrlichia infection in a child: clinical findings and review of the literature]. Arch Argent Pediatr. 2016;114(3):e199-200. Epub 2016/05/11. doi: 10.5546/aap.2016.e199. PubMed PMID: 27164358.

186. Harris RM, Couturier BA, Sample SC, Coulter KS, Casey KK, Schlaberg R. Expanded Geographic Distribution and Clinical Characteristics of Ehrlichia ewingii Infections, United States. Emerg Infect Dis. 2016;22(5):862-5. Epub 2016/04/19. doi: 10.3201/eid2205.152009. PubMed PMID: 27089171; PubMed Central PMCID: PMCPMC4861533.

187. Kaplan RM, Swat SA, Singer BD. Human monocytic ehrlichiosis complicated by hemophagocytic lymphohistiocytosis and multi-organ dysfunction syndrome. Diagn Microbiol Infect Dis. 2016;86(3):327-8. Epub 2016/08/31. doi: 10.1016/j.diagmicrobio.2016.08.007. PubMed PMID: 27575459; PubMed Central PMCID: PMCPMC5061620.

188. Nguyen LP. Hemophagocytic lymphohistiocytosis caused by infection by ehrlichia chaffeensis: A case report. Journal of Investigative Medicine. 2016;64(4):974. doi: 10.1136/jim-2016-000120.131.

189. Raja H, Mercer RC, Bakri SJ. PRESUMED POSTERIOR UVEITIS RELATED TO EHRLICHIA EXPOSURE. Retin Cases Brief Rep. 2016;10(2):134-6. Epub 2015/09/10. doi: 10.1097/icb.0000000000000192. PubMed PMID: 26352324.

190. Sedarati K, Taghadosi F, Aksenov I, Ramage J. An unusual cause of atrial fibrillation with rapid ventricular response. Chest. 2016;150(4):404A. doi: 10.1016/j.chest.2016.08.417.

191. Sosa-Gutierrez CG, Solorzano-Santos F, Walker DH, Torres J, Serrano CA, Gordillo-Perez G. Fatal Monocytic Ehrlichiosis in Woman, Mexico, 2013. Emerg Infect Dis. 2016;22(5):871-4. Epub 2016/04/19. doi: 10.3201/eid2205.151217. PubMed PMID: 27088220; PubMed Central PMCID: PMCPMC4861511.

192. Ta H, Reid B, Bhatia D, Levorson R. Ehrlichiosis Induced Aseptic Meningitis and Second-Degree Heart Block in an Adolescent Male. Pediatr Infect Dis J. 2016;35(7):807-9. Epub 2016/04/21. doi: 10.1097/inf.0000000000001161. PubMed PMID: 27097347.

193. Thipmontree W, Chao CC, Suputtamongkol Y, Zhang Z, Belinskaya T, Ching WM. Human ehrlichiosis and anaplasmosis in northeastern Thailand. American Journal of Tropical Medicine and Hygiene. 2016;95(5):62. doi: 10.4269/ajtmh.abstract2016.

194. Bhalla V, Rodgers B, Lin J. Sudden sensorineural hearing loss in human monocytic ehrlichiosis. Ear Nose Throat J. 2017;96(8):328-42. Epub 2017/08/29. doi: 10.1177/014556131709600820. PubMed PMID: 28846788.

195. García-Baena C, Cárdenas MF, Ramón JF. Cerebral haemorrhage as a clinical manifestation of human ehrlichiosis. BMJ Case Reports. 2017;2017. doi: 10.1136/bcr-2016-219054.

196. Heath JK, Courtright KR. Suspect the unexpected: A 45-year-old man with fevers, rash, and multiorgan failure. Annals of the American Thoracic Society. 2017;14(9):1469-74. doi: 10.1513/AnnalsATS.201703-235CC.

197. Naqash AR, Yogarajah M, Vallangeon BD, Hafiz M, Patel D, Kolychev E, et al. Hemophagocytic lymphohistiocytosis (HLH) secondary to Ehrlichia chaffeensis with bone marrow involvement. Ann Hematol. 2017;96(10):1755-8. Epub 2017/08/02. doi: 10.1007/s00277-017-3085-8. PubMed PMID: 28762082.

198. Regunath H, Rojas-Moreno C, Olano JP, Hammer RD, Salzer W. Early diagnosis of Ehrlichia ewingii infection in a lung transplant recipient by peripheral blood smear. Transpl Infect Dis. 2017;19(2). Epub 2016/12/31. doi: 10.1111/tid.12652. PubMed PMID: 28036138.

199. Zhu H, Arukala V, Sheikh S, Uprety A, McCrary D, Silverman T, et al. Human monocytic ehrlichiosis presenting as guillain-barre syndrome. Neurology. 2017;88(16).

200. Buzzard SL, Bissell BD, Bastin MLT. Ehrlichiosis presenting as severe sepsis and meningoencephalitis in an immunocompetent adult. JMM Case Reports. 2018;5(9). doi: 10.1099/jmmcr.0.005162.

201. Chen D, Cholin L, Jalil BA, El-Kersh K. Ehrlichiosis masquerading as thrombotic thrombocytopenic purpura. BMJ Case Rep. 2018;2018. Epub 2018/10/04. doi: 10.1136/bcr-2018-226665. PubMed PMID: 30279260; PubMed Central PMCID: PMCPMC6169694.

202. Dutta S, Patel C, Sutton C, Genese F, Miller P, Asad R. A ticking time bomb: A mysterious case of altered mental status. Critical Care Medicine. 2018;46:299. doi: 10.1097/01.ccm.0000528640.98141.18.

203. Kaplun O, Smith K, Khoo T, Spitzer E, Weinbaum F, Marcos LA. Key clinical and laboratory features in early diagnosis of ehrlichiosis in an endemic area of long Island, New York. Open Forum Infectious Diseases. 2018;5:S240-S1. doi: 10.1093/ofid/ofy210.672.

204. Khoury JA, Mickey W, Marrocco M, Katyal A. A Fatal Case of Hemophagocytic Lymphohistiocytosis in a Patient with Ehrlichiosis. Infectious Diseases in Clinical Practice. 2018;26(6):e64-e6. doi: 10.1097/IPC.0000000000000621.

205. Kollipara V, Sofi U. HOW HIGH CAN FERRITIN LEVEL BE? A RARE CASE OF EHRLICHIOSIS-INDUCED HLH WITH REACTIVATION OF EBV. CHEST. 2018;154:232A-A. doi: 10.1016/j.chest.2018.08.209. PubMed PMID: 132238878. Language: English. Entry Date: In Process. Revision Date: 20190909. Publication Type: Article. Supplement Title: Oct2018 Supplement. Journal Subset: Biomedical.

206. Lowther GH, Chertoff JL, Cherabuddi K, Zumberg M, Ataya A. A case of ehrlichia chaffeensis induced hemophagocytic lymphohistiocytosis. American Journal of Respiratory and Critical Care Medicine. 2018;197(MeetingAbstracts).

207. Mah A, Viola GM, Ariza Heredia E, Rezvani K, Kebriaei P, Bhatti MM, et al. Graft loss attributed to possible transfusion-transmitted ehrlichiosis following cord blood stem cell transplant. Transpl Infect Dis. 2018;20(4):e12899. Epub 2018/04/19. doi: 10.1111/tid.12899. PubMed PMID: 29668040.

208. Samannan R, McCaffree DR. Successful treatment of hemophagocytic lymphohistiocytosis secondary to ehrlichia infection. American Journal of Respiratory and Critical Care Medicine. 2018;197(MeetingAbstracts).

209. Uvodich ME, Poplin V, Male HJ. Is it a Drug or a Bug? A Case of Chemotherapy and Immune Modulators Complicating the Diagnosis of Ehrlichiosis. Kans J Med. 2018;11(3):76-7. Epub 2018/09/13. PubMed PMID: 30206467; PubMed Central PMCID: PMCPMC6122885.

210. Wilfong EM, Hewlett JC, Kerchberger VE, Bloch KC, Sevin CM. Ehrlichia-an unusual cause of hemophagocytic lymphohistiocytosis. American Journal of Respiratory and Critical Care Medicine. 2018;197(MeetingAbstracts).

211. Almaddah N, Rawal A, Nanda A, Hesterberg K, Ashraf F, Khouzam R, et al. FROM TICK BITE TO HEART FAILURE. Journal of the American College of Cardiology. 2019;73(9 Supplement 1):2367. doi: 10.1016/S0735-1097(19)32973-0.

212. Amariei D, Beer T, Armstrong F, Todd NW, Deepak J. Multi-organ failure in hemophagocytic lymphohistiocytosis secondary to ehrlichiosis: A success story. American Journal of Respiratory and Critical Care Medicine. 2019;199(9).

213. Bissell B, Buzzard S, Bastin MT. Ehrlichiosis presenting as severe sepsis and meningoencephalitis in an immunocompetent adult. Critical Care Medicine. 2019;47(1).

214. Chung P, Arain E, Gilbert E. EHRLICHIOSIS: A RARE CAUSE OF SEPSIS AND HEMOPHAGOCYTIC LYMPHOHISTIOCYTOSIS. Chest. 2019;156(4):A41. doi: 10.1016/j.chest.2019.08.142.

215. Emiroğlu M, Çelebi B. First report of human ehrlichiosis in Turkey. Turk J Pediatr. 2019;61(2):267-70. Epub 2020/01/18. doi: 10.24953/turkjped.2019.02.017. PubMed PMID: 31951339.

216. Mahapatra R, Cohen D, Viccellio AW, Sasson A, Bandovic J, Spitzer ED, et al. Acute acalculous cholecystitis as a manifestation of ehrlichiosis. Ticks Tick Borne Dis. 2019;10(5):1033-4. Epub 2019/06/04. doi: 10.1016/j.ttbdis.2019.05.006. PubMed PMID: 31155368.

217. Messana J, Davison D. An unexpected presentation of ticking off the inflammatory cascade. Critical Care Medicine. 2019;47(1).

218. Otrock ZK, Eby CS, Burnham CAD. Human ehrlichiosis at a tertiary-care academic medical center: Clinical associations and outcomes of transplant patients and patients with hemophagocytic lymphohistiocytosis. Blood Cells, Molecules, and Diseases. 2019;77:17-22. doi: 10.1016/j.bcmd.2019.03.002.

219. Peng SH, Yang SL, Ho YN, Chen HF, Shu PY. Human Case of Ehrlichia chaffeensis Infection, Taiwan. Emerg Infect Dis. 2019;25(11):2141-3. Epub 2019/10/19. doi: 10.3201/eid2511.190665. PubMed PMID: 31625863; PubMed Central PMCID: PMCPMC6810210.

220. Raru Y, Abu-Hashyeh A, Zeid F. SEPTIC SHOCK AND HEMOPHAGOCYTIC LYMPHOHISTOCYTOSIS IN AN IMMUNOCOMPROMISED HOST SECONDARY TO EHRLICHIOSIS. Chest. 2019;156(4):A1976. doi: 10.1016/j.chest.2019.08.1958.

221. Strumpf ZB, Wilding E. Don't forget doxycycline: A case of ehrlichiosis complicated by myocarditis and hemophagocytic lymphohistiocytosis. American Journal of Respiratory and Critical Care Medicine. 2019;199(9).

222. Ahmad A, Ross L. Ehrlichosis-a case of a life threatening tick born disease. Journal of the American Geriatrics Society. 2020;68(SUPPL 1):S183. doi: 10.1111/jgs.16431.

223. Alcántara-Rodríguez VE, Sánchez-Montes S, Contreras H, Colunga-Salas P, Fierro-Flores L, Avalos S, et al. Human Monocytic Ehrlichiosis, Mexico City, Mexico. Emerg Infect Dis. 2020;26(12):3016-9. Epub 2020/11/22. doi: 10.3201/eid2612.200520. PubMed PMID: 33219798; PubMed Central PMCID: PMCPMC7706933.

224. Breitschwerdt EB, Maggi RG. My Mother's Story: Tick Borne Ehrlichiosis and a Life Well-Lived. Vector Borne Zoonotic Dis. 2020;20(5):319-24. Epub 2019/12/17. doi: 10.1089/vbz.2019.2570. PubMed PMID: 31841654.

225. Cabler SS, Hogan PG, Fritz SA, Bednarski JJ, Hunstad DA. Incidence and treatment of hemophagocytic lymphohistiocytosis in hospitalized children with Ehrlichia infection. Pediatr Blood Cancer. 2020;67(10):e28436. Epub 2020/07/25. doi: 10.1002/pbc.28436. PubMed PMID: 32706439.

226. Dahm CN, Yang BQ, Clark DE, Armstrong WC, Stevenson LW. Human Monocytic Ehrlichiosis Associated With Myocarditis and Hemophagocytic Lymphohistiocytosis. JACC Case Rep. 2020;2(3):420-5. Epub 2020/03/18. doi: 10.1016/j.jaccas.2019.12.042. PubMed PMID: 34317254; PubMed Central PMCID: PMCPMC8311717.

227. Hassan W, Talwar M, Balaraman V, Molnar MZ. Ehrlichiosis infection mimicking thrombotic microangiopathy syndrome early after kidney transplantation. Transpl Infect Dis. 2020;22(5):e13305. Epub 2020/05/03. doi: 10.1111/tid.13305. PubMed PMID: 32358827.

228. Kapadia S, Al-Hillan A, Alshami A, Mohamed M, Al-Bayati A, Al Azzawi M, et al. Rare case of hepatitis due to ehrlichiosis. American Journal of Gastroenterology. 2020;115(SUPPL):S1292. doi: 10.14309/01.ajg.0000711804.53662.e9.

229. Kunnumpurath A, Kamoga R. 'you make my hear flutter' an arkansonian tick's love story: An unusual cause of atrial flutter. Journal of Investigative Medicine. 2020;68(2):534-5. doi: 10.1136/jim-2020-SRM.250.

230. Kuriakose K, Pettit AC, Schmitz J, Moncayo A, Bloch KC. Assessment of Risk Factors and Outcomes of Severe Ehrlichiosis Infection. JAMA Netw Open. 2020;3(11):e2025577. Epub 2020/11/18. doi: 10.1001/jamanetworkopen.2020.25577. PubMed PMID: 33201233; PubMed Central PMCID: PMCPMC7672514

231. Masterson EM, Gupta S, Jakharia N, Peacock JE, Jr. Ehrlichiosis in a recent kidney transplant recipient: The repellent that did not repel! A case report and literature review of ehrlichiosis in solid organ transplant patients. Transpl Infect Dis. 2020;22(4):e13299. Epub 2020/04/20. doi: 10.1111/tid.13299. PubMed PMID: 32306509.

232. Morena J, Antimisiaris M, Boggs Z, Singh D, Lustberg M. Ehrlichia meningoencephalitis: A case series. Neurology. 2020;94(15).

233. Patel TP, Beck P, Chairman D, Regunath H. Ehrlichiosis Presenting as Hemophagocytic Lymphohistiocytosis in an Immunocompetent Adult. IDCases. 2020;20:e00813. Epub 2020/05/27. doi: 10.1016/j.idcr.2020.e00813. PubMed PMID: 32455115; PubMed Central PMCID: PMCPMC7235617.

234. Yen TY, Tung YJ, Wang HC, Tsai KH. Detection of Ehrlichia chaffeensis in a febrile patient in Kinmen, an offshore island of Taiwan. J Formos Med Assoc. 2020;119(8):1329-30. Epub 2019/12/14. doi: 10.1016/j.jfma.2019.11.019. PubMed PMID: 31831322.

235. Ambati SR, Sinha S, Barry S, Sanchez J. Lone star tick bites big apple: Ehrlichia chaffeensis-induced hemophagocytic lymphohistiocytosis. Critical Care Medicine. 2021;49(1 SUPPL 1):27. doi: 10.1097/01.ccm.0000726236.26151.c4.

236. Hassan A, Akhtar K, Parmar M, Hussain SYED, Brown B. EHRLICHIOSIS: A RARE CAUSE OF HEMOPHAGOCYTIC LYMPHOHISTIOCYTOSIS. Chest. 2021;160(4):A884. doi: 10.1016/j.chest.2021.07.826.

237. Higuita NIA, Yuen C. Hemophagocytic Lymphohistiocytosis Secondary to Ehrlichia Chaffeensis in Adults: A Case Series From Oklahoma. American Journal of the Medical Sciences. 2021;361(2):269-73. PubMed PMID: WOS:000628856400022.

238. Judge C, Ravichandran A, Walsh A. Secondary hemophagocytic lymphohistiocytosis due to human monocytic ehrlichiosis in an immunosuppressed renal transplant adult. Neurology. 2021;96(15 SUPPL 1).

239. Larsen AM, Martin K. Ehrlichia meningoencephalitis and severe sepsis. Journal of Investigative Medicine. 2021;69(2):587. doi: 10.1136/jim-2021-SRMC.427.

240. Mahapatra L, Wang T, Lee YS, Frater JL. Not all lymphoid aggregates in chronic lymphocytic leukemia (CLL) patients are due to CLL! Clin Case Rep. 2021;9(3):1785-6. Epub 2021/03/27. doi: 10.1002/ccr3.3701. PubMed PMID: 33768938; PubMed Central PMCID: PMCPMC7981747.

241. Malhis JR, Mahmoud A, Belote A, Ebers A. Case of ehrlichiosis induced Guillain-Barre Syndrome in a 71 year-old female. IDCases. 2021;26:e01301. Epub 2021/11/04. doi: 10.1016/j.idcr.2021.e01301. PubMed PMID: 34729357; PubMed Central PMCID: PMCPMC8546407 of this paper.

242. Mitma AA, Burgess MJ, van Rhee F. Ehrlichia-induced hemophagocytic lymphohistiocytosis after autologous stem cell transplant. Transpl Infect Dis. 2021;23(4):e13621. Epub 2021/04/21. doi: 10.1111/tid.13621. PubMed PMID: 33877729.

243. Nelson ME, Pandey R, Nguyen T. Don't forget to ask about ticks: An unusual clinical course of ehrlichiosis in a teenage boy. Pediatrics. 2021;147(3):432-3. doi: 10.1542/peds.147.3-MeetingAbstract.432.

244. Overmiller AC, Bitter CC. Rhabdomyolysis and Multisystem Organ Failure Due to Fulminant Ehrlichiosis Infection. Wilderness Environ Med. 2021;32(2):226-9. Epub 2021/04/18. doi: 10.1016/j.wem.2021.01.009. PubMed PMID: 33863612.

245. Parkinson M, Vuyyuru S, Patel J, Animalu C. Challenges of Diagnosing Severe Ehrlichiosis in Orthotopic Liver Transplant Recipients. Case Rep Transplant. 2021;2021:8285326. Epub 2021/11/30. doi: 10.1155/2021/8285326. PubMed PMID: 34840851; PubMed Central PMCID: PMCPMC8612778.

246. Radcliffe C, Tsay C, Glerum K, Liao J, Goshua G, Kerins G. Fever, pancytopenia, and elevated D-dimer in a 95-year-old woman with ehrlichiosis: a case report. BMC Geriatr. 2021;21(1):178. Epub 2021/03/14. doi: 10.1186/s12877-021-02129-6. PubMed PMID: 33711942; PubMed Central PMCID: PMCPMC7953191.

247. Zavell A, Braudis K. 26432 A case of bullous porokeratosis–like eruption in the setting of Ehrlichia infection. Journal of the American Academy of Dermatology. 2021;85(3):AB100. doi: 10.1016/j.jaad.2021.06.420.

248. Zoglman J, Kapoor A, Naydenov S. A 13 syllable clinical conundrum. American Journal of Respiratory and Critical Care Medicine. 2021;203(9). doi: 10.1164/ajrccm-conference.2021.203.1_MeetingAbstracts.A2938.

249. Abernathy H, Alejo A, Arahirwa V, Mansour O, Brown-Marusiak A, Giandomenico D, et al. "Leopards do not change their spots:" tick borne disease symptomology case report. BMC Infect Dis. 2022;22(1):699. Epub 2022/08/20. doi: 10.1186/s12879-022-07683-x. PubMed PMID: 35986240; PubMed Central PMCID: PMCPMC9390100.

250. Almajali F, Oleary C, Hallcox T, Lok J, Hermelin D, Guenette A, et al. Ehrlichiosis in a Recent Liver Transplant Recipient Leading to Multiorgan Failure. Case Rep Transplant. 2022;2022:3062836. Epub 2022/06/21. doi: 10.1155/2022/3062836. PubMed PMID: 35722444; PubMed Central PMCID: PMCPMC9205736.

251. Bolling T, Ritter AS, Gupte AA. An Unusual Case of Ehrlichiosis Manifesting With Hyponatremia, Acute Encephalopathy, and Hemophagocytic Lymphohistiocytosis. Cureus. 2022;14(7):e26943. Epub 2022/08/23. doi: 10.7759/cureus.26943. PubMed PMID: 35989779; PubMed Central PMCID: PMCPMC9378938.

252. Butterfield JH, Elliott MA. Fulminant Ehrlichia chaffeensis Infection in a Patient Discovered to Have Indolent Systemic Mastocytosis. Infectious Diseases in Clinical Practice. 2022;30(3):2391-405. doi: 10.1097/IPC.0000000000001137.

253. Hongru S, Kenji K, Shigetoshi S, Shinsuke M, Nobuhiro K, Shigehiro A, et al. Serologic Evidence of Human Exposure to Ehrlichiosis Agents in Japan. Emerging Infectious Diseases. 2022;28(11):2355-7. doi: 10.3201/eid2811.212566. PubMed PMID: 159889081. Language: English. Entry Date: 20221030. Revision Date: 20221109. Publication Type: Article. Journal Subset: Biomedical.

254. Jabr R, El Atrouni W. Ehrlichiosis mimicking acute leukemia. IDCases. 2022;28:e01480. Epub 2022/04/12. doi: 10.1016/j.idcr.2022.e01480. PubMed PMID: 35402161; PubMed Central PMCID: PMCPMC8987808.

255. Kosaraju A, Fernandes R, Marcu CB. PREDICTING THE UNPREDICTABLE: A CASE OF EHRLICHIAL MYOCARDITIS. Journal of the American College of Cardiology. 2022;79(9):3037. doi: 10.1016/S0735-1097(22)04028-1.

256. Novoselova V, Organti N, Katyal A, Sadaka F. 707: EHRLICHIOSIS? CHECK FOR HEMOPHAGOCYTIC LYMPHOHISTIOCYTOSIS. Critical Care Medicine. 2022;50:347-. doi: 10.1097/01.ccm.0000809152.66364.19. PubMed PMID: 154197412. Language: English. Entry Date: In Process. Revision Date: 20211222. Publication Type: Article. Supplement Title: 2022 Supplement. Journal Subset: Biomedical.

257. Saha A, Browning C, Dandamudi R, Barton K, Graepel K, Cullity M, et al. Donor-derived Ehrlichiosis: 2 Clusters Following Solid Organ Transplantation. Clin Infect Dis. 2022;74(5):918-23. Epub 2021/07/31. doi: 10.1093/cid/ciab667. PubMed PMID: 34329411; PubMed Central PMCID: PMCPMC8906657.

258. Singh NS, Pagano AL, Hays AJ, Kats A, Dahl SM, Warady BA, et al. Ehrlichia-induced hemophagocytic lymphohistiocytosis in a pediatric kidney transplant recipient. Pediatr Transplant. 2022;26(1):e14134. Epub 2021/10/02. doi: 10.1111/petr.14134. PubMed PMID: 34595809.

259. Amjad MA, Hamid Z, Patel Y, Natarajan S, Ochieng P. 382: EHRLICHIOSIS-INDUCED MYOCARDITIS. Critical Care Medicine. 2023;51:177-. doi: 10.1097/01.ccm.0000907256.06944.3b. PubMed PMID: 160832848. Language: English. Entry Date: In Process. Revision Date: 20221220. Publication Type: Article. Supplement Title: 2023 Supplement. Journal Subset: Biomedical.

260. Begley N, Kolagatla S, Smith F, Piercy J. Ehrlichiosis: a rare case of acute hepatic and renal failure. American Journal of the Medical Sciences. 2023;365:S146-S7. doi: 10.1016/S0002-9629(23)00281-1.

261. Hammoud K, Fulmer R, Hamner M, El Atrouni W. Ehrlichiosis-Associated Hemophagocytic Lymphohistiocytosis: A Case Series and Review of the Literature. Case Rep Hematol. 2023;2023:5521274. Epub 2023/02/28. doi: 10.1155/2023/5521274. PubMed PMID: 36846547; PubMed Central PMCID: PMCPMC9946753.

262. Islam MI, Schecter S. 479: MULTIORGAN FAILURE AND EHRLICHIOSIS: TIMELY TREATMENT IS THE KEY TO SUCCESS. Critical Care Medicine. 2023;51:227-. doi: 10.1097/01.ccm.0000907644.74186.b8. PubMed PMID: 160832945. Language: English. Entry Date: In Process. Revision Date: 20221220. Publication Type: Article. Supplement Title: 2023 Supplement. Journal Subset: Biomedical.

263. Lu M, Qin XC, Jiang YZ, Guo Q, Jin XJ, Teng ZQ, et al. Emergence of ehrlichiosis by a new tick-borne Ehrlichia species in China. International Journal of Infectious Diseases. 2023;131:32-9. doi: 10.1016/j.ijid.2023.03.038. PubMed PMID: WOS:000981486100001.

264. Mikheev AT, Adawi M, Mirza A, Bechtel A. Prolonged fever and inflammation in a young child in the midst of the pandemic - it's not always MIS-C. American Journal of the Medical Sciences. 2023;365:S390. doi: 10.1016/S0002-9629(23)00717-6.

**Excluded publications**

1. Smith T, Johnson HP. ON A COCCIDIUM (KLOSSIELLA MURIS, GEN. ET SPEC. NOV.) PARASITIC IN THE RENAL EPITHELIUM OF THE MOUSE. J Exp Med. 1902;6(3):303-16. Epub 1902/03/01. doi: 10.1084/jem.6.3.303. PubMed PMID: 19866974; PubMed Central PMCID: PMCPMC2124504.

2. Smith T. Further observations on the Transmission of Sarcocystis muris by Feeding. J Med Res. 1905;13(4):429-30. Epub 1905/05/01. PubMed PMID: 19971680; PubMed Central PMCID: PMCPMC2099954.

3. Wenyon CM. Spirochaetosis of Mice due to Spirochaeta muris n.sp. in the Blood. J Hyg (Lond). 1906;6(5):580-5. Epub 1906/10/01. doi: 10.1017/s0022172400003181. PubMed PMID: 20474291; PubMed Central PMCID: PMCPMC2236161.

4. Darling ST. EXPERIMENTAL SARCOSPORIDIOSIS IN THE GUINEA-PIG AND ITS RELATION TO A CASE OF SARCOSPORIDIOSIS IN MAN. J Exp Med. 1910;12(1):19-28. Epub 1910/01/01. doi: 10.1084/jem.12.1.19. PubMed PMID: 19867309; PubMed Central PMCID: PMCPMC2124757.

5. Tyzzer EE. An extracellular Coccidium, Cryptosporidium Muris (Gen. Et Sp. Nov.), of the gastric Glands of the Common Mouse. J Med Res. 1910;23(3):487-510.3. Epub 1910/11/01. PubMed PMID: 19971982; PubMed Central PMCID: PMCPMC2098948.

6. POEMS OF HUMAN PROGRESS, and other Pieces: Including One Hundred and Fifteen Sonnets. Education. 1915;36(2):131-. PubMed PMID: WOS:000205274300029.

7. Blake FG. THE ETIOLOGY OF RAT-BITE FEVER. J Exp Med. 1916;23(1):39-60. Epub 1916/01/01. doi: 10.1084/jem.23.1.39. PubMed PMID: 19867970; PubMed Central PMCID: PMCPMC2125348.

8. Pearce L. KLOSSIELLA INFECTION OF THE GUINEA PIG. J Exp Med. 1916;23(4):431-42. Epub 1916/04/01. doi: 10.1084/jem.23.4.431. PubMed PMID: 19867997; PubMed Central PMCID: PMCPMC2125436.

9. Futaki K, Takaki I, Taniguchi T, Osumi S. SPIROCHAETA MORSUS MURIS, N.SP., THE CAUSE OF RAT-BITE FEVER : SECOND PAPER. J Exp Med. 1917;25(1):33-44. Epub 1917/01/01. doi: 10.1084/jem.25.1.33. PubMed PMID: 19868077; PubMed Central PMCID: PMCPMC2125538.

10. Cowdry EV. STUDIES ON THE ETIOLOGY OF HEARTWATER : II. RICKETTSIA RUMINANTIUM (N. SP.) IN THE TISSUES OF TICKS TRANSMITTING THE DISEASE. J Exp Med. 1925;42(2):253-74. Epub 1925/07/31. doi: 10.1084/jem.42.2.253. PubMed PMID: 19869050; PubMed Central PMCID: PMCPMC2130996.

11. Cowdry EV. STUDIES ON THE ETIOLOGY OF HEARTWATER : I. OBSERVATION OF A RICKETTSIA, RICKETTSIA RUMINANTIUM (N. SP.), IN THE TISSUES OF INFECTED ANIMALS. J Exp Med. 1925;42(2):231-52. Epub 1925/07/31. doi: 10.1084/jem.42.2.231. PubMed PMID: 19869049; PubMed Central PMCID: PMCPMC2130998.

12. Cowdry EV. STUDIES ON THE ETIOLOGY OF HEARTWATER : III. THE MULTIPLICATION OF RICKETTSIA RUMINANTIUM WITHIN THE ENDOTHELIAL CELLS OF INFECTED ANIMALS AND THEIR DISCHARGE INTO THE CIRCULATION. J Exp Med. 1926;44(6):803-14. Epub 1926/11/30. doi: 10.1084/jem.44.6.803. PubMed PMID: 19869225; PubMed Central PMCID: PMCPMC2131225.

13. Ford WW, Eliot CP. THE TRANSFER OF RAT ANEMIA TO NORMAL ANIMALS. J Exp Med. 1928;48(4):475-92. Epub 1928/09/30. doi: 10.1084/jem.48.4.475. PubMed PMID: 19869499; PubMed Central PMCID: PMCPMC2131488.

14. Noguchi H. ETIOLOGY OF OROYA FEVER : XI. COMPARISON OF BARTONELLA BACILLIFORMIS AND BARTONELLA MURIS. CULTIVATION OF BACTERIUM MURIUM, N. SP. J Exp Med. 1928;47(2):235-43. Epub 1928/01/31. doi: 10.1084/jem.47.2.235. PubMed PMID: 19869409; PubMed Central PMCID: PMCPMC2131353.

15. Marmorston-Gottesman J, Perla D. STUDIES ON BARTONELLA MURIS ANEMIA OF ALBINO RATS : I. TRYPANOSOMA LEWISI INFECTION IN NORMAL ALBINO RATS ASSOCIATED WITH BARTONELLA MURIS ANEMIA II. LATENT INFECTION IN ADULT NORMAL RATS. J Exp Med. 1930;52(1):121-9. Epub 1930/06/30. doi: 10.1084/jem.52.1.121. PubMed PMID: 19869745; PubMed Central PMCID: PMCPMC2131866.

16. Perla D, Marmorston-Gottesman J. STUDIES ON BARTONELLA MURIS ANEMIA OF ALBINO RATS : III. THE PROTECTIVE EFFECT OF AUTOPLASTIC SPLENIC TRANSPLANTS ON THE BARTONELLA MURIS ANEMIA OF SPLENECTOMIZED RATS. J Exp Med. 1930;52(1):131-43. Epub 1930/06/30. doi: 10.1084/jem.52.1.131. PubMed PMID: 19869746; PubMed Central PMCID: PMCPMC2131867.

17. Perla D, Marmorston-Gottesman J. FURTHER STUDIES ON T. LEWISI INFECTION IN ALBINO RATS : I. THE EFFECT OF SPLENECTOMY ON T. LEWISI INFECTION IN ALBINO RATS AND THE PROTECTIVE ACTION OF SPLENIC AUTOTRANSPLANTS II. THE EFFECT OF THYMECTOMY AND BILATERAL GONADECTOMY ON T. LEWISI INFECTION IN ALBINO RATS. J Exp Med. 1930;52(4):601-16. Epub 1930/09/30. doi: 10.1084/jem.52.4.601. PubMed PMID: 19869791; PubMed Central PMCID: PMCPMC2131892.

18. Bayon HP. DISCUSSION ON THE PATHOLOGY AND TREATMENT OF HÆMOPATHIES, INCLUDING LEUKÆMIAS, OF MAN AND ANIMALS: The Hæmopathies of Laboratory Animals. Proc R Soc Med. 1931;24(8):1141-56. Epub 1931/06/01. PubMed PMID: 20912613.

19. Mackay EM, Polland WS. COMPENSATORY HYPERTROPHY OF THE SPLEEN. J Exp Med. 1931;53(3):317-24. Epub 1931/02/28. doi: 10.1084/jem.53.3.317. PubMed PMID: 19869845; PubMed Central PMCID: PMCPMC2131971.

20. Marmorston-Gottesman J, Perla D. STUDIES ON BARTONELLA MURIS ANEMIA : V. COMPENSATORY PHENOMENA FOLLOWING SPLENECTOMY IN THE ADULT ALBINO RAT. J Exp Med. 1931;53(6):877-83. Epub 1931/05/31. doi: 10.1084/jem.53.6.877. PubMed PMID: 19869890; PubMed Central PMCID: PMCPMC2132037.

21. Nelson JB. The Biological Characters of B. actinoides Variety Muris. J Bacteriol. 1931;21(3):183-95. Epub 1931/03/01. doi: 10.1128/jb.21.3.183-195.1931. PubMed PMID: 16559478; PubMed Central PMCID: PMCPMC533219.

22. Perla D, Marmorston-Gottesman J. STUDIES ON BARTONELLA MURIS ANEMIA : IV. PATHOLOGICAL CHANGES DURING THE ACUTE ANEMIA. J Exp Med. 1931;53(6):869-76. Epub 1931/05/31. doi: 10.1084/jem.53.6.869. PubMed PMID: 19869889; PubMed Central PMCID: PMCPMC2132033.

23. Marmorston-Gottesman J, Perla D. THE ETIOLOGY OF BARTONELLA MURIS ANEMIA OF THE ALBINO RAT : THE ISOLATION OF BARTONELLA MURIS. J Exp Med. 1932;56(5):763-75. Epub 1932/10/31. doi: 10.1084/jem.56.5.763. PubMed PMID: 19870100; PubMed Central PMCID: PMCPMC2132187.

24. Marmorston-Gottesman J, Perla D. THE EFFECT OF BILATERAL SUPRARENALECTOMY IN ADULT ALBINO RATS ON THE NATURAL AND ACQUIRED RESISTANCE TO BARTONELLA MURIS ANEMIA. J Exp Med. 1932;55(1):109-19. Epub 1932/01/01. doi: 10.1084/jem.55.1.109. PubMed PMID: 19869970; PubMed Central PMCID: PMCPMC2132077.

25. Perla D, Marmorston-Gottesman J. STUDIES ON BARTONELLA MURIS ANEMIA : VI. A LIPOID EXTRACT OF THE SPLEEN THAT PREVENTS BARTONELLA MURIS ANEMIA IN SPLENECTOMIZED ALBINO RATS. J Exp Med. 1932;56(6):777-82. Epub 1932/11/30. doi: 10.1084/jem.56.6.777. PubMed PMID: 19870101; PubMed Central PMCID: PMCPMC2132206.

26. Perla D, Marmorston-Gottesman J. STUDIES ON BARTONELLA MURIS ANEMIA : VII. THE PROTECTIVE ACTION OF COPPER AND IRON AGAINST BARTONELLA MURIS ANEMIA. J Exp Med. 1932;56(6):783-92. Epub 1932/11/30. doi: 10.1084/jem.56.6.783. PubMed PMID: 19870102; PubMed Central PMCID: PMCPMC2132200.

27. Sandberg M, Perla D, Marmorston-Gottesman J. STUDIES ON BARTONELLA MURIS ANEMIA : VIII. CHANGES IN THE CHEMICAL CONSTITUENTS OF THE BLOOD DURING THE ANEMIA. J Exp Med. 1933;57(1):81-93. Epub 1933/01/01. doi: 10.1084/jem.57.1.81. PubMed PMID: 19870123; PubMed Central PMCID: PMCPMC2132214.

28. Kikuth W. The Bartonella and Related Parasites in Man and Animals (Oroya Fever and Verruga peruviana): (Section of Tropical Diseases and Parasitology). Proc R Soc Med. 1934;27(9):1241-50. Epub 1934/07/01. PubMed PMID: 19989875; PubMed Central PMCID: PMCPMC2205072.

29. Sandberg M, Perla D. THE METABOLISM OF COPPER AND IRON IN SPLENECTOMIZED RATS FREE FROM BARTONELLA MURIS INFECTION. J Exp Med. 1934;60(3):395-402. Epub 1934/08/31. doi: 10.1084/jem.60.3.395. PubMed PMID: 19870310; PubMed Central PMCID: PMCPMC2132417.

30. Rhoads CP, Miller DK. THE ASSOCIATION OF BARTONELLA BODIES WITH INDUCED ANEMIA IN THE DOG. J Exp Med. 1935;61(1):139-48. Epub 1935/01/01. doi: 10.1084/jem.61.1.139. PubMed PMID: 19870342; PubMed Central PMCID: PMCPMC2133202.

31. Gordon AS, Kleinberg W, Charipper HA. THE RETICULO-ENDOTHELIAL SYSTEM AND HORMONE REFRACTORINESS. J Exp Med. 1939;70(4):333-46. Epub 1939/09/30. doi: 10.1084/jem.70.4.333. PubMed PMID: 19870913; PubMed Central PMCID: PMCPMC2133798.

32. Nelson JB. INFECTIOUS CATARRH OF THE ALBINO RAT : I. EXPERIMENTAL TRANSMISSION IN RELATION TO THE ROLE OF ACTINOBACILLUS MURIS. J Exp Med. 1940;72(6):645-54. Epub 1940/11/30. doi: 10.1084/jem.72.6.645. PubMed PMID: 19871049; PubMed Central PMCID: PMCPMC2135041.

33. Watt JY, Golden WR, Olason F, Mladinich G. THE RELATIONSHIP OF VITAMIN A TO RESISTENCE TO NIPPOSTRONGYLUS MURIS. Science. 1943;97(2521):381-2. Epub 1943/04/23. doi: 10.1126/science.97.2521.381. PubMed PMID: 17834101.

34. Gard S. A note on the coccidium Klossiella muris. Acta Pathol Microbiol Scand. 1945;22(4):427-34. Epub 1945/01/01. doi: 10.1111/j.1699-0463.1945.tb04078.x. PubMed PMID: 21021835.

35. Rake G, Alexander R, Hamre DM. THE RELATIONSHIP OF THE AGENT OF HEART-WATER FEVER--RICKETTSIA RUMINANTIUM. Science. 1945;102(2652):424-5. Epub 1945/10/26. doi: 10.1126/science.102.2652.424-a. PubMed PMID: 17730623.

36. Wilhelm O. [New contribution to the study of sodoku in Chile; a case of cat bite transmission of spirella morsus muris]. Bol Soc Biol Concepc. 1945;20:17-9. Epub 1945/01/01. PubMed PMID: 21011746.

37. Brumpt E. [Contribution to the study of Hepatozoon muris; use of xenodiagnosis for the identification of haemogregarin species]. Ann Parasitol Hum Comp. 1946;21(1-2):1-24. Epub 1946/01/01. PubMed PMID: 21027453.

38. Dharmendra, Mukherjee N. Action of penicillin on Mycobacterium leprae muris. Indian J Med Res. 1946;34(2):237-9. Epub 1946/10/01. PubMed PMID: 20289744.

39. Donaldson AW, Otto GF. Effects of protein-deficient diets on immunity to a nematode (Nippostrongylus muris) infection. Am J Hyg. 1946;44(3):384-400. Epub 1946/11/01. doi: 10.1093/oxfordjournals.aje.a119105. PubMed PMID: 20274591.

40. Archetti I. [Penicillin action on Bartonella muris]. Rend Ist Sup Sanit. 1947;10(Pt 4):642. Epub 1947/01/01. PubMed PMID: 18919319.

41. Thjotta T, Jonsen J. Streptothrix (Actinomyces) muris ratti (Streptobacillus moniliformis) isolated from a human infection and studied as to its relation to Emmy Klieneberger's L I. Acta Pathol Microbiol Scand. 1947;24(3-4):334-51. Epub 1947/01/01. doi: 10.1111/j.1699-0463.1947.tb00602.x. PubMed PMID: 18900901.

42. Thjotta T, Jonsen J. Streptothrix (Actinomyces) muris ratti (Streptobacillus moniliformis). Acta pathologica et microbiologica Scandinavica. 1947;24(3-4):334-51.

43. Reese JD, Fowler EM. Morphology of Hemobartonella muris as revealed by darkfield microscopy. Proc Soc Exp Biol Med. 1948;68(2):276-8. Epub 1948/06/01. doi: 10.3181/00379727-68-16454. PubMed PMID: 18869317.

44. Brackett S, Bliznick A. Screening large numbers of new chemical compounds for anthelmintic activity using infections with Nippostrongylus muris in mice. The Journal of parasitology. 1949;35(1):8-18. doi: 10.2307/3273379.

45. Kodicek E, Carpenter KJ. Experimental anemias in the rat; I. Macrocytic anemia in chronic pteroylglutamic acid deficiency and after splenectomy in Bartonella muris infection. Blood. 1950;5(6):522-39.

46. Laskowski L, Pinkerton H, Greiff D. A non-pathogenic bartonella-like organism developing in embryonate eggs after injection with Haemobartonella muris. J Immunol. 1950;64(6):455-62. Epub 1950/06/01. PubMed PMID: 15428634.

47. Lindquist WD. Attempts to adapt Nippostrongylus muris to the cotton rat. The Journal of parasitology. 1950;36(1):88. doi: 10.2307/3273506.

48. Neal RA. An experimental study of Entamoeba muris (Grassi, 1879); its morphology, affinities and host-parasite relationship. Parasitology. 1950;40(3-4):343-65. Epub 1950/10/01. doi: 10.1017/s0031182000018229. PubMed PMID: 14785977.

49. Neal RA. An experimental study of Entamoeba muris (Grassi, 1879); its. Parasitology. 1950;40(3-4):343-65.

50. Groupe V, Frankel JW, Leche-Valier MP, Waksman SA. Antiviral properties of ehrlichin, an antibiotic produced by Streptomyces lavendulae. J Immunol. 1951;67(6):471-82. Epub 1951/12/01. PubMed PMID: 14908070.

51. Rekers PE. The effect of x-irradiation on rats with and without Bartonella muris. The Journal of infectious diseases. 1951;88(3):224-9.

52. Simitch T, Petrovitch Z. Culture of Entamoeba muris of the mouse at the temperature of 22-23. Annales de parasitologie humaine et comparée. 1951;26(5-6):389-93. doi: 10.1051/parasite/1951265389.

53. Stokes JF, Gray IR, Stokes EJ. Actinomyces muris endocarditis treated. with chloramphenicol. Br Heart J. 1951;13(2):247-51. Epub 1951/04/01. doi: 10.1136/hrt.13.2.247. PubMed PMID: 14821208; PubMed Central PMCID: PMCPMC479414.

54. Heymann G. Systematic and serological diagnosis of the bacillary dysenteria with special reference to Shigella-sera of the Paul Ehrlich institution. Ärztliche Wochenschrift. 1952;7(30):698-700.

55. Ivanova MG, Gol'Denberg, II, Lukashev, II, Karut TA, Kandyba SG, Mikheilichenko PM, et al. [Studies on biological properties of Mycobacterium tuberculosis muris]. Probl Tuberk. 1952;3:22-8. Epub 1952/05/01. PubMed PMID: 14948975.

56. Ivanova MG, Gol'Denberg II, Lukashev II, Karut TA, Kandyba SG, Mikheilichenko PM, et al. Studies on biological properties of Mycobacterium tuberculosis muris. Problemy tuberkuleza. 1952;3:22-8.

57. Levaditi C. [Ehrlichin: non-bacteriostatic and non-microbicide antibiotic but clearly virulicide]. Presse Med (1893). 1952;60(35):761-2. Epub 1952/05/21. PubMed PMID: 14957813.

58. Okabe K, Shibue H. A new second intermediate host, Neocaridina denticulata, for Plagiorchis muris (Tanabe): Plagiorchidae. Jpn J Med Sci Biol. 1952;5(5):257-8. Epub 1952/10/01. doi: 10.7883/yoken1952.5.257. PubMed PMID: 13034358.

59. Wagener K, Mitscherlich E. Internationale tuberkelbakterienstämme. Pathobiology. 1952;15(6):701-14. doi: 10.1159/000160137.

60. Wigand R, Peters D. [New investigations on Bartonella muris Mayer. II..]. Z Tropenmed Parasitol. 1952;3(4):437-52. Epub 1952/05/01. PubMed PMID: 14959268.

61. Wigand R, Peters D. Blood parasites of the white mouse. II. Bartonella muris musculi Schilling. Zeitschrift für Tropenmedizin und Parasitologie. 1952;4(1):1-10.

62. Yamagishi Y. On the encystation of Entamoeba muris in the host. The Kitasato archives of experimental medicine. 1952;25(2):133-9.

63. De Souza-Araujo HC. Experimental studies on the vole bacillus (Mycobacterium tuberculosis var. muris) at the Oswaldo Cruz Institute. Revista brasileira de medicina. 1953;10(1):1-6.

64. Maldonado JF, Asenjo CF. The role of pteroylglutamic acid and vitamin B12 on the development of Nippostrongylus muris in the rat. Exp Parasitol. 1953;2(4):374-9. Epub 1953/10/01. doi: 10.1016/0014-4894(53)90023-x. PubMed PMID: 13107691.

65. Sula L, Zavadilova Z, Medulanova L, Pokorny J. [New vaccine against tuberculosis. II. Characteristics of Mycobacterium muris Wells OV 166 and preparation of vaccine]. Med Dosw Mikrobiol. 1953;5(1):23-37. Epub 1953/01/01. PubMed PMID: 13071640.

66. Waterson AP, Wedgwood J. Ratbite fever; report of a case due to actinomyces muris. Lancet. 1953;1(6758):472-3. Epub 1953/03/07. doi: 10.1016/s0140-6736(53)91644-4. PubMed PMID: 13036034.

67. Wells AQ. Mycobacterium tuberculosis var. muris. J Gen Microbiol. 1953;9(1):149. Epub 1953/08/01. doi: 10.1099/00221287-9-1-149. PubMed PMID: 13084887.

68. Ansari AR. A note on the morphology and method of division in Giardia muris (Grassi). Pak J Health. 1954;4(2):55-65. Epub 1954/07/01. PubMed PMID: 14356733.

69. Ansari MA. A note on miscellaneous observations on Giardia muris (Grassi, 1879). Pak J Health. 1954;3(4):207-26. Epub 1954/01/01. PubMed PMID: 13154952.

70. Cronin MT. The attenuation of Mycobacterium tuberculosis var. muris (the vole acid-fast bacillus). Journal of comparative pathology. 1954;64(3):234-42.

71. Nicewicz W. [Comparative studies on BCG strain, on Mycobacterium muris, and Lublin strain with special reference to their immunizing properties]. Gruzlica (1926). 1954;22(1):1-10. Epub 1954/01/01. PubMed PMID: 13162246.

72. Wigand R, Peters D. [Attempts of reducing Haemobartonella muris and Eperythrozoon coccoides]. Z Tropenmed Parasitol. 1954;5(4):482-92. Epub 1954/11/01. PubMed PMID: 14360361.

73. De Souza-Araujo HC. [Morphology of Mycobacterium leprae hominis and M. leprae muris; study based on electron microscopy and phase contrast]. Mem Inst Oswaldo Cruz. 1955;53(2-4):389-96. Epub 1955/06/01. PubMed PMID: 13369129.

74. Reusse U. Formation of cysts and life cycle in trichomonads with special reference to Trichomonas foetus and Trichomonas muris. Zeitschrift für Tropenmedizin und Parasitologie. 1955;6(3):348-61.

75. Barber C, Dimboviceanu A, Dumitrescu M, Ionescu-Mihaiesti C, Radulescu E, Soru E, et al. Studies of murine tuberculosis bacillus (Mycobacterium muris; vole bacillus Wells). Buletin ştiinţific Secţiunea de ştiinţe medicale Academia Republicii Populare Române. 1956;8(1):199-218.

76. Nozaki Y. Survival of giardia muris in artificial media. Japanese Journal of National Medical Services. 1956;10(5):401-6. doi: 10.11261/iryo1946.10.401.

77. Wigand R. [New studies on Haemobartonella muris Mayer. III]. Z Tropenmed Parasitol. 1956;7(3):316-21. Epub 1956/09/01. PubMed PMID: 13393279.

78. Wigand R. [Serological reactions with Haemobartonella muris and Eperythrozoon coccoides]. Z Tropenmed Parasitol. 1956;7(3):322-40. Epub 1956/09/01. PubMed PMID: 13393280.

79. Wigand R. A complement-fixation reaction with Haemobartonella muris and Eperythrozoon coccoides. Nature. 1956;178(4545):1288-9. Epub 1956/12/08. doi: 10.1038/1781288a0. PubMed PMID: 13387692.

80. Bool PH, Sutmoller P. Ehrlichia canis infections in dogs on Aruba (Netherlands Antilles). Journal of the American Veterinary Medical Association. 1957;130(9):418-20.

81. Faulkner RR, Habermann RT. Cellular factors in the acquired immunity to Bartonella muris. J Infect Dis. 1957;101(1):62-8. Epub 1957/07/01. doi: 10.1093/infdis/101.1.62. PubMed PMID: 13463384.

82. Galzerano G, Sorrentini R. [Anti-tuberculosis vaccination with Mycobacterium muris]. Arch Tisiol Mal Appar Respir. 1957;12(4):Varia, 105-14. Epub 1957/04/01. PubMed PMID: 13445455.

83. Gutierrez J, Hungate RE. Interrelationship between certain bacteria and the rumen ciliate Dasytricha ruminantium. Science. 1957;126(3272):511.

84. King EJ, Yoganathan M, Harrison CV, Mitchison DA. Experimental infective pneumoconiosis. V. Massive fibrosis of the lungs produced by coal-mine dust and Mycobacterium tuberculosis var. muris (Vole bacillus). AMA Arch Ind Health. 1957;16(5):380-92. Epub 1957/11/01. PubMed PMID: 13468804.

85. Otto H. Findings in mouse kidney in coccidiosis (Klossiella muris). Frankfurter Zeitschrift für Pathologie. 1957;68(1):41-8.

86. Angelov S, Kuiumdzhiev I, Guluboy S, Nikolov P. [Investigations on morphology, biology and immunobiology of Mycobacterium tuberculosis muris and Graub's Mycobacterium tuberculosis bovis]. Izv Mikrobiol Inst (Sofiia). 1958;9:9-21. Epub 1958/01/01. PubMed PMID: 13549088.

87. Smith PH, Hungate RE. Isolation and characterization of Methanobacterium ruminantium n. sp. J Bacteriol. 1958;75(6):713-8. Epub 1958/06/01. doi: 10.1128/jb.75.6.713-718.1958. PubMed PMID: 13549377; PubMed Central PMCID: PMCPMC290140.

88. Sula L. Five years' experience with a vaccine prepared from Mycobacterium tuberculosis var. muris. Tubercle. 1958;39(1):10-7. Epub 1958/02/01. doi: 10.1016/s0041-3879(58)80004-5. PubMed PMID: 13530215.

89. Crystal MM. Extrinsic incubation period of Haemobartonella muris in the spined rat louse, Polyplax spinulosa. Journal of bacteriology. 1959;77(4):511.

90. Gallagher CH, Symons LE. Biochemical studies on Nip-postrongylus muris infestation. Aust J Exp Biol Med Sci. 1959;37:421-32. Epub 1959/08/01. doi: 10.1038/icb.1959.43. PubMed PMID: 13826074.

91. Jackson GJ. Fluorescent antibody studies of Nippostrongylus muris infections. J Infect Dis. 1960;106:20-36. Epub 1960/01/01. doi: 10.1093/infdis/106.1.20. PubMed PMID: 14406289.

92. Byers PD, King EJ. Experimental infective pneumoconiosis with Mycobacterium tuberculosis (var. muris) and haematite by inhalation and by injection. J Pathol Bacteriol. 1961;81:123-34. Epub 1961/01/01. doi: 10.1002/path.1700810115. PubMed PMID: 13689564.

93. Scheneider CC. [Infection experiments with Lamblia muris. I. Experimental studies with the course of infection]. Z Tropenmed Parasitol. 1961;12:276-300. Epub 1961/10/01. PubMed PMID: 14042342.

94. Schneider CC. [Infection experiments with Lamblia muris. II. The effect of nutrition and other factors on the course of infection]. Z Tropenmed Parasitol. 1961;12:368-85. Epub 1961/12/01. PubMed PMID: 13908906.

95. Stahl W. Syphacia muris, the Rat Pinworm. Science. 1961;133(3452):576-7. Epub 1961/02/24. doi: 10.1126/science.133.3452.576-a. PubMed PMID: 17777087.

96. Osada M. Electron microscopic studies on protozoa. II. Studies on Trichomonas muris. The Keio journal of medicine. 1962;11:227-52.

97. Shakhnazarova I. Morphological study of the intestinal wall of mice infected with Lamblia muris. Meditsinskaia parazitologiia i parazitarnye bolezni. 1962;31:694-7.

98. Bailey RW, Howard BH. The biochemistry of rumen protozoa. 6. The maltases of Dasytricha ruminantium, Epidinium ecaudatum (Crawley) and Entodinium caudatum. The Biochemical journal. 1963;86:446-52. doi: 10.1042/bj0860446.

99. Hobson PN, Mann SO, Smith W. Growth factors for Selenomonas ruminantium. Nature. 1963;198:213. Epub 1963/04/13. doi: 10.1038/198213a0. PubMed PMID: 13954887.

100. Jensen EA, Hammond DM. A Morphological Study of Trichomonads and Related Flagellates from the Bovine Digestive Tract. The Journal of Protozoology. 1964;11(3):386-94. doi: 10.1111/j.1550-7408.1964.tb01768.x.

101. Minamishima Y, Mori R. PERSISTENT INFECTION OF FL CELLS BY RICKETTSIA SENNETSU (S. TODAI). J Bacteriol. 1964;88(4):1195-6. Epub 1964/10/01. doi: 10.1128/jb.88.4.1195-1196.1964. PubMed PMID: 14222807; PubMed Central PMCID: PMCPMC314872.

102. Seo BS, Rim HJ, Lee CW. Studies on the parasitic helmiths of Korea: I. Trematodes of rodents. Kisaengchunghak Chapchi. 1964;2(1):20-6. Epub 1964/06/01. doi: 10.3347/kjp.1964.2.1.20. PubMed PMID: 12913605.

103. Yang YH, Grice HC. Klossiella Muris Parasitism in Laboratory Mice. Can J Comp Med Vet Sci. 1964;28(3):63-6. Epub 1964/03/01. PubMed PMID: 17649494; PubMed Central PMCID: PMCPMC1494234.

104. Anderson DR, Hopps HE, Barile MF, Bernheim BC. Comparison of the ultrastructure of several rickettsiae, ornithosis virus, and Mycoplasma in tissue culture. J Bacteriol. 1965;90(5):1387-404. Epub 1965/11/01. doi: 10.1128/jb.90.5.1387-1404.1965. PubMed PMID: 4954556; PubMed Central PMCID: PMCPMC315827.

105. Ohtaki S, Shishido A. STUDIES ON INFECTIOUS MONONUCLEOSIS INDUCED IN THE MONKEY BY EXPERIMENTAL INFECTION WITH RICKETTSIA SENNETSU. II. PATHOLOGICAL FINDINGS. Jpn J Med Sci Biol. 1965;18:85-100. Epub 1965/04/01. doi: 10.7883/yoken1952.18.85. PubMed PMID: 14319489.

106. Shishido A, Honjo S, Suganuma M, Ohtaki S, Hikita M, Fujiwara T, et al. STUDIES ON INFECTIOUS MONONUCLEOSIS INDUCED IN THE MONKEY BY EXPERIMENTAL INFECTION WITH RICKETTSIA SENNETSU. I. CLINICAL OBSERVATIONS AND ETIOLOGICAL INVESTIGATIONS. Jpn J Med Sci Biol. 1965;18:73-83. Epub 1965/04/01. doi: 10.7883/yoken1952.18.73. PubMed PMID: 14322603.

107. Hirai K. [Study of infectious mononucleosis in Kumamoto Prefecture. 1. Isolation of a Rickettsia sennetsu-like organism from patients with epidemic glandular fever]. Kumamoto Igakkai Zasshi. 1966;40(11):1159-73. Epub 1966/11/25. PubMed PMID: 6013564.

108. Tedeschi G, Amici D, Murri O, Paparelli M. [Multiplication in vitro of Haemobartonella muris and H. canis]. G Mal Infett Parassit. 1967;19(12):997-1001. Epub 1967/12/01. PubMed PMID: 5611977.

109. Tedeschi G, Farabollini F. [Formation of Heinz bodies in presence of Haemobartonella muris: inhibition by peroxidase]. G Mal Infett Parassit. 1967;19(12):996-7. Epub 1967/12/01. PubMed PMID: 5611976.

110. van der Gulden WJI. Diurnal rhythm in egg production by Syphacia muris. Experimental Parasitology. 1967;21(3):344-7. doi: 10.1016/0014-4894(67)90094-X.

111. Langenberg KF, Bryant MP, Wolfe RS. Hydrogen-oxidizing methane bacteria. II. Electron microscopy. J Bacteriol. 1968;95(3):1124-9. Epub 1968/03/01. doi: 10.1128/jb.95.3.1124-1129.1968. PubMed PMID: 5643051; PubMed Central PMCID: PMCPMC252140.

112. Seo BS, Rim HJ, Yoon JJ, Koo BY, Hong NT. Studies On The Parasitic Helminths Of Korea: III. Nematodes And Cestodes Of Rodents. Kisaengchunghak Chapchi. 1968;6(3):123-31. Epub 1968/12/01. doi: 10.3347/kjp.1968.6.3.123. PubMed PMID: 12913546.

113. Lalova I. [Species specificity of Lamblia intestinalis and Lamblia muris]. Med Parazitol (Mosk). 1970;39(6):674-6. Epub 1970/11/01. PubMed PMID: 5516298.

114. Caldes G, Prescott B. A comparison of the chemical composition of Mycobacterium tuberculosis muris with Mycobacterium tuberculosis bovis (BCG). Biochem Biophys Res Commun. 1971;44(4):852-8. Epub 1971/08/20. doi: 10.1016/0006-291x(71)90789-3. PubMed PMID: 5001480.

115. Dehority BA. Carbon dioxide requirement of various species of rumen bacteria. J Bacteriol. 1971;105(1):70-6. Epub 1971/01/01. doi: 10.1128/jb.105.1.70-76.1971. PubMed PMID: 5541030; PubMed Central PMCID: PMCPMC248323.

116. Dijk JEv. Studies on Ehrlichia canis. Zentralblatt für Veterinärmedizin Reihe B. 1971;18(10):787-803. doi: 10.1111/j.1439-0450.1971.tb01656.x.

117. Kamio Y, Takahashi H. Chemical structure of lipid A of Selenomonas ruminantium. J Biochem. 1971;70(1):187-91. Epub 1971/07/01. doi: 10.1093/oxfordjournals.jbchem.a129619. PubMed PMID: 5562340.

118. Pike EH. Effects of solid and soluble diets upon Trichuris muris (Schrank, 1788) and DBA-2J mice. J Parasitol. 1972;58(2):257-64. Epub 1972/04/01. PubMed PMID: 4502557.

119. Reddy CA, Bryant MP, Wolin MJ. Characteristics of S organism isolated from Methanobacillus omelianskii. J Bacteriol. 1972;109(2):539-45. Epub 1972/02/01. doi: 10.1128/jb.109.2.539-545.1972. PubMed PMID: 5058442; PubMed Central PMCID: PMCPMC285174.

120. Buhles Jr WC, Huxsoll DL, Elisberg BL. Isolation of Rickettsia rickettsi in primary bone marrow cell and circulating monocyte cultures derived from experimentally infected guinea pigs. Infection and Immunity. 1973;7(6):1003-5. doi: 10.1128/iai.7.6.1003-1005.1973.

121. Holberton DV. Fine structure of the ventral disk apparatus and the mechanism of attachment in the flagellate Giardia muris. Journal of Cell Science. 1973;13(1):11-41.

122. Kingsley VV, Hoeniger JFM. Growth, structure, and classification of Selenomonas. Bacteriological Reviews. 1973;37(4):479-521. doi: 10.1128/mmbr.37.4.479-521.1973.

123. Scheifinger CC, Wolin MJ. Propionate formation from cellulose and soluble sugars by combined cultures of Bacteroides succinogenes and Selenomonas ruminantium. Journal of Applied Microbiology. 1973;26(5):789-95. doi: 10.1128/aem.26.5.789-795.1973.

124. Wojciechowicz M, Ziolecki A, Tomerska H. Electrophoretic patterns of whole cell proteins of some rumen bacteria. ACTA MICROBIOLPOLSERA. 1973;5(1):49-52.

125. De Vries W, Van Wijck Kapteyn WMC, Oosterhuis SKH. The presence and function of cytochromes in Selenomonas ruminantium, Anaerovibrio lipolytica and Veillonella alcalescens. Journal of General Microbiology. 1974;81(1):69-78. doi: 10.1099/00221287-81-1-69.

126. Maluszynska GM, Janota Bassalik L. A cellulolytic rumen bacterium, Micromonospora ruminantium sp.nov. Journal of General Microbiology. 1974;82(1):57-65. doi: 10.1099/00221287-82-1-57.

127. Varel VH, Bryant MP, Holdeman LV, Moore WE. Isolation of ureolytic Peptostreptococcus productus from feces using defined medium; failure of common urease tests. Appl Microbiol. 1974;28(4):594-9. Epub 1974/10/01. doi: 10.1128/am.28.4.594-599.1974. PubMed PMID: 4213672; PubMed Central PMCID: PMCPMC186779.

128. Varel VH, Bryant MP, Holdeman LV, Moore WEC. Isolation of ureolytic Peptostreptococcus productus from feces using defined medium; failure of common urease tests. Journal of Applied Microbiology. 1974;28(4):594-9. doi: 10.1128/aem.28.4.594-599.1974.

129. Ilemobade AA, Blotkamp J, Synge BA. Preservation of Cowdria ruminantium at low temperatures. Research in Veterinary Science. 1975;19(3):337-8.

130. Scheifinger CC, Latham MJ, Wolin MJ. Relationship of lactate dehydrogenase specificity and growth rate to lactate metabolism by Selenomonas ruminantium. Journal of Applied Microbiology. 1975;30(6):916-21.

131. Scheifinger CC, Linehan B, Wolin MJ. H2 production by Selenomonas ruminantium in the absence and presence of methanogenic bacteria. Appl Microbiol. 1975;29(4):480-3. Epub 1975/04/01. doi: 10.1128/am.29.4.480-483.1975. PubMed PMID: 804850; PubMed Central PMCID: PMCPMC187010.

132. Solov'ev MM. [Biometric study of Lamblia trophozoites of mammals in connection with taxonomic problems of the genus Lamblia]. Parazitologiia. 1975;9(5):449-56. Epub 1975/09/01. PubMed PMID: 1103064.

133. Solovjev MM. Biometrical studies of trophozoites of Lamblia from mammals in regard to the taxonomy of the genus Lamblia (Russian). Parazitologiya. 1975;9(5):449-56.

134. Tzeng SF, Bryant MP, Wolfe RS. Factor 420 dependent pyridine nucleotide linked formate metabolism of Methanobacterium ruminantium. Journal of Bacteriology. 1975;121(1):192-6.

135. Tzeng SF, Wolfe RS, Bryant MP. Factor 420-dependent pyridine nucleotide-linked hydrogenase system of Methanobacterium ruminantium. J Bacteriol. 1975;121(1):184-91. Epub 1975/01/01. doi: 10.1128/jb.121.1.184-191.1975. PubMed PMID: 234934; PubMed Central PMCID: PMCPMC285629.

136. Tzeng SF, Wolfe RS, Bryant MP. Factor 420 dependent pyridine nucleotide linked hydrogenase system on Methanobacterium ruminantium. Journal of Bacteriology. 1975;121(1):184-91.

137. Tzing SF, Bryant MP, Wolfe RS. Factor 420-dependent pyridine nucleotide-linked formate metabolism of Methanobacterium ruminantium. J Bacteriol. 1975;121(1):192-6. Epub 1975/01/01. doi: 10.1128/jb.121.1.192-196.1975. PubMed PMID: 234935; PubMed Central PMCID: PMCPMC285630.

138. Balch WE, Wolfe RS. New approach to the cultivation of methanogenic bacteria: 2-mercaptoethanesulfonic acid (HS-CoM)-dependent growth of Methanobacterium ruminantium in a pressureized atmosphere. Appl Environ Microbiol. 1976;32(6):781-91. Epub 1976/12/01. doi: 10.1128/aem.32.6.781-791.1976. PubMed PMID: 827241; PubMed Central PMCID: PMCPMC170461.

139. Balch WE, Wolfe RS. New approach to the cultivation of methanogenic bacteria: 2 mercaptoethanesulfonic acid (HS CoM) dependent growth of Methanobacterium ruminantium in a pressurized atmosphere. Applied and Environmental Microbiology. 1976;32(6):781-91. doi: 10.1128/aem.32.6.781-791.1976.

140. De Carneri I, Candia Carnevali MD, Trane F. Ultrastructural aspects of the effect of Nimorazole on Giardia muris. Giornale di Malattie Infettive e Parassitarie. 1976;28(8):503-11.

141. De Carneri I, Trane F. Orally induced infection of mice with single Giardia muris trophozoites. Parassitologia. 1976;18(1-3):13-8.

142. Dubey JP. A review of Sarcocystis of domestic animals and of other coccidia of cats and dogs. J Am Vet Med Assoc. 1976;169(10):1061-78. Epub 1976/11/15. PubMed PMID: 824260.

143. Tachibana N, Kusaba T, Matsumoto L, Kobayashi Y. Purification of complement fixing antigens of Rickettsia sennetsu by ether treatment. Infection and Immunity. 1976;13(4):1030-6. doi: 10.1128/iai.13.4.1030-1036.1976.

144. Van Der Gulden WJI, Van Aspert Van Erp AJM. Syphacia muris: water permeability of eggs and its effect on hatching. Experimental Parasitology. 1976;39(1):40-4. doi: 10.1016/0014-4894(76)90007-2.

145. Van Der Gulden WJL, Van Aspert Van Erp AJM. Syphacia muris: response to environmental stimuli when hatching in vitro. Experimental Parasitology. 1976;39(1):45-50. doi: 10.1016/0014-4894(76)90008-4.

146. Balch WE, Magrum LJ, Fox GE, Wolfe RS, Woese CR. An ancient divergence among the bacteria. J Mol Evol. 1977;9(4):305-11. Epub 1977/08/05. doi: 10.1007/bf01796092. PubMed PMID: 408502.

147. Chen M, Wolin MJ. Influence of CH4 production by Methanobacterium ruminantium on the fermentation of glucose and lactate by Selenomonas ruminantium. Appl Environ Microbiol. 1977;34(6):756-9. Epub 1977/12/01. doi: 10.1128/aem.34.6.756-759.1977. PubMed PMID: 596874; PubMed Central PMCID: PMCPMC242743.

148. Cheng KJ, Costerton JW. Alkaline phosphatase activity of rumen bacteria. Applied and Environmental Microbiology. 1977;34(5):586-90. doi: 10.1128/aem.34.5.586-590.1977.

149. Latham MJ, Wolin MJ. Fermentation of cellulose by Ruminococcus flavefaciens in the presence and absence of Methanobacterium ruminantium. Applied and Environmental Microbiology. 1977;34(3):297-301. doi: 10.1128/aem.34.3.297-301.1977.

150. Levine ND. Nomenclature of Sarcocystis in the ox and sheep and of fecal coccidia of the dog and cat. Journal of Parasitology. 1977;63(1):36-51. doi: 10.2307/3280101.

151. Schottelius J. [Latex-Chagestest-reactions of immunsera against Bartonella bacilliformis, Haemobartonella muris and Eperythrozoon coccoides (author's transl)]. Tropenmed Parasitol. 1977;28(2):202-4. Epub 1977/06/01. PubMed PMID: 888185.

152. Schottelius J. Latex Chagastest reactions of immunsera against Bartonella bacilliformis, Haemobartonella muris and Eperythrozoon coccoides. Tropenmedizin und Parasitologie. 1977;28(2):202-4.

153. Sharapov MB, Soloviev MM. On susceptibility of mice and rats to human lamblia. An experimental study using a cultivation method. Meditsinskaya Parazitologiya i Parazitarnye Bolezni. 1977;46(4):457-61.

154. Akimova RF. Cavitary and parietal digestion in experimental lambliasis in white mice. Amylolytic activity in the small intestine. Meditsinskaya Parazitologiya i Parazitarnye Bolezni. 1978;47(5):54-9.

155. Allison MJ. Production of branched chain volatile fatty acids by certain anaerobic bacteria. Applied and Environmental Microbiology. 1978;35(5):872-7. doi: 10.1128/aem.35.5.872-877.1978.

156. Ashford RW. Who named Sarcocystis muris? Annals of Tropical Medicine and Parasitology. 1978;72(1):95.

157. Carosi G, Filice G, Carnevale G. Ultrastructural morphology and intrinsic pathogenetic mechanisms of Giardia lamblia. Giornale di Malattie Infettive e Parassitarie. 1978;30(6):489-500.

158. Carrillo JM, Green RA. A case report of canine ehrlichiosis: neutrophilic strain. Journal of the American Animal Hospital Association. 1978;14(1):100-4.

159. Daniels L, Zeikus JG. One-carbon metabolism in methanogenic bacteria: analysis of short-term fixation products of 14CO2 and 14CH3OH incorporated into whole cells. J Bacteriol. 1978;136(1):75-84. Epub 1978/10/01. doi: 10.1128/jb.136.1.75-84.1978. PubMed PMID: 101522; PubMed Central PMCID: PMCPMC218634.

160. Forsberg CW. Effects of heavy metals and other trace elements on the fermentative activity of the rumen microflora and growth of functionally important rumen bacteria. Canadian Journal of Microbiology. 1978;24(3):298-306. doi: 10.1139/m78-050.

161. Ilemobade AA. The persistence of Cowdria ruminantium in the blood of recovered animals. Tropical Animal Health and Production. 1978;10(3):170.

162. Kandler O, König H. Chemical composition of the peptidoglycan-free cell walls of methanogenic bacteria. Arch Microbiol. 1978;118(2):141-52. Epub 1978/08/01. doi: 10.1007/bf00415722. PubMed PMID: 697504.

163. Kunstyr I, Ammerpohl E. Resistance of faecal cysts of Spironucleus muris to some physical factors and chemical substances. Laboratory Animals. 1978;12(2):95-7. PubMed Central PMCID: PMCbacillolfabrik(Germany)

Pharmacia(Sweden)

Specia(France).

164. Lai JS, Okuda S, Takahashi H. β-Acyloxy fatty acids as components of lipid A from Selenomonas ruminantium. Agricultural and Biological Chemistry. 1978;42(7):1441-2.

165. Linehan B, Scheifinger CC, Wolin MJ. Nutritional Requirements of Selenomonas ruminantium for Growth on Lactate, Glycerol, or Glucose. Appl Environ Microbiol. 1978;35(2):317-22. Epub 1978/02/01. doi: 10.1128/aem.35.2.317-322.1978. PubMed PMID: 16345271; PubMed Central PMCID: PMCPMC242832.

166. Tachibana N, Kusune E, Tsuda K, Tamari K, Fukushima I, Minagoshi S, et al. [Immunological study on Rickettsia sennetsu by complement-fixation test (author's transl)]. Kansenshogaku Zasshi. 1978;52(2):50-5. Epub 1978/02/01. doi: 10.11150/kansenshogakuzasshi1970.52.50. PubMed PMID: 101610.

167. Wallace RJ. Control of lactate production by Selenomonas ruminantium: homotropic activation of lactate dehydrogenase by pyruvate. J Gen Microbiol. 1978;107(1):45-52. Epub 1978/07/01. doi: 10.1099/00221287-107-1-45. PubMed PMID: 103995.

168. Wimpenny JWT, Samah OA. Some effects of oxygen on the growth and physiology of Selenomonas ruminantium. Journal of General Microbiology. 1978;108(2):329-32. doi: 10.1099/00221287-108-2-329.

169. Yamamoto S. [Studies on the causative agent of Hyuga-feveri; cultivation of rickettsia-like organisms isolated from metacercariae of Stellantchasumus falcatus in tissue culture cell and their antigenic relation to Rickettsia sennetsu (author's transl)]. Kansenshogaku Zasshi. 1978;52(7):240-5. Epub 1978/07/01. doi: 10.11150/kansenshogakuzasshi1970.52.240. PubMed PMID: 102710.

170. Allison MJ, Robinson IM, Baetz AL. Synthesis of alpha-ketoglutarate by reductive carboxylation of succinate in Veillonella, Selenomonas, and Bacteriodes species. J Bacteriol. 1979;140(3):980-6. Epub 1979/12/01. doi: 10.1128/jb.140.3.980-986.1979. PubMed PMID: 533772; PubMed Central PMCID: PMCPMC216742.

171. Allison MJ, Robinson IM, Baetz AL. Synthesis of α-ketoglutarate by reductive carboxylation of succinate in Veillonella, Selenomonas, and Bacteroides species. Journal of Bacteriology. 1979;140(3):980-6.

172. Balch WE, Wolfe RS. Specificity and biological distribution of coenzyme M (2-mercaptoethanesulfonic acid). J Bacteriol. 1979;137(1):256-63. Epub 1979/01/01. doi: 10.1128/jb.137.1.256-263.1979. PubMed PMID: 104960; PubMed Central PMCID: PMCPMC218444.

173. Balch WE, Wolfe RS. Transport of coenzyme M (2-mercaptoethanesulfonic acid) in Methanobacterium ruminantium. J Bacteriol. 1979;137(1):264-73. Epub 1979/01/01. doi: 10.1128/jb.137.1.264-273.1979. PubMed PMID: 33148; PubMed Central PMCID: PMCPMC218445.

174. Chen M, Wolin MJ. Effect of monensin and lasalocid-sodium on the growth of methanogenic and rumen saccharolytic bacteria. Appl Environ Microbiol. 1979;38(1):72-7. Epub 1979/07/01. doi: 10.1128/aem.38.1.72-77.1979. PubMed PMID: 16345418; PubMed Central PMCID: PMCPMC243437.

175. De Carneri I, Trane F. Infection of mice with trophozoites of Giardia muris administered with food. Parassitologia. 1979;21(1-3):108.

176. Eirich LD, Vogels GD, Wolfe RS. Distribution of coenzyme F420 and properties of its hydrolytic fragments. J Bacteriol. 1979;140(1):20-7. Epub 1979/10/01. doi: 10.1128/jb.140.1.20-27.1979. PubMed PMID: 40952; PubMed Central PMCID: PMCPMC216774.

177. Frenkel JK, Heydorn AO, Mehlhorn H, Rommel M. Sarcocystinae: nomina dubia and available names. Z Parasitenkd. 1979;58(2):115-39. Epub 1979/02/28. doi: 10.1007/bf01951337. PubMed PMID: 107666.

178. Herweg C, Kunstyr I. Effect of intestinal flagellate Spironucleus (Hexamita) muris and of dimetridazole on intestinal microflora in thymus-deficient (nude) mice. Zentralblatt fur Bakteriologie Mikrobiologie und Hygiene - Abt 1 Orig A. 1979;245(1-2):262-9.

179. Minenkova EA, Fomina MM, Poroshenko GG, Evsejenko LS. Polymorphism of the tumor cell population and selective processes. IV. Effect of dibunol and methyl-N-nitrosourea on the variability of a tumor cell population of the Ehrlich-I Ch. Ph. ascites strain. Tsitologiya. 1979;21(9):1081-6.

180. Nemanic PC, Owen RL, Stevens DP, Mueller JC. Ultrastructural observations on giardiasis in a mouse model. II. Endosymbiosis and organelle distribution in Giardia muris and Giardia lamblia. J Infect Dis. 1979;140(2):222-8. Epub 1979/08/01. doi: 10.1093/infdis/140.2.222. PubMed PMID: 479640.

181. Russell JB, Baldwin RL. Comparison of maintenance energy expenditures and growth yields among several rumen bacteria grown on continuous culture. Appl Environ Microbiol. 1979;37(3):537-43. Epub 1979/03/01. doi: 10.1128/aem.37.3.537-543.1979. PubMed PMID: 16345359; PubMed Central PMCID: PMCPMC243251.

182. Russell JB, Baldwin RL. Comparison of substrate affinities among several rumen bacteria: a possible determinant of rumen bacterial competition. Appl Environ Microbiol. 1979;37(3):531-6. Epub 1979/03/01. doi: 10.1128/aem.37.3.531-536.1979. PubMed PMID: 16345358; PubMed Central PMCID: PMCPMC243250.

183. Russell JB, Delfino FJ, Baldwin RL. Effects of combinations of substrates on maximum growth rates of several rumen bacteria. Appl Environ Microbiol. 1979;37(3):544-9. Epub 1979/03/01. doi: 10.1128/aem.37.3.544-549.1979. PubMed PMID: 16345360; PubMed Central PMCID: PMCPMC243252.

184. Sahin I. [Parasitosis and zoonosis in mice and rats caught in and around Beytepe Village (author's transl)]. Mikrobiyol Bul. 1979;13(3):283-90. Epub 1979/07/01. PubMed PMID: 553997.

185. Sauer FD, Erfle JD, Mahadevan S. Methane synthesis without the addition of adenosine triphosphate by cell membranes isolated from Methanobacterium ruminantium. Biochem J. 1979;178(1):165-72. Epub 1979/01/15. doi: 10.1042/bj1780165. PubMed PMID: 435275; PubMed Central PMCID: PMCPMC1186493.

186. Stevenson IL. The effect of l-α-amino-n-butyric acid on growth and production of extracellular isoleucine and valine by Eubacterium ruminantium and a related rumen isolate. Canadian Journal of Microbiology. 1979;25(12):1394-400. doi: 10.1139/m79-218. PubMed Central PMCID: PMCSigma.

187. Tachnibana N, Kusune E, Yokota T, Ogawa A, Fukuma M, Hisano S, et al. [Study on sennetsu rickettsiosis: Seroepidemiological survey of Rickettsia sennetsu in Miyazaki District (author's transl)]. Kansenshogaku Zasshi. 1979;53(5):228-33. Epub 1979/05/01. doi: 10.11150/kansenshogakuzasshi1970.53.228. PubMed PMID: 119016.

188. Williams AG. The selectivity of carbohydrate assimilation by the anaerobic rumen ciliate Dasytricha ruminantium. Journal of Applied Bacteriology. 1979;47(3):511-20.

189. Zehnder AJ, Brock TD. Methane formation and methane oxidation by methanogenic bacteria. J Bacteriol. 1979;137(1):420-32. Epub 1979/01/01. doi: 10.1128/jb.137.1.420-432.1979. PubMed PMID: 762019; PubMed Central PMCID: PMCPMC218466.

190. Brugerolle G, Kunstyr I, Senaud J, Friedhoff KT. Fine structure of trophozoites and cysts of the pathogenic diplomonad Spironucleus muris. Zeitschrift fur Parasitenkunde. 1980;62(1):47-61.

191. Edlinger EA, Benichou J, Labrune B. Positive Ehrlichia canis serology in Kawasaki disease. Lancet. 1980;1(8178):1146-7. Epub 1980/05/24. doi: 10.1016/s0140-6736(80)91603-7. PubMed PMID: 6103489.

192. Henderson C. The influence of extracellular hydrogen on the metabolism of Bacteroides ruminicola, Anaerovibrio lipolytica and Selenomonas ruminantium. J Gen Microbiol. 1980;119(2):485-91. Epub 1980/08/01. doi: 10.1099/00221287-119-2-485. PubMed PMID: 6785381.

193. Kamio Y, Takahashi H. Outer membrane proteins and cell surface structure of Selenomonas ruminantium. J Bacteriol. 1980;141(2):899-907. Epub 1980/02/01. doi: 10.1128/jb.141.2.899-907.1980. PubMed PMID: 7364720; PubMed Central PMCID: PMCPMC293701.

194. Kamio Y, Takahashi H. Isolation and characterization of outer and inner membranes of Selenomonas ruminantium: lipid compositions. J Bacteriol. 1980;141(2):888-98. Epub 1980/02/01. doi: 10.1128/jb.141.2.888-898.1980. PubMed PMID: 7364719; PubMed Central PMCID: PMCPMC293700.

195. Kamoi Y, Takahashi H. Outer membrane proteins and cell surface structure of Selenomonas ruminantium. Journal of Bacteriology. 1980;141(2):899-907.

196. Krzycki J, Zeikus JG. Quantification of corrinoids in methanogenic bacteria. Curr Microbiol. 1980;3(4):243-5. Epub 1980/07/01. doi: 10.1007/bf02602456. PubMed PMID: 27520770.

197. Minter-Goedbloed E, Franca S, Draper CC. The latex agglutination for Trypanosoma cruzi: Unsuitable for testing animals. Journal of Tropical Medicine and Hygiene. 1980;83(4):157-60.

198. Nyindo M, Huxsoll DL, Ristic M, Kakoma I, Brown JL, Carson CA, et al. Cell-mediated and humoral immune responses of German Shepherd Dogs and Beagles to experimental infection with Ehrlichia canis. American Journal of Veterinary Research. 1980;41(2):250-4.

199. Roberts-Thomson IC, Mitchell GF, Anders RF. Genetic studies in human and murine giardiasis. Gut. 1980;21(5):397-401. doi: 10.1136/gut.21.5.397.

200. Roberts-Thomson IC, Mitchell GF, Anders RF, Tait BD, Kerlin P, Kerr-Grant A, et al. Genetic studies in human and murine giardiasis. Gut. 1980;21(5):397-401. Epub 1980/05/01. doi: 10.1136/gut.21.5.397. PubMed PMID: 6933131; PubMed Central PMCID: PMCPMC1419092.

201. Ross CR, Wagner JE, Wightman SR, Dill SE. Experimental transmission of Syphacia muris among rats, mice, hamsters and gerbils. Laboratory Animal Science. 1980;30(1):35-7.

202. Smith CJ, Hespell RB, Bryant MP. Ammonia assimilation and glutamate formation in the anaerobe Selenomonas ruminantium. Journal of Bacteriology. 1980;141(2):593-602. doi: 10.1128/jb.141.2.593-602.1980.

203. Stephenson EH, Osterman JV. Somatic cell hybrids of canine peritoneal macrophages and SV40-transformed human cells: derivation, characterization, and infection with Ehrlichia canis. Am J Vet Res. 1980;41(2):234-40. Epub 1980/02/01. PubMed PMID: 6245605.

204. Stephenson EH, Osterman JV. SOMATIC-CELL HYBRIDS OF CANINE PERITONEAL-MACROPHAGES AND SV40-TRANSFORMED HUMAN-CELLS - DERIVATION, CHARACTERIZATION, AND INFECTION WITH EHRLICHIA-CANIS. American Journal of Veterinary Research. 1980;41(2):234-40. PubMed PMID: WOS:A1980JF18100016.

205. Troy GC, Vulgamott JC, Turnwald GH. Canine ehrlichiosis: a retrospective study of 30 naturally occurring cases. Journal of the American Animal Hospital Association. 1980;16(2):181-7.

206. Wallace RJ. Cytoplasmic reserve polysaccharide of Selenomonas ruminantium. Applied and Environmental Microbiology. 1980;39(3):630-4. doi: 10.1128/aem.39.3.630-634.1980.

207. Ward TE, Frea JI. Sediment distribution of methanogenic bacteria in lake erie and cleveland harbor. Appl Environ Microbiol. 1980;39(3):597-603. Epub 1980/03/01. doi: 10.1128/aem.39.3.597-603.1980. PubMed PMID: 16345529; PubMed Central PMCID: PMCPMC291384.

208. Kamio Y, Itoh Y, Terawaki Y. Chemical structure of peptidoglycan in Selenomonas ruminantium: cadaverine links covalently to the D-glutamic acid residue of peptidoglycan. J Bacteriol. 1981;146(1):49-53. Epub 1981/04/01. doi: 10.1128/jb.146.1.49-53.1981. PubMed PMID: 6783621; PubMed Central PMCID: PMCPMC217050.

209. Kamio Y, Itoh Y, Terawaki Y, Kusano T. Cadaverine is covalently linked to peptidoglycan in Selenomonas ruminantium. J Bacteriol. 1981;145(1):122-8. Epub 1981/01/01. doi: 10.1128/jb.145.1.122-128.1981. PubMed PMID: 7462141; PubMed Central PMCID: PMCPMC217252.

210. Kamio Y, Terawaki Y. Penicillin-binding proteins in Selenomonas ruminantium. Agricultural and Biological Chemistry. 1981;45(4):993-5. PubMed Central PMCID: PMCRadiochemical Centre(United Kingdom).

211. Maczulak AE, Dehority BA, Palmquist DL. Effects of long-chain Fatty acids on growth of rumen bacteria. Appl Environ Microbiol. 1981;42(5):856-62. Epub 1981/11/01. doi: 10.1128/aem.42.5.856-862.1981. PubMed PMID: 16345887; PubMed Central PMCID: PMCPMC244119.

212. McInerney MJ, Bryant MP. Anaerobic Degradation of Lactate by Syntrophic Associations of Methanosarcina barkeri and Desulfovibrio Species and Effect of H(2) on Acetate Degradation. Appl Environ Microbiol. 1981;41(2):346-54. Epub 1981/02/01. doi: 10.1128/aem.41.2.346-354.1981. PubMed PMID: 16345708; PubMed Central PMCID: PMCPMC243697.

213. Mink RW, Hespell RB. Long-term nutrient starvation of continuously cultured (glucose-limited) Selenomonas ruminantium. J Bacteriol. 1981;148(2):541-50. Epub 1981/11/01. doi: 10.1128/jb.148.2.541-550.1981. PubMed PMID: 6170629; PubMed Central PMCID: PMCPMC216238.

214. Owen RL, Allen CL, Stevens DP. Phagocytosis of Giardia muris by macrophages in Peyer's patch epithelium in mice. Infection and Immunity. 1981;33(2):591-601. doi: 10.1128/iai.33.2.591-601.1981.

215. Reardon MJ, Pierce KR. Acute experimental canine ehrlichiosis. I. Sequential reaction of the hemic and lymphoreticular systems. Veterinary Pathology. 1981;18(1):48-61.

216. Ristic M, Huxsoll DL, Tachibana N, Rapmund G. Evidence of a serologic relationship between Ehrlichia canis and Rickettsia sennetsu. Am J Trop Med Hyg. 1981;30(6):1324-8. Epub 1981/11/01. doi: 10.4269/ajtmh.1981.30.1324. PubMed PMID: 7034563.

217. Seo BS, Cho SY, Hong ST, Hong SJ, Lee SH. Studies On Parasitic Helminths Of Korea 5.Survey On Intestinal Trematodes Of House Rats. Kisaengchunghak Chapchi. 1981;19(2):131-6. Epub 1981/12/01. doi: 10.3347/kjp.1981.19.2.131. PubMed PMID: 12902707.

218. Smith CJ, Hespell RB, Bryant MP. Regulation of urease and ammonia assimilatory enzymes in Selenomonas ruminantium. Appl Environ Microbiol. 1981;42(1):89-96. Epub 1981/07/01. doi: 10.1128/aem.42.1.89-96.1981. PubMed PMID: 6114707; PubMed Central PMCID: PMCPMC243968.

219. Brett SJ, Cox FEG. Immunological aspects of Giardia muris and Spironucleus muris infections in inbred and outbred strains of laboratory mice: a comparative study. Parasitology. 1982;85(1):85-99.

220. Harborth PB, Hanert HH. Isolation of Selenomonas ruminantium from an aquatic ecosystem. Archives of Microbiology. 1982;132(2):135-40.

221. Hoilien CA, Ristic M, Huxsoll DL, Rapmund G. Rickettsia sennetsu in human blood monocyte cultures: similarities to the growth cycle of Ehrlichia canis. Infect Immun. 1982;35(1):314-9. Epub 1982/01/01. doi: 10.1128/iai.35.1.314-319.1982. PubMed PMID: 6274798; PubMed Central PMCID: PMCPMC351031.

222. Hoilien CA, Ristic M, Huxsoll DL, Rapmund G. RICKETTSIA-SENNETSU IN HUMAN-BLOOD MONOCYTE CULTURES - SIMILARITIES TO THE GROWTH-CYCLE OF EHRLICHIA-CANIS. Infection and Immunity. 1982;35(1):314-9. doi: 10.1128/iai.35.1.314-319.1982. PubMed PMID: WOS:A1982MW48200046.

223. Kamio Y, Terawaki Y, Izaki K. Biosynthesis of cadaverine-containing peptidoglycan in Selenomonas ruminantium. J Biol Chem. 1982;257(6):3326-33. Epub 1982/03/25. PubMed PMID: 7037782.

224. Keefe TJ, Holland CJ, Salyer PE, Ristic M. Distribution of Ehrlichia canis among military working dogs in the world and selected civilian dogs in the United States. Journal of the American Veterinary Medical Association. 1982;181(3):236-8.

225. Lloyd D, Williams J, Yarlett N, Williams AG. Oxygen affinities of the hydrogenosome-containing protozoa Tritrichomonas foetus and Dasytricha ruminantium, and two aerobic protozoa, determined by bacterial bioluminescence. Journal of General Microbiology. 1982;128(5):1019-22. doi: 10.1099/00221287-128-5-1019.

226. Mink RW, Patterson JA, Hespell RB. Changes in Viability, Cell Composition, and Enzyme Levels During Starvation of Continuously Cultured (Ammonia-Limited) Selenomonas ruminantium. Appl Environ Microbiol. 1982;44(4):913-22. Epub 1982/10/01. doi: 10.1128/aem.44.4.913-922.1982. PubMed PMID: 16346116; PubMed Central PMCID: PMCPMC242117.

227. Samah OA, Wimpenny JWT. Some effects of oxygen on the physiology of Selenomonas ruminantium WPL 151/1 grown in continuous culture. Journal of General Microbiology. 1982;128(2):355-60. doi: 10.1099/00221287-128-2-355.

228. Watanabe T, Okuda SI, Takahashi H. Physiological importance of even-numbered fatty acids and aldehydes in plasmalogen phospholipids of Selenomonas ruminantium. Journal of General and Applied Microbiology. 1982;28(1):23-33.

229. Yarlett N, Lloyd D, Williams AG. Respiration of the rumen ciliate Dasytricha ruminantium Schuberg. Biochem J. 1982;206(2):259-66. Epub 1982/08/15. doi: 10.1042/bj2060259. PubMed PMID: 6293462; PubMed Central PMCID: PMCPMC1158581.

230. Caldwell DR, Rasmussen CK. Alpha-ketoglutarate metabolism by cytochrome-containing anaerobes. Can J Microbiol. 1983;29(7):790-6. Epub 1983/07/01. doi: 10.1139/m83-128. PubMed PMID: 6413047.

231. Faubert GM, Belosevic M, Walker TS, MacLean JD, Meerovitch E. Comparative studies on the pattern of infection with Giardia spp. in mongolian gerbils. J Parasitol. 1983;69(5):802-5. Epub 1983/10/01. PubMed PMID: 6672162.

232. Gardner RM, Fuller MD, Caldwell DR. Tetrapyrrole utilization by protoheme-synthesizing anaerobes. Current Microbiology. 1983;9(2):59-61.

233. Kamio Y, Terawaki Y. Purification and properties of Selenomonas ruminantium lysine decarboxylase. J Bacteriol. 1983;153(2):658-64. Epub 1983/02/01. doi: 10.1128/jb.153.2.658-664.1983. PubMed PMID: 6401702; PubMed Central PMCID: PMCPMC221682.

234. Lee TD, Wakelin D. Cortisone-induced immunotolerance to nematode infection in CBA/Ca mice. II. A model for human chronic trichuriasis. Immunology. 1983;48(3):571-7.

235. Marchin GL, Fina LR, Lambert JL, Fina GT. Effect of resin disinfectants-I3 and -I5 on Giardia muris and Giardia lamblia. Applied and Environmental Microbiology. 1983;46(5):965-9. doi: 10.1128/aem.46.5.965-969.1983.

236. Avakyan AA, Popov VL. Rickettsiaceae and Chlamydiaceae: comparative electron microscopic studies. Acta Virol. 1984;28(2):159-73. Epub 1984/03/01. PubMed PMID: 6145350.

237. Coggins JR, Schaefer Iii FW. Giardia muris: Scanning electron microscopy of in vitro excystation. Experimental Parasitology. 1984;57(1):62-7.

238. Entzeroth R. Electron microscope study of host-parasite interactions of Sarcocystis muris (Protozoa, Coccidia) in tissue culture and in vivo. Zeitschrift fur Parasitenkunde. 1984;70(1):131-4.

239. Hudman JF. Glucose-induced morphological variation in Selenomonas ruminantium. FEMS Microbiology Letters. 1984;22(3):201-4. doi: 10.1016/0378-1097(84)90009-0.

240. Hudman JF, Glenn AR. Selenite uptake and incorporation by Selenomonas ruminantium. Archives of Microbiology. 1984;140(2-3):252-6. doi: 10.1007/BF00454937.

241. Levine ND. Taxonomy and review of the coccidian genus Cryptosporidium (protozoa, apicomplexa). J Protozool. 1984;31(1):94-8. Epub 1984/02/01. doi: 10.1111/j.1550-7408.1984.tb04296.x. PubMed PMID: 6376791.

242. Marounek M, Wallace RJ. Influence of culture E(h) on the growth and metabolism of the rumen bacteria Selenomonas ruminantium, Bacteroides amylophilus, Bacteroides succinogenes and Streptococcus bovis in batch culture. Journal of General Microbiology. 1984;130(2):223-9. doi: 10.1099/00221287-130-2-223.

243. Mehta KI, Callihan CD. Production of protein and fatty acids in the anaerobic fermentation of molasses by E. ruminantium. Journal of the American Oil Chemists' Society. 1984;61(11):1728-34. doi: 10.1007/BF02582137.

244. Peters JG. LOVE, HEAVEN, AND HUMAN EXISTENCE - A NOTE ON BROWNING,ELIZABETH,BARRETT 'SONNET XXII'. Studies in Browning and His Circle. 1984;12:32-3. PubMed PMID: WOS:A1984ACB2500005.

245. Rapmund G. Rickettsial diseases of the Far East: new perspectives. J Infect Dis. 1984;149(3):330-8. Epub 1984/03/01. doi: 10.1093/infdis/149.3.330. PubMed PMID: 6425420.

246. Sauch JF. Purification of Giardia muris cysts by velocity sedimentation. Appl Environ Microbiol. 1984;48(2):454-5. Epub 1984/08/01. doi: 10.1128/aem.48.2.454-455.1984. PubMed PMID: 6486790; PubMed Central PMCID: PMCPMC241540.

247. Silley P, Armstrong DG. Changes in metabolism and cell size of the anaerobic bacterium Selenomonas ruminantium 0078A at the onset of growth in continuous culture. Journal of Applied Bacteriology. 1984;56(3):487-92.

248. Spielman A, Levine JF, Wilson ML. Vectorial capacity of North American Ixodes ticks. Yale J Biol Med. 1984;57(4):507-13. Epub 1984/07/01. PubMed PMID: 6516453; PubMed Central PMCID: PMCPMC2590044.

249. Watanabe T, Okuda S, Takahashi H. Turn-over of phospholipids in Selenomonas ruminantium. J Biochem. 1984;95(2):521-7. Epub 1984/02/01. doi: 10.1093/oxfordjournals.jbchem.a134634. PubMed PMID: 6325403.

250. Wickramanayake GB, Rubin AJ, Sproul OJ. Inactivation of Naegleria and Giardia cysts in water by ozonation. Journal of the Water Pollution Control Federation. 1984;56(8):983-8.

251. Belosevic M, Faubert GM, MacLean JD. Giardia muris-induced depression of the primary immune response in spleen and mesenteric lymph node cell cultures to sheep red blood cells. Parasite Immunology. 1985;7(5):467-78.

252. Chbouki N, Dubremetz JF. Structure, isolation, and protein composition of the pellicle of Sarcocystis muris cystozoites (protozoa, coccidia). Journal of Protozoology. 1985;32(1):54-8.

253. Cole AI, Ristic M, Lewis Jr GE, Rapmund G. Continuous propagation of Ehrlichia sennetsu in murine macrophage cell cultures. American Journal of Tropical Medicine and Hygiene. 1985;34(4):774-80. doi: 10.4269/ajtmh.1985.34.774.

254. Entzeroth R. Invasion and early development of Sarcocystis muris (apicomplexa, sarcocystidae) in tissue cultures. Journal of Protozoology. 1985;32(3):446-53.

255. Furio MM, Wordell CJ. Treatment of infectious complications of acquired immunodeficiency syndrome. Clin Pharm. 1985;4(5):539-54. Epub 1985/09/01. PubMed PMID: 2996829.

256. Holland CJ, Ristic M, Huxsoll DL, Cole AI, Rapmund G. Adaptation of Ehrlichia sennetsu to canine blood monocytes: preliminary structural and serological studies with cell culture-derived Ehrlichia sennetsu. Infect Immun. 1985;48(2):366-71. Epub 1985/05/01. doi: 10.1128/iai.48.2.366-371.1985. PubMed PMID: 2985504; PubMed Central PMCID: PMCPMC261315.

257. Hüster R, Gilles HH, Thauer RK. Is coenzyme M bound to factor F430 in methanogenic bacteria? Experiments with Methanobrevibacter ruminantium. Eur J Biochem. 1985;148(1):107-11. Epub 1985/04/01. doi: 10.1111/j.1432-1033.1985.tb08813.x. PubMed PMID: 3920049.

258. Kelly DJ, Lee M, Lewis GE, Jr. A light and electron microscopic examination of Ehrlichia sennetsu in cultured human endothelial cells. Jpn J Med Sci Biol. 1985;38(4):155-68. Epub 1985/08/01. doi: 10.7883/yoken1952.38.155. PubMed PMID: 3913788.

259. Kelly DJ, Lee M, Lewis GE. A Light And Electron Microscopic Examination Of Emlicnia Sennetsu In Cultured Human Endothelial Cells. Japanese Journal of Medical Science and Biology. 1985;38(4):155-68. doi: 10.7883/yoken1952.38.155.

260. Kelly DJ, Lee M, Lewis GE. A LIGHT AND ELECTRON-MICROSCOPIC EXAMINATION OF EHRLICHIA-SENNETSU IN CULTURED HUMAN-ENDOTHELIAL CELLS. Japanese Journal of Medical Science & Biology. 1985;38(4):155-68. doi: 10.7883/yoken1952.38.155. PubMed PMID: WOS:A1985AVY4800001.

261. Kelly DJ, Lee M, Lewis Jr GE. A light and electron microscopic examination of Ehrlichia sennetsu in cultured human endothelial cells. Japanese Journal of Medical Science and Biology. 1985;38(4):155-68.

262. Musisi FL, Hussein NA. Isolation, transmission and some serological aspects of Cowdria ruminantium (Kafue strain). Rev Sci Tech. 1985;4(1):131-7. Epub 1985/03/01. doi: 10.20506/rst.4.1.194. PubMed PMID: 32988012.

263. Rikihisa Y, Perry BD. Causative ehrlichial organisms in Potomac horse fever. Infection and Immunity. 1985;49(3):513-7. doi: 10.1128/iai.49.3.513-517.1985.

264. Russell JB. Enrichment and Isolation of Rumen Bacteria That Reduce trans- Aconitic Acid to Tricarballylic Acid. Appl Environ Microbiol. 1985;49(1):120-6. Epub 1985/01/01. doi: 10.1128/aem.49.1.120-126.1985. PubMed PMID: 16346691; PubMed Central PMCID: PMCPMC238355.

265. Silley P, Armstrong DG. Metabolism of the rumen bacterium Selenomonas ruminantium grown in continuous culture. Letters in Applied Microbiology. 1985;1(3):53-5.

266. Upton SJ, Current WL. The species of Cryptosporidium (Apicomplexa: cryptosporidiidae) infecting mammals. Journal of Parasitology. 1985;71(5):625-9. doi: 10.2307/3281435.

267. Ward HD, Alroy J, Lev BI, Keusch GT, Pereira MEA. Identification of chitin as a structural component of Giardia cysts. Infection and Immunity. 1985;49(3):629-34. doi: 10.1128/iai.49.3.629-634.1985.

268. Wickramanayake GB, Rubin AJ, Sproul OJ. Effects of ozone and storage temperature on Giardia cysts. Journal / American Water Works Association. 1985;77(8):74-7. doi: 10.1002/j.1551-8833.1985.tb05591.x.

269. Anderson JF, Magnarelli LA, Philip RN, Burgdorfer W. Rickettsia rickettsii and Rickettsia montana from ixodid ticks in Connecticut. American Journal of Tropical Medicine and Hygiene. 1986;35(1):187-91. doi: 10.4269/ajtmh.1986.35.187.

270. Chávez B, Knaippe F, Gonzalez-Mariscal L, Martínez-Palomo A. Giardia lamblia: Electrophysiology and ultrastructure of cytopathology in cultured epithelial cells. Experimental Parasitology. 1986;61(3):379-89. doi: 10.1016/0014-4894(86)90194-3.

271. Coggins JR, Schaefer Iii FW. Giardia muris: Ultrastructural analysis of in vitro excystation. Experimental Parasitology. 1986;61(2):219-28. doi: 10.1016/0014-4894(86)90155-4.

272. Edson CM, Farthing MJ, Thorley-Lawson DA, Keusch GT. An 88,000-Mr Giardia lamblia surface protein which is immunogenic in humans. Infect Immun. 1986;54(3):621-5. Epub 1986/12/01. doi: 10.1128/iai.54.3.621-625.1986. PubMed PMID: 3536744; PubMed Central PMCID: PMCPMC260214.

273. Edson CM, Farthing MJG, Thorley-Lawson DA, Keusch GT. An 88,000-M(r) Giardia lamblia surface protein which is immunogenic in humans. Infection and Immunity. 1986;54(3):621-5. doi: 10.1128/iai.54.3.621-625.1986.

274. Feely DE. A simplified method for in vitro excystation of Giardia muris. Journal of Parasitology. 1986;72(3):474-5. doi: 10.2307/3281691.

275. García Vila A, Crespo MD, Martínez C, Borrás R, Brinés J, García de Lomas J, et al. [Abdominal pain in childhood due to a Cryptosporidium parasitosis]. An Esp Pediatr. 1986;25(2):111-4. Epub 1986/08/01. PubMed PMID: 3752746.

276. Hausinger RP. Purification of a nickel-containing urease from the rumen anaerobe Selenomonas ruminantium. Journal of Biological Chemistry. 1986;261(17):7866-70.

277. Kamio Y, Pösö H, Terawaki Y, Paulin L. Cadaverine covalently linked to a peptidoglycan is an essential constituent of the peptidoglycan necessary for the normal growth in Selenomonas ruminantium. J Biol Chem. 1986;261(14):6585-9. Epub 1986/05/15. PubMed PMID: 3084485.

278. O'Donoghue PJ, Adams M, Dixon BR, Ford GE, Baverstock PR. Morphological and biochemical correlates in the characterization of Sarcocystis spp. J Protozool. 1986;33(1):114-21. Epub 1986/02/01. doi: 10.1111/j.1550-7408.1986.tb05569.x. PubMed PMID: 3083101.

279. Rajasekariah GR, Deb BN, Dhage KR, Bose S. Response of laboratory-adapted human hookworm and other nematodes to ivermectin. Annals of Tropical Medicine and Parasitology. 1986;80(6):615-21. doi: 10.1080/00034983.1986.11812076.

280. New infection traced to ticks... ehrlichiosis. Emergency Medicine (00136654). 1987;19(14):48-53. PubMed PMID: 107564201. Language: English. Entry Date: 19871101. Revision Date: 20150712. Publication Type: Journal Article. Journal Subset: Allied Health.

281. Buchel LA, Gorenflot A, Chochillon C, Savel J, Gobert JG. In vitro excystation of Giardia from humans: a scanning electron microscopy study. J Parasitol. 1987;73(3):487-93. Epub 1987/06/01. PubMed PMID: 3598798.

282. Conway De Macario E. Antigenic Diversity of Methanogenic Bacteria from Intestinal Tracts of Animals. Systematic and Applied Microbiology. 1987;9(3):210-3. doi: 10.1016/S0723-2020(87)80023-1.

283. Dutta SK, Rice RM, Hughes TD, Savage PK, Myrup AC. Detection of serum antibodies against Ehrlichia risticii in Potomac horse fever by enzyme-linked immunosorbent assay. Veterinary Immunology and Immunopathology. 1987;14(1):85-92. doi: 10.1016/0165-2427(87)90077-8.

284. Ellison 3rd RT. Colorado tick fever or ehrlichiosis. JAMA : the journal of the American Medical Association. 1987;258(13):1731-2.

285. Ellison RT, 3rd. Colorado tick fever or ehrlichiosis. Jama. 1987;258(13):1731-2. Epub 1987/10/02. PubMed PMID: 3041059.

286. Ewing SA, Johnson EM, Kocan KM. Human infection with Ehrlichia canis. N Engl J Med. 1987;317(14):899-900. Epub 1987/10/01. doi: 10.1056/nejm198710013171412. PubMed PMID: 3627212.

287. Ewing SA, Johnson EM, Kocan KM. HUMAN INFECTION WITH EHRLICHIA-CANIS. New England Journal of Medicine. 1987;317(14):899-. PubMed PMID: WOS:A1987K173700017.

288. Ewing SA, Johnson EM, Kocan KM, Maeda K, Markowitz N, Hawley RC, et al. Human infection with Ehrlichia canis. New England Journal of Medicine. 1987;317(14):899-900.

289. Feely DE, Dyer JK. Localization of acid phosphatase activity in Giardia lamblia and Giardia muris trophozoites. Journal of Protozoology. 1987;34(1):80-3.

290. Fishbein DB, Sawyer LA, Holland CJ, Hayes EB, Okoroanyanwu W, Williams D, et al. Unexplained febrile illnesses after exposure to ticks. Infection with an Ehrlichia? Jama. 1987;257(22):3100-4. Epub 1987/06/12. PubMed PMID: 3586228.

291. Fishbein DB, Sawyer LA, Holland CJ, Hayes EB, Okoroanyanwu W, Williams D, et al. Unexplained Febrile Illnesses After Exposure to Ticks: Infection With an Ehrlichia? JAMA: The Journal of the American Medical Association. 1987;257(22):3100-4. doi: 10.1001/jama.1987.03390220098028.

292. Fishbein DB, Sawyer LA, McDade JE, Holland CJ, Dawson J, Ristic M. EHRLICHIA-CANIS INFECTION IN HUMANS - A NEW ZOONOSIS. Journal of the American Veterinary Medical Association. 1987;190(12):1614-. PubMed PMID: WOS:A1987H789300121.

293. Kamio Y. Structural specificity of diamines covalently linked to peptidoglycan for cell growth of Veillonella alcalescens and Selenomonas ruminantium. J Bacteriol. 1987;169(10):4837-40. Epub 1987/10/01. doi: 10.1128/jb.169.10.4837-4840.1987. PubMed PMID: 3654585; PubMed Central PMCID: PMCPMC213864.

294. Leahy JG, Rubin AJ, Sproul OJ. Inactivation of Giardia muris cysts by free chlorine. Applied and Environmental Microbiology. 1987;53(7):1448-53. doi: 10.1128/aem.53.7.1448-1453.1987.

295. Maeda K, Markowitz N, Hawley RC. Human infection with Ehrlichia canis, a leukocyte rickettsia. New England Journal of Medicine. 1987;316(14):853-6.

296. Maeda K, Markowitz N, Hawley RC, Ristic M, Cox D, McDade JE. Human infection with Ehrlichia canis, a leukocytic rickettsia. N Engl J Med. 1987;316(14):853-6. Epub 1987/04/02. doi: 10.1056/nejm198704023161406. PubMed PMID: 3029590.

297. Maeda K, Markowitz N, Hawley RC, Ristic M, McDade JE. HUMAN INFECTION WITH EHRLICHIA-CANIS - REPLY. New England Journal of Medicine. 1987;317(14):899-900. PubMed PMID: WOS:A1987K173700018.

298. Melville SB, Michel TA, Macy JM. Involvement of D-lactate and lactic acid racemase in the metabolism of glucose by Selenomonas ruminantium. FEMS Microbiology Letters. 1987;40(2-3):289-93.

299. Posada G, Pizarro D, Mohs E. [Oral rehydration in children with Cryptosporidium muris diarrhea]. Bol Med Hosp Infant Mex. 1987;44(12):740-4. Epub 1987/12/01. PubMed PMID: 3426779.

300. Todd MJ, Hausinger RP. Purification and characterization of the nickel-containing multicomponent urease from Klebsiella aerogenes. J Biol Chem. 1987;262(13):5963-7. Epub 1987/05/05. PubMed PMID: 3553184.

301. Ziemer EL, Whitlock RH, Palmer JE, Spencer PA. Clinical and hematologic variables in ponies with experimentally induced equine ehrlichial colitis (Potomac horse fever). American Journal of Veterinary Research. 1987;48(1):63-7.

302. Human ehrlichiosis--United States. MMWR Morb Mortal Wkly Rep. 1988;37(17):270, 5-7. Epub 1988/05/06. PubMed PMID: 3129641.

303. Human Ehrlichiosis—United States. Archives of Dermatology. 1988;124(7):993-4. doi: 10.1001/archderm.124.7.993.

304. Human ehrlichiosis--United States. MMWR Morbidity and mortality weekly report. 1988;37(17):270,5-7.

305. Caruana LB. Ehrlichia canis infections in humans. Clinical Laboratory Science. 1988;1(5):283-4.

306. Erlandsen SL, Sherlock LA, Januschka M, Schupp DG, Schaefer FW, 3rd, Jakubowski W, et al. Cross-species transmission of Giardia spp.: inoculation of beavers and muskrats with cysts of human, beaver, mouse, and muskrat origin. Appl Environ Microbiol. 1988;54(11):2777-85. Epub 1988/11/01. doi: 10.1128/aem.54.11.2777-2785.1988. PubMed PMID: 3063208; PubMed Central PMCID: PMCPMC204372.

307. Erlandsen SL, Sherlock LA, Januschka M, Schupp DG, Schaefer Iii FW, Jakubowski W, et al. Cross-species transmission of Giardia spp.: Inoculation of beavers and muskrats with cysts of human, beaver, mouse, and muskrat origin. Applied and Environmental Microbiology. 1988;54(11):2777-85. doi: 10.1128/aem.54.11.2777-2785.1988.

308. Fishbein DB, editor HUMAN EHRLICHIOSIS IN THE UNITED-STATES. Symp on Ehrlichiosis : A Vector-Borne Disease of Animals and Humans; 1988 Dec; Washington, Dc1990.

309. Goddard J. Was Bullis fever actually ehrlichiosis? Jama. 1988;260(20):3006-7. Epub 1988/11/25. PubMed PMID: 3184365.

310. Goddard J. The changing status of tickborne disease in the U.S. Military Medicine. 1988;153(10):513-9. doi: 10.1093/milmed/153.10.513.

311. Melville SB, Michel TA, Macy JM. Pathway and sites for energy conservation in the metabolism of glucose by Selenomonas ruminantium. J Bacteriol. 1988;170(11):5298-304. Epub 1988/11/01. doi: 10.1128/jb.170.11.5298-5304.1988. PubMed PMID: 3141385; PubMed Central PMCID: PMCPMC211604.

312. Melville SB, Michel TA, Macy JM. Regulation of carbon flow in Selenomonas ruminantium grown in glucose-limited continuous culture. J Bacteriol. 1988;170(11):5305-11. Epub 1988/11/01. doi: 10.1128/jb.170.11.5305-5311.1988. PubMed PMID: 3182729; PubMed Central PMCID: PMCPMC211605.

313. Pearce CJ, Conrad ME, Nolan PE, Fishbein DB, Dawson JE. Ehrlichiosis: a cause of bone marrow hypoplasia in humans. Am J Hematol. 1988;28(1):53-5. Epub 1988/05/01. doi: 10.1002/ajh.2830280111. PubMed PMID: 3369436.

314. Rikihisa Y, Jiang BM. In vitro susceptibilities of Ehrlichia risticii to eight antibiotics. Antimicrob Agents Chemother. 1988;32(7):986-91. Epub 1988/07/01. doi: 10.1128/aac.32.7.986. PubMed PMID: 3142345; PubMed Central PMCID: PMCPMC172330.

315. Rikihisa Y, Pretzman CI, Johnson GC, Reed SM, Yamamoto S, Andrews F. Clinical, histopathological, and immunological responses of ponies to Ehrlichia sennetsu and subsequent Ehrlichia risticii challenge. Infection and Immunity. 1988;56(11):2960-6.

316. Roach TI, Wakelin D, Else KJ, Bundy DA. Antigenic cross-reactivity between the human whipworm, Trichuris trichiura, and the mouse trichuroids Trichuris muris and Trichinella spiralis. Parasite Immunol. 1988;10(3):279-91. Epub 1988/05/01. doi: 10.1111/j.1365-3024.1988.tb00221.x. PubMed PMID: 3412784.

317. Roach TIA, Wakelin D, Else KJ, Bundy DAP. Antigenic cross-reactivity between the human whipworm, Trichuris trichiura, and the mouse trichuroids Trichuris muris and Trichinella spiralis. Parasite Immunology. 1988;10(3):279-91.

318. Roach TIA, Wakelin D, Else KJ, Bundy DAP. Antigenic cross‐reactivity between the human whipworm, Trichuris trichiura, and the mouse trichuroids Trichuris muris and Trichinella spiralis. Parasite Immunology. 1988;10(3):279-91. doi: 10.1111/j.1365-3024.1988.tb00221.x.

319. Roach TIA, Wakelin D, Else KJ, Bundy DAP. ANTIGENIC CROSS-REACTIVITY BETWEEN THE HUMAN WHIPWORM, TRICHURIS-TRICHIURA, AND THE MOUSE TRICHUROIDS TRICHURIS-MURIS AND TRICHINELLA-SPIRALIS. Parasite Immunology. 1988;10(3):279-91. doi: 10.1111/j.1365-3024.1988.tb00221.x. PubMed PMID: WOS:A1988N544100004.

320. Sharma AW, Mayrhofer G. A comparative study of infections with rodent isolates of Giardia duodenalis in inbred strains of rats and mice and in hypothymic nude rats. Parasite Immunology. 1988;10(2):169-79. doi: 10.1111/j.1365-3024.1988.tb00212.x.

321. Wells MY, Rikihisa Y. Lack of lysosomal fusion with phagosomes containing Ehrlichia risticii in P388D1 cells: abrogation of inhibition with oxytetracycline. Infect Immun. 1988;56(12):3209-15. Epub 1988/12/01. doi: 10.1128/iai.56.12.3209-3215.1988. PubMed PMID: 3182078; PubMed Central PMCID: PMCPMC259726.

322. Another tick-borne illness... ehrlichiosis, a rickettsial infection. Emergency Medicine (00136654). 1989;21(14):102-6. PubMed PMID: 107537371. Language: English. Entry Date: 19891101. Revision Date: 20150712. Publication Type: Journal Article. Journal Subset: Allied Health.

323. Brouqui P, Raoult D, Vidor E. Lack of co-transmission of Rickettsia conorii and Ehrlichia canis in human beings in the south of France. Eur J Epidemiol. 1989;5(1):110-2. Epub 1989/03/01. doi: 10.1007/bf00145056. PubMed PMID: 2707389.

324. Brouqui P, Raoult D, Vidor E. LACK OF CO-TRANSMISSION OF RICKETTSIA-CONORII AND EHRLICHIA-CANIS IN HUMAN-BEINGS IN THE SOUTH OF FRANCE. European Journal of Epidemiology. 1989;5(1):110-2. doi: 10.1007/bf00145056. PubMed PMID: WOS:A1989T789800020.

325. Caldwell DR. Effects of methanol on the growth of gastrointestinal anaerobes. Canadian Journal of Microbiology. 1989;35(2):313-7. doi: 10.1139/m89-047.

326. Conrad ME. Ehrlichia canis: a tick-borne rickettsial-like infection in humans living in the southeastern United States. Am J Med Sci. 1989;297(1):35-7. Epub 1989/01/01. doi: 10.1097/00000441-198901000-00008. PubMed PMID: 2643878.

327. Conrad ME. Review: Ehrlichia canis: A tick-borne rickettsial-like infection in humans living in the Southeastern United States. American Journal of the Medical Sciences. 1989;297(1):35-7. doi: 10.1097/00000441-198901000-00008.

328. Conrad ME. EHRLICHIA-CANIS - A TICK-BORNE RICKETTSIAL-LIKE INFECTION IN HUMANS LIVING IN THE SOUTHEASTERN UNITED-STATES. American Journal of the Medical Sciences. 1989;297(1):35-7. doi: 10.1097/00000441-198901000-00008. PubMed PMID: WOS:A1989T162000007.

329. Conrad ME. EHRLICHIOSIS - A CAUSE OF BONE-MARROW HYPOPLASIA IN HUMANS - REPLY. American Journal of Hematology. 1989;30(4):267-. PubMed PMID: WOS:A1989T677500017.

330. Dawson J, Fishbein D, Eng T, Redus M, Greene N, editors. DIAGNOSIS OF HUMAN EHRLICHIOSIS WITH THE INDIRECT FLUORESCENT-ANTIBODY TEST - KINETICS AND SPECIFICITY. 8th Sesquiannual Meeting of the American Soc for Rickettsiology and Rickettsial Diseases; 1989 Sep 22-26; Diamond Point, Ny1990.

331. Dean RG, Martin SA, Carver C. Isolation of plasmid DNA from the ruminal bacterium Selenomonas ruminantium HD4. Letters in Applied Microbiology. 1989;8(2):45-8.

332. DeRegnier DP, Cole L, Schupp DG, Erlandsen SL. Viability of Giardia cysts suspended in lake, river, and tap water. Applied and Environmental Microbiology. 1989;55(5):1223-9. doi: 10.1128/aem.55.5.1223-1229.1989.

333. Dimmitt DC, Fishbein DB, Dawson JE. Human ehrlichiosis associated with cerebrospinal fluid pleocytosis: a case report. Am J Med. 1989;87(6):677-8. Epub 1989/12/01. doi: 10.1016/s0002-9343(89)80404-8. PubMed PMID: 2589403.

334. Dutta SK, Mattingly BL, Shankarappa B. Antibody response to Ehrlichia risticii and antibody reactivity to the component antigens in horses with induced Potomac horse fever. Infect Immun. 1989;57(10):2959-62. Epub 1989/10/01. doi: 10.1128/iai.57.10.2959-2962.1989. PubMed PMID: 2777369; PubMed Central PMCID: PMCPMC260755.

335. Else KJ, Wakelin D, Roach TIA. Host predisposition to trichuriasis: The mouse—t. muris model. Parasitology. 1989;98(2):275-82. doi: 10.1017/S0031182000062193.

336. Eng TR, Fishbein DB, Dawson JE, Greene CR, Redus M, editors. SURVEILLANCE OF HUMAN EHRLICHIOSIS IN THE UNITED-STATES - 1988. 8th Sesquiannual Meeting of the American Soc for Rickettsiology and Rickettsial Diseases; 1989 Sep 22-26; Diamond Point, Ny1990.

337. Eng TR, Giles R. Ehrlichiosis. J Am Vet Med Assoc. 1989;194(4):497-500. Epub 1989/02/15. PubMed PMID: 2921197.

338. Entzeroth R, Chobotar B. A freeze-fracture study of the host cell-parasite interface during and after invasion of cultured cells by cystozoites of Sarcocystis muris. Eur J Protistol. 1989;25(2):89-99. Epub 1989/10/27. doi: 10.1016/s0932-4739(89)80020-3. PubMed PMID: 23195869.

339. Erlandsen SL, Bemrick WJ, Pawley J. High-resolution electron microscopic evidence for the filamentous structure of the cyst wall in Giardia muris and Giardia duodenalis. J Parasitol. 1989;75(5):787-97. Epub 1989/10/01. PubMed PMID: 2795382.

340. Fishbein DB, Kemp A, Dawson JE, Greene NR, Redus MA, Fields DH. Human ehrlichiosis: prospective active surveillance in febrile hospitalized patients. J Infect Dis. 1989;160(5):803-9. Epub 1989/11/01. doi: 10.1093/infdis/160.5.803. PubMed PMID: 2809255.

341. Fishbein DR, Kemp A, Dawson JE, Greene NR, Redus MA, Fields DH. Human ehrlichiosis: Prospective active surveillance in febrile hospitalized patients. Journal of Infectious Diseases. 1989;160(5):803-9.

342. Goddard J. Focus of human parasitism by the brown dog tick, Rhipicephalus sanguineus (Acari: Ixodidae). J Med Entomol. 1989;26(6):628-9. Epub 1989/11/01. doi: 10.1093/jmedent/26.6.628. PubMed PMID: 2585458.

343. Gordon Paul R, Butler RD, Williams AG. Ultrastructure of the rumen ciliate Dasytricha ruminantium. Eur J Protistol. 1989;24(3):205-15. Epub 1989/04/14. doi: 10.1016/s0932-4739(89)80057-4. PubMed PMID: 23195656.

344. Harkess JR. Ehrlichiosis: a cause of bone marrow hypoplasia in humans. Am J Hematol. 1989;30(4):265-7. Epub 1989/04/01. doi: 10.1002/ajh.2830300417. PubMed PMID: 2929590.

345. Harkess JR, Conrad ME. Ehrlichiosis: A cause of bone marrow hypoplasia in humans. American Journal of Hematology. 1989;30(4):265-6. doi: 10.1002/ajh.2830300417.

346. Harkess JR, Ewing SA, Crutcher JM, Kudlac J, Mc Kee G, Istre GR. Human Ehrlichiosis in Oklahoma. Journal of Infectious Diseases. 1989;159(3):576-9. doi: 10.1093/infdis/159.3.576.

347. Harkess JR, Ewing SA, Crutcher JM, Kudlac J, McKee G, Istre GR. Human ehrlichiosis in Oklahoma. J Infect Dis. 1989;159(3):576-9. Epub 1989/03/01. doi: 10.1093/infdis/159.3.576. PubMed PMID: 2915171.

348. Heyworth MF, Pappo J. Use of two-colour flow cytometry to assess killing of Giardia muris trophozoites by antibody and complement. Parasitology. 1989;99(2):199-203.

349. Iseki M, Maekawa T, Moriya K, Uni S, Takada S. Infectivity of Cryptosporidium muris (strain RN 66) in various laboratory animals. Parasitology Research. 1989;75(3):218-22. doi: 10.1007/BF00931279.

350. Kasprzak W, Pawlowski Z. Zoonotic aspects of giardiasis: a review. Vet Parasitol. 1989;32(2-3):101-8. Epub 1989/07/15. doi: 10.1016/0304-4017(89)90110-6. PubMed PMID: 2672545.

351. Martin SA, Dean RG. Characterization of a plasmid from the ruminal bacterium Selenomonas ruminantium. Applied and Environmental Microbiology. 1989;55(12):3035-8. doi: 10.1128/aem.55.12.3035-3038.1989. PubMed Central PMCID: PMCSigma(United States).

352. McCalmont C, Zanolli MD. Rickettsial diseases. Dermatol Clin. 1989;7(3):591-601. Epub 1989/07/01. PubMed PMID: 2665989.

353. McCloskey RV. Ehrlichiosis--"spotless spotted fever". Del Med J. 1989;61(7):335-7. Epub 1989/07/01. PubMed PMID: 2668039.

354. Mulder R, De Mattos MT, Neijssel OM. The mechanism of aggregate formation by Selenomonas ruminantium. Applied Microbiology and Biotechnology. 1989;32(3):350-5.

355. Paget TA, Jarroll EL, Manning P, Lindmark DG, Lloyd D. Respiration in the cysts and trophozoites of Giardia muris. Journal of General Microbiology. 1989;135(1):145-54. doi: 10.1099/00221287-135-1-145.

356. Rikihisa Y, editor GROWTH OF EHRLICHIA-RISTICII IN HUMAN COLONIC EPITHELIAL-CELLS. 8th Sesquiannual Meeting of the American Soc for Rickettsiology and Rickettsial Diseases; 1989 Sep 22-26; Diamond Point, Ny1990.

357. Riley ET, Stibbs HH. Antigenic conservation and variation in Giardia cysts from various vertebrate hosts. Western Journal of Medicine. 1989;151(4):403-5.

358. Schmidtmann ET. American dog tick: vector of the Potomac horse fever Rickettsia, Ehrlichia risticii? Parasitol Today. 1989;5(11):355-6. Epub 1989/11/01. doi: 10.1016/0169-4758(89)90109-9. PubMed PMID: 15463150.

359. Sreedhara Swamy KH. Filarial parasites exhibit unusually high levels of choline acetyltransferase activity. Molecular and Biochemical Parasitology. 1989;35(3):259-68. doi: 10.1016/0166-6851(89)90212-0.

360. Swamy KH. Filarial parasites exhibit unusually high levels of choline acetyltransferase activity. Mol Biochem Parasitol. 1989;35(3):259-68. Epub 1989/07/01. doi: 10.1016/0166-6851(89)90212-0. PubMed PMID: 2664508.

361. Weisburg WG, Dobson ME, Samuel JE, Dasch GA, Mallavia LP, Baca O, et al. Phylogenetic diversity of the Rickettsiae. J Bacteriol. 1989;171(8):4202-6. Epub 1989/08/01. doi: 10.1128/jb.171.8.4202-4206.1989. PubMed PMID: 2753854; PubMed Central PMCID: PMCPMC210191.

362. Weisburg WG, Tully JG, Rose DL, Petzel JP, Oyaizu H, Yang D, et al. A phylogenetic analysis of the mycoplasmas: basis for their classification. J Bacteriol. 1989;171(12):6455-67. Epub 1989/12/01. doi: 10.1128/jb.171.12.6455-6467.1989. PubMed PMID: 2592342; PubMed Central PMCID: PMCPMC210534.

363. Weisburg WG, Tully JG, Rose DL, Petzel JP, Oyaizu H, Yang D, et al. A phylogenetic analysis of the mycoplasmas: Basis for their classification. Journal of Bacteriology. 1989;171(12):6455-67. doi: 10.1128/jb.171.12.6455-6467.1989.

364. Wolfe RL, Stewart MH, Scott KN, McGuire MJ. Inactivation of Giardia muris and indicator organisms seeded in surface water supplies by peroxone and ozone. Environmental Science and Technology. 1989;23(6):744-5. doi: 10.1021/es00064a015.

365. Rocky Mountain spotted fever and human ehrlichiosis--United States, 1989. MMWR Morb Mortal Wkly Rep. 1990;39(17):281-4. Epub 1990/05/04. PubMed PMID: 2109175.

366. Rocky Mountain spotted fever and human ehrlichiosis - United States, 1989 Rocky Mountain spotted fever. Archives of Dermatology. 1990;126(7):858-9.

367. Rocky Mountain Spotted Fever and Human Ehrlichiosis—United States, 1989 Rocky Mountain Spotted Fever. Archives of Dermatology. 1990;126(7):859. doi: 10.1001/archderm.1990.01670310016001.

368. Barton LL, Dawson JE, Letson GW, Luisiri A, Scalzo AJ. Simultaneous ehrlichiosis and Lyme disease. Pediatr Infect Dis J. 1990;9(2):127-9. Epub 1990/02/01. doi: 10.1097/00006454-199002000-00011. PubMed PMID: 2314951.

369. Barton LL, Luisiri A, Dawson JE, Letson GW, Quan TJ. Simultaneous infection with an Ehrlichia and Borrelia burgdorferi in a child. Ann N Y Acad Sci. 1990;590:68-9. Epub 1990/01/01. doi: 10.1111/j.1749-6632.1990.tb42208.x. PubMed PMID: 2378481.

370. Bhat S, Wallace RJ, Orskov ER. Adhesion of cellulolytic ruminal bacteria to barley straw. Appl Environ Microbiol. 1990;56(9):2698-703. Epub 1990/09/01. doi: 10.1128/aem.56.9.2698-2703.1990. PubMed PMID: 16348278; PubMed Central PMCID: PMCPMC184830.

371. Biswas B, Dutta SK, Mattingly-Napier B. Gene amplification by polymerase chain reaction for detection of Ehrlichia risticii DNA in potomac horse fever. Annals of the New York Academy of Sciences. 1990;590:582-3. doi: 10.1111/j.1749-6632.1990.tb42269.x.

372. Brooker JD, Stokes B. Monoclonal antibodies against the ruminal bacterium Selenomonas ruminantium. Applied and Environmental Microbiology. 1990;56(7):2193-9. doi: 10.1128/aem.56.7.2193-2199.1990.

373. Brouqui P, Raoult D. In vitro susceptibility of Ehrlichia sennetsu to antibiotics. Antimicrobial Agents and Chemotherapy. 1990;34(8):1593-6. doi: 10.1128/AAC.34.8.1593. PubMed Central PMCID: PMCAbbott(France)

Bayer(France)

Diamant(France)

Lepetit(France)

Merck(France)

Pfizer(France).

374. Campbell SR, van Keulen H, Erlandsen SL, Senturia JB, Jarroll EL. Giardia sp.: comparison of electrophoretic karyotypes. Exp Parasitol. 1990;71(4):470-82. Epub 1990/11/01. doi: 10.1016/0014-4894(90)90073-l. PubMed PMID: 2226707.

375. Carpenter TL, McMeans MC, McHugh CP. Additional instances of human parasitism by the brown dog tick (Acari: Ixodidae). J Med Entomol. 1990;27(6):1065-6. Epub 1990/11/01. doi: 10.1093/jmedent/27.6.1065. PubMed PMID: 2280392.

376. Cotta MA. Utilization of nucleic acids by Selenomonas ruminantium and other ruminal bacteria. Applied and Environmental Microbiology. 1990;56(12):3867-70. doi: 10.1128/aem.56.12.3867-3870.1990.

377. Dawson J, Fishbein D, Eng T, Redus M, Greene N. Diagnosis of human ehrlichiosis with the indirect fluorescent antibody test: kinetics and specificity. Ann N Y Acad Sci. 1990;590:308. Epub 1990/01/01. doi: 10.1111/j.1749-6632.1990.tb42238.x. PubMed PMID: 2198831.

378. Dawson J, Fishbein D, Eng T, Redus M, Greene N. DIAGNOSIS OF HUMAN EHRLICHIOSIS WITH THE INDIRECT FLUORESCENT-ANTIBODY TEST - KINETICS AND SPECIFICITY. Annals of the New York Academy of Sciences. 1990;590:308-. doi: 10.1111/j.1749-6632.1990.tb42238.x. PubMed PMID: WOS:A1990EC75000039.

379. Dawson JE, Fishbein DB, Eng TR, Redus MA, Green NR. Diagnosis of human ehrlichiosis with the indirect fluorescent antibody test: kinetics and specificity. J Infect Dis. 1990;162(1):91-5. Epub 1990/07/01. doi: 10.1093/infdis/162.1.91. PubMed PMID: 2192013.

380. Dawson JE, Fishbein DB, Eng TR, Redus MA, Greene NR. Diagnosis of human ehrlichiosis with the indirect antibody test: Kinetics and specificity. Journal of Infectious Diseases. 1990;162(1):91-5.

381. Dawson JE, Fishbein DB, Eng TR, Redus MA, Greene NR. Diagnosis of human ehrlichiosis with the indirect fluorescent antibody test: Kinetics and specificity. Journal of Infectious Diseases. 1990;162(1):91-5. doi: 10.1093/infdis/162.1.91.

382. Dawson JE, Fishbein DB, Eng TR, Redus MA, Greene NR. DIAGNOSIS OF HUMAN EHRLICHIOSIS WITH THE INDIRECT FLUORESCENT-ANTIBODY TEST - KINETICS AND SPECIFICITY. Journal of Infectious Diseases. 1990;162(1):91-5. doi: 10.1093/infdis/162.1.91. PubMed PMID: WOS:A1990DK93700014.

383. Dutta SK, Shankarappa B, Mattingly-Napier BL. Characterization and antigenicity of recombinant major antigens of Ehrlichia risticii. Ann N Y Acad Sci. 1990;590:395-6. Epub 1990/01/01. doi: 10.1111/j.1749-6632.1990.tb42246.x. PubMed PMID: 2378467.

384. Elliott LB, Fournier PV, Teltow GJ. Rickettsia in Texas. Ann N Y Acad Sci. 1990;590:221-6. Epub 1990/01/01. doi: 10.1111/j.1749-6632.1990.tb42223.x. PubMed PMID: 2378448.

385. Ende M, O'Donnal PM, Ende FI. Ehrlichiosis in Virginia: Case reports. Virginia Medical. 1990;117(4):160-5.

386. Eng TR, Fishbein DB, Dawson JE, Greene CR, Redus M. Surveillance of human ehrlichiosis in the United States: 1988. Ann N Y Acad Sci. 1990;590:306-7. Epub 1990/01/01. doi: 10.1111/j.1749-6632.1990.tb42237.x. PubMed PMID: 2378462.

387. Eng TR, Fishbein DB, Dawson JE, Greene CR, Redus M. SURVEILLANCE OF HUMAN EHRLICHIOSIS IN THE UNITED-STATES - 1988. Annals of the New York Academy of Sciences. 1990;590:306-7. doi: 10.1111/j.1749-6632.1990.tb42237.x. PubMed PMID: WOS:A1990EC75000038.

388. Eng TR, Harkess JR, Fishbein DB, Dawson JE, Greene CN, Redus MA, et al. Epidemiologic, clinical, and laboratory findings of human ehrlichiosis in the United States, 1988. Jama. 1990;264(17):2251-8. Epub 1990/11/07. PubMed PMID: 2214103.

389. Erlandsen SL, Bemrick WJ, Wells CL, Feely DE, Knudson L, Campbell SR, et al. Axenic culture and characterization of Giardia ardeae from the great blue heron (Ardea herodias). J Parasitol. 1990;76(5):717-24. Epub 1990/10/01. PubMed PMID: 2213415.

390. Erlandsen SL, Sherlock LA, Bemrick WJ. The detection of Giardia muris and Giardia lamblia cysts by immunofluorescence in animal tissues and fecal samples subjected to cycles of freezing and thawing. Journal of Parasitology. 1990;76(2):267-71. doi: 10.2307/3283031.

391. Ewing SA, Harkess JR, Kocan KM, Barker RW, Fox JC, Tyler RD, et al. Failure to transmit Ehrlichia canis (Rickettsiales: Ehrlichieae) with Otobius megnini (Acari: Argasidae). J Med Entomol. 1990;27(5):803-6. Epub 1990/09/01. doi: 10.1093/jmedent/27.5.803. PubMed PMID: 2231616.

392. Firneisz GD, Cochrane SM, Parent J, Houston DM. Ontario. Canine ehrlichiosis in Ontario. Can Vet J. 1990;31(9):652-3. Epub 1990/09/01. PubMed PMID: 17423666; PubMed Central PMCID: PMCPMC1480908.

393. Flint HJ, Bisset J. Genetic diversity in Selenomonas ruminantium isolated from the rumen. FEMS Microbiology Ecology. 1990;73(4):351-9.

394. Ghorbel A. [Human ehrlichiosis, a new zoonosis]. Arch Inst Pasteur Tunis. 1990;67(1-2):11-24. Epub 1990/01/01. PubMed PMID: 2101572.

395. Hart CA, Baxby D. Cryptosporidium muris is particularly hazardous for people with suppressed immune systems from drugs or diseases, e.g. AIDS. J Hosp Infect. 1990;16(4):379-81. Epub 1990/11/01. doi: 10.1016/0195-6701(90)90009-d. PubMed PMID: 1980509.

396. Haukisalmi V, Henttonen H. The impact of climatic factors and host density on the long-term population dynamics of vole helminths. Oecologia. 1990;83(3):309-15. Epub 1990/06/01. doi: 10.1007/bf00317553. PubMed PMID: 28313000.

397. Kocan KM, Crawford TB, Dilbeck PM, Evermann JF, McGuire TC. Development of a rickettsia isolated from an aborted bovine fetus. Journal of Bacteriology. 1990;172(10):5949-55. doi: 10.1128/jb.172.10.5949-5955.1990.

398. Mackenstedt U, Wagner D, Heydorn AO, Mehlhorn H. DNA measurements and ploidy determination of different stages in the life cycle of Sarcocystis muris. Parasitology Research. 1990;76(8):662-8.

399. Marvin-Sikkema FD, Richardson AJ, Stewart CS, Gottschal JC, Prins RA. Influence of hydrogen-consuming bacteria on cellulose degradation by anaerobic fungi. Applied and Environmental Microbiology. 1990;56(12):3793-7. doi: 10.1128/aem.56.12.3793-3797.1990.

400. McDade JE. Ehrlichiosis--a disease of animals and humans. J Infect Dis. 1990;161(4):609-17. Epub 1990/04/01. doi: 10.1093/infdis/161.4.609. PubMed PMID: 2181027.

401. McDade JE. Ehrlichiosis - A disease of animals and humans. Journal of Infectious Diseases. 1990;161(4):609-17.

402. McDade JE. Ehrlichiosis—A disease of animals and humans. Journal of Infectious Diseases. 1990;161(4):609-17. doi: 10.1093/infdis/161.4.609.

403. Michel TA, Macy JM. Generation of a membrane potential by sodium-dependent succinate efflux in Selenomonas ruminantium. J Bacteriol. 1990;172(3):1430-5. Epub 1990/03/01. doi: 10.1128/jb.172.3.1430-1435.1990. PubMed PMID: 2307654; PubMed Central PMCID: PMCPMC208616.

404. Michel TA, Macy JM. Ferredoxin from Selenomonas ruminantium. Archives of Microbiology. 1990;153(5):518-20.

405. Michel TA, Macy JM. Preparation of spheroplasts from the strict anaerobe Selenomonas ruminantium. Journal of Microbiological Methods. 1990;11(1):37-41. doi: 10.1016/0167-7012(90)90045-8.

406. Michel TA, Macy JM. Purification of an enzyme responsible for acetate formation from acetyl coenzyme A in Selenomonas ruminantium. FEMS Microbiology Letters. 1990;68(1-2):189-94.

407. Nisbet DJ, Martin SA. Effect of Dicarboxylic Acids and Aspergillus oryzae Fermentation Extract on Lactate Uptake by the Ruminal Bacterium Selenomonas ruminantium. Appl Environ Microbiol. 1990;56(11):3515-8. Epub 1990/11/01. doi: 10.1128/aem.56.11.3515-3518.1990. PubMed PMID: 16348354; PubMed Central PMCID: PMCPMC185007.

408. Ricke SC, Schaefer DM. Characterization of Egg Yolk Antibodies for Detection and Quantification of Selenomonas ruminantium by Using an Enzyme-Linked Immunosorbent Assay. Appl Environ Microbiol. 1990;56(9):2795-800. Epub 1990/09/01. doi: 10.1128/aem.56.9.2795-2800.1990. PubMed PMID: 16348287; PubMed Central PMCID: PMCPMC184845.

409. Ricke SC, Schaefer DM. An ascorbate-induced medium for nitrogen metabolism studies with Selenomonas ruminantium. Journal of Microbiological Methods. 1990;11(3-4):219-27.

410. Rikihisa Y. Growth of Ehrlichia risticii in human colonic epithelial cells. Ann N Y Acad Sci. 1990;590:104-10. Epub 1990/01/01. doi: 10.1111/j.1749-6632.1990.tb42212.x. PubMed PMID: 2378444.

411. Rikihisa Y. GROWTH OF EHRLICHIA-RISTICII IN HUMAN COLONIC EPITHELIAL-CELLS. Annals of the New York Academy of Sciences-Series. 1990;590:104-10. doi: 10.1111/j.1749-6632.1990.tb42212.x. PubMed PMID: WOS:A1990EC75000013.

412. Rohrbach BW, Harkess JR, Ewing SA, Kudlac J, McKee GL, Istre GR. Epidemiologic and clinical characteristics of persons with serologic evidence of E. canis infection. Am J Public Health. 1990;80(4):442-5. Epub 1990/04/01. doi: 10.2105/ajph.80.4.442. PubMed PMID: 2316766; PubMed Central PMCID: PMCPMC1404562.

413. Romia SA, Abou-Zakham AA, Gamil T, el-Khouly ES. Virulence of Giardia lamblia isolates to laboratory mice. J Egypt Soc Parasitol. 1990;20(2):633-8. Epub 1990/12/01. PubMed PMID: 2230320.

414. Rommel M, Tenter AM, Vietmeyer C, Mencke N. Production and characterisation of monoclonal antibodies for species diagnosis of sarcosporidia. Rev Sci Tech. 1990;9(1):235-8. Epub 1990/03/01. doi: 10.20506/rst.9.1.476. PubMed PMID: 2132150.

415. Sarmati L, Vella S, Rocchi G. Epidemiological record on infectious diseases. Medicina - Rivista della Enciclopedia Medica Italiana. 1990;10(2):195-7.

416. Sotto A, Alvarez JL, García B, Pomar F, Cendán A. [Acute hepatic lesion caused by Giardia lamblia]. Rev Esp Enferm Dig. 1990;77(1):24-8. Epub 1990/01/01. PubMed PMID: 2334580.

417. Stockham SL, Tyler JW, Schmidt DA, Curtis KS. Experimental transmission of granulocytic ehrlichial organisms in dogs. Vet Clin Pathol. 1990;19(4):99-104. Epub 1990/01/01. doi: 10.1111/j.1939-165x.1990.tb00552.x. PubMed PMID: 12684945.

418. Thaker SR, Dutta SK, Adhya SL, Mattingly-Napier BL. Molecular cloning of Ehrlichia risticii and development of a gene probe for the diagnosis of potomac horse fever. Journal of Clinical Microbiology. 1990;28(9):1963-7. doi: 10.1128/jcm.28.9.1963-1967.1990.

419. Tilley M, Upton SJ, Blagburn BL, Anderson BC. Identification of outer oocyst wall proteins of three Cryptosporidium (apicomplexa: Cryptosporidiidae) species by 125I surface labeling. Infection and Immunity. 1990;58(1):252-3.

420. Van Gylswyk NO. Enumeration and presumptive identification of some functional groups of bacteria in the rumen of dairy cows fed grass silage-based diets. FEMS Microbiology Ecology. 1990;73(3):243-53.

421. Weiss E, Dasch GA, Kang YH, Williams JC. Comparison of properties of isolated ehrlichiae and scrub typhus rickettsiae. Ann N Y Acad Sci. 1990;590:76-84. Epub 1990/01/01. doi: 10.1111/j.1749-6632.1990.tb42210.x. PubMed PMID: 1974128.

422. Wilkins SC, Ambrosio RE. A differential lysis method for the isolation of Cowdria ruminantium DNA. Veterinary Microbiology. 1990;25(1):39-44. doi: 10.1016/0378-1135(90)90091-9.

423. Williams DK, Martin SA. Xylose uptake by the ruminal bacterium Selenomonas ruminantium. Applied and Environmental Microbiology. 1990;56(6):1683-8. doi: 10.1128/aem.56.6.1683-1688.1990.

424. First European case of ehrlichiosis. Lancet; 1991. p. 633-4.

425. Another tick-borne illness... ehrlichiosis, a rickettsial infection... reprinted from Emergency Medicine, August 15, 1989. Emergency Medicine (00136654). 1991;23(14):173-4. PubMed PMID: 107433962. Language: English. Entry Date: 19940201. Revision Date: 20150711. Publication Type: Journal Article. Journal Subset: Allied Health.

426. Abbott KC, Vukelja SJ, Smith CE, McAllister CK, Konkol KA, O'Rourke TJ, et al. Hemophagocytic syndrome: a cause of pancytopenia in human ehrlichiosis. Am J Hematol. 1991;38(3):230-4. Epub 1991/11/01. doi: 10.1002/ajh.2830380315. PubMed PMID: 1951324.

427. Adam RD. The biology of Giardia spp. Microbiol Rev. 1991;55(4):706-32. Epub 1991/12/01. doi: 10.1128/mr.55.4.706-732.1991. PubMed PMID: 1779932; PubMed Central PMCID: PMCPMC372844.

428. Anderson BE, Dawson JE, Jones DC, Wilson KH. Ehrlichia chaffeensis, a new species associated with human ehrlichiosis. J Clin Microbiol. 1991;29(12):2838-42. Epub 1991/12/01. doi: 10.1128/jcm.29.12.2838-2842.1991. PubMed PMID: 1757557; PubMed Central PMCID: PMCPMC270443.

429. Anderson BE, Dawson JE, Jones DC, Wilson KH. EHRLICHIA-CHAFFEENSIS, A NEW SPECIES ASSOCIATED WITH HUMAN EHRLICHIOSIS. Journal of Clinical Microbiology. 1991;29(12):2838-42. doi: 10.1128/jcm.29.12.2838-2842.1991. PubMed PMID: WOS:A1991GP88400030.

430. Barta JR, Jenkins MC, Danforth HD. Evolutionary relationships of avian Eimeria species among other apicomplexan protozoa: Monophyly of the apicomplexa is supported. Molecular Biology and Evolution. 1991;8(3):345-55.

431. Barton LL. Therapy of human ehrlichiosis reconsidered. Antimicrob Agents Chemother. 1991;35(2):398. Epub 1991/02/01. doi: 10.1128/aac.35.2.398. PubMed PMID: 2024976; PubMed Central PMCID: PMCPMC245017.

432. Biavati B, Mattarelli P. Bifidobacterium ruminantium sp. nov. and Bifidobacterium merycicum sp. nov. from the rumens of cattle. International Journal of Systematic Bacteriology. 1991;41(1):163-8. doi: 10.1099/00207713-41-1-163.

433. Biswas B, Mukherjee D, Mattingly-Napier BL, Dutta SK. Diagnostic application of polymerase chain reaction for detection of Ehrlichia risticii in equine monocytic ehrlichiosis (Potomac horse fever). J Clin Microbiol. 1991;29(10):2228-33. Epub 1991/10/01. doi: 10.1128/jcm.29.10.2228-2233.1991. PubMed PMID: 1939575; PubMed Central PMCID: PMCPMC270303.

434. Buchel LA, Chochillon C, Gorenflot A, Brugerolle G, Gobert JG, Savel J. GIARDIA FROM HUMANS - ANALYSIS OF INVITRO EXCYSTATION BY TRANSMISSION ELECTRON-MICROSCOPY. Comptes Rendus Des Seances De La Societe De Biologie Et De Ses Filiales. 1991;185(1-2):69-77. PubMed PMID: WOS:A1991GF36000009.

435. Caldwell CW, Poje E, Cooperstock M. Expansion of immature thymic precursor cells in peripheral blood after acute marrow suppression. Am J Clin Pathol. 1991;95(6):824-7. Epub 1991/06/01. doi: 10.1093/ajcp/95.6.824. PubMed PMID: 2042592.

436. Crouch AA, Seow WK, Whitman LM, Smith SE, Thong YH. INHIBITION OF ADHERENCE OF GIARDIA-INTESTINALIS BY HUMAN NEUTROPHILS AND MONOCYTES. Transactions of the Royal Society of Tropical Medicine and Hygiene. 1991;85(3):375-9. doi: 10.1016/0035-9203(91)90297-c. PubMed PMID: WOS:A1991FU71800015.

437. Dawson JE, Anderson BE, Fishbein DB, Sanchez JL, Goldsmith CS, Wilson KH, et al. Isolation and characterization of an Ehrlichia sp. from a patient diagnosed with human ehrlichiosis. J Clin Microbiol. 1991;29(12):2741-5. Epub 1991/12/01. doi: 10.1128/jcm.29.12.2741-2745.1991. PubMed PMID: 1757543; PubMed Central PMCID: PMCPMC270425.

438. Dawson JE, Rikihisa Y, Ewing SA, Fishbein DB. Serologic diagnosis of human ehrlichiosis using two Ehrlichia canis isolates. J Infect Dis. 1991;163(3):564-7. Epub 1991/03/01. doi: 10.1093/infdis/163.3.564. PubMed PMID: 1995730.

439. Dawson JE, Rikihisa Y, Ewing SA, Fishbein DB. SEROLOGIC DIAGNOSIS OF HUMAN EHRLICHIOSIS USING 2 EHRLICHIA-CANIS ISOLATES. Journal of Infectious Diseases. 1991;163(3):564-7. doi: 10.1093/infdis/163.3.564. PubMed PMID: WOS:A1991EY49500021.

440. Denkers EY, Hayes CE, Wassom DL. Trichinella spiralis: Influence of an immunodominant, carbohydrate-associated determinant on the host antibody response repertoire. Experimental Parasitology. 1991;72(4):403-10. doi: 10.1016/0014-4894(91)90086-C.

441. Dumler JS, Aronson JF, Walker DH. HUMAN EHRLICHIOSIS - PATHOLOGICAL FINDINGS IN 3 FATAL CASES. Laboratory Investigation. 1991;64(1):A87-A. PubMed PMID: WOS:A1991EV36600526.

442. Dumler JS, Brouqui P, Aronson J, Taylor JP, Walker DH. Identification of Ehrlichia in human tissue. N Engl J Med. 1991;325(15):1109-10. Epub 1991/10/10. doi: 10.1056/nejm199110103251517. PubMed PMID: 1891018.

443. Dumler JS, Walker DH. Ehrlichia. Jama. 1991;266(8):1082. Epub 1991/08/28. PubMed PMID: 1865539.

444. Dumler JS, Walker DH. Ehrlichia [8]. Journal of the American Medical Association. 1991;266(8):1082. doi: 10.1001/jama.266.8.1082.

445. Dumler JS, Walker DH. Human ehrlichiosis. Current Opinion in Infectious Diseases. 1991;4(5):597-602. doi: 10.1097/00001432-199110000-00002.

446. Edwards MS. Human ehrlichiosis. Adv Pediatr Infect Dis. 1991;6:163-78. Epub 1991/01/01. PubMed PMID: 2054119.

447. Ellis JE, McIntyre PS, Saleh M, Williams AG, Lloyd D. Influence of CO2 and low concentrations of O2 on fermentative metabolism of the rumen ciliate Dasytricha ruminantium. Journal of General Microbiology. 1991;137(6):1409-17. doi: 10.1099/00221287-137-6-1409.

448. Else KJ, Grencis RK. Helper T-cell subsets in mouse trichuriasis. Parasitol Today. 1991;7(11):313-6. Epub 1991/11/01. doi: 10.1016/0169-4758(91)90268-s. PubMed PMID: 15463401.

449. Entzeroth R, Konig A, Dubremetz JF. Monoclonal antibodies identify micronemes and a new population of cytoplasmic granules cross-reacting with micronemes of cystozoites of Sarcocystis muris. Parasitology Research. 1991;77(1):59-64.

450. Feely DE, Gardner MD, Hardin EL. Excystation of Giardia muris induced by a phosphate-bicarbonate medium: Localization of acid phosphatase. Journal of Parasitology. 1991;77(3):441-8. doi: 10.2307/3283133.

451. Gajadhar AA, Marquardt WC, Hall R, Gunderson J, Ariztia-Carmona EV, Sogin ML. Ribosomal RNA sequences of Sarcocystis muris, Theileria annulata and Crypthecodinium cohnii reveal evolutionary relationships among apicomplexans, dinoflagellates, and ciliates. Molecular and Biochemical Parasitology. 1991;45(1):147-54. doi: 10.1016/0166-6851(91)90036-6.

452. Gajadhar AA, Marquardt WC, Hall R, Gunderson J, Ariztiacarmona EV, Sogin ML. RIBOSOMAL-RNA SEQUENCES OF SARCOCYSTIS-MURIS, THEILERIA-ANNULATA AND CRYPTHECODINIUM-COHNII REVEAL EVOLUTIONARY RELATIONSHIPS AMONG APICOMPLEXANS, DINOFLAGELLATES, AND CILIATES. Molecular and Biochemical Parasitology. 1991;45(1):147-54. doi: 10.1016/0166-6851(91)90036-6. PubMed PMID: WOS:A1991EY72100016.

453. Ghorbel A, Kennou MF, Ben Hamed S, Ben Jemaa M, Vidor E. Human ehrlichiosis in Tunisia preliminary study. Medecine et Maladies Infectieuses. 1991;21(12):725-31.

454. Gottstein B, Nash TE. ANTIGENIC VARIATION IN GIARDIA-LAMBLIA - INFECTION OF CONGENITALLY ATHYMIC NUDE AND SCID MICE. Parasite Immunology. 1991;13(6):649-59. doi: 10.1111/j.1365-3024.1991.tb00560.x. PubMed PMID: WOS:A1991GR63200008.

455. Gottstein B, Stocks NI, Shearer GM, Nash TE. HUMAN CELLULAR IMMUNE-RESPONSE TO GIARDIA-LAMBLIA. Infection. 1991;19(6):421-6. doi: 10.1007/bf01726454. PubMed PMID: WOS:A1991GW34300009.

456. Guerrero A, Fishbein DB, Mesa E, Escudero R. [Human infection by Ehrlichia canis in Spain?]. Med Clin (Barc). 1991;96(6):236-7. Epub 1991/02/16. PubMed PMID: 2041392.

457. Harkess JR. Ehrlichiosis. Infect Dis Clin North Am. 1991;5(1):37-51. Epub 1991/03/01. PubMed PMID: 2051014.

458. Hoskins JD. Tick-borne zoonoses: Lyme disease, ehrlichiosis, and Rocky Mountain spotted fever. Semin Vet Med Surg Small Anim. 1991;6(3):236-43. Epub 1991/08/01. PubMed PMID: 1962008.

459. Jongejan F, Bax R, Meddens MJM, Quint WGV. Cowdria ruminantium is recognized by a monoclonal antibody directed against the major outer membrane protein of Chlamydia trachomatis. Veterinary Microbiology. 1991;27(2):115-23. doi: 10.1016/0378-1135(91)90002-W.

460. Kaylor PS, Crawford TB, McElwain TF, Palmer GH. PASSIVE TRANSFER OF ANTIBODY TO EHRLICHIA-RISTICII PROTECTS MICE FROM EHRLICHIOSIS. Infection and Immunity. 1991;59(6):2058-62. doi: 10.1128/iai.59.6.2058-2062.1991. PubMed PMID: WOS:A1991FN77500030.

461. Kennedy MW, Wassom DL, McIntosh AE, Thomas JC. H-2 (I-A) CONTROL OF THE ANTIBODY REPERTOIRE TO SECRETED ANTIGENS OF TRICHINELLA-SPIRALIS IN INFECTION AND ITS RELEVANCE TO RESISTANCE AND SUSCEPTIBILITY. Immunology. 1991;73(1):36-43. PubMed PMID: WOS:A1991FM92400007.

462. Klag AR, Dunbar LE, Girard CA. Concurrent ehrlichiosis and babesiosis in a dog. Can Vet J. 1991;32(5):305-7. Epub 1991/05/01. PubMed PMID: 17423787; PubMed Central PMCID: PMCPMC1481478.

463. Kontos VI, Papadopoulos O, French TW. Natural and experimental canine infections with a Greek strain of Ehrlichia platys. Vet Clin Pathol. 1991;20(4):101-5. Epub 1991/01/01. doi: 10.1111/j.1939-165x.1991.tb00867.x. PubMed PMID: 12673539.

464. Lillywhite JE, Bundy DAP, Didier JM, Cooper ES, Bianco AE. HUMORAL IMMUNE-RESPONSES IN HUMAN INFECTION WITH THE WHIPWORM TRICHURIS-TRICHIURA. Parasite Immunology. 1991;13(5):491-507. doi: 10.1111/j.1365-3024.1991.tb00546.x. PubMed PMID: WOS:A1991GG34700003.

465. Magnarelli LA, Andreadis TG, Stafford KC, 3rd, Holland CJ. Rickettsiae and Borrelia burgdorferi in ixodid ticks. J Clin Microbiol. 1991;29(12):2798-804. Epub 1991/12/01. doi: 10.1128/jcm.29.12.2798-2804.1991. PubMed PMID: 1757551; PubMed Central PMCID: PMCPMC270436.

466. Magnarelli LA, Andreadis TG, Stafford KC, Holland CJ. RICKETTSIAE AND BORRELIA-BURGDORFERI IN IXODID TICKS. Journal of Clinical Microbiology. 1991;29(12):2798-804. doi: 10.1128/jcm.29.12.2798-2804.1991. PubMed PMID: WOS:A1991GP88400023.

467. Morais JD, Dawson JE, Greene C, Filipe AR, Galhardas LC, Bacellar F. First European cases of ehrlichiosis [9]. Lancet. 1991;338(8767):633-4.

468. Moskovitz M, Fadden R, Min T. Human ehrlichiosis: a rickettsial disease associated with severe cholestasis and multisystemic disease. J Clin Gastroenterol. 1991;13(1):86-90. Epub 1991/02/01. PubMed PMID: 2007754.

469. Nichols GL, McLauchlin J, Samuel D. A technique for typing Cryptosporidium isolates. J Protozool. 1991;38(6):237s-40s. Epub 1991/11/01. PubMed PMID: 1818185.

470. Ning Z, Attwood GT, Lockington RA, Brooker JD. Genetic diversity in ruminal isolates of Selenomonas ruminantium. Current Microbiology. 1991;22(5):279-84.

471. Park J, Rikihisa Y. Inhibition of Ehrlichia risticii infection in murine peritoneal macrophages by gamma interferon, a calcium ionophore, and concanavalin A. Infection and Immunity. 1991;59(10):3418-23. PubMed Central PMCID: PMCGenzyme(United States)

Valeant(United States)

Sigma(United States).

472. Parzy D, Davoust B, Bissuel G, Vidor E. Human pathogenicity of Ehrlichia canis. Lancet. 1991;337(8750):1169. Epub 1991/05/11. doi: 10.1016/0140-6736(91)92843-q. PubMed PMID: 1674051.

473. Parzy D, Davoust B, Bissuel G, Vidor E. HUMAN PATHOGENICITY OF EHRLICHIA-CANIS. Lancet. 1991;337(8750):1169-. doi: 10.1016/0140-6736(91)92843-q. PubMed PMID: WOS:A1991FL12400055.

474. Parzy D, Davoust B, Haddad S, Vidor E, Lecamus JL. SEROPREVALENCE OF HUMAN AND CANINE EHRLICHIOSIS STUDY IN A MILITARY ENVIRONMENT IN THE NORTH TUNISIAN. Medecine Et Armees. 1991;19(4):215-9. PubMed PMID: WOS:A1991GL88800006.

475. Parzy D, Davoust B, Raphenon G, Vidor E. [Canine ehrlichiosis in Senegal: human and canine seroepidemiological survey in Dakar]. Med Trop (Mars). 1991;51(1):59-63. Epub 1991/01/01. PubMed PMID: 2072850.

476. Parzy D, Davoust B, Raphenon G, Vidor E. Canine ehrlichiosis in Senegal: Human and canine seroepidemiologic survey in Dakar. Medecine Tropicale. 1991;51(1):59-63.

477. Rajasekariah GR, Parab PB, Subrahmanyam D. Detection of Wuchereria bancrofti specific antigens in the serum of endemic residents. Trop Med Parasitol. 1991;42(2):103-5. Epub 1991/06/01. PubMed PMID: 1896764.

478. Rajasekariah GR, Parab PB, Subrahmanyam D. DETECTION OF WUCHERERIA-BANCROFTI SPECIFIC ANTIGENS IN THE SERUM OF ENDEMIC RESIDENTS. Tropical Medicine and Parasitology. 1991;42(2):103-5. PubMed PMID: WOS:A1991FV76900007.

479. Raoult D. Therapy of human ehrlichiosis reconsidered (Reply). Antimicrobial Agents and Chemotherapy. 1991;35(2):398.

480. Raoult D. THERAPY OF HUMAN EHRLICHIOSIS RECONSIDERED - REPLY. Antimicrobial Agents and Chemotherapy. 1991;35(2):398-. PubMed PMID: WOS:A1991EV74200041.

481. Rautenbach GH, Boomker J, de Villiers IL. A descriptive study of the canine population in a rural town in southern Africa. J S Afr Vet Assoc. 1991;62(4):158-62. Epub 1991/12/01. PubMed PMID: 1770490.

482. Reed GH. ERLICHIOSIS - ANOTHER HUMAN TICK-BORNE DISEASE. Journal of Environmental Health. 1991;54(1):34-5. PubMed PMID: WOS:A1991FU12300007.

483. Reed Jr GH. Erlichiosis: another human tick-borne disease. Journal of Environmental Health. 1991;54(1):34-5.

484. Reiner KL, Huycke MM, McNabb SJ. The descriptive epidemiology of Lyme disease in Oklahoma. J Okla State Med Assoc. 1991;84(10):503-9. Epub 1991/10/01. PubMed PMID: 1744781.

485. Rhee JK, Seu YS, Park BK. Isolation and identification of Cryptosporidium from various animals in Korea. III. Identification of Cryptosporidium baileyi from Korean chicken. Kisaengch'unghak chapchi The Korean journal of parasitology. 1991;29(4):315-24. doi: 10.3347/kjp.1991.29.4.315.

486. Ricke SC, Schaefer DM. Growth inhibition of the rumen bacterium Selenomonas ruminantium by ammonium salts. Applied Microbiology and Biotechnology. 1991;36(3):394-9.

487. Rikihisa Y. The tribe Ehrlichieae and ehrlichial diseases. Clin Microbiol Rev. 1991;4(3):286-308. Epub 1991/07/01. doi: 10.1128/cmr.4.3.286. PubMed PMID: 1889044; PubMed Central PMCID: PMCPMC358200.

488. Ristic M, Holland CJ, Khondowe M. An overview of research on ehrlichiosis. Eur J Epidemiol. 1991;7(3):246-52. Epub 1991/05/01. doi: 10.1007/bf00145673. PubMed PMID: 1884776.

489. Robinson M, Krco CJ, Beito TG, David CS. GENETIC-CONTROL OF THE IMMUNE-RESPONSE TO TRICHINELLA-SPIRALIS - RECOGNITION OF MUSCLE LARVAL ANTIGENS. Parasite Immunology. 1991;13(4):391-404. doi: 10.1111/j.1365-3024.1991.tb00292.x. PubMed PMID: WOS:A1991FW16700005.

490. Russell JB. Intracellular pH of acid-tolerant ruminal bacteria. Applied and Environmental Microbiology. 1991;57(11):3383-4. doi: 10.1128/aem.57.11.3383-3384.1991.

491. Sauch JF, Flanigan D, Galvin ML, Berman D, Jakubowski W. Propidium iodide as an indicator of Giardia cyst viability. Applied and Environmental Microbiology. 1991;57(11):3243-7. doi: 10.1128/aem.57.11.3243-3247.1991.

492. Schuler PF, Ghosh MM, Gopalan P. Slow sand and diatomaceous earth filtration of cysts and other particulates. Water Research. 1991;25(8):995-1005. doi: 10.1016/0043-1354(91)90149-K.

493. Strobel HJ, Russel JB. Succinate transport by a ruminal selenomonad and its regulation by carbohydrate availability and osmotic strength. Applied and Environmental Microbiology. 1991;57(1):248-54. doi: 10.1128/aem.57.1.248-254.1991.

494. Tenter AM, Johnson MR, Johnson AM. Effect of tryptic or peptic digestion or mechanical isolation on the extraction of proteins, antigens, and ribonucleic acids from Sarcocystis muris bradyzoites. Journal of Parasitology. 1991;77(2):194-9. doi: 10.2307/3283079.

495. Tenter AM, Zimmerman GL, Johnson AM. Separation of antigens from Sarcocystis species using chromatofocusing. Journal of Parasitology. 1991;77(5):727-36. doi: 10.2307/3282706.

496. Van Keulen H, Campbell SR, Erlandsen SL, Jarroll EL. Cloning and restriction enzyme mapping of ribosomal DNA of Giardia duodenalis, Giardia ardeae and Giardia muris. Molecular and Biochemical Parasitology. 1991;46(2):275-84. doi: 10.1016/0166-6851(91)90051-7.

497. Walker DH, Fishbein DB. Epidemiology of rickettsial diseases. Eur J Epidemiol. 1991;7(3):237-45. Epub 1991/05/01. doi: 10.1007/bf00145672. PubMed PMID: 1884775.

498. Weisburg WG, Barns SM, Pelletier DA, Lane DJ. 16S ribosomal DNA amplification for phylogenetic study. J Bacteriol. 1991;173(2):697-703. Epub 1991/01/01. doi: 10.1128/jb.173.2.697-703.1991. PubMed PMID: 1987160; PubMed Central PMCID: PMCPMC207061.

499. Weiss E. Biology of ehrlichiae. Eur J Epidemiol. 1991;7(3):253-8. Epub 1991/05/01. doi: 10.1007/bf00145674. PubMed PMID: 1884777.

500. Williams AG, Withers SE, Joblin KN. Xylanolysis by cocultures of the rumen fungus Neocallimastix frontalis and ruminal bacteria. Letters in Applied Microbiology. 1991;12(6):232-5.

501. Zellner G, Geveke M, Conway de Macario E, Diekmann H. Population dynamics of biofilm development during start-up of a butyrate-degrading fluidized-bed reactor. Applied Microbiology and Biotechnology. 1991;36(3):404-9.

502. Anderson B. Etiologic agent of human ehrlichiosis. Pediatr Infect Dis J. 1992;11(7):597-8. Epub 1992/07/01. doi: 10.1097/00006454-199207000-00021. PubMed PMID: 1528656.

503. Anderson B. Etiologic agent of human ehrlichiosis [1]. Pediatric Infectious Disease Journal. 1992;11(7):597-8.

504. Anderson BE, Greene CE, Jones DC, Dawson JE. Ehrlichia ewingii sp. nov., the etiologic agent of canine granulocytic ehrlichiosis. Int J Syst Bacteriol. 1992;42(2):299-302. Epub 1992/04/01. doi: 10.1099/00207713-42-2-299. PubMed PMID: 1581189.

505. Anderson BE, Greene CE, Jones DC, Dawson JE. EHRLICHIA-EWINGII SP-NOV, THE ETIOLOGIC AGENT OF CANINE GRANULOCYTIC EHRLICHIOSIS. International Journal of Systematic Bacteriology. 1992;42(2):299-302. doi: 10.1099/00207713-42-2-299. PubMed PMID: WOS:A1992HN40500016.

506. Anderson BE, Sumner JW, Dawson JE, Tzianabos T, Greene CR, Olson JG, et al. Detection of the etiologic agent of human ehrlichiosis by polymerase chain reaction. Journal of Clinical Microbiology. 1992;30(4):775-80. doi: 10.1128/jcm.30.4.775-780.1992.

507. Anderson BE, Sumner JW, Dawson JE, Tzianabos T, Greene CR, Olson JG, et al. Detection of the etiologic agent of human ehrlichiosis by polymerase chain reaction. J Clin Microbiol. 1992;30(4):775-80. Epub 1992/04/01. doi: 10.1128/jcm.30.4.775-780.1992. PubMed PMID: 1374076; PubMed Central PMCID: PMCPMC265160.

508. Anderson BE, Sumner JW, Dawson JE, Tzianabos T, Greene CR, Olson JG, et al. DETECTION OF THE ETIOLOGIC AGENT OF HUMAN EHRLICHIOSIS BY POLYMERASE CHAIN-REACTION. Journal of Clinical Microbiology. 1992;30(4):775-80. doi: 10.1128/jcm.30.4.775-780.1992. PubMed PMID: WOS:A1992HJ48700004.

509. Attwood GT, Brooker JD. Complete nucleotide sequence of a Selenomonas ruminantium plasmid and definition of a region necessary for its replication in Escherichia coli. Plasmid. 1992;28(2):123-9. Epub 1992/09/01. doi: 10.1016/0147-619x(92)90043-a. PubMed PMID: 1409969.

510. Barker IK, Surgeoner GA, Artsob H, McEwen SA, Elliott LA, Campbell GD, et al. DISTRIBUTION OF THE LYME-DISEASE VECTOR, IXODES-DAMMINI (ACARI, IXODIDAE) AND ISOLATION OF BORRELIA-BURGDORFERI IN ONTARIO, CANADA. Journal of Medical Entomology. 1992;29(6):1011-22. doi: 10.1093/jmedent/29.6.1011. PubMed PMID: WOS:A1992JW62300019.

511. Barton LL, Rathore MH, Dawson JE. Infection with Ehrlichia in childhood. J Pediatr. 1992;120(6):998-1001. Epub 1992/06/01. doi: 10.1016/s0022-3476(05)81978-x. PubMed PMID: 1593364.

512. Brouqui P, Dumler JS, Raoult D, Walker DH. Antigenic characterization of ehrlichiae: protein immunoblotting of Ehrlichia canis, Ehrlichia sennetsu, and Ehrlichia risticii. J Clin Microbiol. 1992;30(5):1062-6. Epub 1992/05/01. doi: 10.1128/jcm.30.5.1062-1066.1992. PubMed PMID: 1583101; PubMed Central PMCID: PMCPMC265224.

513. Brouqui P, Dumler JS, Raoult D, Walker DH. ANTIGENIC CHARACTERIZATION OF EHRLICHIAE - PROTEIN IMMUNOBLOTTING OF EHRLICHIA-CANIS, EHRLICHIA-SENNETSU, AND EHRLICHIA-RISTICII. Journal of Clinical Microbiology. 1992;30(5):1062-6. doi: 10.1128/jcm.30.5.1062-1066.1992. PubMed PMID: WOS:A1992HP82800003.

514. Brouqui P, Raoult D. In vitro antibiotic susceptibility of the newly recognized agent of ehrlichiosis in humans, Ehrlichia chaffeensis. Antimicrob Agents Chemother. 1992;36(12):2799-803. Epub 1992/12/01. doi: 10.1128/aac.36.12.2799. PubMed PMID: 1482148; PubMed Central PMCID: PMCPMC245548.

515. Brouqui P, Raoult D. INVITRO ANTIBIOTIC SUSCEPTIBILITY OF THE NEWLY RECOGNIZED AGENT OF EHRLICHIOSIS IN HUMANS, EHRLICHIA-CHAFFEENSIS. Antimicrobial Agents and Chemotherapy. 1992;36(12):2799-803. doi: 10.1128/aac.36.12.2799. PubMed PMID: WOS:A1992KA93400039.

516. Cai J, Collins MD, McDonald V, Thompson DE. PCR cloning and nucleotide sequence determination of the 18S rRNA genes and internal transcribed spacer 1 of the protozoan parasites Cryptosporidium parvum and Cryptosporidium muris. Biochimica et Biophysica Acta - Gene Structure and Expression. 1992;1131(3):317-20. doi: 10.1016/0167-4781(92)90032-U.

517. Corwin RM. CRYPTOSPORIDIOSIS - A COCCIDIOSIS OF CALVES. Compendium on Continuing Education for the Practicing Veterinarian. 1992;14(7):1005-7. PubMed PMID: WOS:A1992JE08600014.

518. Cotta MA. Interaction of ruminal bacteria in the production and utilization of maltooligosaccharides from starch. Applied and Environmental Microbiology. 1992;58(1):48-54. doi: 10.1128/aem.58.1.48-54.1992.

519. Dame JB, Mahan SM, Yowell CA. Phylogenetic relationship of Cowdria ruminantium, agent of heartwater, to Anaplasma marginale and other members of the order Rickettsiales determined on the basis of 16S rRNA sequence. Int J Syst Bacteriol. 1992;42(2):270-4. Epub 1992/04/01. doi: 10.1099/00207713-42-2-270. PubMed PMID: 1581187.

520. Dawson JE, Ewing SA. Susceptibility of dogs to infection with Ehrlichia chaffeensis, causative agent of human ehrlichiosis. American journal of veterinary research. 1992;53(8):1322-7.

521. Dawson JE, Ewing SA. SUSCEPTIBILITY OF DOGS TO INFECTION WITH EHRLICHIA-CHAFFEENSIS, CAUSATIVE AGENT OF HUMAN EHRLICHIOSIS. American Journal of Veterinary Research. 1992;53(8):1322-7. PubMed PMID: WOS:A1992JF10000010.

522. Deng MY, Cliver DO. DEGRADATION OF GIARDIA-LAMBLIA CYSTS IN MIXED HUMAN AND SWINE WASTES. Applied and Environmental Microbiology. 1992;58(8):2368-74. doi: 10.1128/aem.58.8.2368-2374.1992. PubMed PMID: WOS:A1992JF83900004.

523. Dumler JS, Walker DH. HEMATOPATHOLOGY OF HUMAN EHRLICHIOSIS - BONE-MARROW FINDINGS IN SEROLOGICALLY CONFIRMED CASES. Laboratory Investigation. 1992;66(1):A92-A. PubMed PMID: WOS:A1992HA27600552.

524. Dunn BE, Monson TP, Dumler JS, Morris CC, Westbrook AB, Duncan JL, et al. Identification of Ehrlichia chaffeensis morulae in cerebrospinal fluid mononuclear cells. J Clin Microbiol. 1992;30(8):2207-10. Epub 1992/08/01. doi: 10.1128/jcm.30.8.2207-2210.1992. PubMed PMID: 1500537; PubMed Central PMCID: PMCPMC265474.

525. Eschenbacher KH, Sommer I, Meyer HE, Mehlhorn H, Ruger W. Cloning and expression in Escherichia coli of cDNAs encoding a 31-kilodalton surface antigen of Sarcocystis muris. Molecular and Biochemical Parasitology. 1992;53(1-2):159-68. doi: 10.1016/0166-6851(92)90018-F.

526. Fraker LD, Gentile DA, Krivoy D, Condon M, Backer HD. Giardia cyst inactivation by iodine. Journal of Wilderness Medicine. 1992;3(4):351-7. doi: 10.1580/0953-9859-3.4.351.

527. Goldman DP, Artenstein AW, Bolan CD. Human ehrlichiosis: a newly recognized tick-borne disease. Am Fam Physician. 1992;46(1):199-208. Epub 1992/07/01. PubMed PMID: 1621632.

528. Goldman DP, Artenstein AW, Bolan CD. HUMAN EHRLICHIOSIS - A NEWLY RECOGNIZED TICK-BORNE DISEASE. American Family Physician. 1992;46(1):199-208. PubMed PMID: WOS:A1992JD74300019.

529. Heinrichová K, Dzúrová M, Rexová-Benková L. Mechanism of action of D-galacturonan digalacturonohydrolase of Selenomonas ruminantium on oligogalactosiduronic acids. Carbohydr Res. 1992;235:269-80. Epub 1992/11/04. doi: 10.1016/0008-6215(92)80095-i. PubMed PMID: 1473108.

530. Kardatzke JT, Neidhardt K, Dzuban DP, Sanchez JL, Azad AF. Cluster of tick-borne infections at Fort Chaffee, Arkansas: Rickettsiae and Borrelia burgdorferi in ixodid ticks. J Med Entomol. 1992;29(4):669-72. Epub 1992/07/01. doi: 10.1093/jmedent/29.4.669. PubMed PMID: 1495077.

531. Marrie TJ, Raoult D. RICKETTSIAL INFECTIONS OF THE CENTRAL-NERVOUS-SYSTEM. Seminars in Neurology. 1992;12(3):213-24. doi: 10.1055/s-2008-1041178. PubMed PMID: WOS:A1992JT72100009.

532. Matte A, Forsberg CW, Verrinder Gibbins AM. Erratum: Enzymes associated with metabolism of xylose and other pentoses by Prevotella (Bacteroides) ruminicola strains, Selenomonas ruminantium D, and Fibrobacter succinogenes S85 (Ref. Can. J. Microbiol. 38: 370-376). Canadian Journal of Microbiology. 1992;38(9):1003. doi: 10.1139/m92-164.

533. Matte A, Forsberg CW, Verrinder Gibbins AM. Enzymes associated with metabolism of xylose and other pentoses by Prevotella (Bacteroides) ruminicola strains, Selenomas ruminantium D, and Fibrobacter succinogenes S85. Canadian Journal of Microbiology. 1992;38(5):370-6. doi: 10.1139/m92-063.

534. Nina JMS, McDonald V, Dyson DA, Catchpole J, Uni S, Iseki M, et al. Analysis of oocyst wall and sporozoite antigens from three Cryptosporidium species. Infection and Immunity. 1992;60(4):1509-13. doi: 10.1128/iai.60.4.1509-1513.1992.

535. Petro TM, Watson RR, Feely DE, Darban H. Suppression of resistance to Giardia muris and cytokine production in a murine model of acquired immune deficiency syndrome. Regional Immunology. 1992;4(6):409-14.

536. Randhawa VS, Sharma VK, Baveja UK, Vij JC, Malhotra V. HUMAN GIARDIASIS - CORRELATION OF SPECIFIC SECRETORY IGA LEVELS IN DUODENAL FLUID TO THE SEVERITY OF DISEASE AND INFESTATION BY GIARDIA-LAMBLIA. Zentralblatt Fur Bakteriologie-International Journal of Medical Microbiology Virology Parasitology and Infectious Diseases. 1992;277(1):106-11. PubMed PMID: WOS:A1992JC79600015.

537. Rathore MH. Infection due to Ehrlichia canis in children. South Med J. 1992;85(7):703-5. Epub 1992/07/01. doi: 10.1097/00007611-199207000-00008. PubMed PMID: 1631682.

538. Sadler LA, McNally KL, Govind NS, Brunk CF, Trench RK. The nucleotide sequence of the small subunit ribosomal RNA gene from Symbiodinium pilosum, a symbiotic dinoflagellate. Current Genetics. 1992;21(4-5):409-16.

539. Sanchez JL, Candler WH, Fishbein DB, Greene CR, Coté TR, Kelly DJ, et al. A cluster of tick-borne infections: association with military training and asymptomatic infections due to Rickettsia rickettsii. Trans R Soc Trop Med Hyg. 1992;86(3):321-5. Epub 1992/05/01. doi: 10.1016/0035-9203(92)90330-f. PubMed PMID: 1412666.

540. Sanchez JL, Candler WH, Fishbein DB, Greene CR, Cote TR, Kelly DJ, et al. A cluster of tick-borne infections: Association with military training and asymptomatic infections due to Rickettsia rickettsii. Transactions of the Royal Society of Tropical Medicine and Hygiene. 1992;86(3):321-5. doi: 10.1016/0035-9203(92)90330-F.

541. Shankarappa B, Dutta SK, Mattingly-Napier BL. Antigenic and genomic relatedness among Ehrlichia risticii, Ehrlichia sennetsu, and Ehrlichia canis. Int J Syst Bacteriol. 1992;42(1):127-32. Epub 1992/01/01. doi: 10.1099/00207713-42-1-127. PubMed PMID: 1736961.

542. Sommer I, Horn K, Heydorn AO, Mehlhorn H, Ruger W. A comparison of sporozoite and cyst merozoite surface proteins of Sarcocystis. Parasitology Research. 1992;78(5):398-403. PubMed Central PMCID: PMCBiorad(United States)

Sigma(United States).

543. Taylor E, Sinnott 4th JT. Ehrlichiosis. Infection control and hospital epidemiology : the official journal of the Society of Hospital Epidemiologists of America. 1992;13(2):104-6.

544. Taylor E, Sinnott JT. EHRLICHIOSIS. Infection Control and Hospital Epidemiology. 1992;13(2):104-6. PubMed PMID: WOS:A1992HE03400008.

545. Taylor E, Sinnott JTt. Ehrlichiosis. Infect Control Hosp Epidemiol. 1992;13(2):104-6. Epub 1992/02/11. doi: 10.1086/646481. PubMed PMID: 1541801.

546. Uhaa IJ, MacLean JD, Greene CR, Fishbein DB. A case of human ehrlichiosis acquired in Mali: clinical and laboratory findings. Am J Trop Med Hyg. 1992;46(2):161-4. Epub 1992/02/01. doi: 10.4269/ajtmh.1992.46.161. PubMed PMID: 1539750.

547. Van Keulen H, Gutell RR, Campbell SR, Erlandsen SL, Jarroll EL. The nucleotide sequence of the entire ribosomal DNA operon and the structure of the large subunit rRNA of Giardia muris. Journal of Molecular Evolution. 1992;35(4):318-28. doi: 10.1007/BF00161169.

548. van Kuppeveld FJ, van der Logt JT, Angulo AF, van Zoest MJ, Quint WG, Niesters HG, et al. Genus- and species-specific identification of mycoplasmas by 16S rRNA amplification. Appl Environ Microbiol. 1992;58(8):2606-15. Epub 1992/08/01. doi: 10.1128/aem.58.8.2606-2615.1992. PubMed PMID: 1381174; PubMed Central PMCID: PMCPMC195828.

549. Van Kuppeveld FJM, Van der Logt JTM, Angulo AF, Van Zoest MJ, Quint WGV, Niesters HGM, et al. Genus- and species-specific identification of mycoplasmas by 16S rRNA amplification. Applied and Environmental Microbiology. 1992;58(8):2606-15. doi: 10.1128/aem.58.8.2606-2615.1992.

550. van Vliet AH, Jongejan F, van der Zeijst BA. Phylogenetic position of Cowdria ruminantium (Rickettsiales) determined by analysis of amplified 16S ribosomal DNA sequences. Int J Syst Bacteriol. 1992;42(3):494-8. Epub 1992/07/01. doi: 10.1099/00207713-42-3-494. PubMed PMID: 1380292.

551. Van Vliet AHM, Jongejan F, Van der Zeijst BAM. Phylogenetic position of Cowdria ruminantium (rickettsiales) determined by analysis of amplified 16S ribosomal DNA sequences. International Journal of Systematic Bacteriology. 1992;42(3):494-8. doi: 10.1099/00207713-42-3-494.

552. Vankeulen H, Gutell RR, Campbell SR, Erlandsen SL, Jarroll EL. THE NUCLEOTIDE-SEQUENCE OF THE ENTIRE RIBOSOMAL DNA OPERON AND THE STRUCTURE OF THE LARGE SUBUNIT RIBOSOMAL-RNA OF GIARDIA-MURIS. Journal of Molecular Evolution. 1992;35(4):318-28. doi: 10.1007/bf00161169. PubMed PMID: WOS:A1992JN42000007.

553. Vanvliet AHM, Jongejan F, Vanderzeijst BAM. PHYLOGENETIC POSITION OF COWDRIA-RUMINANTIUM (RICKETTSIALES) DETERMINED BY ANALYSIS OF AMPLIFIED 16S RIBOSOMAL DNA-SEQUENCES. International Journal of Systematic Bacteriology. 1992;42(3):494-8. doi: 10.1099/00207713-42-3-494. PubMed PMID: WOS:A1992JE08300024.

554. Abbaszadegan M, Gerba CP, Yahya MT, Rusin P. Evaluation of a microbiological water purifier for inactivation of viruses, bacteria and Giardia cysts. Water Science and Technology. 1993;27(3-4):329-33. doi: 10.2166/wst.1993.0369.

555. Anderson BE, Sims KG, Olson JG, Childs JE, Piesman JF, Happ CM, et al. Amblyomma americanum: a potential vector of human ehrlichiosis. Am J Trop Med Hyg. 1993;49(2):239-44. Epub 1993/08/01. doi: 10.4269/ajtmh.1993.49.239. PubMed PMID: 8357086.

556. Anderson BE, Sims KG, Olson JG, Childs JE, Piesman JF, Happ CM, et al. Amblyomma americanum: A potential vector of human ehrlichiosis. American Journal of Tropical Medicine and Hygiene. 1993;49(2):239-44. doi: 10.4269/ajtmh.1993.49.239.

557. Anderson BE, Sims KG, Olson JG, Childs JE, Piesman JF, Happ CM, et al. AMBLYOMMA-AMERICANUM - A POTENTIAL VECTOR OF HUMAN EHRLICHIOSIS. American Journal of Tropical Medicine and Hygiene. 1993;49(2):239-44. doi: 10.4269/ajtmh.1993.49.239. PubMed PMID: WOS:A1993LW87100014.

558. Cotta MA. Utilization of xylooligosaccharides by selected ruminal bacteria. Applied and Environmental Microbiology. 1993;59(11):3557-63. doi: 10.1128/aem.59.11.3557-3563.1993.

559. Dawson JE, Candal FJ, George VG, Ades EW. Human endothelial cells as an alternative to DH82 cells for isolation of Ehrlichia chaffeensis, E. canis, and Rickettsia rickettsii. Pathobiology. 1993;61(5-6):293-6. Epub 1993/01/01. doi: 10.1159/000163808. PubMed PMID: 8297496.

560. Dawson JE, Candal FJ, George VG, Ades EW. HUMAN ENDOTHELIAL-CELLS AS AN ALTERNATIVE TO DH82 CELLS FOR ISOLATION OF EHRLICHIA-CHAFFEENSIS, EHRLICHIA-CANIS, AND RICKETTSIA-RICKETTSII. Pathobiology. 1993;61(5-6):293-6. doi: 10.1159/000163808. PubMed PMID: WOS:A1993MN87700007.

561. Dewhirst FE, Paster BJ, Olsen I, Fraser GJ. Phylogeny of the Pasteurellaceae as determined by comparison of 16S ribosomal ribonucleic acid sequences. Zentralbl Bakteriol. 1993;279(1):35-44. Epub 1993/06/01. doi: 10.1016/s0934-8840(11)80489-5. PubMed PMID: 7690271.

562. Dumler JS, Dawson JE, Walker DH. Human ehrlichiosis: hematopathology and immunohistologic detection of Ehrlichia chaffeensis. Hum Pathol. 1993;24(4):391-6. Epub 1993/04/01. doi: 10.1016/0046-8177(93)90087-w. PubMed PMID: 8491479.

563. Dumler JS, Dawson JE, Walker DH. HUMAN EHRLICHIOSIS - HEMATOPATHOLOGY AND IMMUNOHISTOLOGIC DETECTION OF EHRLICHIA-CHAFFEENSIS. Human Pathology. 1993;24(4):391-6. PubMed PMID: WOS:A1993KX35400009.

564. Dumler JS, Sutker WL, Walker DH. Persistent infection with Ehrlichia chaffeensis. Clin Infect Dis. 1993;17(5):903-5. Epub 1993/11/01. doi: 10.1093/clinids/17.5.903. PubMed PMID: 8286638.

565. Eschenbacher KH, Klein H, Sommer I, Meyer HE, Entzeroth R, Mehlhorn H, et al. Characterization of cDNA clones encoding a major microneme antigen of Sarcocystis muris (Apicomplexa) cyst merozoites. Molecular and Biochemical Parasitology. 1993;62(1):27-36. doi: 10.1016/0166-6851(93)90174-V.

566. Ey PL, Andrews RH, Mayrhofer G. Differentiation of major genotypes of Giardia intestinalis by polymerase chain reaction analysis of a gene encoding a trophozoite surface antigen. Parasitology. 1993;106 ( Pt 4):347-56. Epub 1993/05/01. doi: 10.1017/s0031182000067081. PubMed PMID: 8316431.

567. Ey PL, Andrews RH, Mayrhofer G. DIFFERENTIATION OF MAJOR GENOTYPES OF GIARDIA-INTESTINALIS BY POLYMERASE CHAIN-REACTION ANALYSIS OF A GENE ENCODING A TROPHOZOITE SURFACE-ANTIGEN. Parasitology. 1993;106:347-56. doi: 10.1017/s0031182000067081. PubMed PMID: WOS:A1993LA63600002.

568. Gavaghan PD, Sykora JL, Jakubowski W, Sorber CA, Sninsky AM, Lichte MD, et al. Inactivation of Giardia by anaerobic digestion of sludge. Water Science and Technology. 1993;27(3-4):111-4. doi: 10.2166/wst.1993.0331.

569. Guerrero A. [Human ehrlichiosis]. Enferm Infecc Microbiol Clin. 1993;11(5):271-6. Epub 1993/05/01. PubMed PMID: 8324026.

570. Hayashi S. Effects of immune rabbit serum and monoclonal antibodies on infectivity of Cryptosporidium muris in mice. Journal of the Osaka City Medical Center. 1993;42(2):165-87.

571. Krumholz LR, Bryant MP, Brulla WJ, Vicini JL, Clark JH, Stahl DA. Proposal of Quinella ovalis gen. nov., sp. nov., based on phylogenetic analysis. International Journal of Systematic Bacteriology. 1993;43(2):293-6. doi: 10.1099/00207713-43-2-293.

572. Lukin EP, Vorob'ev AA. [Ehrlichia and ehrlichiosis (leukocyte Rickettsia and rickettsiosis)]. Zh Mikrobiol Epidemiol Immunobiol. 1993;(3):95-9. Epub 1993/05/01. PubMed PMID: 8067101.

573. Majewska AC, Kasprzak W, Kaczmarek E. COMPARATIVE MORPHOMETRY OF GIARDIA TROPHOZOITES FROM MAN AND ANIMALS. Acta Protozoologica. 1993;32(3):191-7. PubMed PMID: WOS:A1993LT08000010.

574. Nisbet DJ, Martin SA. Effects of fumarate, L-malate, and an Aspergillus oryzae fermentation extract on D-lactate utilization by the ruminal bacterium Selenomonas ruminantium. Current Microbiology. 1993;26(3):133-6.

575. Ogunkolade BW, Robinson HA, McDonald V, Webster K, Evans DA. Isoenzyme variation within the genus Cryptosporidium. Parasitol Res. 1993;79(5):385-8. Epub 1993/01/01. doi: 10.1007/bf00931827. PubMed PMID: 8415544.

576. Paddock CD, Suchard DP, Grumbach KL, Hadley WK, Kerschmann RL, Abbey NW, et al. Brief report: fatal seronegative ehrlichiosis in a patient with HIV infection. New England Journal of Medicine. 1993;329(16):1164-7. PubMed PMID: 105851792. Language: English. Entry Date: 20080314. Revision Date: 20150711. Publication Type: Journal Article.

577. Paddock CD, Suchard DP, Grumbach KL, Hadley WK, Kerschmann RL, Abbey NW, et al. Brief report: fatal seronegative ehrlichiosis in a patient with HIV infection. N Engl J Med. 1993;329(16):1164-7. Epub 1993/10/14. doi: 10.1056/nejm199310143291605. PubMed PMID: 8377780.

578. Paddock CD, Suchard DP, Grumbach KL, Hadley WK, Kerschmann RL, Abbey NW, et al. Fatal Seronegative Ehrlichiosis in a Patient with HIV Infection. New England Journal of Medicine. 1993;329(16):1164-7. doi: 10.1056/NEJM199310143291605.

579. Paddock CD, Suchard DP, Grumbach KL, Hadley WK, Kerschmann RL, Abbey NW, et al. FATAL SERONEGATIVE EHRLICHIOSIS IN A PATIENT WITH HIV-INFECTION. New England Journal of Medicine. 1993;329(16):1164-7. doi: 10.1056/nejm199310143291605. PubMed PMID: WOS:A1993MA66700005.

580. Paget TA, Manning P, Jarroll EL. Oxygen uptake in cysts and trophozoites of Giardia lamblia. J Eukaryot Microbiol. 1993;40(3):246-50. Epub 1993/05/01. doi: 10.1111/j.1550-7408.1993.tb04911.x. PubMed PMID: 8508163.

581. Rasmussen MA. Isolation and characterization of Selenomonas ruminantium strains capable of 2-deoxyribose utilization. Applied and Environmental Microbiology. 1993;59(7):2077-81. doi: 10.1128/aem.59.7.2077-2081.1993.

582. Rathore MH. Human ehrlichiosis: 'Spotless' Rocky Mountain spotted fever. Infections in Medicine. 1993;10(5):21-2+5.

583. Rathore MH, Barton LL, Dawson JE, Regnery RL, Ayoub EM. Ehrlichia chaffeensis and Rochalimaea antibodies in Kawasaki disease. J Clin Microbiol. 1993;31(11):3058-9. Epub 1993/11/01. doi: 10.1128/jcm.31.11.3058-3059.1993. PubMed PMID: 8263201; PubMed Central PMCID: PMCPMC266216.

584. Reagan WJ, editor A review of myelofibrosis in dogs. Toxicologic Pathology; 1993.

585. Reagan WJ. A REVIEW OF MYELOFIBROSIS IN DOGS. Toxicologic Pathology. 1993;21(2):164-9. doi: 10.1177/019262339302100208. PubMed PMID: WOS:A1993LR95300007.

586. Reed GH. LYME-DISEASE AND OTHER TICK-BORNE DISEASES - A REVIEW. Journal of Environmental Health. 1993;55(8):6-10. PubMed PMID: WOS:A1993LF78000002.

587. Reed Jr GH. Lyme disease and other tick-borne diseases: A review. Journal of Environmental Health. 1993;55(8):6-10.

588. Roberts-Thomson IC. Genetic studies of human and murine giardiasis. Clin Infect Dis. 1993;16 Suppl 2:S98-104. Epub 1993/03/01. doi: 10.1093/clinids/16.supplement_2.s98. PubMed PMID: 8452966.

589. Robertsthomson IC. GENETIC-STUDIES OF HUMAN AND MURINE GIARDIASIS. Clinical Infectious Diseases. 1993;16:S98-S104. doi: 10.1093/clinids/16.Supplement_2.S98. PubMed PMID: WOS:A1993KN47700006.

590. Sanford JP. Human ehrlichiosis: A therapeutic and diagnostic dilemma in children. Infections in Medicine. 1993;10(5):18+20.

591. Schiff BB, Lamon M. Unilateral contraction of facial muscles do effect emotions: a "failed replication's" failure to perform a replication. Cortex. 1993;29(3):549-51; discussion 53-4. Epub 1993/09/01. doi: 10.1016/s0010-9452(13)80260-2. PubMed PMID: 8258292.

592. Schiff BB, Lamon M, Kop WJ, Merckelbach H, Muris P. Unilateral contraction of facial muscles do effect emotions: A 'failed replication's' failure to perform a replication. Cortex. 1993;29(3):549-51+53-54.

593. Silberg SL, Bisonni R, Parker DE, Lawrence CH, Hunt L. Human ehrlichiosis--an overview. J Okla State Med Assoc. 1993;86(3):124-7. Epub 1993/03/01. PubMed PMID: 8445459.

594. Slaff M, Newton NH. LOCATION OF TICK (ACARI, IXODIDAE) ATTACHMENT SITES ON HUMANS IN NORTH-CAROLINA. Journal of Medical Entomology. 1993;30(2):485-8. doi: 10.1093/jmedent/30.2.485. PubMed PMID: WOS:A1993KP40200029.

595. Spach DH, Liles WC, Campbell GL, Quick RE, Anderson DE, Fritsche TR. TICK-BORNE DISEASES IN THE UNITED-STATES. New England Journal of Medicine. 1993;329(13):936-47. doi: 10.1056/nejm199309233291308. PubMed PMID: WOS:A1993LX75100008.

596. Stephen Dumler J, Dawson JE, Walker DH. Human ehrlichiosis: Hematopathology and immunohistologic detection of Ehrlichia chaffeensis. Human Pathology. 1993;24(4):391-6. doi: 10.1016/0046-8177(93)90087-W.

597. Strobel HJ. Evidence for catabolite inhibition in regulation of pentose utilization and transport in the ruminal bacterium Selenomonas ruminantium. Applied and Environmental Microbiology. 1993;59(1):40-6. doi: 10.1128/aem.59.1.40-46.1993.

598. Sumner JW, Sims KG, Jones DC, Anderson BE. Ehrlichia chaffeensis expresses an immunoreactive protein homologous to the Escherichia coli GroEL protein. Infect Immun. 1993;61(8):3536-9. Epub 1993/08/01. doi: 10.1128/iai.61.8.3536-3539.1993. PubMed PMID: 8101510; PubMed Central PMCID: PMCPMC281035.

599. Sumner JW, Sims KG, Jones DC, Anderson BE. EHRLICHIA-CHAFFEENSIS EXPRESSES AN IMMUNOREACTIVE PROTEIN HOMOLOGOUS TO THE ESCHERICHIA-COLI GROEL PROTEIN. Infection and Immunity. 1993;61(8):3536-9. doi: 10.1128/iai.61.8.3536-3539.1993. PubMed PMID: WOS:A1993LP35700060.

600. Taylor DM, Neal DL, Farquhar CF. Elimination of Giardia muris from Syrian Hamsters. Contemp Top Lab Anim Sci. 1993;33(6):13-4. Epub 1993/11/01. PubMed PMID: 16468698.

601. Totté P, Blankaert D, Marique T, Kirkpatrick C, Van Vooren JP, Wérenne J. Bovine and human endothelial cell growth on collagen microspheres and their infection with the rickettsia Cowdria ruminantium: prospects for cells and vaccine production. Rev Elev Med Vet Pays Trop. 1993;46(1-2):153-6. Epub 1993/01/01. PubMed PMID: 8134625.

602. Totte P, Blankaert D, Marique T, Kirkpatrick C, Vanvooren JP, Werenne J, editors. BOVINE AND HUMAN ENDOTHELIAL-CELL GROWTH ON COLLAGEN MICROSPHERES AND THEIR INFECTION WITH THE RICKETTSIA COWDRIA-RUMINANTIUM - PROSPECTS FOR CELLS AND VACCINE PRODUCTION. 2nd Biennial Meeting of the Society-for-Tropical-Veterinary-Medicine; 1993 Feb 02-06; St Francois, Guadeloupe1993.

603. Totté P, Blankaert D, Zilimwabagabo P, Wérenne J. Inhibition of Cowdria ruminantium infectious yield by interferons alpha and gamma in endothelial cells. Revue d'élevage et de médecine vétérinaire des pays tropicaux. 1993;46(1-2):189-94.

604. Van Keulen H, Gutell RR, Gates MA, Campbell SR, Erlandsen SL, Jarroll EL, et al. Unique phylogenetic position of Diplomonadida based on the complete small subunit ribosomal RNA sequence of Giardia ardeae, G. muris, G. duodenalis and Hexamita sp. FASEB Journal. 1993;7(1):223-31. doi: 10.1096/fasebj.7.1.8422968.

605. Van Kleef M, Neitz AW, De Waal DT. Isolation and characterization of antigenic proteins of Cowdria ruminantium. Rev Elev Med Vet Pays Trop. 1993;46(1-2):157-64. Epub 1993/01/01. PubMed PMID: 8134626.

606. Van Praagh AD, Gavaghan PD, Sykora JL. Giardia muris cyst inactivation in anaerobic digester sludge. Water Science and Technology. 1993;27(3-4):105-9. doi: 10.2166/wst.1993.0330.

607. Vanat I, Pristas P, Rybosoval E, Godany A, Javorsky P. SruI restriction endonuclease from Selenomonas ruminantium. FEMS Microbiology Letters. 1993;113(2):129-32. doi: 10.1016/0378-1097(93)90257-3.

608. Vanheeckeren AM, Rikihisa Y, Park J, Fertel R. TUMOR-NECROSIS-FACTOR-ALPHA, INTERLEUKIN-1-ALPHA, INTERLEUKIN-6, AND PROSTAGLANDIN-E2 PRODUCTION IN MURINE PERITONEAL-MACROPHAGES INFECTED WITH EHRLICHIA-RISTICII. Infection and Immunity. 1993;61(10):4333-7. doi: 10.1128/iai.61.10.4333-4337.1993. PubMed PMID: WOS:A1993LZ26400044.

609. Viscogliosi E, Brugerolle G. Cytoskeleton in trichomonads: II. Immunological and biochemical characterization of the preaxostylar fibres and undulating membrane in the genus Tritrichomonas. Eur J Protistol. 1993;29(4):381-9. Epub 1993/11/12. doi: 10.1016/s0932-4739(11)80400-1. PubMed PMID: 23195736.

610. Viscogliosi E, Brugerolle G. Cytoskeleton in trichomonads: I. Immunological and biochemical comparative study of costal proteins in the genus Tritrichomonas. Eur J Protistol. 1993;29(2):160-70. Epub 1993/05/28. doi: 10.1016/s0932-4739(11)80269-5. PubMed PMID: 23195538.

611. Yu X, Brouqui P, Dumler JS, Raoult D. Detection of Ehrlichia chaffeensis in human tissue by using a species-specific monoclonal antibody. J Clin Microbiol. 1993;31(12):3284-8. Epub 1993/12/01. doi: 10.1128/jcm.31.12.3284-3288.1993. PubMed PMID: 7508458; PubMed Central PMCID: PMCPMC266402.

612. Yu XJ, Brouqui P, Dumler JS, Raoult D. DETECTION OF EHRLICHIA-CHAFFEENSIS IN HUMAN TISSUE BY USING A SPECIES-SPECIFIC MONOCLONAL-ANTIBODY. Journal of Clinical Microbiology. 1993;31(12):3284-8. doi: 10.1128/jcm.31.12.3284-3288.1993. PubMed PMID: WOS:A1993MG72500033.

613. Yunker CE, Mahan SM, Waghela SD, McGuire TC, Rurangirwa FR, Barbet AF, et al. Detection of Cowdria ruminantium by means of a DNA probe, pCS20 in infected bont ticks, Amblyomma hebraeum, the major vector of heartwater in southern Africa. Epidemiology & Infection. 1993;110(1):95-104. doi: 10.1017/S095026880005072X. PubMed PMID: 104787855. Language: English. Entry Date: 20110610. Revision Date: 20200708. Publication Type: journal article.

614. Zhang N, Brooker JD. Characterization, sequence, and replication of a small cryptic plasmid from Selenomonas ruminantium subspecies lactilytica. Plasmid. 1993;29(2):125-34. Epub 1993/03/01. doi: 10.1006/plas.1993.1014. PubMed PMID: 8469719.

615. Granulocytic ehrlichiosis: is it in your future? Emergency Medicine (00136654). 1994;26(13):37-. PubMed PMID: 107392329. Language: English. Entry Date: 19961201. Revision Date: 20150711. Publication Type: Journal Article. Journal Subset: Allied Health.

616. Arraga-Alvarado C. [Human ehrlichiosis. Review]. Invest Clin. 1994;35(4):209-22. Epub 1994/12/01. PubMed PMID: 7734523.

617. Arraya-Alvarado C. Human ehrlichiosis. Review. Investigacion Clinica. 1994;35(4):209-22.

618. Ashour AA, Wannas MQ, el-Alfy NM, Salama MM, Arafa MA, Abdel Mawla MM, et al. Scaning electron microscopy of Capillaria muris sylvatici from Clethrionomys glareolus with reference to the zoonotic species. J Egypt Soc Parasitol. 1994;24(3):663-70. Epub 1994/12/01. PubMed PMID: 7844433.

619. Awad-El-Kariem FM, McDonald V. Detection and Species Identification of Cryptosporidium Oocysts using a System Based on PCR and Endonuclease Restriction. Parasitology. 1994;109(1):19-22. doi: 10.1017/S0031182000077714.

620. Awad-el-Kariem FM, Warhurst DC, McDonald V. Detection and species identification of Cryptosporidium oocysts using a system based on PCR and endonuclease restriction. Parasitology. 1994;109 ( Pt 1):19-22. Epub 1994/07/01. doi: 10.1017/s0031182000077714. PubMed PMID: 8058364.

621. Awadelkariem FM, Warhurst DC, McDonald V. DETECTION AND SPECIES IDENTIFICATION OF CRYPTOSPORIDIUM OOCYSTS USING A SYSTEM BASED ON PCR AND ENDONUCLEASE RESTRICTION. Parasitology. 1994;109:19-22. doi: 10.1017/s0031182000077714. PubMed PMID: WOS:A1994NX90200003.

622. Bakken JS, Dumler JS, Chen SM, Eckman MR, Van Etta LL, Walker DH. Human granulocytic ehrlichiosis in the upper Midwest United States. A new species emerging? Jama. 1994;272(3):212-8. Epub 1994/07/20. PubMed PMID: 8022040.

623. Bakken JS, Dumler JS, Chen SM, Eckman MR, Vanetta LL, Walker DH. HUMAN GRANULOCYTIC EHRLICHIOSIS IN THE UPPER MIDWEST UNITED-STATES - A NEW SPECIES EMERGING. Jama-Journal of the American Medical Association. 1994;272(3):212-8. doi: 10.1001/jama.272.3.212. PubMed PMID: WOS:A1994NW18500022.

624. Barnewall RE, Rikihisa Y. Abrogation of gamma interferon-induced inhibition of Ehrlichia chaffeensis infection in human monocytes with iron-transferrin. Infect Immun. 1994;62(11):4804-10. Epub 1994/11/01. doi: 10.1128/iai.62.11.4804-4810.1994. PubMed PMID: 7927758; PubMed Central PMCID: PMCPMC303190.

625. Barnewall RE, Rikihisa Y. Abrogation of gamma interferon-induced inhibition of Ehrlichia chaffeensis infection in human monocytes with iron transferrin. Infection and Immunity. 1994;62(11):4804-10. PubMed Central PMCID: PMCGenzyme(United States)

Sigma.

626. Barnewall RE, Rikihisa Y. ABROGATION OF GAMMA-INTERFERON-INDUCED INHIBITION OF EHRLICHIA-CHAFFEENSIS INFECTION IN HUMAN MONOCYTES WITH IRON TRANSFERRIN. Infection and Immunity. 1994;62(11):4804-10. doi: 10.1128/iai.62.11.4804-4810.1994. PubMed PMID: WOS:A1994PN30400015.

627. Boopathy R. Transformation of nitroaromatic compounds by a methanogenic bacterium, Methanococcus sp. (strain B). Archives of Microbiology. 1994;162(3):167-72. doi: 10.1007/s002030050120.

628. Brouqui P, Birg ML, Raoult D. Cytopathic effect, plaque formation, and lysis of Ehrlichia chaffeensis grown on continuous cell lines. Infect Immun. 1994;62(2):405-11. Epub 1994/02/01. doi: 10.1128/iai.62.2.405-411.1994. PubMed PMID: 8300201; PubMed Central PMCID: PMCPMC186122.

629. Brouqui P, Birg ML, Raoult D. CYTOPATHIC EFFECT, PLAQUE-FORMATION, AND LYSIS OF EHRLICHIA-CHAFFEENSIS GROWN ON CONTINUOUS CELL-LINES. Infection and Immunity. 1994;62(2):405-11. doi: 10.1128/iai.62.2.405-411.1994. PubMed PMID: WOS:A1994MR84100011.

630. Brouqui P, Le Cam C, Kelly PJ, Laurens R, Tounkara A, Sawadogo S, et al. Serologic evidence for human ehrlichiosis in Africa. European Journal of Epidemiology. 1994;10(6):695-8. doi: 10.1007/BF01719283.

631. Brouqui P, Le Cam C, Kelly PJ, Laurens R, Tounkara A, Sawadogo S, et al. Serologic evidence for human ehrlichiosis in Africa. European Journal of Epidemiology. 1994;10(6):695-8. doi: 10.1007/BF01719283.

632. Brouqui P, Le Cam C, Kelly PJ, Laurens R, Tounkara A, Sawadogo S, et al. Serologic evidence for human ehrlichiosis in Africa. Eur J Epidemiol. 1994;10(6):695-8. Epub 1994/12/01. doi: 10.1007/bf01719283. PubMed PMID: 7672049.

633. Brouqui P, Lecam C, Kelly PJ, Laurens R, Tounkara A, Sawadogo S, et al. SEROLOGIC EVIDENCE FOR HUMAN EHRLICHIOSIS IN AFRICA. European Journal of Epidemiology. 1994;10(6):695-8. doi: 10.1007/bf01719283. PubMed PMID: WOS:A1994RE21500007.

634. Brouqui P, Lecam C, Olson J, Raoult D. Serologic diagnosis of human monocytic ehrlichiosis by immunoblot analysis. Clin Diagn Lab Immunol. 1994;1(6):645-9. Epub 1994/11/01. doi: 10.1128/cdli.1.6.645-649.1994. PubMed PMID: 8556515; PubMed Central PMCID: PMCPMC368382.

635. Brouqui P, Raoult D. Human ehrlichiosis. N Engl J Med. 1994;330(24):1760-1. Epub 1994/06/16. doi: 10.1056/nejm199406163302418. PubMed PMID: 8043106.

636. Brouqui P, Raoult D, Rynkiewicz D, Liu LX. Human ehrlichiosis [7]. New England Journal of Medicine. 1994;330(24):1760-1. doi: 10.1056/NEJM199406163302417.

637. Brouqui P, Raoult D, Rynkiewicz D, Liu LX. Human ehrlichiosis. New England Journal of Medicine. 1994;330(24):1760-1. doi: 10.1056/NEJM199406163302418.

638. Chen SM, Dumler JS, Bakken JS, Walker DH. Identification of a granulocytotropic Ehrlichia species as the etiologic agent of human disease. J Clin Microbiol. 1994;32(3):589-95. Epub 1994/03/01. doi: 10.1128/jcm.32.3.589-595.1994. PubMed PMID: 8195363; PubMed Central PMCID: PMCPMC263091.

639. Chen SM, Dumler JS, Bakken JS, Walker DH. IDENTIFICATION OF A GRANULOCYTOTROPIC EHRLICHIA SPECIES AS THE ETIOLOGIC AGENT OF HUMAN-DISEASE. Journal of Clinical Microbiology. 1994;32(3):589-95. doi: 10.1128/jcm.32.3.589-595.1994. PubMed PMID: WOS:A1994MW51600002.

640. Chen SM, Dumler JS, Feng HM, Walker DH. Identification of the antigenic constituents of Ehrlichia chaffeensis. Am J Trop Med Hyg. 1994;50(1):52-8. Epub 1994/01/01. PubMed PMID: 8304572.

641. Chen SM, Dumler JS, Feng HM, Walker DH. IDENTIFICATION OF THE ANTIGENIC CONSTITUENTS OF EHRLICHIA-CHAFFEENSIS. American Journal of Tropical Medicine and Hygiene. 1994;50(1):52-8. doi: 10.4269/ajtmh.1994.50.1.TM0500010052. PubMed PMID: WOS:A1994MW29600008.

642. Cook GM, Russell JB. Alternative strategies of 2-deoxyglucose resistance and low affinity glucose transport in the ruminal bacteria, Streptococcus bovis and Selenomonas ruminantium. FEMS Microbiology Letters. 1994;123(1-2):207-12.

643. Cotta MA, Wheeler MB, Whitehead TR. Cyclic AMP in ruminal and other anaerobic bacteria. FEMS Microbiology Letters. 1994;124(3):355-9. doi: 10.1016/0378-1097(94)00454-4.

644. Dawson JE, Childs JE, Biggie KL, Moore C, Stallknecht D, Shaddock J, et al. WHITE-TAILED DEER AS A POTENTIAL RESERVOIR OF EHRLICHIA SPP. Journal of Wildlife Diseases. 1994;30(2):162-8. doi: 10.7589/0090-3558-30.2.162. PubMed PMID: WOS:A1994NF99400004.

645. Dawson JE, Stallknecht DE, Howerth EW, Warner C, Biggie K, Davidson WR, et al. Susceptibility of white-tailed deer (Odocoileus virginianus) to infection with Ehrlichia chaffeensis, the etiologic agent of human ehrlichiosis. J Clin Microbiol. 1994;32(11):2725-8. Epub 1994/11/01. doi: 10.1128/jcm.32.11.2725-2728.1994. PubMed PMID: 7852563; PubMed Central PMCID: PMCPMC264150.

646. Dawson JE, Stallknecht DE, Howerth EW, Warner C, Biggie K, Davidson WR, et al. SUSCEPTIBILITY OF WHITE-TAILED DEER (ODOCOILEUS-VIRGINIANUS) TO INFECTION WITH EHRLICHIA-CHAFFEENSIS, THE ETIOLOGIC AGENT OF HUMAN EHRLICHIOSIS. Journal of Clinical Microbiology. 1994;32(11):2725-8. doi: 10.1128/jcm.32.11.2725-2728.1994. PubMed PMID: WOS:A1994PM49500017.

647. Drake L, Korchev Y, Bashford L, Djamgoz M, Wakelin D, Ashall F, et al. The major secreted product of the whipworm, Trichuris, is a pore-forming protein. Proc Biol Sci. 1994;257(1350):255-61. Epub 1994/09/22. doi: 10.1098/rspb.1994.0123. PubMed PMID: 7991635.

648. Drancourt M, Raoult D. Taxonomic position of the rickettsiae: current knowledge. FEMS Microbiol Rev. 1994;13(1):13-24. Epub 1994/01/01. doi: 10.1111/j.1574-6976.1994.tb00032.x. PubMed PMID: 8117465.

649. Dumler JS, Bakken JS, Eckman MR, Vanetta LL, Chen SM, Walker DH. HUMAN GRANULOCYTIC EHRLICHIOSIS - A NEW, POTENTIALLY FATAL TICK-BORNE INFECTION DIAGNOSED BY PERIPHERAL-BLOOD SMEAR AND PCR. Laboratory Investigation. 1994;70(1):A126-A. PubMed PMID: WOS:A1994MW42600745.

650. Ellis JE, Lindmark DG, Williams AG, Lloyd D. Polypeptides of hydrogenosome-enriched fractions from rumen ciliate protozoa and trichomonads: Immunological studies. FEMS Microbiology Letters. 1994;117(2):211-6. doi: 10.1016/0378-1097(94)90197-X.

651. Evans GS, Chwalinski S, Owen G, Booth C, Singh A, Potten CS. Expression of pokeweed lectin binding in murine intestinal Paneth cells. Epithelial Cell Biol. 1994;3(1):7-15. Epub 1994/01/01. PubMed PMID: 7514935.

652. Everett ED, Evans KA, Henry RB, McDonald G. Human ehrlichiosis in adults after tick exposure. Diagnosis using polymerase chain reaction. Ann Intern Med. 1994;120(9):730-5. Epub 1994/05/01. doi: 10.7326/0003-4819-120-9-199405010-00002. PubMed PMID: 8147545.

653. Fishbein DB, Dawson JE, Robinson LE. Human ehrlichiosis in the United States, 1985 to 1990. Ann Intern Med. 1994;120(9):736-43. Epub 1994/05/01. doi: 10.7326/0003-4819-120-9-199405010-00003. PubMed PMID: 8147546.

654. Gilmour M, Flint HJ, Mitchell WJ. Multiple lactate dehydrogenase activities of the rumen bacterium Selenomonas ruminantium. Microbiology. 1994;140(8):2077-84. doi: 10.1099/13500872-140-8-2077.

655. Heyworth MF, Vergara JA. Giardia muris trophozoite antigenic targets for mouse intestinal IgA antibody. Journal of Infectious Diseases. 1994;169(2):395-8.

656. Heyworth MF, Vergara JA. GIARDIA-MURIS TROPHOZOITE ANTIGENIC TARGETS FOR MOUSE INTESTINAL IGA ANTIBODY. Journal of Infectious Diseases. 1994;169(2):395-8. doi: 10.1093/infdis/169.2.395. PubMed PMID: WOS:A1994MV82900023.

657. Jantausch BA. Lyme disease, Rocky Mountain spotted fever, ehrlichiosis: emerging and established challenges for the clinician. Ann Allergy. 1994;73(1):4-11; quiz -2. Epub 1994/07/01. PubMed PMID: 8030801.

658. Jantausch BA. LYME-DISEASE, ROCKY-MOUNTAIN-SPOTTED-FEVER, EHRLICHIOSIS - EMERGING AND ESTABLISHED CHALLENGES FOR THE CLINICIAN. Annals of Allergy. 1994;73(1):4-11. PubMed PMID: WOS:A1994NX60100002.

659. Jenkins D, Wakelin D. Immunoepidemiology of intestinal helminth infections 4. Immunopathology in trichuriasis: Lessons from the mouse model. Transactions of the Royal Society of Tropical Medicine and Hygiene. 1994;88(3):269-73. doi: 10.1016/0035-9203(94)90074-4.

660. Jenkins D, Wakelin D. 4. Immunopathology in trichuriasis: lessons from the mouse model. Transactions of the Royal Society of Tropical Medicine and Hygiene. 1994;88(3):269-73. doi: 10.1016/0035-9203(94)90074-4.

661. Jenkins D, Wakelin D. IMMUNOEPIDEMIOLOGY OF INTESTINAL HELMINTHIC INFECTIONS .4. IMMUNOPATHOLOGY IN TRICHURIASIS - LESSONS FROM THE MOUSE MODEL. Transactions of the Royal Society of Tropical Medicine and Hygiene. 1994;88(3):269-73. doi: 10.1016/0035-9203(94)90074-4. PubMed PMID: WOS:A1994NX71100005.

662. Johnson CL. Human ehrlichiosis: A brief review. Clinical Laboratory Science. 1994;7(1):20-2.

663. Lechner S, Eschenbacher KH, Entzeroth R, Mehlhorn H, Ruger W. cDNA clones of Sarcocystis muris (Apicomplexa) encoding a repetitive, arginine-rich region of a putative microneme antigen. Parasitology Research. 1994;80(4):352-4.

664. Middleton DB. Tick-borne infections. Postgrad Med. 1994;95(5):131-9. Epub 1994/05/01. doi: 10.1080/00325481.1994.11945835. PubMed PMID: 29219644.

665. Middleton DB. Tick-borne infections. What starts as a tiny bite may have a serious outcome. Postgrad Med. 1994;95(5):131-9. Epub 1994/04/01. PubMed PMID: 8153039.

666. Middleton DB. TICK-BORNE INFECTIONS - WHAT STARTS AS A TINY BITE MAY HAVE A SERIOUS OUTCOME. Postgraduate Medicine. 1994;95(5):131-&. doi: 10.1080/00325481.1994.11945835. PubMed PMID: WOS:A1994NE77000016.

667. Munderloh UG, Liu Y, Wang M, Chen C, Kurtti TJ. Establishment, maintenance and description of cell lines from the tick Ixodes scapularis. Journal of Parasitology. 1994;80(4):533-43. doi: 10.2307/3283188.

668. Munderloh UG, Liu Y, Wang MM, Chen CS, Kurtti TJ. ESTABLISHMENT, MAINTENANCE AND DESCRIPTION OF CELL-LINES FROM THE TICK IXODES-SCAPULARIS. Journal of Parasitology. 1994;80(4):533-43. doi: 10.2307/3283188. PubMed PMID: WOS:A1994PE28400007.

669. Myers SA, Sexton DJ. Dermatologic manifestations of arthropod-borne diseases. Infect Dis Clin North Am. 1994;8(3):689-712. Epub 1994/09/01. PubMed PMID: 7814841.

670. Nageswaran C, Craig PS, Devaney E. COPROANTIGEN DETECTION IN RATS EXPERIMENTALLY INFECTED WITH STRONGYLOIDES-RATTI. Parasitology. 1994;108:335-42. doi: 10.1017/s0031182000076174. PubMed PMID: WOS:A1994NF07100011.

671. Ortega-Barria E, Ward HD, Keusch GT, Pereira MEA. Growth inhibition of the intestinal parasite Giardia lamblia by a dietary lectin is associated with arrest of the cell cycle. Journal of Clinical Investigation. 1994;94(6):2283-8. doi: 10.1172/JCI117591.

672. Ortegabarria E, Ward HD, Keusch GT, Pereira MEA. GROWTH-INHIBITION OF THE INTESTINAL PARASITE GIARDIA-LAMBLIA BY A DIETARY LECTIN IS ASSOCIATED WITH ARREST OF THE CELL-CYCLE. Journal of Clinical Investigation. 1994;94(6):2283-8. doi: 10.1172/jci117591. PubMed PMID: WOS:A1994PV81000017.

673. Oxberry ME, Thompson RCA, Reynoldson JA. Evaluation of the effects of albendazole and metronidazole on the ultrastructure of Giardia duodenalis, Trichomonas vaginalis and Spironucleus muris using transmission electron microscopy. International Journal for Parasitology. 1994;24(5):695-703. doi: 10.1016/0020-7519(94)90123-6.

674. Palmer GH, Eid G, Barbet AF, McGuire TC, McElwain TF. The immunoprotective Anaplasma marginale major surface protein 2 is encoded by a polymorphic multigene family. Infect Immun. 1994;62(9):3808-16. Epub 1994/09/01. doi: 10.1128/iai.62.9.3808-3816.1994. PubMed PMID: 8063397; PubMed Central PMCID: PMCPMC303035.

675. Powers EM, Hernandez C, Boutros SN, Harper BG. Biocidal efficacy of a flocculating emergency water purification tablet. Appl Environ Microbiol. 1994;60(7):2316-23. Epub 1994/07/01. doi: 10.1128/aem.60.7.2316-2323.1994. PubMed PMID: 16349318; PubMed Central PMCID: PMCPMC201649.

676. Pristas P, Vanat I, Godany A, Javorsky P. Restriction endonucleases from Selenomonas ruminantium which recognize and cleave 5'-AT/TAAT-3'. Archives of Microbiology. 1994;161(5):439-41. doi: 10.1007/s002030050079.

677. Rikihisa Y, Ewing SA, Fox JC. Western immunoblot analysis of Ehrlichia chaffeensis, E. canis, or E. ewingii infections in dogs and humans. J Clin Microbiol. 1994;32(9):2107-12. Epub 1994/09/01. doi: 10.1128/jcm.32.9.2107-2112.1994. PubMed PMID: 7814533; PubMed Central PMCID: PMCPMC263951.

678. Rikihisa Y, Ewing SA, Fox JC. WESTERN IMMUNOBLOT ANALYSIS OF EHRLICHIA-CHAFFEENSIS, EHRLICHIA-CANIS, OR E-EWINGII INFECTIONS IN DOGS AND HUMANS. Journal of Clinical Microbiology. 1994;32(9):2107-12. doi: 10.1128/jcm.32.9.2107-2112.1994. PubMed PMID: WOS:A1994PB54100014.

679. Rikihisa Y, Zhang Y, Park J. Inhibition of infection of macrophages with Ehrlichia risticii by cytochalasins, monodansylcadaverine, and taxol. Infection and Immunity. 1994;62(11):5126-32.

680. Rikihisa Y, Zhang YL, Park J. INHIBITION OF INFECTION OF MACROPHAGES WITH EHRLICHIA-RISTICII BY CYTOCHALASINS, MONODANSYLCADAVERINE, AND TAXOL. Infection and Immunity. 1994;62(11):5126-32. doi: 10.1128/iai.62.11.5126-5132.1994. PubMed PMID: WOS:A1994PN30400059.

681. Rynkiewicz D, Liu LX. HUMAN EHRLICHIOSIS - REPLY. New England Journal of Medicine. 1994;330(24):1761-. PubMed PMID: WOS:A1994NR10900030.

682. Rynkiewicz DL, Liu LX. Human ehrlichiosis in New England. N Engl J Med. 1994;330(4):292-3. Epub 1994/01/27. doi: 10.1056/nejm199401273300418. PubMed PMID: 8272102.

683. Rynkiewicz DL, Liu LX. HUMAN EHRLICHIOSIS IN NEW-ENGLAND. New England Journal of Medicine. 1994;330(4):292-3. doi: 10.1056/nejm199401273300418. PubMed PMID: WOS:A1994MR49600027.

684. Saz JV, Dawson JE, Bacellar F, Merino FJ, Filipe AR. [Human ehrlichiosis in Spain]. Enferm Infecc Microbiol Clin. 1994;12(7):357-8. Epub 1994/08/01. PubMed PMID: 7948119.

685. Segura Porta F. [Human ehrlichiosis. Epidemiologic considerations]. Enferm Infecc Microbiol Clin. 1994;12(7):323-4. Epub 1994/08/01. PubMed PMID: 7948110.

686. Stephen Dumler J. Serodiagnosis of human ehrlichioses. Clinical Immunology Newsletter. 1994;14(5):65-8. doi: 10.1016/0197-1859(94)90037-X.

687. Totte P, Jongejan F, Degee ALW, Werenne J. PRODUCTION OF ALPHA-INTERFERON IN COWDRIA RUMINANTIUM-INFECTED CATTLE AND ITS EFFECT ON INFECTED ENDOTHELIAL-CELL CULTURES. Infection and Immunity. 1994;62(6):2600-4. PubMed PMID: WOS:A1994NM78200061.

688. Upcroft JA, Healey A, Upcroft P. A NEW RDNA REPEAT UNIT IN HUMAN GIARDIA. Journal of Eukaryotic Microbiology. 1994;41(6):639-42. doi: 10.1111/j.1550-7408.1994.tb01526.x. PubMed PMID: WOS:A1994QD42500015.

689. Van Vliet AHM, Jongejan F, Van Kleef M, Van der Zeijst BAM. Molecular cloning, sequence analysis, and expression of the gene encoding the immunodominant 32-kilodalton protein of Cowdria ruminantium. Infection and Immunity. 1994;62(4):1451-6.

690. Viale AM. The chaperone connection to the origins of the eukaryotic organelles. FEBS Letters. 1994;341(2-3):146-51. doi: 10.1016/0014-5793(94)80446-X.

691. Viale AM, Arakaki AK. The chaperone connection to the origins of the eukaryotic organelles. FEBS Lett. 1994;341(2-3):146-51. Epub 1994/03/21. doi: 10.1016/0014-5793(94)80446-x. PubMed PMID: 7907991.

692. Wallace RJ, Arthaud L, Newbold CJ. Influence of Yucca shidigera extract on ruminal ammonia concentrations and ruminal microorganisms. Applied and Environmental Microbiology. 1994;60(6):1762-7. doi: 10.1128/aem.60.6.1762-1767.1994.

693. Webster JP. The effect of Toxoplasma gondii and other parasites on activity levels in wild and hybrid Rattus norvegicus. Parasitology. 1994;109 ( Pt 5):583-9. Epub 1994/12/01. doi: 10.1017/s0031182000076460. PubMed PMID: 7831094.

694. Weiss N, Biavati B. Murein types in Bifidobacterium ruminantium, Bifidobacterium merycicum and Bifidobacterium saeculare. New Microbiol. 1994;17(2):159-62. Epub 1994/04/01. PubMed PMID: 8065274.

695. Wikel SK, Ramachandra RN, Bergman DK. TICK-INDUCED MODULATION OF THE HOST IMMUNE-RESPONSE. International Journal for Parasitology. 1994;24(1):59-66. doi: 10.1016/0020-7519(94)90059-0. PubMed PMID: WOS:A1994NG78300007.

696. Wilson KH. Detection of culture-resistant bacterial pathogens by amplification and sequencing of ribosomal DNA. Clin Infect Dis. 1994;18(6):958-62. Epub 1994/06/01. doi: 10.1093/clinids/18.6.958. PubMed PMID: 7522060.

697. Zeiss CJ, Jardine J, Huchzermeyer H. A case of disseminated tuberculosis in a dog caused by Mycobacterium avium-intracellulare. Journal of the American Animal Hospital Association. 1994;30(5):419-24. PubMed Central PMCID: PMCCentaur(South Africa).

698. Human granulocytic ehrlichiosis--New York, 1995. MMWR Morb Mortal Wkly Rep. 1995;44(32):593-5. Epub 1995/08/18. PubMed PMID: 7662028.

699. From the Centers for Disease Control and Prevention. Human granulocytic ehrlichiosis--New York, 1995. Jama. 1995;274(11):867. Epub 1995/09/20. PubMed PMID: 7674488.

700. Erratum: Human granulocytic ehrlichiosis - New York, 1995 (Journal of the American Medical Association (1995) 274 (867)). JAMA. 1995;274(12):937. doi: 10.1001/jama.274.12.937.

701. Human Granulocytic Ehrlichiosis—New York, 1995. JAMA: The Journal of the American Medical Association. 1995;274(11):867. doi: 10.1001/jama.1995.03530110023009.

702. Getting a head start against ehrlichiosis. Emergency Medicine (00136654). 1995;27(5):16-23. PubMed PMID: 107392262. Language: English. Entry Date: 19961201. Revision Date: 20150711. Publication Type: Journal Article.

703. Ehrlichiosis in Belgium. Lancet; 1995. p. 1233-4.

704. Transmission of human granulocytic ehrlichiosis. American Medical Association; 1995. p. 23-.

705. Antony SJ, Dummer JS, Hunter E. Human ehrlichiosis in a liver transplant recipient. Transplantation. 1995;60(8):879-81. Epub 1995/10/27. PubMed PMID: 7482752.

706. Bacellar F, Dawson JE, Silveira CA, Filipe AR. Antibodies against rickettsiaceae in dogs of Setubal, Portugal. Central European Journal of Public Health. 1995;3(2):100-2.

707. Bakken JS, Dumler JS. TRANSMISSION OF HUMAN GRANULOCYTIC EHRLICHIOSIS - REPLY. Jama-Journal of the American Medical Association. 1995;273(1):23-. PubMed PMID: WOS:A1995PZ04200013.

708. Bakken JS, Stephen Dumler J. Transmission of Human Granulocytic Ehrlichiosis-Reply. JAMA: The Journal of the American Medical Association. 1995;273(1):23. doi: 10.1001/jama.1995.03520250037020.

709. Barbet AF. Recent developments in the molecular biology of anaplasmosis. Veterinary Parasitology. 1995;57(1-3):43-9. doi: 10.1016/0304-4017(94)03108-9.

710. Barlough JE, Madigan JE, DeRock E, Dumler JS, Bakken JS. Protection against Ehrlichia equi is conferred by prior infection with the human granulocytotropic Ehrlichia (HGE agent). J Clin Microbiol. 1995;33(12):3333-4. Epub 1995/12/01. doi: 10.1128/jcm.33.12.3333-3334.1995. PubMed PMID: 8586731; PubMed Central PMCID: PMCPMC228702.

711. Barlough JE, Madigan JE, Derock E, Dumler JS, Bakken JS. PROTECTION AGAINST EHRLICHIA-EQUI IS CONFERRED BY PRIOR INFECTION WITH THE HUMAN GRANULOCYTOTROPIC EHRLICHIA (HGE AGENT). Journal of Clinical Microbiology. 1995;33(12):3333-4. doi: 10.1128/jcm.33.12.3333-3334.1995. PubMed PMID: WOS:A1995TF01500055.

712. Breitschwerdt EB, Kordick DL, Malarkey DE, Keene B, Hadfield TL, Wilson K. ENDOCARDITIS IN A DOG DUE TO INFECTION WITH A NOVEL BARTONELLA SUBSPECIES. Journal of Clinical Microbiology. 1995;33(1):154-60. doi: 10.1128/jcm.33.1.154-160.1995. PubMed PMID: WOS:A1995PX47200032.

713. Brouqui P, Dumler JS, Lienhard R, Brossard M, Raoult D. Human granulocytic ehrlichiosis in Europe. Lancet. 1995;346(8977):782-3. Epub 1995/09/16. doi: 10.1016/s0140-6736(95)91544-3. PubMed PMID: 7658904.

714. Brouqui P, Dumler JS, Lienhard R, Brossard M, Raoult D. Human granulocytic ehrlichiosis in Europe [20]. Lancet. 1995;346(8977):782-3.

715. Brouqui P, Raoult D. Human ehrlichiosis with features of toxic shock syndrome. Am J Med. 1995;99(1):107. Epub 1995/07/01. doi: 10.1016/s0002-9343(99)80120-x. PubMed PMID: 7598131.

716. Brouqui P, Raoult D, Durand JM. Ehrlichia species as possible causative agents of blood culture-negative endocarditis. Clin Microbiol Infect. 1995;1(2):148-50. Epub 1995/02/01. doi: 10.1111/j.1469-0691.1995.tb00462.x. PubMed PMID: 11866746.

717. Brouqui P, Raoult D, Fichtenbaum CJ, Weil GJ. Human ehrlichiosis with features of toxic shock syndrome [4]. American Journal of Medicine. 1995;99(1):107.

718. Bryan CS. Ehrlichiosis: more tick-borne terrorism? J S C Med Assoc. 1995;91(5):241-2. Epub 1995/05/01. PubMed PMID: 7776628.

719. Caldwell CW, Everett ED, McDonald G, Yesus YW, Roland WE. Lymphocytosis of gamma/delta T cells in human ehrlichiosis. Am J Clin Pathol. 1995;103(6):761-6. Epub 1995/06/01. doi: 10.1093/ajcp/103.6.761. PubMed PMID: 7785663.

720. Caldwell CW, Everett ED, McDonald G, Yesus YW, Roland WE. Lymphocytosis of γ/δ T cells in human ehrlichiosis. American Journal of Clinical Pathology. 1995;103(6):761-6. doi: 10.1093/ajcp/103.6.761.

721. Caldwell CW, Everett ED, McDonald G, Yesus YW, Roland WE. LYMPHOCYTOSIS OF GAMMA/DELTA T-CELLS IN HUMAN EHRLICHIOSIS. American Journal of Clinical Pathology. 1995;103(6):761-6. PubMed PMID: WOS:A1995RC24100018.

722. Caporale DA, Rich SM, Spielman A, Telford 3rd SR, Kocher TD. Discriminating between Ixodes ticks by means of mitochondrial DNA sequences. Molecular phylogenetics and evolution. 1995;4(4):361-5.

723. Caporale DA, Rich SM, Spielman A, Telford SR, 3rd, Kocher TD. Discriminating between Ixodes ticks by means of mitochondrial DNA sequences. Mol Phylogenet Evol. 1995;4(4):361-5. Epub 1995/12/01. doi: 10.1006/mpev.1995.1033. PubMed PMID: 8747292.

724. Cesbron-Delauw MF. The SAG2 antigen of Toxoplasma gondii and the 31-kDa surface antigen of Sarcocystis muris share similar sequence features. Parasitology Research. 1995;81(5):444-5.

725. Chen SM, Popov VL, Feng HM, Wen J, Walker DH. Cultivation of Ehrlichia chaffeensis in mouse embryo, vero, BGM, and L929 cells and study of Ehrlichia-induced cytopathic effect and plaque formation. Infection and Immunity. 1995;63(2):647-55. doi: 10.1128/iai.63.2.647-655.1995.

726. Chen SM, Popov VL, Feng HM, Wen J, Walker DH. CULTIVATION OF EHRLICHIA-CHAFFEENSIS IN MOUSE EMBRYO, VERO, BGM, AND L929 CELLS AND STUDY OF EHRLICHIA-INDUCED CYTOPATHIC EFFECT AND PLAQUE-FORMATION. Infection and Immunity. 1995;63(2):647-55. doi: 10.1128/iai.63.2.647-655.1995. PubMed PMID: WOS:A1995QC60300040.

727. Cianciotto NP, O'Connell W, Dasch GA, Mallavia LP. Detection of mip-like sequences and Mip-related proteins within the family Rickettsiaceae. Curr Microbiol. 1995;30(3):149-53. Epub 1995/03/01. doi: 10.1007/bf00296200. PubMed PMID: 7532505.

728. Cotta MA, Zeltwanger RL. Degradation and utilization of xylan by the ruminal bacteria Butyrivibrio fibrisolvens and Selenomonas ruminantium. Applied and Environmental Microbiology. 1995;61(12):4396-402. doi: 10.1128/aem.61.12.4396-4402.1995.

729. Curry AJ, Else KJ, Jones F, Bancroft A, Grencis RK, Dunne DW. Evidence that cytokine-mediated immune interactions induced by Schistosoma mansoni alter disease outcome in mice concurrently infected with Trichuris muris. J Exp Med. 1995;181(2):769-74. Epub 1995/02/01. doi: 10.1084/jem.181.2.769. PubMed PMID: 7836929; PubMed Central PMCID: PMCPMC2191884.

730. Curry AJ, Else KJ, Jones F, Bancroft A, Grencis RK, Dunne DW. Evidence that cytokine-mediated immune interactions induced by Schistosoma Mansonl alter disease outcome in mice concurrently infected with Trichuris Muris. Journal of Experimental Medicine. 1995;181(2):769-74. doi: 10.1084/jem.181.2.769.

731. Curry AJ, Else KJ, Jones F, Bancroft A, Grencis RK, Dunne DW. EVIDENCE THAT CYTOKINE-MEDIATED IMMUNE INTERACTIONS INDUCED BY SCHISTOSOMA-MANSONI ALTER DISEASE OUTCOME IN MICE CONCURRENTLY INFECTED WITH TRICHURIS-MURIS. Journal of Experimental Medicine. 1995;181(2):769-74. doi: 10.1084/jem.181.2.769. PubMed PMID: WOS:A1995QD01200033.

732. Dumler JS, Asanovich KM, Bakken JS, Richter P, Kimsey R, Madigan JE. Serologic cross-reactions among Ehrlichia equi, Ehrlichia phagocytophila, and human granulocytic Ehrlichia. J Clin Microbiol. 1995;33(5):1098-103. Epub 1995/05/01. doi: 10.1128/jcm.33.5.1098-1103.1995. PubMed PMID: 7542262; PubMed Central PMCID: PMCPMC228112.

733. Dumler JS, Asanovich KM, Bakken JS, Richter P, Kimsey R, Madigan JE. SEROLOGIC CROSS-REACTIONS AMONG EHRLICHIA-EQUI, EHRLICHIA-PHAGOCYTOPHILA, AND HUMAN GRANULOCYTIC EHRLICHIA. Journal of Clinical Microbiology. 1995;33(5):1098-103. doi: 10.1128/jcm.33.5.1098-1103.1995. PubMed PMID: WOS:A1995QT30600011.

734. Dumler JS, Bakken JS. Ehrlichial diseases of humans: emerging tick-borne infections. Clin Infect Dis. 1995;20(5):1102-10. Epub 1995/05/01. doi: 10.1093/clinids/20.5.1102. PubMed PMID: 7619983.

735. Dumler JS, Bakken JS. EHRLICHIAL DISEASES OF HUMANS - EMERGING TICK-BORNE INFECTIONS. Clinical Infectious Diseases. 1995;20(5):1102-10. doi: 10.1093/clinids/20.5.1102. PubMed PMID: WOS:A1995QY77100002.

736. Dumler JS, Chen SM, Asanovich K, Trigiani E, Popov VL, Walker DH. Isolation and characterization of a new strain of Ehrlichia chaffeensis from a patient with nearly fatal monocytic ehrlichiosis. J Clin Microbiol. 1995;33(7):1704-11. Epub 1995/07/01. doi: 10.1128/jcm.33.7.1704-1711.1995. PubMed PMID: 7665632; PubMed Central PMCID: PMCPMC228253.

737. Dumler JS, Chen SM, Asanovich K, Trigiani E, Popov VL, Walker DH. ISOLATION AND CHARACTERIZATION OF A NEW STRAIN OF EHRLICHIA-CHAFFEENSIS FROM A PATIENT WITH NEARLY FATAL MONOCYTIC EHRLICHIOSIS. Journal of Clinical Microbiology. 1995;33(7):1704-11. doi: 10.1128/jcm.33.7.1704-1711.1995. PubMed PMID: WOS:A1995RD99000004.

738. Eaton DC, Gabelman A. Fed-batch and continuous fermentation of Selenomonas ruminantium for natural propionic, acetic and succinic aids. Journal of Industrial Microbiology. 1995;15(1):32-8.

739. Everett ED. Human Ehrlichia infections. Current Opinion in Infectious Diseases. 1995;8(2):127-9. doi: 10.1097/00001432-199504000-00009.

740. Ewing SA, Dawson JE, Kocan AA, Barker RW, Warner CK, Panciera RJ, et al. EXPERIMENTAL TRANSMISSION OF EHRLICHIA-CHAFFEENSIS (RICKETTSIALES, EHRLICHIEAE) AMONG WHITE-TAILED DEER BY AMBLYOMMA-AMERICANUM (ACARI, IXODIDAE). Journal of Medical Entomology. 1995;32(3):368-74. doi: 10.1093/jmedent/32.3.368. PubMed PMID: WOS:A1995QW42900021.

741. Fichtenbaum CJ, Weil GJ. HUMAN EHRLICHIOSIS WITH FEATURES OF TOXIC SHOCK SYNDROME - REPLY. American Journal of Medicine. 1995;99(1):107-. doi: 10.1016/s0002-9343(99)80121-1. PubMed PMID: WOS:A1995RH25900026.

742. Fishbein DB, Dennis DT. Tick-borne diseases – a growing risk. New England Journal of Medicine. 1995;333(7):452-3. doi: 10.1056/NEJM199508173330711.

743. Fishbein DB, Dennis DT. TICK-BORNE DISEASES - A GROWING RISK. New England Journal of Medicine. 1995;333(7):452-3. doi: 10.1056/nejm199508173330711. PubMed PMID: WOS:A1995RP24300011.

744. Gage KL, Ostfeld RS, Olson JG. Nonviral vector-borne zoonoses associated with mammals in the United States. Journal of Mammalogy. 1995;76(3):695-715. doi: 10.2307/1382741.

745. Gage KL, Ostfeld RS, Olson JG. NONVIRAL VECTOR-BORNE ZOONOSES ASSOCIATED WITH MAMMALS IN THE UNITED-STATES. Journal of Mammalogy. 1995;76(3):695-715. doi: 10.2307/1382741. PubMed PMID: WOS:A1995RQ81100004.

746. Ghiglietti R, Rossi P, Ramsan M, Colombi A. Viability of Ascaris suum, Ascaris lumbricoides and Trichuris muris eggs to alkaline pH and different temperatures. Parassitologia. 1995;37(2-3):229-32. Epub 1995/12/01. PubMed PMID: 8778663.

747. Glaser C, Johnson E. Images in clinical medicine. Ehrlichiosis. N Engl J Med. 1995;332(21):1417. Epub 1995/05/25. doi: 10.1056/nejm199505253322105. PubMed PMID: 7723798.

748. Guo ZG, Johnson AM. Genetic comparison of neospora caninum with toxoplasma and sarcocystis by random amplified polymorphic DNA-polymerase chain reaction. Parasitology Research. 1995;81(5):365-70.

749. Gupta RS. Evolution of the chaperonin families (Hsp60, Hsp10 and Tcp-1) of proteins and the origin of eukaryotic cells. Mol Microbiol. 1995;15(1):1-11. Epub 1995/01/01. doi: 10.1111/j.1365-2958.1995.tb02216.x. PubMed PMID: 7752884.

750. Hackstein JH, Mackenstedt U, Mehlhorn H, Meijerink JP, Schubert H, Leunissen JA. Parasitic apicomplexans harbor a chlorophyll a-D1 complex, the potential target for therapeutic triazines. Parasitol Res. 1995;81(3):207-16. Epub 1995/01/01. doi: 10.1007/bf00937111. PubMed PMID: 7770426.

751. Hackstein JHP, Mackenstedt U, Mehlhorn H, Meijerink JPP, Schubert H, Leunissen JAM. Parasitic apicomplexans harbor a chlorophyll a-D1 complex, the potential target for therapeutic triazines. Parasitology Research. 1995;81(3):207-16.

752. Hardalo CJ, Quagliarello V, Dumler JS. Human granulocytic ehrlichiosis in Connecticut: report of a fatal case. Clin Infect Dis. 1995;21(4):910-4. Epub 1995/10/01. doi: 10.1093/clinids/21.4.910. PubMed PMID: 8645839.

753. Hardalo CJ, Quagliarello V, Dumler JS. HUMAN GRANULOCYTIC EHRLICHIOSIS IN CONNECTICUT - REPORT OF A FATAL CASE. Clinical Infectious Diseases. 1995;21(4):910-4. doi: 10.1093/clinids/21.4.910. PubMed PMID: WOS:A1995RZ13900012.

754. Heymann WR. Human ehrlichiosis. Int J Dermatol. 1995;34(9):618-9. Epub 1995/09/01. doi: 10.1111/j.1365-4362.1995.tb01085.x. PubMed PMID: 7591456.

755. Higgins JA, Azad AF. USE OF POLYMERASE CHAIN-REACTION TO DETECT BACTERIA IN ARTHROPODS - A REVIEW. Journal of Medical Entomology. 1995;32(3):213-22. doi: 10.1093/jmedent/32.3.213. PubMed PMID: WOS:A1995QW42900001.

756. Holmes AH, Greenough TC, Balady GJ, Regnery RL, Anderson BE, Okeane JC, et al. BARTONELLA HENSELAE ENDOCARDITIS IN AN IMMUNOCOMPETENT ADULT. Clinical Infectious Diseases. 1995;21(4):1004-7. doi: 10.1093/clinids/21.4.1004. PubMed PMID: WOS:A1995RZ13900031.

757. Johansson KE, Pettersson B, Uhlén M, Gunnarsson A, Malmqvist M, Olsson E. Identification of the causative agent of granulocytic ehrlichiosis in Swedish dogs and horses by direct solid phase sequencing of PCR products from the 16S rRNA gene. Research in Veterinary Science. 1995;58(2):109-12. doi: 10.1016/0034-5288(95)90061-6.

758. Johansson KE, Pettersson B, Uhlen M, Gunnarsson A, Malmqvist M, Olsson E. IDENTIFICATION OF THE CAUSATIVE AGENT OF GRANULOCYTIC EHRLICHIOSIS IN SWEDISH DOGS AND HORSES BY DIRECT SOLID-PHASE SEQUENCING OF PCR PRODUCTS FROM THE 16S RIBOSOMAL-RNA GENE. Research in Veterinary Science. 1995;58(2):109-12. doi: 10.1016/0034-5288(95)90061-6. PubMed PMID: WOS:A1995QN38400002.

759. Kanof EP. Rocky Mountain Spotted Fever, tick paralysis, Lyme disease, and Ehrlichiosis. N C Med J. 1995;56(7):319-22. Epub 1995/07/01. PubMed PMID: 7643927.

760. Kfir R, Hilner C, Du Preez M, Bateman B. Studies evaluating the applicability of utilising the same concentration techniques for the detection of protozoan parasites and viruses in water. Water Science and Technology. 1995;31(5-6):417-23. doi: 10.1016/0273-1223(95)00303-5.

761. Knysz B, Inglot M, Gladysz A, Augustyniak K. [Ehrlichiosis: a new infectious disease]. Przegl Epidemiol. 1995;49(1-2):3-7. Epub 1995/01/01. PubMed PMID: 7676057.

762. Kock ND, Van Vliet AHM, Charlton K, Jongejan F. Detection of Cowdria ruminantium in blood and bone marrow samples from clinically normal, free-ranging Zimbabwean wild ungulates. Journal of Clinical Microbiology. 1995;33(9):2501-4. doi: 10.1128/jcm.33.9.2501-2504.1995.

763. Lee E, Rikihisa Y. CYTOKINE NRNA EXPRESSION IN HUMAN MONOCYTES INFECTED WITH EHRLICHIA-CHAFFEENSIS. Molecular Biology of the Cell. 1995;6:636-. PubMed PMID: WOS:A1995TF51300636.

764. Lee TDG, Xie CY. IGE REGULATION BY NEMATODES - THE BODY-FLUID OF ASCARIS CONTAINS A B-CELL MITOGEN. Journal of Allergy and Clinical Immunology. 1995;95(6):1246-54. doi: 10.1016/s0091-6749(95)70082-x. PubMed PMID: WOS:A1995RD30900013.

765. Letaief AO, Yacoub S, Dupont H, Cam CL, Ghachem L, Jemni L, et al. Seroepidemiological survey of rickettsial infections among blood donors in central Tunisia. Transactions of the Royal Society of Tropical Medicine and Hygiene. 1995;89(3):266-8. doi: 10.1016/0035-9203(95)90531-6.

766. Letaief AO, Yacoub S, Dupont HT, Le Cam C, Ghachem L, Jemni L, et al. Seroepidemiological survey of rickettsial infections among blood donors in central Tunisia. Trans R Soc Trop Med Hyg. 1995;89(3):266-8. Epub 1995/05/01. doi: 10.1016/0035-9203(95)90531-6. PubMed PMID: 7660428.

767. Lillywhite JE, Cooper ES, Needham CS, Venugopal S, Bundy DA, Bianco AE. Identification and characterization of excreted/secreted products of Trichuris trichiura. Parasite Immunol. 1995;17(1):47-54. Epub 1995/01/01. doi: 10.1111/j.1365-3024.1995.tb00965.x. PubMed PMID: 7731735.

768. Lillywhite JE, Cooper ES, Needham CS, Venugopal S, Bundy DAP, Bianco AE. Identification and characterization of excreted/secreted products of Trichuris trichiura. Parasite Immunology. 1995;17(1):47-54. doi: 10.1111/j.1365-3024.1995.tb00965.x.

769. Lillywhite JE, Cooper ES, Needham CS, Venugopal S, Bundy DAP, Bianco AE. IDENTIFICATION AND CHARACTERIZATION OF EXCRETED SECRETED PRODUCTS OF TRICHURIS-TRICHIURA. Parasite Immunology. 1995;17(1):47-54. doi: 10.1111/j.1365-3024.1995.tb00965.x. PubMed PMID: WOS:A1995QG75500006.

770. Ling JR, Armstead IP. The in vitro uptake and metabolism of peptides and amino acids by five species of rumen bacteria. Journal of Applied Bacteriology. 1995;78(2):116-24.

771. Lockhart JM, Davidson WR, Dawson JE, Stallknecht DE. Temporal association of Amblyomma americanum with the presence of Ehrlichia chaffeensis reactive antibodies in white-tailed deer. Journal of wildlife diseases. 1995;31(2):119-24. doi: 10.7589/0090-3558-31.2.119.

772. Lockhart JM, Davidson WR, Dawson JE, Stallknecht DE. TEMPORAL ASSOCIATION OF AMBLYOMMA-AMERICANUM WITH THE PRESENCE OF EHRLICHIA-CHAFFEENSIS REACTIVE ANTIBODIES IN WHITE-TAILED DEER. Journal of Wildlife Diseases. 1995;31(2):119-24. doi: 10.7589/0090-3558-31.2.119. PubMed PMID: WOS:A1995QU26700001.

773. Macpherson CNL. The effect of transhumance on the epidemiology of animal diseases. Preventive Veterinary Medicine. 1995;25(2):213-24. doi: 10.1016/0167-5877(95)00539-0.

774. Madigan JE, Richter PJ, Kimsey RB, Barlough JE, Bakken JS. TRANSMISSION AND PASSAGE IN HORSES OF THE AGENT OF HUMAN GRANULOCYTIC EHRLICHIOSIS. Journal of Infectious Diseases. 1995;172(4):1141-4. doi: 10.1093/infdis/172.4.1141. PubMed PMID: WOS:A1995RW06200040.

775. Madigan JE, Richter PJ, Jr., Kimsey RB, Barlough JE, Bakken JS, Dumler JS. Transmission and passage in horses of the agent of human granulocytic ehrlichiosis. J Infect Dis. 1995;172(4):1141-4. Epub 1995/10/01. doi: 10.1093/infdis/172.4.1141. PubMed PMID: 7561199.

776. Magnarelli LA, Dumler JS, Anderson JF, Johnson RC, Fikrig E. Coexistence of antibodies to tick-borne pathogens of babesiosis, ehrlichiosis, and Lyme borreliosis in human sera. J Clin Microbiol. 1995;33(11):3054-7. Epub 1995/11/01. doi: 10.1128/jcm.33.11.3054-3057.1995. PubMed PMID: 8576376; PubMed Central PMCID: PMCPMC228637.

777. Magnarelli LA, Stafford Iii KC, Mather TN, Yeh MT, Horn KD, Dumler JS. Hemocytic rickettsia-like organisms in ticks: Serologic reactivity with antisera to ehrlichiae and detection of DNA of agent of human granulocytic ehrlichiosis by PCR. Journal of Clinical Microbiology. 1995;33(10):2710-4. doi: 10.1128/jcm.33.10.2710-2714.1995.

778. Magnarelli LA, Stafford KC, 3rd, Mather TN, Yeh MT, Horn KD, Dumler JS. Hemocytic rickettsia-like organisms in ticks: serologic reactivity with antisera to Ehrlichiae and detection of DNA of agent of human granulocytic ehrlichiosis by PCR. J Clin Microbiol. 1995;33(10):2710-4. Epub 1995/10/01. doi: 10.1128/jcm.33.10.2710-2714.1995. PubMed PMID: 8567911; PubMed Central PMCID: PMCPMC228561.

779. Magnarelli LA, Stafford KC, Mather TN, Yeh MT, Horn KD, Dumler JS. HEMOCYTIC RICKETTSIA-LIKE ORGANISMS IN TICKS - SEROLOGIC REACTIVITY WITH ANTISERA TO EHRLICHIAE AND DETECTION OF DNA OF AGENT OF HUMAN GRANULOCYTIC EHRLICHIOSIS BY PCR. Journal of Clinical Microbiology. 1995;33(10):2710-4. doi: 10.1128/jcm.33.10.2710-2714.1995. PubMed PMID: WOS:A1995RV56500035.

780. Mahan SM, Peter TF, Semu SM, Simbi BH, Norval RA, Barbet AF. Laboratory reared Amblyomma hebraeum and Amblyomma variegatum ticks differ in their susceptibility to infection with Cowdria ruminantium. Epidemiology & Infection. 1995;115(2):345-53. doi: 10.1017/S0950268800058465. PubMed PMID: 104780018. Language: English. Entry Date: 20110610. Revision Date: 20200708. Publication Type: journal article.

781. Majewska AC. COMPARATIVE-STUDIES OF EXPERIMENTAL GIARDIOSIS IN MONGOLIAN GERBILS .2. INFECTIONS INDUCED WITH DIFFERENT GIARDIA ISOLATES FROM ZOO ANIMALS. Acta Protozoologica. 1995;34(2):95-100. PubMed PMID: WOS:A1995QV07500003.

782. Marrie TJ, Purdy RA, Johnston BL, McCormick CW, Benstead T, Ansell J, et al. ENCEPHALOMYELORADICULOPATHY OF INFECTIOUS OR PARAINFECTIOUS ETIOLOGY - A NEW ENTITY. Clinical Infectious Diseases. 1995;20(4):945-53. doi: 10.1093/clinids/20.4.945. PubMed PMID: WOS:A1995QP66200034.

783. Marsh AE, Barr BC, Sverlow K, Ho M, Dubey JP, Conrad PA. Sequence analysis and comparison of ribosomal DNA from bovine Neospora to similar coccidial parasites. J Parasitol. 1995;81(4):530-5. Epub 1995/08/01. PubMed PMID: 7623193.

784. Marty AM, Dumler JS, Imes G, Brusman HP, Smrkovski LL, Frisman DM. Ehrlichiosis mimicking thrombotic thrombocytopenic purpura. Case report and pathological correlation. Hum Pathol. 1995;26(8):920-5. Epub 1995/08/01. doi: 10.1016/0046-8177(95)90017-9. PubMed PMID: 7635455.

785. Mayrhofer G, Andrews RH, Ey PL. Division of Giardia isolates from humans into two genetically distinct assemblages by electrophoretic analysis of enzymes encoded at 27 loci and comparison with Giardia muris. Parasitology. 1995;111(1):11-7. doi: 10.1017/S0031182000064556.

786. Mayrhofer G, Andrews RH, Ey PL, Chilton NB. Division of Giardia isolates from humans into two genetically distinct assemblages by electrophoretic analysis of enzymes encoded at 27 loci and comparison with Giardia muris. Parasitology. 1995;111 ( Pt 1):11-7. Epub 1995/07/01. doi: 10.1017/s0031182000064556. PubMed PMID: 7609985.

787. Mayrhofer G, Andrews RH, Ey PL, Chilton NB. DIVISION OF GIARDIA ISOLATES FROM HUMANS INTO 2 GENETICALLY DISTINCT ASSEMBLAGES BY ELECTROPHORETIC ANALYSIS OF ENZYMES ENCODED AT 27-LOCI AND COMPARISON WITH GIARDIA-MURIS. Parasitology. 1995;111:11-7. doi: 10.1017/s0031182000064556. PubMed PMID: WOS:A1995RH33000002.

788. Meskini M, Beati L, Benslimane A, Raoult D. Seroepidemiology of rickettsial infections in Morocco. Eur J Epidemiol. 1995;11(6):655-60. Epub 1995/12/01. doi: 10.1007/bf01720299. PubMed PMID: 8861849.

789. Michel R, Muller KD, Schmid EN. EHRLICHIA-LIKE ORGANISMS (KSL(1)) OBSERVED AS OBLIGATE INTRACELLULAR PARASITES OF SACCAMOEBA SPECIES. Endocytobiosis and Cell Research. 1995;11(1):69-80. PubMed PMID: WOS:A1995RR39000005.

790. Mitsumori M, Minato H. Distribution of cellulose-binding proteins among the representative strains of rumen bacteria. Journal of General and Applied Microbiology. 1995;41(4):297-306.

791. Nakamura I, Ogimoto K, Izumi H. Antimicrobial activity of lasalocid against Selenomonas ruminantium--effect of changes in pH induced by changing glucose concentration. J Vet Med Sci. 1995;57(4):611-6. Epub 1995/08/01. doi: 10.1292/jvms.57.611. PubMed PMID: 8519886.

792. Oriola Font S, Loriente Tur MM, Manas Pagan E, Margarit Vercher T, Martinez Costa C. Abdominal pain and intestinal parasitism in paediatric population. Acta Pediatrica Espanola. 1995;53(7):428-32.

793. Oteo JA, Martínez de Artola V, Eiros JM. [Human ehrlichiosis in Spain. Various epidemiologic considerations]. Enferm Infecc Microbiol Clin. 1995;13(4):265. Epub 1995/04/01. PubMed PMID: 7779887.

794. Pancholi P, Kolbert CP, Mitchell PD, Reed KD, Dumler JS, Bakken JS, et al. Ixodes dammini as a potential vector of human granulocytic ehrlichiosis. Journal of Infectious Diseases. 1995;172(4):1007-12.

795. Pancholi P, Kolbert CP, Mitchell PD, Reed KD, Jr., Dumler JS, Bakken JS, et al. Ixodes dammini as a potential vector of human granulocytic ehrlichiosis. J Infect Dis. 1995;172(4):1007-12. Epub 1995/10/01. doi: 10.1093/infdis/172.4.1007. PubMed PMID: 7561173.

796. Pancholi P, Kolbert CP, Mitchell PD, Reed KD, Dumler JS, Bakken JS, et al. IXODES-DAMMINI AS A POTENTIAL VECTOR OF HUMAN GRANULOCYTIC EHRLICHIOSIS. Journal of Infectious Diseases. 1995;172(4):1007-12. doi: 10.1093/infdis/172.4.1007. PubMed PMID: WOS:A1995RW06200014.

797. Pierard D, Levtchenko N, Dawson JE, Lauwers S. Ehrlichiosis in Belgium [20]. Lancet. 1995;346(8984):1233-4.

798. Pretzman C, Ralph D, Stothard DR, Fuerst PA, Rikihisa Y. 16S rRNA gene sequence of Neorickettsia helminthoeca and its phylogenetic alignment with members of the genus Ehrlichia. International Journal of Systematic Bacteriology. 1995;45(2):207-11. doi: 10.1099/00207713-45-2-207.

799. Pristaš P. Isolation and characterization of a new restriction endonuclease, Sru30DI, from Selenomonas ruminantium. Gene. 1995;158(1):139-40. doi: 10.1016/0378-1119(95)00093-L.

800. Pruthi RK, Marshall WF, Wiltsie JC, Persing DH. Human Babesiosis. Mayo Clinic Proceedings. 1995;70(9):853-62. doi: 10.4065/70.9.853.

801. Reed KD, Mitchell PD, Persing DH, Kolbert CP, Cameron V. Transmission of human granulocytic ehrlichiosis. Jama. 1995;273(1):23. Epub 1995/01/04. PubMed PMID: 7864987.

802. Reed KD, Mitchell PD, Persing DH, Kolbert CP, Cameron V, Bakken JS, et al. Transmission of human granulocytic ehrlichiosis [3]. JAMA. 1995;273(1):23. doi: 10.1001/jama.273.1.23.

803. Rikihisa Y, Zhang Y, Park J. Role of Ca2+ and calmodulin in ehrlichial infection in macrophages. Infection and Immunity. 1995;63(6):2310-6. doi: 10.1128/iai.63.6.2310-2316.1995.

804. Roland WE, McDonald G, Caldwell CW, Everett ED. Ehrlichiosis--a cause of prolonged fever. Clin Infect Dis. 1995;20(4):821-5. Epub 1995/04/01. doi: 10.1093/clinids/20.4.821. PubMed PMID: 7795080.

805. Roland WE, McDonald G, Caldwell CW, Everett ED. Ehrlichiosis—A Cause of Prolonged Fever. Clinical Infectious Diseases. 1995;20(4):821-5. doi: 10.1093/clinids/20.4.821.

806. Roux V, Raoult D. Phylogenetic analysis of the genus Rickettsia by 16S rDNA sequencing. Res Microbiol. 1995;146(5):385-96. Epub 1995/06/01. doi: 10.1016/0923-2508(96)80284-1. PubMed PMID: 8525055.

807. Salgado JH, Evans ME, Hoven AD, Noble RC. Ehrlichiosis in Kentucky. J Ky Med Assoc. 1995;93(4):132-5. Epub 1995/04/01. PubMed PMID: 7751803.

808. Skene IK, Brooker JD. Characterization of tannin acylhydrolase activity in the ruminal bacterium Selenomonas ruminantium. Anaerobe. 1995;1(6):321-7. Epub 1995/12/01. doi: 10.1006/anae.1995.1034. PubMed PMID: 16887543.

809. Standaert SM, Dawson JE, Schaffner W, Childs JE, Biggie KL, Singleton J, Jr., et al. Ehrlichiosis in a golf-oriented retirement community. N Engl J Med. 1995;333(7):420-5. Epub 1995/08/17. doi: 10.1056/nejm199508173330704. PubMed PMID: 7616991.

810. Standaert SM, Dawson JE, Schaffner W, Childs JE, Biggie KL, Singleton Jr J, et al. Ehrlichiosis in a golf-oriented retirement community. New England Journal of Medicine. 1995;333(7):420-5. doi: 10.1056/NEJM199508173330704.

811. Sumption KJ, Wright DJ, Cutler SJ, Dale BA. Human ehrlichiosis in the UK. Lancet. 1995;346(8988):1487-8. Epub 1995/12/02. doi: 10.1016/s0140-6736(95)92502-3. PubMed PMID: 7491006.

812. Sumption KJ, Wright DJM, Cutler SJ, Dale BAS. Human ehrlichiosis in the UK [4]. Lancet. 1995;346(8988):1487-8.

813. Sumption KJ, Wright DJM, Cutler SJ, Dale BAS. Human ehrlichiosis in the UK. The Lancet. 1995;346(8988):1487-8. doi: 10.1016/S0140-6736(95)92502-3.

814. Tal A, Shannahan D. Ehrlichiosis presenting as a life-threatening illness. Am J Med. 1995;98(3):318-9. Epub 1995/03/01. doi: 10.1016/s0002-9343(99)80388-x. PubMed PMID: 7872355.

815. Tatar G, Haziroğlu R, Hasçelik G. Helicobacter felis as a cofactor alone or together with stress in cryptosporidial activation in mice. J Int Med Res. 1995;23(6):473-9. Epub 1995/11/01. doi: 10.1177/030006059502300609. PubMed PMID: 8746615.

816. Telford Iii SR, Lepore TJ, Snow P, Warner CK, Dawson JE. Human granulocytic ehrlichiosis in Massachusetts. Annals of Internal Medicine. 1995;123(4):277-9. doi: 10.7326/0003-4819-123-4-199508150-00006.

817. Telford SR, 3rd, Lepore TJ, Snow P, Warner CK, Dawson JE. Human granulocytic ehrlichiosis in Massachusetts. Ann Intern Med. 1995;123(4):277-9. Epub 1995/08/15. doi: 10.7326/0003-4819-123-4-199508150-00006. PubMed PMID: 7611594.

818. Telford SR, 3rd, Lepore TJ, Snow P, Warner CK, Dawson JE, Telford SR, 3rd, et al. Human granulocytic ehrlichiosis in Massachusetts. Annals of Internal Medicine. 1995;123(4):277-9. doi: 10.7326/0003-4819-123-4-199508150-00006. PubMed PMID: 105852571. Language: English. Entry Date: 20080314. Revision Date: 20200708. Publication Type: journal article.

819. Van Vliet AHM, Van der Zeijst BAM, Camus E, Mahan SM, Martinez D, Jongejan F. Use of a specific immunogenic region on the Cowdria ruminantium MAP1 protein in a serological assay. Journal of Clinical Microbiology. 1995;33(9):2405-10. doi: 10.1128/jcm.33.9.2405-2410.1995.

820. Vankeulen H, Homan WL, Erlandsen SL, Jarroll EL. A 3 NUCLEOTIDE SIGNATURE SEQUENCE IN SMALL-SUBUNIT RIBOSOMAL-RNA DIVIDES HUMAN GIARDIA IN 2 DIFFERENT GENOTYPES. Journal of Eukaryotic Microbiology. 1995;42(4):392-4. PubMed PMID: WOS:A1995RK91300010.

821. Vemulapalli R, Biswas B, Dutta SK. Pathogenic, immunologic, and molecular differences between two Ehrlichia risticii strains. Journal of Clinical Microbiology. 1995;33(11):2987-93. doi: 10.1128/jcm.33.11.2987-2993.1995. PubMed Central PMCID: PMCDifco(United States)

Genzyme(United States).

822. Wen B, Rikihisa Y, Mott J, Fuerst PA, Kawahara M, Suto C. Ehrlichia muris sp. nov., identified on the basis of 16S rRNA base sequences and serological, morphological, and biological characteristics. International Journal of Systematic Bacteriology. 1995;45(2):250-4. doi: 10.1099/00207713-45-2-250.

823. Wen BH, Rikihisa Y, Mott J, Fuerst PA, Kawahara M, Suto C. EHRLICHIA MURIS SP-NOV, IDENTIFIED ON THE BASIS OF 16S RIBOSOMAL-RNA BASE SEQUENCES AND SEROLOGICAL, MORPHOLOGICAL, AND BIOLOGICAL CHARACTERISTICS. International Journal of Systematic Bacteriology. 1995;45(2):250-4. doi: 10.1099/00207713-45-2-250. PubMed PMID: WOS:A1995QR83400010.

824. Wormser G. HUMAN GRANULOCYTIC EHRLICHIOSIS - NEW-YORK, 1995 (VOL 274, PG 867, 1995). Jama-Journal of the American Medical Association. 1995;274(12):937-. PubMed PMID: WOS:A1995RV73400009.

825. Wormser G, McKenna D, Aguerorosenfeld M, Horowitz H, Munoz J, Nowakowski J, et al. HUMAN GRANULOCYTIC EHRLICHIOSIS - NEW-YORK, 1995 (REPRINTED FROM MMWR, VOL 44, PG 593, 1995). Jama-Journal of the American Medical Association. 1995;274(11):867-. PubMed PMID: WOS:A1995RU60300006.

826. Wu ZL, Iseki M, Kimata I, Xu D, Nagano I, Takahashi Y. Construction of polymerase chain reaction primer to detect Cryptosporidium parvum or C-muris. Journal of Protozoology Research. 1995;5(4):149-56. PubMed PMID: WOS:000169555800003.

827. Yevich SJ, Sánchez JL, DeFraites RF, Rives CC, Dawson JE, Uhaa IJ, et al. Seroepidemiology of infections due to spotted fever group rickettsiae and Ehrlichia species in military personnel exposed in areas of the United States where such infections are endemic. J Infect Dis. 1995;171(5):1266-73. Epub 1995/05/01. doi: 10.1093/infdis/171.5.1266. PubMed PMID: 7751702.

828. Yevich SJ, Sánchez JL, DeFraites RF, Rives CC, Dawson JE, Uhaa IJ, et al. Seroepidemiology of infections due to spotted fever group rickettsiae and Ehrlichia species in military personnel exposed in areas of the United States where such infections are endemic. Journal of Infectious Diseases. 1995;171(5):1266-73.

829. Human ehrlichiosis--Maryland, 1994. MMWR Morb Mortal Wkly Rep. 1996;45(37):798-802. Epub 1996/09/20. PubMed PMID: 8926996.

830. From the Centers for Disease Control and Prevention. Human ehrlichiosis--Maryland, 1994. Jama. 1996;276(15):1212-3. Epub 1996/10/16. PubMed PMID: 8849737.

831. Erratum: Direct cultivation of the causative agent of human granulocytic ehrlichiosis (New England Journal of Medicine (Jan 25, 1996) 334 (209-15)). New England Journal of Medicine. 1996;335(5):361. doi: 10.1056/NEJM199608013350522.

832. Human ehrlichiosis - Maryland, 1994. JAMA. 1996;276(15):1212-3. doi: 10.1001/jama.276.15.1212.

833. Getting a head start against ehrlichiosis. Emergency Medicine (00136654). 1996;28(5):148-55. PubMed PMID: 107347462. Language: English. Entry Date: 19971201. Revision Date: 20150711. Publication Type: Journal Article.

834. Diagnosing ehrlichiosis. American College of Physicians; 1996. p. 854-5.

835. Aguero-Rosenfeld ME, Horowitz HW, Wormser GP, McKenna DF, Nowakowski J, Muñoz J, et al. Human granulocytic ehrlichiosis: a case series from a medical center in New York State. Ann Intern Med. 1996;125(11):904-8. Epub 1996/12/01. doi: 10.7326/0003-4819-125-11-199612010-00006. PubMed PMID: 8967671.

836. AgueroRosenfeld ME, Horowitz HW, Wormser GP, McKenna DF, Nowakowski J, Munoz J, et al. Human granulocytic ehrlichiosis: A case series from a medical center in New York state. Annals of Internal Medicine. 1996;125(11):904-8. doi: 10.7326/0003-4819-125-11-199612010-00006. PubMed PMID: WOS:A1996VV54100006.

837. Ahkee S, Ramirez J. A case of concurrent Lyme meningitis with ehrlichiosis. Scand J Infect Dis. 1996;28(5):527-8. Epub 1996/01/01. doi: 10.3109/00365549609037953. PubMed PMID: 8953687.

838. Alleman AR, Barbet AF. Evaluation of Anaplasma marginale major surface protein 3 (MSP3) as a diagnostic test antigen. Journal of Clinical Microbiology. 1996;34(2):270-6.

839. Allsopp BA, Allsopp MT, Du Plessis JH, Visser AS. Uncharacterized Ehrlichia spp. may contribute to clinical heartwater. 1996. p. 17-23.

840. Allsopp BA, Allsopp MT, Du Plessis JH, Visser ES. Uncharacterized Ehrlichia spp. may contribute to clinical heartwater. Ann N Y Acad Sci. 1996;791:17-23. Epub 1996/07/23. doi: 10.1111/j.1749-6632.1996.tb53507.x. PubMed PMID: 8784482.

841. Arraga-Alvarado C, Montero-Ojeda M, Bernardoni A, Anderson BE, Parra O. [Human ehrlichiosis: report of the 1st case in Venezuela]. Invest Clin. 1996;37(1):35-49. Epub 1996/03/01. PubMed PMID: 8920030.

842. Arraga-Alvarado C, Montero-Ojeda M, Bernardoni A, Anderson BE, Parra O. Human ehrlichiosis: Report of the first Venezuelan case. Investigacion Clinica. 1996;37(1):35-49.

843. Bakken JS. Human granulocytic ehrlichiosis in the United States. Infections in Medicine. 1996;13(10):877-912.

844. Bakken JS, Krueth J, Tilden RL, Dumler JS, Kristiansen BE. Serological evidence of human granulocytic ehrlichiosis in Norway. Eur J Clin Microbiol Infect Dis. 1996;15(10):829-32. Epub 1996/10/01. doi: 10.1007/bf01701530. PubMed PMID: 8950565.

845. Bakken JS, Krueth J, Wilson-Nordskog C, Tilden RL, Asanovich K, Dumler JS. Clinical and laboratory characteristics of human granulocytic ehrlichiosis. Jama. 1996;275(3):199-205. Epub 1996/01/17. PubMed PMID: 8604172.

846. Bakken JS, Krueth J, WilsonNordskog C, Tilden RL, Asanovich K, Dumler JS. Clinical and laboratory characteristics of human granulocytic ehrlichiosis. Jama-Journal of the American Medical Association. 1996;275(3):199-205. doi: 10.1001/jama.275.3.199. PubMed PMID: WOS:A1996TP28200027.

847. Bakken JS, Krueth JK, Lund T, Malkovitch D, Asanovich K, Dumler JS. Exposure to deer blood may be a cause of human granulocytic ehrlichiosis. Clin Infect Dis. 1996;23(1):198. Epub 1996/07/01. doi: 10.1093/clinids/23.1.198. PubMed PMID: 8816164.

848. Barbour AG. Does Lyme disease occur in the south?: A survey of emerging tick-borne infections in the region. American Journal of the Medical Sciences. 1996;311(1):34-40. doi: 10.1097/00000441-199601000-00007.

849. Barenfanger J, Patel PG, Dumler JS, Walker DH. Identifying human ehrlichiosis. Laboratory Medicine. 1996;27(6):372-4.

850. Barenfanger J, Patel PG, Dumler JS, Walker DH. Clinical pathology rounds. Identifying human ehrlichiosis. Laboratory Medicine. 1996;27(6):372-4. PubMed PMID: 107377177. Language: English. Entry Date: 19960801. Revision Date: 20150819. Publication Type: Journal Article.

851. Barlough JE, Madigan JE, DeRock E, Bigornia L. Nested polymerase chain reaction for detection of Ehrlichia equi genomic DNA in horses and ticks (Ixodes pacificus). Vet Parasitol. 1996;63(3-4):319-29. Epub 1996/06/01. doi: 10.1016/0304-4017(95)00904-3. PubMed PMID: 8966998.

852. Brouqui P, Raouit D. Bartonella quintana invades and multiplies within endothelial cells in vitro and in vivo and forms intracellular blebs. Research in Microbiology. 1996;147(9):719-31. doi: 10.1016/S0923-2508(97)85119-4.

853. Brouqui P, Raoult D. Bartonella quintana invades and multiplies within endothelial cells in vitro and in vivo and forms intracellular blebs. Res Microbiol. 1996;147(9):719-31. Epub 1996/11/01. doi: 10.1016/s0923-2508(97)85119-4. PubMed PMID: 9296106.

854. Brown SMA, McDonald V, Denton H, Coombs GH. The use of a new viability assay to determine the susceptibility of Cryptosporidium and Eimeria sporozoites to respiratory inhibitors and extremes of pH. FEMS Microbiology Letters. 1996;142(2-3):203-8. doi: 10.1016/0378-1097(96)00266-2.

855. Caldwell CW, Everett ED, McDonald G, Yesus YW, Roland WE, Huang HM. Apoptosis of gamma/delta T cells in human ehrlichiosis. Am J Clin Pathol. 1996;105(5):640-6. Epub 1996/05/01. doi: 10.1093/ajcp/105.5.640. PubMed PMID: 8623774.

856. Caldwell CW, Everett ED, McDonald G, Yesus YW, Roland WE, Huang HM. Apoptosis of γ/δ T cells in human ehrlichiosis. American Journal of Clinical Pathology. 1996;105(5):640-6. doi: 10.1093/ajcp/105.5.640.

857. Chen SM, Popov VL, Feng HM, Walker DH. Analysis and ultrastructural localization of Ehrlichia chaffeensis proteins with monoclonal antibodies. Am J Trop Med Hyg. 1996;54(4):405-12. Epub 1996/04/01. doi: 10.4269/ajtmh.1996.54.405. PubMed PMID: 8615456.

858. Das P. Cryptosporidium related diarrhoea. Indian Journal of Medical Research. 1996;104(JULY):86-95.

859. Dawson JE. Human ehrlichiosis in the United States. Curr Clin Top Infect Dis. 1996;16:164-71. Epub 1996/01/01. PubMed PMID: 8714253.

860. Dawson JE, Biggie KL, Warner CK, Cookson K, Jenkins S, Levine JF, et al. Polymerase chain reaction evidence of Ehrlichia chaffeensis, an etiologic agent of human ehrlichiosis, in dogs from southeast Virginia. Am J Vet Res. 1996;57(8):1175-9. Epub 1996/08/01. PubMed PMID: 8836370.

861. Dawson JE, Warner CK, Baker V, Ewing SA, Stallknecht DE, Davidson WR, et al. Ehrlichia-like 16S rDNA sequence from wild white-tailed deer (Odocoileus virginianus). J Parasitol. 1996;82(1):52-8. Epub 1996/02/01. PubMed PMID: 8627501.

862. Dawson JE, Warner CK, Standaert S, Olsan JG. The interface between research and the diagnosis of an emerging tick-borne disease, human ehrlichiosis due to Ehrlichia chaffeensis. Archives of Internal Medicine. 1996;156(2):137-42. doi: 10.1001/archinte.156.2.137.

863. Dawson JE, Warner CK, Standaert S, Olson JG. The interface between research and the diagnosis of an emerging tick-borne disease, human ehrlichiosis due to Ehrlichia chaffeensis. Arch Intern Med. 1996;156(2):137-42. Epub 1996/01/22. PubMed PMID: 8546547.

864. Deem SL, Donachie PL, Norval RAI, Mahan SM. Erratum: Colostrum from dams living in a heartwater-endemic area influences calfhood immunity to Cowdria ruminantium (Veterinary Parasitology (1996) 61 (133-144)). Veterinary Parasitology. 1996;65(3-4):317. doi: 10.1016/S0304-4017(96)01048-5.

865. Deem SL, Norval RA, Donachie PL, Mahan SM. Demonstration of vertical transmission of Cowdria ruminantium, the causative agent of heartwater, from cows to their calves. Vet Parasitol. 1996;61(1-2):119-32. Epub 1996/01/01. doi: 10.1016/0304-4017(95)00819-5. PubMed PMID: 8750689.

866. Deem SL, Norval RAI, Yonow T, Peter TF, Mahan SM, Burridge MJ. The epidemiology of heartwater: Establishment and maintenance of endemic stability. Parasitology Today. 1996;12(10):402-5. doi: 10.1016/0169-4758(96)10057-0.

867. Deem SL, Noval RA, Yonow T, Peter TF, Mahan SM, Burridge MJ. The epidemiology of heartwater: Establishment and maintenance of endemic stability. Parasitol Today. 1996;12(10):402-5. Epub 1996/10/01. doi: 10.1016/0169-4758(96)10057-0. PubMed PMID: 15275292.

868. Dumler JS, Bakken JS. Human granulocytic ehrlichiosis in Wisconsin and Minnesota: a frequent infection with the potential for persistence. J Infect Dis. 1996;173(4):1027-30. Epub 1996/04/01. doi: 10.1093/infdis/173.4.1027. PubMed PMID: 8603945.

869. Edelman DC, Dumler JS. Evaluation of an Improved PCR Diagnostic Assay for Human Granulocytic Ehrlichiosis. Mol Diagn. 1996;1(1):41-9. Epub 1996/06/01. doi: 10.1054/modi00100041. PubMed PMID: 10330196.

870. Edelman DC, Stephen Dumler J. Evaluation of an improved PCR diagnostic assay for human granulocytic ehrlichiosis. Molecular Diagnosis. 1996;1(1):41-9. doi: 10.1016/S1084-8592(96)70020-6.

871. Faubert GM. The immune response to Giardia. Parasitology Today. 1996;12(4):140-5. doi: 10.1016/0169-4758(96)10004-1. PubMed PMID: WOS:A1996UB39600005.

872. Fourmaux MN, GarciaReguet N, MercereauPuijalon O, Dubremetz JF. Toxoplasma gondii microneme proteins: Gene cloning and possible function. In: Gross U, editor. Toxoplasma Gondii. Current Topics in Microbiology and Immunology. 2191996. p. 55-8.

873. Fredricks DN, Relman DA. Sequence-based identification of microbial pathogens: a reconsideration of Koch's postulates. Clin Microbiol Rev. 1996;9(1):18-33. Epub 1996/01/01. doi: 10.1128/cmr.9.1.18. PubMed PMID: 8665474; PubMed Central PMCID: PMCPMC172879.

874. Gewirtz AS, Cornbleet PJ, Vugia DJ, Traver C, Niederhuber J, Kolbert CP, et al. Human granulocytic ehrlichiosis: report of a case in Northern California. Clin Infect Dis. 1996;23(3):653-4. Epub 1996/09/01. doi: 10.1093/clinids/23.3.653. PubMed PMID: 8879806.

875. Gewirtz AS, Joanne Cornbleet P, Vugia DJ, Traver C, Niederhuber J, Kolbert CP, et al. Human granulocytic ehrlichiosis: Report of a case in Northern California. Clinical Infectious Diseases. 1996;23(3):653-4. doi: 10.1093/clinids/23.3.653.

876. Gilmour M, Mitchell WJ, Flint HJ. Genetic transfer of lactate-utilizing ability in the rumen bacterium Selenomonas ruminantium. Letters in Applied Microbiology. 1996;22(1):52-6. doi: 10.1111/j.1472-765X.1996.tb01107.x.

877. Goodhead AD. Uveitis in dogs and cats: Guidelines for the practitioner. Journal of the South African Veterinary Association. 1996;67(1):12-9. PubMed Central PMCID: PMCAlcon

Allergan

Burroughs Wellcome

Lederle

Logos Agvet.

878. Goodman JL. Direct cultivation of the causative agent of human granulocytic ehrlichiosis (vol 334, pg 209, 1996). New England Journal of Medicine. 1996;335(5):361-. PubMed PMID: WOS:A1996UY98200030.

879. Goodman JL, Nelson C, Vitale B, Madigan JE, Dumler JS, Kurtti TJ, et al. Direct cultivation of the causative agent of human granulocytic ehrlichiosis. N Engl J Med. 1996;334(4):209-15. Epub 1996/01/25. doi: 10.1056/nejm199601253340401. PubMed PMID: 8531996.

880. Gordon SM. Recognizing and treating new and emerging infections encountered in everyday practice. Cleveland Clinic Journal of Medicine. 1996;63(3):172-8. doi: 10.3949/ccjm.63.3.172. PubMed PMID: WOS:A1996UK40400009.

881. Graczyk TK, Cranfield MR, Fayer R. Evaluation of commercial enzyme immunoassay (EIA) and immunofluorescent antibody (FA) test kits for detection of Cryptosporidium oocysts of species other than Cryptosporidium parvum. Am J Trop Med Hyg. 1996;54(3):274-9. Epub 1996/03/01. doi: 10.4269/ajtmh.1996.54.274. PubMed PMID: 8600765.

882. Graczyk TK, Cranfield MR, Fayer R. Evaluation of commercial enzyme immunoassay (EIA) and immunofluorescent antibody (IFA) test kits for detection of Cryptosporidium oocysts of species other than Cryptosporidium parvum. American Journal of Tropical Medicine and Hygiene. 1996;54(3):274-9. doi: 10.4269/ajtmh.1996.54.274.

883. Greig B, Asanovich KM, Armstrong PJ, Dumler JS. Geographic, clinical, serologic, and molecular evidence of granulocytic ehrlichiosis, a likely zoonotic disease, in Minnesota and Wisconsin dogs. J Clin Microbiol. 1996;34(1):44-8. Epub 1996/01/01. doi: 10.1128/jcm.34.1.44-48.1996. PubMed PMID: 8748270; PubMed Central PMCID: PMCPMC228727.

884. Greig B, Asanovich KM, Jane Armstrong P, Stephen Dumler J. Geographic, clinical, serologic, and molecular evidence of granulocytic ehrlichiosis, a likely zoonotic disease, in minnesota and wisconsin dogs. Journal of Clinical Microbiology. 1996;34(1):44-8. doi: 10.1128/jcm.34.1.44-48.1996.

885. Grencis RK, Cooper ES. Enterobius, Trichuris, Capillaria, and hookworm including Ancylostoma caninum. Gastroenterology Clinics of North America. 1996;25(3):579-+. doi: 10.1016/s0889-8553(05)70264-8. PubMed PMID: WOS:A1996VB53000009.

886. Hong SJ, Woo HC, Chai JY. A human case of Plagiorchis muris (Tanabe, 1922: Digenea) infection in the Republic of Korea: freshwater fish as a possible source of infection. J Parasitol. 1996;82(4):647-9. Epub 1996/08/01. PubMed PMID: 8691378.

887. Horowitz HW, Marks SJ, Weintraub M, Dumler JS. Brachial plexopathy associated with human granulocytic ehrlichiosis. Neurology. 1996;46(4):1026-9. Epub 1996/04/01. doi: 10.1212/wnl.46.4.1026. PubMed PMID: 8780084.

888. KeaneMyers A, Maliszewski CR, Finkelman FD, Nickell SP. Recombinant IL-4 treatment augments resistance to Borrelia burgdorferi infections in both normal susceptible and antibody-deficient susceptible mice. Journal of Immunology. 1996;156(7):2488-94. PubMed PMID: WOS:A1996UB15500023.

889. Keeling PJ, Doolittle WF. A non-canonical genetic code in an early diverging eukaryotic lineage. EMBO Journal. 1996;15(9):2285-90. doi: 10.1002/j.1460-2075.1996.tb00581.x.

890. Kelly PJ, Masanvi N, Cadman HF, Mahan SM, Beati L, Raoult D. Serosurvey for Cowdria ruminantium, Coxiella burnetii, and Spotted fever group rickettsiae in ostriches (Struthio camelus) from Zimbabwe. Avian Dis. 1996;40(2):448-52. Epub 1996/04/01. PubMed PMID: 8790897.

891. Keysary A, Waner T, Rosner M, Warner CK, Dawson JE, Zass R, et al. The first isolation, in vitro propagation, and genetic characterization of Ehrlichia canis in Israel. Vet Parasitol. 1996;62(3-4):331-40. Epub 1996/04/01. doi: 10.1016/0304-4017(95)00866-7. PubMed PMID: 8686178.

892. Khan A, Kakkar A, Lebovics E, Kapur S, Forseter G, Wormser G, et al. Liver biochemical profiles in human granulocytic ehrlichiosis (HGE). Gastroenterology. 1996;110(4):A1232-A. PubMed PMID: WOS:A1996UF73704906.

893. Klein H, Mehlhorn H, Rüger W. In vitro biosynthesis and in vivo processing of the major microneme antigen of Sarcocystis muris cyst merozoites. Parasitology Research. 1996;82(5):468-74. doi: 10.1007/s004360050146.

894. Klein H, Mehlhorn H, Rüger W. Characterization of genomic clones encoding two micrtpneme antigens of Sarcocystis muris (Apicomplexa). Parasitology Research. 1996;82(3):230-7. doi: 10.1007/s004360050101.

895. Klein H, Mehlhorn H, Ruger W. Characterization of genomic clones encoding two microneme antigens of Sarcocystis muris (Apicomplexa). Parasitology Research. 1996;82(3):230-7. doi: 10.1007/s004360050101. PubMed PMID: WOS:A1996TZ33300008.

896. Kostman JR. Laboratory diagnosis of rickettsial diseases. Clinics in Dermatology. 1996;14(3):301-6. doi: 10.1016/0738-081x(96)00016-8. PubMed PMID: WOS:A1996UM28600012.

897. Lally NC, Jenkins MC, Dubey JP. Development of a polymerase chain reaction assay for the diagnosis of neosporosis using the Neospora caninum 14-3-3 gene. Molecular and Biochemical Parasitology. 1996;75(2):169-78. doi: 10.1016/0166-6851(95)02530-8.

898. Lee E, Rikihisa Y. Anti-Ehrlichia chaffeensis antibody enhances proinflammatory cytokine mRNA expression in human monocytes exposed to E-chaffeensis through NF-kappa B activation. Molecular Biology of the Cell. 1996;7:881-. PubMed PMID: WOS:A1996WB01800880.

899. Lee EH, Rikihisa Y. Absence of tumor necrosis factor alpha, interleukin-6 (IL-6), and granulocyte-macrophage colony-stimulating factor expression but presence of IL-1beta, IL-8, and IL-10 expression in human monocytes exposed to viable or killed Ehrlichia chaffeensis. Infect Immun. 1996;64(10):4211-9. Epub 1996/10/01. doi: 10.1128/iai.64.10.4211-4219.1996. PubMed PMID: 8926090; PubMed Central PMCID: PMCPMC174358.

900. Lee EH, Rikihisa Y. Absence of tumor necrosis factor alpha, interleukin-6 (IL-6), and granulocyte-macrophage colony-stimulating factor expression but presence of IL-1β, IL-8, and IL-10 expression in human monocytes exposed to viable or killed Ehrlichia chaffeensis. Infection and Immunity. 1996;64(10):4211-9. doi: 10.1128/iai.64.10.4211-4219.1996.

901. Leng X, Mosier DA, Oberst RD. Differentiation of Cryptosporidium parvum, C. muris, and C. baileyi by PCR-RFLP analysis of the 18s rRNA gene. Veterinary Parasitology. 1996;62(1-2):1-7. doi: 10.1016/0304-4017(95)00863-2.

902. Leng XG, Mosier DA, Oberst RD. Differentiation of Cryptosporidium parvum, C-muris, and C-baileyi by PCR-RFLP analysis of the 18s rRNA gene. Veterinary Parasitology. 1996;62(1-2):1-7. PubMed PMID: WOS:A1996TW64700001.

903. Liebisch A, Schein E, Dorn H, Liebisch G. Prevention of infestation with ticks and fleas with the dog collar KILTIX. Praktische Tierarzt. 1996;77(6):493-&. PubMed PMID: WOS:A1996UT87700003.

904. Liveris D. Lyme disease and ehrlichiosis. Clin Podiatr Med Surg. 1996;13(4):595-634. Epub 1996/10/01. PubMed PMID: 8902334.

905. Lockhart JM, Davidson WR, Stallknecht DE, Dawson JE. Site-Specific Geographic Association between Amblyomma americanum (Acari: Ixodidae) Infestations and Ehrlichia chaffeensis-Reactive (Rickettsiales: Ehrlichieae) Antibodies in White-Tailed Deer. Journal of Medical Entomology. 1996;33(1):153-8. doi: 10.1093/jmedent/33.1.153.

906. Lockhart JM, Davidson WR, Stallknecht DE, Dawson JE. Site-specific geographic association between Amblyomma americanum (Acari: Ixodidae) infestations and Ehrlichia chaffeensis reactive (Rickettsiales: Ehrlichieae) antibodies in white-tailed deer. Journal of Medical Entomology. 1996;33(1):153-8. doi: 10.1093/jmedent/33.1.153. PubMed PMID: WOS:A1996TP03900024.

907. Madigan JE, Barlough JE, Dumler JS, Schankman NS, DeRock E. Equine granulocytic ehrlichiosis in Connecticut caused by an agent resembling the human granulocytotropic ehrlichia. J Clin Microbiol. 1996;34(2):434-5. Epub 1996/02/01. doi: 10.1128/jcm.34.2.434-435.1996. PubMed PMID: 8789032; PubMed Central PMCID: PMCPMC228814.

908. Mahan SM, Sileghem M, Smith GE, Byrom B. Neutralization of bovine Concanavalin-A T cell supernatant-mediated anti-Cowdria ruminantium activity with antibodies specific to Interferon gamma but not to tumor necrosis factor. Parasite Immunology. 1996;18(6):317-24. doi: 10.1046/j.1365-3024.1996.d01-106.x.

909. Majewska AC, Gustowska L. Comparative studies of experimental giardiosis in Mongolian gerbils .3. Changes in small intestine induced with human and zoo animal Giardia isolates. Acta Parasitologica. 1996;41(3):128-35. PubMed PMID: WOS:A1996VW13100006.

910. Martin SA, Park CM. Effect of extracellular hydrogen on organic acid utilization by the ruminal bacterium Selenomonas ruminantium. Current Microbiology. 1996;32(6):327-31. doi: 10.1007/s002849900058.

911. Maurin M, Raoult D. Optimum treatment of intracellular infection. Drugs. 1996;52(1):45-59. doi: 10.2165/00003495-199652010-00004.

912. Mazzella FM, Roman A, Perez A. A case of concurrent presentation of human ehrlichiosis and Lyme disease in Connecticut. Conn Med. 1996;60(9):515-9. Epub 1996/09/01. PubMed PMID: 8908776.

913. McDade JE, Anderson BE. Molecular epidemiology: Applications of nucleic acid amplification and sequence analysis. Epidemiologic Reviews. 1996;18(1):90-7. doi: 10.1093/oxfordjournals.epirev.a017919. PubMed PMID: WOS:A1996VF96100008.

914. McRoberts KM, Meloni BP, Morgan UM, Marano R, Binz N, Erlandsen SL, et al. Morphological and molecular characterization of Giardia isolated from the straw-necked ibis (Threskiornis spinicollis) in Western Australia. Journal of Parasitology. 1996;82(5):711-8. doi: 10.2307/3283880. PubMed PMID: WOS:A1996VN95900006.

915. Meeusen ENT. Rational design of nematode vaccines; Natural antigens. International Journal for Parasitology. 1996;26(8-9):813-8. PubMed PMID: WOS:A1996VR27700003.

916. Mitchell PD, Reed KD, Hofkes JM. Immunoserologic evidence of coinfection with Borrelia burgdorferi, Babesia microti, and human granulocytic Ehrlichia species in residents of Wisconsin and Minnesota. J Clin Microbiol. 1996;34(3):724-7. Epub 1996/03/01. doi: 10.1128/jcm.34.3.724-727.1996. PubMed PMID: 8904446; PubMed Central PMCID: PMCPMC228878.

917. Mounzer KC, Dinubile MJ. Ehrlichial infections. Clin Dermatol. 1996;14(3):289-93. Epub 1996/05/01. doi: 10.1016/0738-081x(96)00014-4. PubMed PMID: 8727132.

918. Munderloh UG, Madigan JE, Dumler JS, Goodman JL, Hayes SF, Barlough JE, et al. Isolation of the equine granulocytic ehrlichiosis agent, Ehrlichia equi, in tick cell culture. Journal of Clinical Microbiology. 1996;34(3):664-70. doi: 10.1128/jcm.34.3.664-670.1996. PubMed PMID: WOS:A1996UD13700035.

919. Munderloh UG, Madigan JE, Stephen Dumler J, Goodman JL, Hayes SF, Barlough JE, et al. Isolation of the equine granulocytic ehrlichiosis agent, Ehrlichia equi, in tick cell culture. Journal of Clinical Microbiology. 1996;34(3):664-70. doi: 10.1128/jcm.34.3.664-670.1996.

920. Musoke AJ, Palmer GH, McElwain TF, Nene V, Imckeever D. Prospects for subunit vaccines against tick-borne diseases. British Veterinary Journal. 1996;152(6):621-39. doi: 10.1016/S0007-1935(96)80117-5.

921. Neitz AWH, Yunker CE. Amino acid and protein depletion in medium of cell cultures infected with Cowdria ruminantium. 1996. p. 24-34.

922. Newbold CJ, Ushida K, Morvan B, Fonty G, Jouany JP. The role of ciliate protozoa in the lysis of methanogenic archaea in rumen fluid. Letters in Applied Microbiology. 1996;23(6):421-5. doi: 10.1111/j.1472-765X.1996.tb01350.x.

923. Nuti M. [Ehrlichiosis: a new emerging disease]. Ann Ig. 1996;8(6):681-5. Epub 1996/11/01. PubMed PMID: 9312236.

924. Nuti M, Russino F, Grazioli D, Rombola P, Macri G, Lillini E. Anti-Ehrlichia antibodies in high-risk subjects living in the piedmont of the Veneto region. Microbiologia Medica. 1996;11(4):492-5.

925. Osborne J, Hunter SJ, Devaney E. Anti-interleukin-4 modulation of the Th2 polarized response to the parasitic nematode Brugia pahangi. Infection and Immunity. 1996;64(9):3461-6. doi: 10.1128/iai.64.9.3461-3466.1996. PubMed PMID: WOS:A1996VE44300004.

926. Perez M, Rikihisa Y, Wen B. Ehrlichia canis-like agent isolated from a man in Venezuela: antigenic and genetic characterization. J Clin Microbiol. 1996;34(9):2133-9. Epub 1996/09/01. doi: 10.1128/jcm.34.9.2133-2139.1996. PubMed PMID: 8862572; PubMed Central PMCID: PMCPMC229204.

927. Perez M, Rikihisa Y, Wen BH. Ehrlichia canis-like agent isolated from a man in Venezuela: Antigenic and genetic characterization. Journal of Clinical Microbiology. 1996;34(9):2133-9. doi: 10.1128/jcm.34.9.2133-2139.1996. PubMed PMID: WOS:A1996VD33500016.

928. Pinner RW. Addressing the challenges of emerging infectious disease. Am J Med Sci. 1996;311(1):3-8. Epub 1996/01/01. doi: 10.1097/00000441-199601000-00002. PubMed PMID: 8571983.

929. Pinner RW. Addressing the challenges of emerging infectious diseases. American Journal of the Medical Sciences. 1996;311(1):3-8. doi: 10.1097/00000441-199601000-00002.

930. Ramzan NN, Gross JB, Kolbert C, Persing DH. Hepatitis in patients with human granulocytic ehrlichiosis. Gastroenterology. 1996;110(4):A1302-A. PubMed PMID: WOS:A1996UF73705186.

931. Ratnasamy N, Everett ED, Roland WE, McDonald G, Caldwell CW. Central nervous system manifestations of human ehrlichiosis. Clin Infect Dis. 1996;23(2):314-9. Epub 1996/08/01. doi: 10.1093/clinids/23.2.314. PubMed PMID: 8842270.

932. Redondo MC. Identification of Ehrlichia species in blood smear. Infectious Diseases in Clinical Practice. 1996;5(9):555-7. doi: 10.1097/00019048-199612000-00011.

933. Relman DA, Schmidt TM, Gajadhar A, Sogin M, Cross J, Yoder K, et al. Molecular phylogenetic analysis of Cyclospora, the human intestinal pathogen, suggests that it is closely related to Eimeria species. Journal of Infectious Diseases. 1996;173(2):440-5. PubMed PMID: WOS:A1996TT66500022.

934. Rex JH, Vanek NN, Kazi S, Cepero NM, Tang S. Human ehrlichiosis causing left ventricular dilatation and dysfunction. Clinical Infectious Diseases. 1996;22(2):386-7. doi: 10.1093/clinids/22.2.386.

935. Richter Jr PJ, Kimsey RB, Madigan JE, Barlough JE, Dumler JS, Brooks DL. Ixodes pacificus (Acari: Ixodidae) as a Vector of Ehrlichia equi (Rickettsiales: Ehrlichieae). Journal of Medical Entomology. 1996;33(1):1-5. doi: 10.1093/jmedent/33.1.1.

936. Richter PJ, Kimsey RB, Madigan JE, Barlough JE, Dumler JS, Brooks DL. Ixodes pacificus (Acari: Ixodidae) as a vector of Ehrlichia equi (Rickettsiales: Ehrlichieae). Journal of Medical Entomology. 1996;33(1):1-5. PubMed PMID: WOS:A1996TP03900001.

937. Ricke SC, Martin SA, Nisbet DJ. Ecology, metabolism, and genetics of ruminal selenomonads. Critical Reviews in Microbiology. 1996;22(1):27-65.

938. Ricke SC, Schaefer DM. Growth and fermentation responses of Selenomonas ruminantium to limiting and non-limiting concentrations of ammonium chloride. Appl Microbiol Biotechnol. 1996;46(2):169-75. Epub 1996/09/01. doi: 10.1007/s002530050800. PubMed PMID: 8987647.

939. Ricke SC, Schaefer DM. Glucose fermentation and growth of Selenomonas sputigena on a minimal medium. Journal of Rapid Methods and Automation in Microbiology. 1996;4(3):173-81. doi: 10.1111/j.1745-4581.1996.tb00121.x.

940. Roman Reddy G, Sulsona CR, Harrison RH, Mahan SM, Burridge MJ, Barbet AF. Sequence heterogeneity of the major antigenic protein 1 genes from Cowdria ruminantium isolates from different geographical areas. Clinical and Diagnostic Laboratory Immunology. 1996;3(4):417-22. doi: 10.1128/cdli.3.4.417-422.1996.

941. Rosenblatt J, Sloan L, Magera J, Finkel M, Dattwyler R, Persing D. Detection of serum antibodies to the agent of human granulocytic ehrlichiosis using an indirect fluorescent antibody procedure. Clinical Infectious Diseases. 1996;23(4):195-. PubMed PMID: WOS:A1996VN24600241.

942. Santiago EB, Garcia PM. Emerging bacteria causing infectious diseases. Revista Clinica Espanola. 1996;196:59-65. PubMed PMID: WOS:A1996VN13100016.

943. Schaffner W, Standaert SM. Ehrlichiosis--in pursuit of an emerging infection. N Engl J Med. 1996;334(4):262-3. Epub 1996/01/25. doi: 10.1056/nejm199601253340410. PubMed PMID: 8532005.

944. Schaffner W, Standaert SM. Ehrlichiosis - In pursuit of an emerging infection. New England Journal of Medicine. 1996;334(4):262-3. doi: 10.1056/NEJM199601253340410.

945. Schulze GE, Jacobs RF. Human monocytic ehrlichiosis in children. Clinical Infectious Diseases. 1996;23(4):344-. PubMed PMID: WOS:A1996VN24600390.

946. Schwartz BS, Sanchez JL, Sanders ML, DeFraites RF. Tick avoidance behaviors associated with a decreased risk of anti-tick salivary gland protein antibody seropositivity in military personnel exposed to Amblyomma americanum in Arkansas. Am J Trop Med Hyg. 1996;55(4):410-6. Epub 1996/10/01. doi: 10.4269/ajtmh.1996.55.410. PubMed PMID: 8916798.

947. Shea KW, Calio AJ, Klein NC, Cunha BA. Ehrlichia equi infection associated with rhabdomyolysis. Clin Infect Dis. 1996;22(3):605. Epub 1996/03/01. doi: 10.1093/clinids/22.3.605-a. PubMed PMID: 8853010.

948. Shea KW, Calio AJ, Klein NC, Cunha BA. Ehrlichia equi infection associated with rhabdomyolysis [5]. Clinical Infectious Diseases. 1996;22(3):605. doi: 10.1093/clinids/22.3.605-a.

949. Stith DM. Diagnosing ehrlichiosis. Ann Intern Med. 1996;124(9):854-5. Epub 1996/05/01. doi: 10.7326/0003-4819-124-9-199605010-00012. PubMed PMID: 8610957.

950. Taghi-Kilani R, Gyürék LL, Millard PJ, Finch GR, Belosevic M. Nucleic acid stains as indicators of Giardia muris viability following cyst inactivation. International Journal for Parasitology. 1996;26(6):637-46. doi: 10.1016/0020-7519(96)00033-1.

951. Tami I, Martinez JI, Tami M, Redondo MC, Finol H, Simonovis N. Identification of Ehrlichia species in blood smear. Infectious Diseases in Clinical Practice. 1996;5(9):555-7. doi: 10.1097/00019048-199612000-00011. PubMed PMID: WOS:A1996VZ86900012.

952. Telford Iii SR, Dawson JE. Persistent infection of C3H/HeJ mice by Ehrlichia chaffeensis. Veterinary Microbiology. 1996;52(1-2):103-12. doi: 10.1016/0378-1135(96)00064-8.

953. Telford Iii SR, Dawson JE, Katavolos P, Warner CK, Kolbert CP, Persing DH. Perpetuation of the agent of human granulocytic ehrlichiosis in a deer tick-rodent cycle. Proceedings of the National Academy of Sciences of the United States of America. 1996;93(12):6209-14. doi: 10.1073/pnas.93.12.6209.

954. Telford SR, 3rd, Dawson JE. Persistent infection of C3H/HeJ mice by Ehrlichia chaffeensis. Vet Microbiol. 1996;52(1-2):103-12. Epub 1996/09/01. doi: 10.1016/0378-1135(96)00064-8. PubMed PMID: 8914255.

955. Telford SR, 3rd, Dawson JE, Katavolos P, Warner CK, Kolbert CP, Persing DH. Perpetuation of the agent of human granulocytic ehrlichiosis in a deer tick-rodent cycle. Proc Natl Acad Sci U S A. 1996;93(12):6209-14. Epub 1996/06/11. doi: 10.1073/pnas.93.12.6209. PubMed PMID: 8650245; PubMed Central PMCID: PMCPMC39215.

956. Tomley FM, Bumstead JM, Billington KJ, Dunn PPJ. Molecular cloning and characterization of a novel acidic microneme protein (Etmic-2) from the apicomplexan protozoan parasite, Eimeria tenella. Molecular and Biochemical Parasitology. 1996;79(2):195-206. doi: 10.1016/0166-6851(96)02662-x. PubMed PMID: WOS:A1996VA64100006.

957. Tournay C, Courtoy PJ, Marodi L, Totté P, Werenne J, Jacquet A, et al. Uptake of recombinant myeloperoxidase, free or fused to Fc gamma, by macrophages enhances killing activity toward micro-organisms. DNA Cell Biol. 1996;15(8):617-24. Epub 1996/08/01. doi: 10.1089/dna.1996.15.617. PubMed PMID: 8769563.

958. Van Vliet AHM, Van Der Zeijst BAM, Camus E, Mahan SM, Martinez D, Jongejan F. Recombinant expression and use in serology of a specific fragment from the Cowdria ruminantium MAP1 protein. 1996. p. 35-45.

959. Vugia DJ, Kramer VL. Human babesiosis and ehrlichioses--emerging tick-borne diseases. West J Med. 1996;165(4):224. Epub 1996/10/01. PubMed PMID: 8987430; PubMed Central PMCID: PMCPMC1303752.

960. Vugia DJ, Kramer VL. Human babesiosis and ehrlichioses - Emerging tick-borne diseases. Western Journal of Medicine. 1996;165(4):224.

961. Waladde SM, Young AS, Morzaria SP. Artificial feeding of ixodid ticks. Parasitol Today. 1996;12(7):272-8. Epub 1996/07/01. doi: 10.1016/0169-4758(96)10027-2. PubMed PMID: 15275192.

962. Walker DH. Rickettsiae. In: Baron S, editor. Medical Microbiology. Galveston (TX): University of Texas Medical Branch at Galveston

Copyright © 1996, The University of Texas Medical Branch at Galveston.; 1996.

963. Walker DH. Human ehrlichiosis: more trouble from ticks. Hosp Pract (1995). 1996;31(4):47-57. Epub 1996/04/15. PubMed PMID: 8609191.

964. Walker DH. Clinical experience. Human ehrlichiosis: more trouble from ticks. Hospital Practice. 1996;31(4):47-168. PubMed PMID: 107353888. Language: English. Entry Date: 19971201. Revision Date: 20150711. Publication Type: Journal Article.

965. Walker DH, Barbour AG, Oliver JH, Lane RS, Dumler JS, Dennis DT, et al. Emerging bacterial zoonotic and vector-borne diseases. Ecological and epidemiological factors. Jama. 1996;275(6):463-9. Epub 1996/02/14. PubMed PMID: 8627968.

966. Walker DH, Barbour AG, Oliver JH, Lane RS, Dumler JS, Dennis DT, et al. Emerging bacterial zoonotic and vector-borne diseases - Ecological and epidemiological factors. Jama-Journal of the American Medical Association. 1996;275(6):463-9. doi: 10.1001/jama.275.6.463. PubMed PMID: WOS:A1996TU63800035.

967. Walker DH, Dumler JS. Emergence of the ehrlichioses as human health problems. Emerg Infect Dis. 1996;2(1):18-29. Epub 1996/01/01. doi: 10.3201/eid0201.960102. PubMed PMID: 8903194; PubMed Central PMCID: PMCPMC2639805.

968. Weinstein RS. Human ehrlichiosis. Am Fam Physician. 1996;54(6):1971-6. Epub 1996/11/01. PubMed PMID: 8900357.

969. Wen B, Rikihisa Y, Yamamoto S, Kawabata N, Fuerst PA. Characterization of the SF agent, an Ehrlichia sp. isolated from the fluke Stellantchasmus falcatus, by 16S rRNA base sequence, serological, and morphological analyses. Int J Syst Bacteriol. 1996;46(1):149-54. Epub 1996/01/01. doi: 10.1099/00207713-46-1-149. PubMed PMID: 8573488.

970. Wen BH, Rikihisa Y, Yamamoto S, Kawabata N, Fuerst PA. Characterization of the SF agent, an Ehrlichia sp isolated from the fluke Stellantchasmus falcatus, by 16S rRNA base sequence, serological, and morphological analyses. International Journal of Systematic Bacteriology. 1996;46(1):149-54. doi: 10.1099/00207713-46-1-149. PubMed PMID: WOS:A1996TP73800021.

971. Wilske B, Fingerle V. Ehrlichiosis, a New Tick-borne Infectious Disease. Munchener Medizinische Wochenschrift. 1996;138(12):202-4.

972. Wong S, Grady LJ. Ehrlichia infection as a cause of severe respiratory distress. N Engl J Med. 1996;334(4):273. Epub 1996/01/25. doi: 10.1056/nejm199601253340418. PubMed PMID: 8532017.

973. Wong S, Grady LJ. Ehrlichia infection as a cause of severe respiratory distress [6]. New England Journal of Medicine. 1996;334(4):273. doi: 10.1056/NEJM199601253340418.

974. Wormser GP, Horowitz HW, Dumler JS, Schwartz I, Aguero-Rosenfeld M. False-positive Lyme disease serology in human granulocytic ehrlichiosis. Lancet. 1996;347(9006):981-2. Epub 1996/04/06. doi: 10.1016/s0140-6736(96)91475-0. PubMed PMID: 8598802.

975. Wormser GP, Horowitz HW, Dumler JS, Schwartz I, Aguero-Rosenfeld M. False-positive Lyme disease serology in human granulocytic ehrlichiosis [29]. Lancet. 1996;347(9006):981-2. doi: 10.1016/S0140-6736(96)91475-0.

976. Wormser GP, Horowitz HW, Dumler JS, Schwartz I, Aguero-Rosenfeld M. False-positive Lyme disease serology in human granulocytic ehrlichiosis. Philadelphia, Pennsylvania: Lancet; 1996. p. 981-2.

977. Wormser GP, Horowitz HW, Dumler JS, Schwartz I, AgueroRosenfeld M. False-positive Lyme disease serology in human granulocytic ehrlichiosis. Lancet. 1996;347(9006):981-2. doi: 10.1016/s0140-6736(96)91475-0. PubMed PMID: WOS:A1996UD59100076.

978. Yu XJ, Crocquet-Valdes P, Cullman LC, Walker DH. The recombinant 120-kilodalton protein of Ehrlichia chaffeensis, a potential diagnostic tool. J Clin Microbiol. 1996;34(11):2853-5. Epub 1996/11/01. doi: 10.1128/jcm.34.11.2853-2855.1996. PubMed PMID: 8897200; PubMed Central PMCID: PMCPMC229421.

979. Yu XJ, CrocquetValdes P, Cullman LC, Walker DH. The recombinant 120-kilodalton protein of Ehrlichia chaffeensis, a potential diagnostic tool. Journal of Clinical Microbiology. 1996;34(11):2853-5. doi: 10.1128/jcm.34.11.2853-2855.1996. PubMed PMID: WOS:A1996VM55600045.

980. Adachi JA, Grimm EM, Johnson P, Uthman M, Kaplan B, Rakita RM. Human granulocytic ehrlichiosis in a renal transplant patient: case report and review of the literature. Transplantation. 1997;64(8):1139-42. Epub 1997/11/14. doi: 10.1097/00007890-199710270-00010. PubMed PMID: 9355830.

981. Adachi JA, Grimm EM, Johnson P, Uthman M, Kaplan B, Rakita RM. Human granulocytic ehrlichiosis in a renal transplant patient - Case report and review of the literature. Transplantation. 1997;64(8):1139-42. doi: 10.1097/00007890-199710270-00010. PubMed PMID: WOS:A1997YE23300010.

982. AgueroRosenfeld ME, Wormser GP, Dumler JS. Human granulocytic ehrlichiosis: A cardiac risk factor? Response. Annals of Internal Medicine. 1997;127(1):90-. doi: 10.7326/0003-4819-127-1-199707010-00024. PubMed PMID: WOS:A1997XG91400023.

983. Andras S, Anna M. Infectology at the end of the 20th century - Human ehrlichiosis. Lege Artis Medicine. 1997;7(11):692-6.

984. Arraga De Alvarado CM, Parra M ODC, Palmar M, Chango RE, Alvarado A MC. Ehrlichia platys: Antigen processing and use of the indirect fluorescent antibody test (IFA) in canines and human. Revista Cientifica de la Facultad de Ciencias Veterinarias de la Universidad del Zulia. 1997;7(2):99-109.

985. Asanovich KM, Bakken JS, Madigan JE, Aguero-Rosenfeld M, Wormser GP, Dumler JS. Antigenic diversity of granulocytic Ehrlichia isolates from humans in Wisconsin and New York and a horse in California. J Infect Dis. 1997;176(4):1029-34. Epub 1997/10/23. doi: 10.1086/516529. PubMed PMID: 9333162.

986. Asanovich KM, Bakken JS, Madigan JE, AgueroRosenfeld M, Wormser GP, Dumler JS. Antigenic diversity of granulocytic Ehrlichia isolates from humans in Wisconsin and New York and a horse in California. Journal of Infectious Diseases. 1997;176(4):1029-34. doi: 10.1086/516529. PubMed PMID: WOS:A1997XZ46500023.

987. Bakken JS, Dumler JS. Risk for acquiring human granulocytic ehrlichiosis: Exposure to deer blood or deer ticks? [2]. Clinical Infectious Diseases. 1997;24(3):531-3. doi: 10.1093/clinids/24.3.531.

988. Bakken JS, Dumler JS. Risk for acquiring human granulocytic ehrlichiosis: Exposure to deer blood or deer ticks? Reply. Clinical Infectious Diseases. 1997;24(3):532-3. doi: 10.1093/clinids/24.3.532. PubMed PMID: WOS:A1997WK38200054.

989. Bakken JS, Krueth J, Riddell D, Tilden RL, Asanovich K, Dumler JS. The effect of doxycycline on blood counts in patients infected with human granulocytic ehrlichiosis (HGE). Clinical Infectious Diseases. 1997;25(2):368.

990. Bakken JS, Krueth J, Tilden RL, Asanovich K, Walls J, Dumler JS. Duration of IFA urologic response in humans infected with the agent of Human Granulocytic Ehrlichiosis (HGE). Clinical Infectious Diseases. 1997;25(2):368.

991. Barlough JE, Madigan JE, Kramer VL, Clover JR, Hui LT, Webb JP, et al. Ehrlichia phagocytophila genogroup rickettsiae in ixodid ticks from California collected in 1995 and 1996. Journal of Clinical Microbiology. 1997;35(8):2018-21. doi: 10.1128/jcm.35.8.2018-2021.1997.

992. Barlough JE, Madigan JE, Turoff DR, Clover JR, Shelly SM, Dumler S. An Ehrlichia strain from a llama (Lama glama) and llama-associated ticks (Ixodes pacificus). Journal of Clinical Microbiology. 1997;35(4):1005-7. doi: 10.1128/jcm.35.4.1005-1007.1997. PubMed PMID: WOS:A1997WP40500044.

993. Barnewall RE, Rikihisa Y, Lee EH. Ehrlichia chaffeensis inclusions are early endosomes which selectively accumulate transferrin receptor. Infect Immun. 1997;65(4):1455-61. Epub 1997/04/01. doi: 10.1128/iai.65.4.1455-1461.1997. PubMed PMID: 9119487; PubMed Central PMCID: PMCPMC175153.

994. Behl R, Klein MB, Dandelet LA, Bach RR, Goodman JL, Key NS. Induction of tissue factor procoagulant activity in HL-60 cells inocculated by the agent of human granulocytic ehrlichiosis. Blood. 1997;90(10):2002-. PubMed PMID: WOS:A1997YG42401998.

995. Belay N, Boopathy R, Voskuilen G. Anaerobic Transformation of Furfural by Methanococcus deltae (Delta)LH. Appl Environ Microbiol. 1997;63(5):2092-4. Epub 1997/05/01. doi: 10.1128/aem.63.5.2092-2094.1997. PubMed PMID: 16535618; PubMed Central PMCID: PMCPMC1389173.

996. Belay N, Boopathy R, Voskuilen G. Anaerobic transformation of furfural by Methanococcus deltae ΔLH. Applied and Environmental Microbiology. 1997;63(5):2092-4. doi: 10.1128/aem.63.5.2092-2094.1997.

997. Belongia EA, Chyou PH, Reed KD. Lyme disease and human granulocytic ehrlichiosis. Am J Clin Pathol. 1997;108(4):479-80; author reply 82-3. Epub 1997/10/10. PubMed PMID: 9322603.

998. Belongia EA, Reed KD, Mitchell PD, Kolbert CP, Persing DH, Gill JS, et al. Prevalence of granulocytic Ehrlichia infection among white-tailed deer in Wisconsin. Journal of Clinical Microbiology. 1997;35(6):1465-8. doi: 10.1128/jcm.35.6.1465-1468.1997.

999. Brayton KA, Fehrsen J, De Villiers EP, Van Kleef M, Allsopp BA. Construction and initial analysis of a representative λZAPII expression library of the intracellular rickettsia Cowdria ruminantium: Cloning of map1 and three other Cowdria genes. Veterinary Parasitology. 1997;72(2):185-99. doi: 10.1016/S0304-4017(97)00020-4.

1000. Brouqui P. [Tropical animal and human rickettsial infections]. Med Trop (Mars). 1997;57(3 Suppl):23-7. Epub 1997/01/01. PubMed PMID: 9513175.

1001. Brouqui P. Human ehrlichiose, an emerging infectious disease. Medecine et Maladies Infectieuses. 1997;27(3):256-66.

1002. Brouqui P. Human ehrlichiosis, an emerging infectious disease. Medecine Et Maladies Infectieuses. 1997;27(3):256-66. doi: 10.1016/s0399-077x(97)80162-x. PubMed PMID: WOS:A1997WW41500001.

1003. Brown TJ, Clarke JK, Ionas G, Farrant KJ, McLenachan PA. Molecular (PCR) differentiation of Giardia muris and Giardia intestinalis. International Journal of Environmental Health Research. 1997;7(1):63-. PubMed PMID: 106058547. Language: English. Entry Date: 20070101. Revision Date: 20150711. Publication Type: Journal Article. Journal Subset: Biomedical.

1004. Carruthers VB, Sibley LD. Sequential protein secretion from three distinct organelles of Toxoplasma gondii accompanies invasion of human fibroblasts. European Journal of Cell Biology. 1997;73(2):114-23. PubMed PMID: WOS:A1997XC56900004.

1005. Chen SM, Cullman LC, Walker DH. Western immunoblotting analysis of the antibody responses of patients with human monocytotropic ehrlichiosis to different strains of Ehrlichia chaffeensis and Ehrlichia canis. Clin Diagn Lab Immunol. 1997;4(6):731-5. Epub 1998/01/10. doi: 10.1128/cdli.4.6.731-735.1997. PubMed PMID: 9384299; PubMed Central PMCID: PMCPMC170650.

1006. Chen SM, Yu XJ, Popov VL, Westerman EL, Hamilton FG, Walker DH. Genetic and antigenic diversity of Ehrlichia chaffeensis: comparative analysis of a novel human strain from Oklahoma and previously isolated strains. J Infect Dis. 1997;175(4):856-63. Epub 1997/04/01. doi: 10.1086/513982. PubMed PMID: 9086141.

1007. Christmann D, Hansmann Y, Staub-Schmidt T. Community acquired infectious diseases. Medecine et Maladies Infectieuses. 1997;27(1):14-7.

1008. Cinco M, Padovan D, Murgia R, Maroli M, Frusteri L, Heldtander M, et al. Coexistence of Ehrlichia phagocytophila and Borrelia burgdorferi sensu lato in Ixodes ricinus ticks from Italy as determined by 16S rRNA gene sequencing. J Clin Microbiol. 1997;35(12):3365-6. Epub 1997/12/17. doi: 10.1128/jcm.35.12.3365-3366.1997. PubMed PMID: 9399564; PubMed Central PMCID: PMCPMC230192.

1009. Cohen MR. Tick-borne diseases in the United States. South Med J. 1997;90(6):663. Epub 1997/06/01. doi: 10.1097/00007611-199706000-00019. PubMed PMID: 9191750.

1010. Daniels TJ, Falco RC, Schwartz I, Varde S, Robbins RG. Deer ticks (Ixodes scapularis) and the agents of Lyme disease and human granulocytic ehrlichiosis in a New York City park. Emerg Infect Dis. 1997;3(3):353-5. Epub 1997/07/01. doi: 10.3201/eid0303.970312. PubMed PMID: 9284380; PubMed Central PMCID: PMCPMC2627640.

1011. Dawson JE, Warner CK, Ewing SA, Telford SR, Corstvet RE, Brennan R, et al. Fingerprinting of Ehrlichia species by repetitive element polymerase chain reaction. Am J Trop Med Hyg. 1997;57(1):109-14. Epub 1997/07/01. doi: 10.4269/ajtmh.1997.57.109. PubMed PMID: 9242329.

1012. De Pena HFJ, Kasai N, Gennari SM. Cryptosporidium muris in dairy cattle in Brazil. Veterinary Parasitology. 1997;73(3-4):353-5. doi: 10.1016/S0304-4017(97)00093-9.

1013. deAlvarado CMA, Parra OD, Palmar M, Chango RE, Alvarado MC. Ehrlichia platys: Antigen processing and use of the indirect fluorescent antibody test (IFA) in canines and human. Revista Cientifica-Facultad De Ciencias Veterinarias. 1997;7(2):99-109. PubMed PMID: WOS:A1997XU47100006.

1014. Des Vignes F, Fish D. Transmission of the agent of human granulocytic ehrlichiosis by host-seeking Ixodus scapularis (Acari:Ixodidae) in southern New York state. J Med Entomol. 1997;34(4):379-82. Epub 1997/07/01. doi: 10.1093/jmedent/34.4.379. PubMed PMID: 9220669.

1015. DesVignes F, Fish D. Transmission of the agent of human granulocytic ehrlichiosis by host-seeking Ixodus scapularis (Acari: Ixodidae) in southern New York state. Journal of Medical Entomology. 1997;34(4):379-82. PubMed PMID: WOS:A1997XK08000001.

1016. Duffy J, Pittlekow MR, Kolbert CP, Rutledge BJ, Persing DH. Coinfection with Borrelia burgdorferi and the agent of human granulocytic ehrlichiosis. Lancet. 1997;349(9049):399. Epub 1997/02/08. doi: 10.1016/s0140-6736(97)80017-7. PubMed PMID: 9033471.

1017. Dumler JS. Is human granulocytic ehrlichiosis a new Lyme disease? Review and comparison of clinical, laboratory, epidemiological, and some biological features. Clin Infect Dis. 1997;25 Suppl 1:S43-7. Epub 1997/07/01. doi: 10.1086/516164. PubMed PMID: 9233663.

1018. Dumler JS, editor Is human granulocytic ehrlichiosis a new Lyme disease? Review and comparison of clinical, laboratory, epidemiological, and some biological features. Clinical Infectious Diseases; 1997.

1019. Dumler JS, Dotevall L, Gustafson R, Granström M. A population-based seroepidemiologic study of human granulocytic ehrlichiosis and Lyme borreliosis on the west coast of Sweden. J Infect Dis. 1997;175(3):720-2. Epub 1997/03/01. doi: 10.1093/infdis/175.3.720. PubMed PMID: 9041353.

1020. Dumler JS, Dotevall L, Gustafson R, Granström MA. A population-based seroepidemiologic study of human granulocytic ehrlichiosis and Lyme borreliosis on the west coast of Sweden. Journal of Infectious Diseases. 1997;175(3):720-2. doi: 10.1093/infdis/175.3.720.

1021. Eliasson I, Bjöersdorff A. [Does the infectious disease, human ehrlichiosis, exist in Sweden? Ticks get a hold of new zoonoses]. Lakartidningen. 1997;94(40):3487-8. Epub 1997/12/31. PubMed PMID: 9411085.

1022. Eliasson I, Bjöersdorff A. Does human ehrlichiosis exist in Sweden? New tick-borne zoonoses. Lakartidningen. 1997;(40):3487-8.

1023. Else KJ, Betts CJ. Antibody isotype responses to Trichuris infection in humanized SCID mice. Parasite Immunol. 1997;19(11):485-91. Epub 1998/01/15. doi: 10.1046/j.1365-3024.1997.d01-161.x. PubMed PMID: 9427995.

1024. Evans JD, Martin SA. Factors affecting lactate and malate utilization by Selenomonas ruminantium. Applied and Environmental Microbiology. 1997;63(12):4853-8. doi: 10.1128/aem.63.12.4853-4858.1997.

1025. Ewing SA, Dawson JE, Mathew JS, Barker RW, Pratt KW, Telford Iii SR. Attempted transmission of human granulocytotropic Ehrlichia (HGE) by Amblyomma americanum and Amblyomma maculatum. Veterinary Parasitology. 1997;70(1-3):183-90. doi: 10.1016/S0304-4017(96)01157-0.

1026. Ewing SA, Dawson JE, Mathew JS, Barker RW, Pratt KW, Telford SR, 3rd. Attempted transmission of human granulocytotropic Ehrlichia (HGE) by Amblyomma americanum and Amblyomma maculatum. Vet Parasitol. 1997;70(1-3):183-90. Epub 1997/06/01. doi: 10.1016/s0304-4017(96)01157-0. PubMed PMID: 9195722.

1027. Ewing SA, Dawson JE, Panciera RJ, Mathew JS, Pratt KW, Katavolos P, et al. Dogs infected with a human granulocytotropic Ehrlichia spp. (Rickettsiales: Ehrlichieae). Journal of medical entomology. 1997;34(6):710-8.

1028. Ewing SA, Dawson JE, Panciera RJ, Mathew JS, Pratt KW, Katavolos P, et al. Dogs Infected with a Human Granulocytotropic Ehrlichia spp. (Rickettsiales: Ehrlichieae). Journal of Medical Entomology. 1997;34(6):710-8. doi: 10.1093/jmedent/34.6.710.

1029. Ewing SA, Dawson JE, Panciera RJ, Mathew JS, Pratt KW, Katavolos P, et al. Dogs infected with a human granulocytotropic Ehrlichia spp. (Rickettsiales: Ehrlichieae). J Med Entomol. 1997;34(6):710-8. Epub 1998/01/24. doi: 10.1093/jmedent/34.6.710. PubMed PMID: 9439127.

1030. Fingerle V, Goodman JL, Johnson RC, Kurtti TJ, Munderloh UG, Wilske B. Human granulocytic ehrlichiosis in southern Germany: increased seroprevalence in high-risk groups. J Clin Microbiol. 1997;35(12):3244-7. Epub 1997/12/17. doi: 10.1128/jcm.35.12.3244-3247.1997. PubMed PMID: 9399527; PubMed Central PMCID: PMCPMC230155.

1031. Finkelman FD, Shea-Donohue T, Goldhill J, Sullivan CA, Morris SC, Madden KB, et al. Cytokine regulation of host defense against parasitic gastrointestinal nematodes: lessons from studies with rodent models. Annu Rev Immunol. 1997;15:505-33. Epub 1997/01/01. doi: 10.1146/annurev.immunol.15.1.505. PubMed PMID: 9143698.

1032. Fritz CL, Kjemtrup AM, Conrad PA, Flores GR, Campbell GL, Schriefer ME, et al. Seroepidemiology of emerging tickborne infectious diseases in a Northern California community. J Infect Dis. 1997;175(6):1432-9. Epub 1997/06/01. doi: 10.1086/516476. PubMed PMID: 9180183.

1033. Gluckman SJ. Ehrlichia infections of humans. Infectious Diseases in Clinical Practice. 1997;6(2):96-100. doi: 10.1097/00019048-199702000-00005.

1034. Glushko GM. Human ehrlichiosis. Postgrad Med. 1997;101(6):225-30. Epub 1997/06/01. doi: 10.3810/pgm.1997.06.231. PubMed PMID: 9194875.

1035. Glushko GM. Human ehrlichiosis. Yet another zebra or a recently recognized horse? Postgraduate Medicine. 1997;101(6):225-30. doi: 10.3810/pgm.1997.06.231.

1036. Goddard J. Rickettsial organisms transmitted by ticks: Ehrlichiosis. Infections in Medicine. 1997;14(3):224+9-30.

1037. Grencis RK, Entwistle GM. Production of an interferon-gamma homologue by an intestinal nematode: functionally significant or interesting artefact? Parasitology. 1997;115 Suppl:S101-6. Epub 1997/01/01. doi: 10.1017/s0031182097002114. PubMed PMID: 9571695.

1038. Heimer R, Van Andel A, Wormser GP, Wilson ML. Propagation of granulocytic Ehrlichia spp. from human and equine sources in HL-60 cells induced to differentiate into functional granulocytes. J Clin Microbiol. 1997;35(4):923-7. Epub 1997/04/01. doi: 10.1128/jcm.35.4.923-927.1997. PubMed PMID: 9157154; PubMed Central PMCID: PMCPMC229702.

1039. Heimer R, VanAndel A, Wormser GP, Wilson ML. Propagation of granulocytic Ehrlichia spp. from human and equine sources in HL-60 cells induced to differentiate into functional granulocytes. Journal of Clinical Microbiology. 1997;35(4):923-7. doi: 10.1128/jcm.35.4.923-927.1997. PubMed PMID: WOS:A1997WP40500023.

1040. Heppner DG, Wongsrichanalai C, Walsh DS, McDaniel P, Eamsila C, Hanson B, et al. Human ehrlichiosis in Thailand. Lancet. 1997;350(9080):785-6. Epub 1997/09/23. doi: 10.1016/s0140-6736(05)62571-8. PubMed PMID: 9298007.

1041. Heppner DG, Wongsrichanalai C, Walsh DS, McDaniel P, Eamsila C, Hanson B, et al. Human ehrlichiosis in Thailand [8]. Lancet. 1997;350(9080):785-6. doi: 10.1016/S0140-6736(05)62571-8.

1042. Horowitz HW, Wormser GP. Human granulocytic ehrlichiosis. Clinical Immunology Newsletter. 1997;17(10-11):141-6. doi: 10.1016/S0197-1859(00)80018-7.

1043. Hsieh TC, Aguero-Rosenfeld ME, Wu JM, Ng C, Papanikolaou NA, Varde SA, et al. Cellular changes and induction of apoptosis in human promyelocytic HL-60 cells infected with the agent of human granulocytic ehrlichiosis (HGE). Biochem Biophys Res Commun. 1997;232(2):298-303. Epub 1997/03/17. doi: 10.1006/bbrc.1997.6276. PubMed PMID: 9125168.

1044. Hsieh TC, AgueroRosenfeld ME, Wu JM, Ng CY, Papanikolaou NA, Varde SA, et al. Cellular changes and induction of apoptosis in human promyelocytic HL-60 cells infected with the agent of human granulocytic ehrlichiosis (HGE). Biochemical and Biophysical Research Communications. 1997;232(2):298-303. doi: 10.1006/bbrc.1997.6276. PubMed PMID: WOS:A1997WP22300008.

1045. Ijdo JW, Zhang Y, Hodzic E, Magnarelli LA, Wilson ML, Telford Iii SR, et al. The early humoral response in human granulocytic ehrlichiosis. Journal of Infectious Diseases. 1997;176(3):687-92. doi: 10.1086/514091.

1046. Ijdo JW, Zhang Y, Hodzic E, Magnarelli LA, Wilson ML, Telford SR, et al. The early humoral response in human granulocytic ehrlichiosis. Journal of Infectious Diseases. 1997;176(3):687-92. doi: 10.1086/514091. PubMed PMID: WOS:A1997XT81900019.

1047. Ionas G, Farrant KJ, McLenachan PA, Clarke JK, Brown TJ. Molecular (PCR) differentiation of Giardia muris and Giardia intestinalis. International Journal of Environmental Health Research. 1997;7(1):63-9.

1048. Jacobs RF, Schutze GE. Ehrlichiosis in children. J Pediatr. 1997;131(2):184-92. Epub 1997/08/01. doi: 10.1016/s0022-3476(97)70152-5. PubMed PMID: 9290602.

1049. Johnson DC, Enriquez CE, Pepper IL, Davis TL, Gerba CP, Rose JB. Survival of Giardia, Cryptosporidium, poliovirus and Salmonella in marine waters. Water Science and Technology. 1997;35(11-12):261-8. doi: 10.1016/S0273-1223(97)00270-9.

1050. Jorde UP. Human granulocytic ehrlichiosis: a cardiac risk factor? Ann Intern Med. 1997;127(1):89-90. Epub 1997/07/01. doi: 10.7326/0003-4819-127-1-199707010-00023. PubMed PMID: 9214264.

1051. Jorde UP. Human granulocytic ehrlichiosis: a cardiac risk factor? Philadelphia, Pennsylvania: American College of Physicians; 1997. p. 89-90.

1052. Jorde UP, Aguero-Rosenfeld ME, Wormser GP, Dumler JS. Human granulocytic ehrlichiosis: A cardiac risk factor? [5] (multiple letters). Annals of Internal Medicine. 1997;127(1):89-90. doi: 10.7326/0003-4819-127-1-199707010-00023.

1053. JW IJ, Zhang Y, Hodzic E, Magnarelli LA, Wilson ML, Telford SR, 3rd, et al. The early humoral response in human granulocytic ehrlichiosis. J Infect Dis. 1997;176(3):687-92. Epub 1997/09/18. doi: 10.1086/514091. PubMed PMID: 9291316.

1054. Kirkland KB, Klimko TB, Meriwether RA, Schriefer M, Levin M, Levine J, et al. Erythema migrans-like rash illness at a camp in North Carolina: a new tick-borne disease? Arch Intern Med. 1997;157(22):2635-41. Epub 1998/04/08. PubMed PMID: 9531233.

1055. Klein MB, Hu S, Chao CC, Goodman JL. The agent of human granulocytic ehrlichiosis (HGE) induces the production of myelosuppressing chemokines. Clinical Infectious Diseases. 1997;25(2):427.

1056. Klein MB, Miller JS, Nelson CM, Goodman JL. Primary bone marrow progenitors of both granulocytic and monocytic lineages are susceptible to infection with the agent of human granulocytic ehrlichiosis. J Infect Dis. 1997;176(5):1405-9. Epub 1997/11/14. doi: 10.1086/517332. PubMed PMID: 9359749.

1057. Klein MB, Nelson CM, Goodman JL. Antibiotic susceptibility of the newly cultivated agent of human granulocytic ehrlichiosis: promising activity of quinolones and rifamycins. Antimicrob Agents Chemother. 1997;41(1):76-9. Epub 1997/01/01. doi: 10.1128/aac.41.1.76. PubMed PMID: 8980758; PubMed Central PMCID: PMCPMC163663.

1058. Kolbert CP, Bruinsma ES, Abdulkarim AS, Hofmeister EK, Tompkins RB, Telford Iii SR, et al. Characterization of an immunoreactive protein from the agent of human granulocytic ehrlichiosis. Journal of Clinical Microbiology. 1997;35(5):1172-8. doi: 10.1128/jcm.35.5.1172-1178.1997.

1059. Kolbert CP, Bruinsma ES, Abdulkarim AS, Hofmeister EK, Tompkins RB, Telford SR, 3rd, et al. Characterization of an immunoreactive protein from the agent of human granulocytic ehrlichiosis. J Clin Microbiol. 1997;35(5):1172-8. Epub 1997/05/01. doi: 10.1128/jcm.35.5.1172-1178.1997. PubMed PMID: 9114402; PubMed Central PMCID: PMCPMC232724.

1060. Kolbert CP, Bruinsma ES, Abdulkarim AS, Hofmeister EK, Tompkins RB, Telford SR, et al. Characterization of an immunoreactive protein from the agent of human granulocytic ehrlichiosis. Journal of Clinical Microbiology. 1997;35(5):1172-8. doi: 10.1128/jcm.35.5.1172-1178.1997. PubMed PMID: WOS:A1997WV17800025.

1061. Krause PJ, Telford Iii SR. Emerging tick-borne zoonoses: Lyme disease, babesiosis, human granulocytic ehrlichiosis. Seminars in Pediatric Infectious Diseases. 1997;8(1):34-43.

1062. Lee EH, Rikihisa Y. Anti-Ehrlichia chaffeensis antibody complexed with E. chaffeensis induces potent proinflammatory cytokine mRNA expression in human monocytes through sustained reduction of IkappaB-alpha and activation of NF-kappaB. Infect Immun. 1997;65(7):2890-7. Epub 1997/07/01. doi: 10.1128/iai.65.7.2890-2897.1997. PubMed PMID: 9199464; PubMed Central PMCID: PMCPMC175406.

1063. Lee EH, Rikihisa Y. Anti-Ehrlichia chaffeensis antibody complexed with E. chaffeensis Induces potent proinflammatory cytokine mRNA expression in human monocytes through sustained reduction of IκB-α and activation of NF-κB. Infection and Immunity. 1997;65(7):2890-7. doi: 10.1128/iai.65.7.2890-2897.1997.

1064. Lee EH, Rikihisa Y. Anti Ehrlichia chaffeensis antibody complexed with E-chaffeensis induces potent proinflammatory cytokine mRNA expression in human monocytes through sustained reduction of I kappa B-alpha and activation of NF-kappa B. Infection and Immunity. 1997;65(7):2890-7. doi: 10.1128/iai.65.7.2890-2897.1997. PubMed PMID: WOS:A1997XF63100053.

1065. Little SE, Dawson JE, Lockhart JM, Stallknecht DE, Warner CK, Davidson WR. Development and use of specific polymerase reaction for the detection of an organism resembling Ehrlichia sp. in white-tailed deer. Journal of Wildlife Diseases. 1997;33(2):246-53. doi: 10.7589/0090-3558-33.2.246.

1066. Lockhart JM, Davidson WR, Stallknecht DE, Dawson JE, Howerth EW. Isolation of Ehrlichia chaffeensis from wild white-tailed deer (Odocoileus virginianus) confirms their role as natural reservoir hosts. J Clin Microbiol. 1997;35(7):1681-6. Epub 1997/07/01. doi: 10.1128/jcm.35.7.1681-1686.1997. PubMed PMID: 9196173; PubMed Central PMCID: PMCPMC229821.

1067. Lukin EP, Makhlaĭ AA, Perepelkin VS. [Rickettsioses: the epidemiological assessment]. Voen Med Zh. 1997;318(8):25-33, 78. Epub 1997/08/01. PubMed PMID: 9424811.

1068. Mafiana CF, Osho MB, Sam-Wobo S. Gastrointestinal helminth parasites of the black rat (Rattus rattus) in Abeokuta, southwest Nigeria. J Helminthol. 1997;71(3):217-20. Epub 1998/08/15. doi: 10.1017/s0022149x00015947. PubMed PMID: 9705679.

1069. Magnarelli LA, Anderson JF, Stafford KC, Dumler JS. Antibodies to multiple tick-borne pathogens of babesiosis, ehrlichiosis, and Lyme borreliosis in white-footed mice. Journal of Wildlife Diseases. 1997;33(3):466-73. doi: 10.7589/0090-3558-33.3.466. PubMed PMID: WOS:A1997XM00400009.

1070. Magnarelli LA, Ijdo JW, Anderson JF, Madigan JE, Dumler JS, Fikrig E. Antibodies to Ehrlichia equi in dogs from the northeastern United States. Journal of the American Veterinary Medical Association. 1997;211(9):1134-7.

1071. Marique T, Blankaert D, Hendrick V, Raschella A, Declerck B, Alloin C, et al. Biological response of endothelial cells and its modulation by cytokines: prospects for therapy and bioprocesses. Cytotechnology. 1997;25(1-3):183-9. Epub 1997/11/01. doi: 10.1023/a:1007928203997. PubMed PMID: 22358890; PubMed Central PMCID: PMCPMC3466742.

1072. Mazzella FM, Kranwinkel R. Lyme disease and human granulocytic ehrlichiosis. Am J Clin Pathol. 1997;108(4):481; author reply 2-3. Epub 1997/10/10. PubMed PMID: 9322604.

1073. Means RG, White DJ. New Distribution Records of Amblyomma americanum (L.) (Acari: Ixodidae) in New York State. Journal of Vector Ecology. 1997;22(2):133-45.

1074. Merckelbach H, Muris P. The etiology of childhood spider phobia. Behav Res Ther. 1997;35(11):1031-4. Epub 1998/02/12. doi: 10.1016/s0005-7967(97)00054-5. PubMed PMID: 9431733.

1075. Monteyne P, Renauld JC, VanBroeck J, Dunne DW, Brombacher F, Coutelier JP. IL-4-independent regulation of in vivo IL-9 expression. Journal of Immunology. 1997;159(6):2616-23. PubMed PMID: WOS:A1997XV75000011.

1076. Nadelman RB, Horowitz HW, Hsieh TC, Wu JM, Aguero-Rosenfeld ME, Schwartz I, et al. Simultaneous human granulocytic ehrlichiosis and Lyme borreliosis. N Engl J Med. 1997;337(1):27-30. Epub 1997/07/03. doi: 10.1056/nejm199707033370105. PubMed PMID: 9203428.

1077. Nadelman RB, Horowitz HW, Hsieh TC, Wu JM, AgueroRosenfeld ME, Schwartz I, et al. Simultaneous human granulocytic ehrlichiosis and Lyme borreliosis. New England Journal of Medicine. 1997;337(1):27-30. doi: 10.1056/nejm199707033370105. PubMed PMID: WOS:A1997XH18600005.

1078. Nicholson WL, Comer JA, Sumner JW, Gingrich-Baker C, Coughlin RT, Magnarelli LA, et al. An indirect immunofluorescence assay using a cell culture-derived antigen for detection of antibodies to the agent of human granulocytic ehrlichiosis. J Clin Microbiol. 1997;35(6):1510-6. Epub 1997/06/01. doi: 10.1128/jcm.35.6.1510-1516.1997. PubMed PMID: 9163471; PubMed Central PMCID: PMCPMC229776.

1079. Nicholson WL, Comer JA, Sumner JW, GingrichBaker C, Coughlin RT, Magnarelli LA, et al. An indirect immunofluorescence assay using a cell culture-derived antigen for detection of antibodies to the agent of human granulocytic ehrlichiosis. Journal of Clinical Microbiology. 1997;35(6):1510-6. doi: 10.1128/jcm.35.6.1510-1516.1997. PubMed PMID: WOS:A1997XA75500040.

1080. Olsen I. Salient structural features in the chemical composition of oral anaerobes, with particular emphasis on plasmalogens and sphingolipids. Reviews and Research in Medical Microbiology. 1997;8(SUPPL. 1):S3-S6.

1081. Omezzine-Letaief A, Tissot Dupont H, Bahri F, Ernez M, Raoult D, Jemni L. Seroepidemiologic study among 300 febrile patients in a infectious disease hospital ward. Medecine et Maladies Infectieuses. 1997;27(SPEC. ISS. JUNE):663-6.

1082. Patel SM, Heptinstall J. Species specific polymerase chain reaction to detect Cryptosporidium parvum and C. muris. Biochem Soc Trans. 1997;25(1):19s. Epub 1997/02/01. doi: 10.1042/bst025019s. PubMed PMID: 9056917.

1083. Perez JM, Martinez D, Debus A, Christian S, Bensaid A. Detection of genomic polymorphisms among isolates of the intracellular bacterium Cowdria ruminantium by random amplified polymorphic DNA and southern blotting. FEMS Microbiology Letters. 1997;154(1):73-9. doi: 10.1016/S0378-1097(97)00303-0.

1084. Perez JM, Martinez D, Debus A, Sheikboudou C, Bensaid A. Detection of genomic polymorphisms among isolates of the intracellular bacterium Cowdria ruminantium by random amplified polymorphic DNA and Southern blotting. FEMS Microbiol Lett. 1997;154(1):73-9. Epub 1997/09/23. doi: 10.1111/j.1574-6968.1997.tb12626.x. PubMed PMID: 9297823.

1085. Persing DH. The cold zone: a curious convergence of tick-transmitted diseases. Clin Infect Dis. 1997;25 Suppl 1:S35-42. Epub 1997/07/01. doi: 10.1086/516170. PubMed PMID: 9233662.

1086. Persing DH, editor The cold zone: A curious convergence of tick-transmitted diseases. Clinical Infectious Diseases; 1997.

1087. Petrovec M, Furlan SL, Zupanc TA, Strle F, Brouqui P, Roux V, et al. Human disease in Europe caused by a granulocytic Ehrlichia species. Journal of Clinical Microbiology. 1997;35(6):1556-9. doi: 10.1128/jcm.35.6.1556-1559.1997.

1088. Petrovec M, Lotric Furlan S, Zupanc TA, Strle F, Brouqui P, Roux V, et al. Human disease in Europe caused by a granulocytic Ehrlichia species. J Clin Microbiol. 1997;35(6):1556-9. Epub 1997/06/01. doi: 10.1128/jcm.35.6.1556-1559.1997. PubMed PMID: 9163481; PubMed Central PMCID: PMCPMC229786.

1089. Pristas P, Vanat I, Javorský P. Variability of endonucleolytic activity indicates high genetic diversity within the natural population ofSelenomonas ruminantium. Folia Microbiol (Praha). 1997;42(2):121-4. Epub 1997/04/01. doi: 10.1007/bf02898719. PubMed PMID: 18454331.

1090. Pusterla N, Huder J, Wolfensberger C, Litschi B, Parvis A, Lutz H. Granulocytic ehrlichiosis in two dogs in Switzerland. J Clin Microbiol. 1997;35(9):2307-9. Epub 1997/09/01. doi: 10.1128/jcm.35.9.2307-2309.1997. PubMed PMID: 9276407; PubMed Central PMCID: PMCPMC229959.

1091. Pusterla N, Wolfensberger C, GerberBretscher R, Lutz H. Comparison of indirect immunofluorescence for Ehrlichia phagocytophila and Ehrlichia equi in horses. Equine Veterinary Journal. 1997;29(6):490-2. doi: 10.1111/j.2042-3306.1997.tb03165.x. PubMed PMID: WOS:A1997YH60400017.

1092. Rikihisa Y, Zhi N, Wormser GP, Wen B, Horowitz HW, Hechemy KE. Ultrastructural and antigenic characterization of a granulocytic ehrlichiosis agent directly isolated and stably cultivated from a patient in New York state. J Infect Dis. 1997;175(1):210-3. Epub 1997/01/01. doi: 10.1093/infdis/175.1.210. PubMed PMID: 8985223.

1093. Rikihisa Y, Zhi N, Wormser GP, Wen B, Horowitz HW, Hechemy KE, et al. Ultrastructural and antigenic characterization of a granulocytic ehrlichiosis agent directly isolated and stably cultivated from a patient in New York state. Journal of Infectious Diseases. 1997;175(1):210-3. PubMed PMID: 79840382. Language: English. Entry Date: 19970801. Revision Date: 20190522. Publication Type: journal article.

1094. Rikihisa Y, Zhi N, Wormser GP, Wen BH, Horowitz HW, Hechemy KE. Ultrastructural and antigenic characterization of a granulocytic ehrlichiosis agent directly isolated and stably cultivated from a patient in New York State. Journal of Infectious Diseases. 1997;175(1):210-3. doi: 10.1093/infdis/175.1.210. PubMed PMID: WOS:A1997WA93700035.

1095. Roberts R, Soave R. Emerging Pathogens Associated with Tick-Borne Infections. Braz J Infect Dis. 1997;1(1):17-26. Epub 1997/03/01. PubMed PMID: 11107234.

1096. Schutze GE, Jacobs RF. Human monocytic ehrlichiosis in children. Pediatrics. 1997;100(1):E10. Epub 1997/07/01. doi: 10.1542/peds.100.1.e10. PubMed PMID: 9200384.

1097. Schwartz I, Fish D, Daniels TJ. Prevalence of the rickettsial agent of human granulocytic ehrlichiosis in ticks from a hyperendemic focus of lyme disease [1]. New England Journal of Medicine. 1997;337(1):49-50. doi: 10.1056/NEJM199707033370111.

1098. Schwartz I, Fish D, Daniels TJ. Prevalence of the rickettsial agent of human granulocytic ehrlichiosis in ticks from a hyperendemic focus of Lyme disease. New England Journal of Medicine. 1997;337(1):49-50. doi: 10.1056/nejm199707033370111. PubMed PMID: WOS:A1997XH18600011.

1099. Sood SK, Stephen Dumler J. Human Ehrlichia equi/phagocytophila (EEP) infection on long Island (LI). Clinical Infectious Diseases. 1997;25(2):480.

1100. Strausbaugh LJ. Emerging infectious diseases: a challenge to all. Am Fam Physician. 1997;55(1):111-7. Epub 1997/01/01. PubMed PMID: 9012271.

1101. Sumner JW, Nicholson WL, Massung RF. PCR amplification and comparison of nucleotide sequences from the groESL heat shock operon of Ehrlichia species. J Clin Microbiol. 1997;35(8):2087-92. Epub 1997/08/01. doi: 10.1128/jcm.35.8.2087-2092.1997. PubMed PMID: 9230387; PubMed Central PMCID: PMCPMC229908.

1102. Sun W, Ijdo JW, Telford Iii SR, Hodzic E, Zhang Y, Barthold SW, et al. Immunization against the agent of human granulocytic ehrlichiosis in a murine model. Journal of Clinical Investigation. 1997;100(12):3014-8. doi: 10.1172/JCI119855.

1103. Sun W, Ijdo JW, Telford SR, Hodzic E, Zhang Y, Barthold SW, et al. Immunization against the agent of human granulocytic ehrlichiosis in a murine model. Journal of Clinical Investigation. 1997;100(12):3014-8. doi: 10.1172/jci119855. PubMed PMID: WOS:000071149100013.

1104. Sun W, JW IJ, Telford SR, 3rd, Hodzic E, Zhang Y, Barthold SW, et al. Immunization against the agent of human granulocytic ehrlichiosis in a murine model. J Clin Invest. 1997;100(12):3014-8. Epub 1998/01/31 20:29. doi: 10.1172/jci119855. PubMed PMID: 9399947; PubMed Central PMCID: PMCPMC508513.

1105. Telford SR, 3rd. Risk for acquiring human granulocytic ehrlichiosis: exposure to deer blood or deer ticks? Clin Infect Dis. 1997;24(3):531-3. Epub 1997/03/01. doi: 10.1093/clinids/24.3.531. PubMed PMID: 9114225.

1106. Telford SR, Armstrong PM, Katavolos P, Foppa I, Garcia ASO, Wilson ML, et al. A new tick-borne encephalitis-like virus infecting New England deer ticks, Ixodes dammini. Emerging Infectious Diseases. 1997;3(2):165-70. doi: 10.3201/eid0302.970209. PubMed PMID: WOS:A1997XL15500009.

1107. Telford SR, III, Bakken JS, Dumler JS. Risk for acquiring human granulocytic ehrlichiosis: Exposure to deer blood or deer ticks? [2]. Clinical Infectious Diseases. 1997;24(3):531-3. doi: 10.1093/clinids/24.3.531.

1108. von Stedingk LV, Gürtelschmid M, Hanson HS, Gustafson R, Dotevall L, Engvall EO, et al. The human granulocytic ehrlichiosis (HGE) agent in Swedish ticks. Clin Microbiol Infect. 1997;3(5):573-4. Epub 1997/01/01. doi: 10.1111/j.1469-0691.1997.tb00311.x. PubMed PMID: 11864185.

1109. Walker DH, Dumler JS. Human monocytic and granulocytic ehrlichioses. Discovery and diagnosis of emerging tick-borne infections and the critical role of the pathologist. Arch Pathol Lab Med. 1997;121(8):785-91. Epub 1997/08/01. PubMed PMID: 9278605.

1110. Walker DH, Dumler JS. Human monocytic and granulocytic ehrlichioses: Discovery and diagnosis of emerging tick, borne infections and the critical role of the pathologist. Archives of Pathology and Laboratory Medicine. 1997;121(8):785-91.

1111. Walker DH, Dumler JS. Human monocytic and granulocytic ehrlichioses - Discovery and diagnosis of emerging tick-borne infections and the critical role of the pathologist. Archives of Pathology & Laboratory Medicine. 1997;121(8):785-91. PubMed PMID: WOS:A1997XR79100003.

1112. Walls JJ, Greig B, Neitzel DF, Dumler JS. Natural infection of small mammal species in Minnesota with the agent of human granulocytic ehrlichiosis. J Clin Microbiol. 1997;35(4):853-5. Epub 1997/04/01. doi: 10.1128/jcm.35.4.853-855.1997. PubMed PMID: 9157141; PubMed Central PMCID: PMCPMC229689.

1113. Wong SJ, Brady GS, Dumler JS. Serological responses to Ehrlichia equi, Ehrlichia chaffeensis, and Borrelia burgdorferi in patients from New York State. J Clin Microbiol. 1997;35(9):2198-205. Epub 1997/09/01. doi: 10.1128/jcm.35.9.2198-2205.1997. PubMed PMID: 9276387; PubMed Central PMCID: PMCPMC229939.

1114. Wormser GP, Horowitz HW, Nowakowski J, McKenna D, Dumler JS, Varde S, et al. Positive Lyme disease serology in patients with clinical and laboratory evidence of human granulocytic ehrlichiosis. Am J Clin Pathol. 1997;107(2):142-7. Epub 1997/02/01. doi: 10.1093/ajcp/107.2.142. PubMed PMID: 9024062.

1115. Wormser GP, Horowitz HW, Nowakowski J, McKenna D, Dumler JS, Varde S, et al. Positive Lyme disease serology in patients with clinical and laboratory evidence of human granulocytic ehrlichiosis. American Journal of Clinical Pathology. 1997;107(2):142-7. doi: 10.1093/ajcp/107.2.142. PubMed PMID: WOS:A1997WF17100002.

1116. Wormser GP, Horowitz HW, Nowakowski J, McKenna D, Schwartz I, AgueroRosenfeld M, et al. Lyme disease and human granulocytic ehrlichiosis - Reply. American Journal of Clinical Pathology. 1997;108(4):482-3. PubMed PMID: WOS:A1997XX43400019.

1117. Yeh MT, Mather TN, Coughlin RT, Gingrich-Baker C, Sumner JW, Massung RF. Serologic and molecular detection of granulocytic ehrlichiosis in Rhode Island. J Clin Microbiol. 1997;35(4):944-7. Epub 1997/04/01. doi: 10.1128/jcm.35.4.944-947.1997. PubMed PMID: 9157157; PubMed Central PMCID: PMCPMC229705.

1118. Yeh MT, Mather TN, Coughlin RT, GingrichBaker C, Sumner JW, Massung RF. Serologic and molecular detection of granulocytic ehrlichiosis in Rhode Island. Journal of Clinical Microbiology. 1997;35(4):944-7. doi: 10.1128/jcm.35.4.944-947.1997. PubMed PMID: WOS:A1997WP40500026.

1119. Yu XJ, Crocquet-Valdes P, Walker DH. Corrigendum: Cloning and sequencing of the gene for a 120-kDa immunodominant protein of Ehrlichia chaffeensis (Gene (1997) 184 (149-154)). Gene. 1997;191(2):233. doi: 10.1016/S0378-1119(97)00157-1.

1120. Yu XJ, Crocquet-Valdes P, Walker DH. Cloning and sequencing of the gene for a 120-kDa immunodominant protein of Ehrlichia chaffeensis. Gene. 1997;184(2):149-54. doi: 10.1016/S0378-1119(96)00586-0.

1121. Yu XJ, CrocquetValdes P, Walker DH. Cloning and sequencing of the gene for a 120-kDa immunodominant protein of Ehrlichia chaffeensis. Gene. 1997;184(2):149-54. doi: 10.1016/s0378-1119(96)00586-0. PubMed PMID: WOS:A1997WE85500002.

1122. Yu XJ, Walker DH. Sequence and characterization of an Ehrlichia chaffeensis gene encoding 314 amino acids highly homologous to the NAD A enzyme. FEMS Microbiol Lett. 1997;154(1):53-8. Epub 1997/09/23. doi: 10.1111/j.1574-6968.1997.tb12623.x. PubMed PMID: 9297820.

1123. Zhang Y, Ohashi N, Lee EH, Tamura A, Rikihisa Y. Ehrlichia sennetsu groE operon and antigenic properties of the GroEL homolog. FEMS Immunol Med Microbiol. 1997;18(1):39-46. Epub 1997/05/01. doi: 10.1111/j.1574-695X.1997.tb01025.x. PubMed PMID: 9215585.

1124. Zhang Y, Rikihisa Y. Tyrosine phosphorylation is required for Ehrlichial internalization and replication in P388D1 cells. Infection and Immunity. 1997;65(7):2959-64. doi: 10.1128/iai.65.7.2959-2964.1997.

1125. Zhang YL, Ohashi N, Lee EH, Tamura A, Rikihisa Y. Ehrlichia sennetsu groE operon and antigenic properties of the GroEL homolog. Fems Immunology and Medical Microbiology. 1997;18(1):39-46. doi: 10.1111/j.1574-695X.1997.tb01025.x. PubMed PMID: WOS:A1997XH31400005.

1126. Zhi N, Rikihisa Y, Kim HY, Wormser GP, Horowitz HW. Comparison of major antigenic proteins of six strains of the human granulocytic ehrlichiosis agent by Western immunoblot analysis. J Clin Microbiol. 1997;35(10):2606-11. Epub 1997/10/08. doi: 10.1128/jcm.35.10.2606-2611.1997. PubMed PMID: 9316916; PubMed Central PMCID: PMCPMC230019.

1127. Statewide surveillance for ehrlichiosis--Connecticut and New York, 1994-1997. MMWR Morb Mortal Wkly Rep. 1998;47(23):476-80. Epub 1998/07/02. PubMed PMID: 9649234.

1128. From the Centers for Disease Control and Prevention. Statewide surveillance for ehrlichiosis--Connecticut and New York, 1994-1997. Jama. 1998;280(15):1300-1. Epub 1998/10/30. PubMed PMID: 9794298.

1129. Alberdi MP, Walker AR, Paxton EA, Sumption KJ. Natural prevalence of infection with Ehrlichia (Cytoecetes) phagocytophila of Ixodes ricinus ticks in Scotland. Veterinary Parasitology. 1998;78(3):203-13. doi: 10.1016/s0304-4017(98)00138-1. PubMed PMID: WOS:000075837800005.

1130. Anderson BC. Cryptosporidiosis in bovine and human health. J Dairy Sci. 1998;81(11):3036-41. Epub 1998/12/05. doi: 10.3168/jds.S0022-0302(98)75868-0. PubMed PMID: 9839243.

1131. Andrews RH, Monis PT, Ey PL, Mayrhofer G. Comparison of the levels of intra-specific genetic variation within Giardia muris and Giardia intestinalis. Int J Parasitol. 1998;28(8):1179-85. Epub 1998/10/08. doi: 10.1016/s0020-7519(98)00097-6. PubMed PMID: 9762562.

1132. Arraga-Alvarado C, Palmar M, Parra O, Salas P, Ramos N, editors. Identification of a human platelet's organism similar but not identical to Ehrlichia platys using transmission electron microscopy. 14th International Congress on Electron Microscopy; 1998 Aug 31-Sep 04; Cancun, Mexico1998.

1133. Atasoglu C, Valdés C, Walker ND, Newbold CJ, Wallace RJ. De novo synthesis of amino acids by the ruminal bacteria Prevotella bryantii B14, Selenomonas ruminantium HD4, and Streptococcus bovis ES1. Applied and Environmental Microbiology. 1998;64(8):2836-43. doi: 10.1128/aem.64.8.2836-2843.1998.

1134. Bakken JS. The discovery of human granulocytotropic ehrlichiosis. J Lab Clin Med. 1998;132(3):175-80. Epub 1998/09/15. doi: 10.1016/s0022-2143(98)90165-2. PubMed PMID: 9735922.

1135. Bakken JS, Dumler JS, Kristiansen BE. [A new tick-borne disease--human granulocytic ehrlichiosis]. Tidsskr Nor Laegeforen. 1998;118(26):4117-8. Epub 1998/12/09. PubMed PMID: 9844520.

1136. Bakken JS, Erlemeyer SA, Kanoff RJ, Silvestrini Ii TC, Goodwin DD, Dumler JS. Demyelinating polyneuropathy associated with human granulocytic ehrlichiosis. Clinical Infectious Diseases. 1998;27(5):1323-4. doi: 10.1093/clinids/27.5.1323.

1137. Bakken JS, Erlemeyer SA, Kanoff RJ, Silvestrini TC, 2nd, Goodwin DD, Dumler JS. Demyelinating polyneuropathy associated with human granulocytic ehrlichiosis. Clin Infect Dis. 1998;27(5):1323-4. Epub 1998/11/25. PubMed PMID: 9827293.

1138. Bakken JS, Goellner P, Van Etten M, Boyle DZ, Swonger OL, Mattson S, et al. Seroprevalence of human granulocytic ehrlichiosis among permanent residents of northwestern Wisconsin. Clin Infect Dis. 1998;27(6):1491-6. Epub 1998/12/30. doi: 10.1086/515048. PubMed PMID: 9868666.

1139. Barbour AG. Fall and rise of Lyme disease and other Ixodes tick-borne infections in North America and Europe. British Medical Bulletin. 1998;54(3):647-58. PubMed PMID: WOS:000080271300012.

1140. Bedner E, Burfeind P, Hsieh T, Wu JM, Aguero-Rosenfeld ME, Melamed MR, et al. Cell cycle effects and induction of apoptosis caused by infection of HL-60 cells with human granulocytic ehrlichiosis pathogen measured by flow and laser scanning cytometry. Cytometry. 1998;33(1):47-55. doi: 10.1002/(sici)1097-0320(19980901)33:1<47::Aid-cyto6>3.0.Co;2-8. PubMed PMID: WOS:000075434100006.

1141. Bedner E, Burfeind P, Hsieh TC, Wu JM, Aguero-Rosenfeld ME, Melamed MR, et al. Cell cycle effects and induction of apoptosis caused by infection of HL-60 cells with human granulocytic ehrlichiosis pathogen measured by flow and laser scanning cytometry. Cytometry. 1998;33(1):47-55. Epub 1998/09/02. PubMed PMID: 9725558.

1142. Billings AN, Rawlings JA, Walker DH. Tick-borne diseases in Texas: a 10-year retrospective examination of cases. Tex Med. 1998;94(12):66-76. Epub 1998/12/17. PubMed PMID: 9854421.

1143. Biswas B, Vemulapalli R, Dutta SK. Molecular basis for antigenic variation of a protective strain-specific antigen of Ehrlichia risticii. Infect Immun. 1998;66(8):3682-8. Epub 1998/07/23. doi: 10.1128/iai.66.8.3682-3688.1998. PubMed PMID: 9673249; PubMed Central PMCID: PMCPMC108402.

1144. Brouqui P. Human ehrlichiose, an emerging infectious disease. Medecine et Maladies Infectieuses. 1998;28(4 SPECIAL MAY):349-53. doi: 10.1016/s0399-077x(98)70220-3.

1145. Brouqui P, editor Human ehrlichiose, an emerging infectious disease. Medecine et Maladies Infectieuses; 1998.

1146. Buitrago MI, Ijdo JW, Rinaudo P, Simon H, Copel J, Gadbaw J, et al. Human granulocytic ehrlichiosis during pregnancy treated successfully with rifampin. Clin Infect Dis. 1998;27(1):213-5. Epub 1998/07/24. doi: 10.1086/517678. PubMed PMID: 9675481.

1147. Bull SA, Chalmers RM, Sturdee AP, Healing TD. A survey of Cryptosporidium species in Skomer bank voles (Clethrionomys glareolus skomerensis). Journal of Zoology. 1998;244(1):119-22. doi: 10.1017/S0952836998001137.

1148. Bunnell JE, Dumler JS, Childs JE, Glass GE. Retrospective serosurvey for human granulocytic ehrlichiosis agent in urban white-footed mice from Maryland. J Wildl Dis. 1998;34(1):179-81. Epub 1998/02/26. doi: 10.7589/0090-3558-34.1.179. PubMed PMID: 9476244.

1149. Burket CT, Vann CN, Pinger RR, Chatot CL, Steiner FE. Minimum infection rate of Ambylomma americanum (Acari: Ixodidae) by Ehrlichia chaffeensis (Rickettsiales: Ehrlichieae) in southern Indiana. J Med Entomol. 1998;35(5):653-9. Epub 1998/10/17. doi: 10.1093/jmedent/35.5.653. PubMed PMID: 9775587.

1150. Casleton BG, Salata K, Dasch GA, Strickman D, Kelly DJ. Recovery and viability of Orientia tsutsugamushi from packed red cells and the danger of acquiring scrub typhus from blood transfusion. Transfusion. 1998;38(7):680-9. doi: 10.1046/j.1537-2995.1998.38798346638.x. PubMed PMID: WOS:000074956300011.

1151. Cerundolo, De C, Manna, Gravino. Recurrent deep pyoderma in German Shepherd dogs with underlying ehrlichioses and hypergammaglobulinaemia. Vet Dermatol. 1998;9(2):135-42. Epub 1998/06/01. doi: 10.1046/j.1365-3164.1998.00102.x. PubMed PMID: 34645013.

1152. Champliaud D, Gobet P, Naciri M, Vagner O, Lopez J, Buisson JC, et al. Failure to differentiate Cryptosporidium parvum from C. meleagridis based on PCR amplification of eight DNA sequences. Appl Environ Microbiol. 1998;64(4):1454-8. Epub 1998/05/09. doi: 10.1128/aem.64.4.1454-1458.1998. PubMed PMID: 9575132; PubMed Central PMCID: PMCPMC106169.

1153. Champliaud D, Gobet P, Naciri M, Vagner O, Lopez J, Buisson JC, et al. Failure to differentiate Cryptosporidium parvum from C-meleagridis based on PCR amplification of eight DNA sequences. Applied and Environmental Microbiology. 1998;64(4):1454-8. PubMed PMID: WOS:000072894900045.

1154. Chang YF, Novosel V, Chang CF, Kim JB, Shin SJ, Lein DH. Detection of human granulocytic ehrlichiosis agent and Borrelia burgdorferi in ticks by polymerase chain reaction. J Vet Diagn Invest. 1998;10(1):56-9. Epub 1998/04/04. doi: 10.1177/104063879801000110. PubMed PMID: 9526861.

1155. Chang YF, Novosel V, Dubovi E, Wong SJ, Chu FK, Chang CF, et al. Experimental infection of the human granulocytic ehrlichiosis agent in horses. Vet Parasitol. 1998;78(2):137-45. Epub 1998/09/15. doi: 10.1016/s0304-4017(98)00133-2. PubMed PMID: 9735918.

1156. Cheong JPE, Brooker JD. Lysogenic bacteriophage M1 from Selenomonas ruminantium: Isolation, characterization and DNA sequence analysis of the integration site. Microbiology. 1998;144(8):2195-202. doi: 10.1099/00221287-144-8-2195.

1157. Chu FK. Rapid and sensitive PCR-based detection and differentiation of aetiologic agents of human granulocytotropic and monocytotropic ehrlichiosis. Mol Cell Probes. 1998;12(2):93-9. Epub 1998/06/20. doi: 10.1006/mcpr.1998.0150. PubMed PMID: 9633044.

1158. Cinco M, Padovan D, Murgia R, Heldtander M, Engvall EO. Detection of HGE agent-like Ehrlichia in Ixodes ricinus ticks in northern Italy by PCR. Wien Klin Wochenschr. 1998;110(24):898-900. Epub 1999/02/27. PubMed PMID: 10048173.

1159. Cinco M, Padovan D, Murgia R, Heldtander M, Olsson Engvall E. Detection of HGE agent-like Ehrlichia in Ixodes ricinus ticks in Northern Italy by PCR. Wiener Klinische Wochenschrift. 1998;110(24):898-900.

1160. Collins NE, De Villiers EP, Brayton KA, Allsopp BA. DNA sequence of a cosmid clone of Cowdria ruminantium. 1998. p. 365-8.

1161. Corcaci DC. [An update on human ehrlichiosis]. Rev Med Chir Soc Med Nat Iasi. 1998;102(3-4):65-8. Epub 2000/04/11. PubMed PMID: 10756846.

1162. Cotta MA, Whitehead TR. Xylooligosaccharide utilization by the ruminal anaerobic bacterium Selenomonas ruminantium. Current Microbiology. 1998;36(4):183-9. doi: 10.1007/s002849900291.

1163. Daniels TJ, Boccia TM, Varde S, Marcus J, Jianhua L, Bucher DJ, et al. Geographic risk for Lyme disease and human granulocytic ehrlichiosis in southern New York State. Applied and Environmental Microbiology. 1998;64(12):4663-9. doi: 10.1128/aem.64.12.4663-4669.1998.

1164. Daniels TJ, Boccia TM, Varde S, Marcus J, Le J, Bucher DJ, et al. Geographic risk for lyme disease and human granulocytic ehrlichiosis in southern New York state. Appl Environ Microbiol. 1998;64(12):4663-9. Epub 1998/12/03. doi: 10.1128/aem.64.12.4663-4669.1998. PubMed PMID: 9835546; PubMed Central PMCID: PMCPMC90906.

1165. Daniels TJ, Boccia TM, Varde S, Marcus J, Le JH, Bucher DJ, et al. Geographic risk for Lyme disease and human granulocytic ehrlichiosis in southern New York state. Applied and Environmental Microbiology. 1998;64(12):4663-9. PubMed PMID: WOS:000077396700006.

1166. Das S, Deponte K, Marcantonio NL, Ijdo JW, Hodzic E, Katavolos P, et al. Granulocytic ehrlichiosis in tick-immune guinea pigs. Infection and immunity. 1998;66(4):1803-5.

1167. Das S, Deponte K, Marcantonio NL, Ijdo JW, Hodzic E, Katavolos P, et al. Granulocytic ehrlichiosis in tick-immune guinea pigs. Infect Immun. 1998;66(4):1803-5. Epub 1998/04/07. doi: 10.1128/iai.66.4.1803-1805.1998. PubMed PMID: 9529119; PubMed Central PMCID: PMCPMC108126.

1168. Dighe AS, Shouche YS, Ranade DR. Selenomonas lipolytica sp. nov., an obligately anaerobic bacterium possessing lipolytic activity. Int J Syst Bacteriol. 1998;48 Pt 3:783-91. Epub 1998/09/12. doi: 10.1099/00207713-48-3-783. PubMed PMID: 9734032.

1169. Dumler JS. Ehrlichioses: emerging infections. Curr Opin Infect Dis. 1998;11(2):183-7. Epub 2006/10/13. PubMed PMID: 17033387.

1170. Dumler JS, Bakken JS. Human ehrlichioses: newly recognized infections transmitted by ticks. Annu Rev Med. 1998;49:201-13. Epub 1998/03/24. doi: 10.1146/annurev.med.49.1.201. PubMed PMID: 9509259.

1171. Dumler JS, Bakken JS. Human ehrlichioses: Newly recognized infections transmitted by ticks. 1998. p. 201-13.

1172. Dumler JS, Christova I. CD15 monoclonal antibodies and heparin sulfate abrogate Human Granulocytic Ehrlichiosis (HGE) agent infection of HL60 cells. FASEB Journal. 1998;12(5):A807.

1173. Dutta SK, Vemulapalli R, Biswas B. Association of deficiency in antibody response to vaccine and heterogeneity of Ehrlichia risticii strains with Potomac horse fever vaccine failure in horses. Journal of Clinical Microbiology. 1998;36(2):506-12. doi: 10.1128/jcm.36.2.506-512.1998.

1174. Duval X, Chosidow O, Tissot-Dupont H, Raoult D, Frances C. Cutaneous manifestations of rickettsiosis. Revue De Medecine Interne. 1998;19(8):548-57. doi: 10.1016/s0248-8663(99)80022-8. PubMed PMID: WOS:000075834500004.

1175. Edlow JA. Perinatal transmission of human granulocytic ehrlichiosis. N Engl J Med. 1998;339(26):1942-3. Epub 1999/01/05. PubMed PMID: 9874611.

1176. Egenvall A, Bjoersdorff A, Lilliehook I, Engvall EO, Karlstam E, Artursson K, et al. Early manifestations of granulocytic ehrlichiosis in dogs inoculated experimentally with a Swedish Ehrlichia species isolate. Veterinary Record. 1998;143(15):412-7. doi: 10.1136/vr.143.15.412. PubMed PMID: WOS:000076651800006.

1177. Egenvall A, Bjöersdorff A, Lilliehöök I, Olsson Engvall E, Karlstam E, Artursson K, et al. Early manifestations of granulocytic ehrlichiosis in dogs inoculated experimentally with a Swedish Ehrlichia species isolate. Veterinary Record. 1998;143(15):412-7. doi: 10.1136/vr.143.15.412.

1178. Elston DM. Perinatal transmission of human granulocytic ehrlichiosis. N Engl J Med. 1998;339(26):1941-2; author reply 2-3. Epub 1999/01/05. PubMed PMID: 9874610.

1179. Elston DM, Edlow JA, Horowitz H, Kilchevsky E. Perinatal transmission of human granulocytic ehrlichiosis [2] (multiple letters). New England Journal of Medicine. 1998;339(26):1941-3. doi: 10.1056/NEJM199812243392615.

1180. Eremeeva ME, Ching WM, Wu Y, Silverman DJ, Dasch GA. Western blotting analysis of heat shock proteins of Rickettsiales and other eubacteria. FEMS Microbiology Letters. 1998;167(2):229-37. doi: 10.1016/S0378-1097(98)00394-2.

1181. Evans J. Lyme disease. Curr Opin Rheumatol. 1998;10(4):339-46. Epub 1998/09/02. doi: 10.1097/00002281-199807000-00011. PubMed PMID: 9725096.

1182. Evans J, Evans J. Lyme disease. Current Opinion in Rheumatology. 1998;10(4):339-46. doi: 10.1097/00002281-199807000-00011. PubMed PMID: 107303281. Language: English. Entry Date: 19981201. Revision Date: 20210407. Publication Type: journal article.

1183. Foley JE, Barlough JE, Kimsey RB, Madigan JE, DeRock E, Poland A. Ehrlichia spp. in cervids from California. J Wildl Dis. 1998;34(4):731-7. Epub 1998/11/14. doi: 10.7589/0090-3558-34.4.731. PubMed PMID: 9813842.

1184. Freyer B, Eschenbacher KH, Mehlhorn H, Rueger W. Isolation and characterization of cDNA clones encoding a 32-kDa dense- granule antigen of Sarcocystis muris (Apicomplexa). Parasitology Research. 1998;84(7):583-9. doi: 10.1007/s004360050453.

1185. Fritz CL, Glaser CA. Ehrlichiosis. Infect Dis Clin North Am. 1998;12(1):123-36. Epub 1998/03/12. doi: 10.1016/s0891-5520(05)70413-x. PubMed PMID: 9494834.

1186. Fröhlich W, Edelhofer R. The first case of equine granulocytic ehrlichiosis (EGE) in an Austrian horse. Wiener Tierarztliche Monatsschrift. 1998;85(11):389-94.

1187. Gibbons A CL, Rigi FM, Awad-Ei-Kariem FM. Detection of cryptosporidium parvum and C. muris Oocysts in spiked backwash water using three PCR-based protocols. Protist. 1998;149(2):127-34. doi: 10.1016/s1434-4610(98)70017-3.

1188. Gibbons CL, Rigi FM, Awad-Ei-Kariem FM. Detection of Cryptosporidium parvum and C. muris Oocysts in Spiked Backwash Water using Three PCR-Based Protocols. Protist. 1998;149(2):127-34. Epub 1998/05/01. doi: 10.1016/s1434-4610(98)70017-3. PubMed PMID: 23196162.

1189. Gibbons CL, Rigi FM, Awad-El-Kariem FM. Detection of Cryptosporidium parvum and C-muris oocysts in spiked backwash water using three PCR-based protocols. Protist. 1998;149(2):127-34. doi: 10.1016/s1434-4610(98)70017-3. PubMed PMID: WOS:000074962800005.

1190. Goddard J. Residual effects of selected pyrethroid insecticides on lone star ticks. Journal of Environmental Health. 1998;61(4):19-22.

1191. Goddard J. Residual effects of selected pyrethroid insecticides on tone star ticks. Journal of Environmental Health. 1998;61(4):19-22. PubMed PMID: WOS:000076782100004.

1192. Goldman EE, Breitschwerdt EB, Grindem CB, Hegarty BC, Walls JJ, Dumler JS. Granulocytic ehrlichiosis in dogs from North Carolina and Virginia. J Vet Intern Med. 1998;12(2):61-70. Epub 1998/04/30. doi: 10.1111/j.1939-1676.1998.tb02096.x. PubMed PMID: 9560760.

1193. Guarino PD, Meek JI, Kusha B, Tenuta SW, Heimer R, Ryder RW, et al. Statewide surveillance for ehrlichiosis - Connecticut and New York, 1994-1997. JAMA. 1998;280(15):1300-1. doi: 10.1001/jama.280.15.1300.

1194. Guy E, Tasker S, Joynson DH. Detection of the agent of human granulocytic ehrlichiosis (HGE) in UK ticks using polymerase chain reaction. Epidemiol Infect. 1998;121(3):681-3. Epub 1999/02/25. doi: 10.1017/s0950268898001708. PubMed PMID: 10030718; PubMed Central PMCID: PMCPMC2809576.

1195. Guy E, Tasker S, Joynson DHM. Detection of the agent of human granulocytic ehrlichiosis (HGE) in UK ticks using polymerase chain reaction. Epidemiology and Infection. 1998;121(3):681-3. doi: 10.1017/S0950268898001708.

1196. Haas CN, Joffe J, Heath M, Jacangelo J, Anmangandla U. Predicting disinfection performance in continuous flow systems from batch disinfection kinetics. Water Science and Technology. 1998;38(6 pt 5):171-9. doi: 10.1016/S0273-1223(98)00579-4.

1197. Hansner T, Freyer B, Mehlhorn H, Rüger W. Amplification of genomic DNA fragments of Sarcocystis muris (Apicomplexa) cyst merozoites encoding a thiol (cysteine) proteinase. Parasitology Research. 1998;84(7):578-82. doi: 10.1007/s004360050452.

1198. Harrus S, Waner T, Aizenberg I, Bark H. Therapeutic effect of doxycycline in experimental subclinical canine monocytic ehrlichiosis: Evaluation of a 6-week course. Journal of Clinical Microbiology. 1998;36(7):2140-2. doi: 10.1128/jcm.36.7.2140-2142.1998.

1199. Harrus S, Waner T, Keysary A, Aroch I, Voet H, Bark H. Investigation of splenic functions in canine monocytic ehrlichiosis. Veterinary Immunology and Immunopathology. 1998;62(1):15-27. doi: 10.1016/S0165-2427(97)00127-X.

1200. Heilpern KL. Update: human ehrlichiosis--Maryland and Wisconsin, 1994. Ann Emerg Med. 1998;32(1):108-10. Epub 1998/07/10. doi: 10.1016/s0196-0644(98)70111-8. PubMed PMID: 9656961.

1201. Heilpern KL. Update: Human Ehrlichiosis - Maryland and Wisconsin, 1994. Annals of Emergency Medicine. 1998;32(1):108-10. doi: 10.1016/s0196-0644(98)70111-8.

1202. Heilpern KL, Heilpern KL. Update: human ehrlichiosis--Maryland and Wisconsin, 1994...including commentary by Heilpern KL. Annals of Emergency Medicine. 1998;32(1):108-10. PubMed PMID: 107217532. Language: English. Entry Date: 19991001. Revision Date: 20190814. Publication Type: journal article.

1203. Heimer R, Tisdale D, Dawson JE. A single tissue culture system for the propagation of the agents of the human ehrlichioses. Am J Trop Med Hyg. 1998;58(6):812-5. Epub 1998/07/11. doi: 10.4269/ajtmh.1998.58.812. PubMed PMID: 9660470.

1204. Hetrick SM. You're the flight surgeon. 35-year-old helicopter pilot with ehrlichiosis. Aviat Space Environ Med. 1998;69(12):1217-8. Epub 1998/12/18. PubMed PMID: 9856551.

1205. Hodzic E, Fish D, Maretzki CM, De Silva AM, Feng S, Barthold SW. Acquisition and transmission of the agent of human granulocytic ehrlichiosis by Ixodes scapularis ticks. J Clin Microbiol. 1998;36(12):3574-8. Epub 1998/11/18. doi: 10.1128/jcm.36.12.3574-3578.1998. PubMed PMID: 9817875; PubMed Central PMCID: PMCPMC105242.

1206. Hodzic E, Fish D, Maretzki CM, de Silva AM, Feng SL, Barthold SW. Acquisition and transmission of the agent of human granulocytic ehrlichiosis by Ixodes scapularis ticks. Journal of Clinical Microbiology. 1998;36(12):3574-8. doi: 10.1128/jcm.36.12.3574-3578.1998. PubMed PMID: WOS:000077069400024.

1207. Hodzic E, Ijdo JW, Feng S, Katavolos P, Sun W, Maretzki CH, et al. Granulocytic ehrlichiosis in the laboratory mouse. J Infect Dis. 1998;177(3):737-45. Epub 1998/03/14. doi: 10.1086/514236. PubMed PMID: 9498456.

1208. Hodzic E, Ijdo JWI, Feng S, Katavolos P, Sun W, Maretzki CH, et al. Granulocytic ehrlichiosis in the laboratory mouse. Journal of Infectious Diseases. 1998;177(3):737-45. doi: 10.1086/514236.

1209. Hodzic E, Ijdo JWI, Feng SL, Katavolos P, Sun W, Maretzki CH, et al. Granulocytic ehrlichiosis in the laboratory mouse. Journal of Infectious Diseases. 1998;177(3):737-45. doi: 10.1086/514236. PubMed PMID: WOS:000072159900030.

1210. Hofmeister EK, Kolbert CP, Abdulkarim AS, Magera JMH, Hopkins MK, Uhl JR, et al. Cosegregation of a novel Bartonella species with Borrelia burgdorferi and Babesia microti in Peromyscus leucopus. Journal of Infectious Diseases. 1998;177(2):409-16. doi: 10.1086/514201. PubMed PMID: WOS:000071801500019.

1211. Horowitz H, Kilchevsky E. Perinatal transmission of human granulocytic ehrlichiosis - Reply. New England Journal of Medicine. 1998;339(26):1942-3. PubMed PMID: WOS:000077696600020.

1212. Horowitz HW, Aguero-Rosenfeld M, Dumler JS, McKenna DF, Hsieh TC, Wu J, et al. Reinfection with the agent of human granulocytic ehrlichiosis. Ann Intern Med. 1998;129(6):461-3. Epub 1998/09/12. doi: 10.7326/0003-4819-129-6-199809150-00007. PubMed PMID: 9735084.

1213. Horowitz HW, Aguero-Rosenfeld M, Horowitz HW, Aguero-Rosenfeld M, Dumler JS, McKenna DF, et al. Reinfection with the agent of human granulocytic ehrlichiosis. Annals of Internal Medicine. 1998;129(6):461-3. doi: 10.7326/0003-4819-129-6-199809150-00007. PubMed PMID: 1125855. Language: English. Entry Date: 19981001. Revision Date: 20191111. Publication Type: journal article.

1214. Horowitz HW, Aguero-Rosenfeld ME, McKenna DF, Holmgren D, Hsieh TC, Varde SA, et al. Clinical and laboratory spectrum of culture-proven human granulocytic ehrlichiosis: comparison with culture-negative cases. Clin Infect Dis. 1998;27(5):1314-7. Epub 1998/11/25. doi: 10.1086/515000. PubMed PMID: 9827289.

1215. Horowitz HW, Kilchevsky E, Haber S, Aguero-Rosenfeld M, Kranwinkel R, James EK, et al. Perinatal transmission of the agent of human granulocytic ehrlichiosis. N Engl J Med. 1998;339(6):375-8. Epub 1998/08/06. doi: 10.1056/nejm199808063390604. PubMed PMID: 9691104.

1216. Horowitz HW, Raffalli J, Nadelman RB, Wu J, Wormser GP. Saddleback fever due to human granulocytic ehrlichiosis. Lancet. 1998;351(9103):650. Epub 1998/03/21. doi: 10.1016/s0140-6736(05)78434-8. PubMed PMID: 9500331.

1217. Horowitz HW, Wormser GP. Doxycycline revisited: An old medicine for emerging diseases. Archives of Internal Medicine. 1998;158(2):192-3. doi: 10.1001/archinte.158.2.192-a. PubMed PMID: WOS:000071556300014.

1218. Hsieh TC, DiPietrantonio A, Kumor A, Horowitz HW, Wormser GP. Attenuated responses to interferon (IFN) in HL-60 leukemia cells infected with the agent of Human Granulocytic Ehrlichiosis (HGE). FASEB Journal. 1998;12(5):A808.

1219. Hunfeld KP, Allwinn R, Peters S, Kraiczy P, Brade V. Serologic evidence for tick-borne pathogens other than Borrelia burgdorferi (TOBB) in Lyme borreliosis patients from midwestern Germany. Wien Klin Wochenschr. 1998;110(24):901-8. Epub 1999/02/27. PubMed PMID: 10048174.

1220. Ijdo JW, Fikrig E. [Human granulocytic ehrlichiosis, a tick-borne disease]. Ned Tijdschr Geneeskd. 1998;142(31):1778-81. Epub 1998/12/18. PubMed PMID: 9856144.

1221. Ijdo JW, Sun W, Zhang Y, Magnarelli LA, Fikrig E. Cloning of the gene encoding the 44-kilodalton antigen of the agent of human granulocytic ehrlichiosis and characterization of the humoral response. Infect Immun. 1998;66(7):3264-9. Epub 1998/06/25. doi: 10.1128/iai.66.7.3264-3269.1998. PubMed PMID: 9632594; PubMed Central PMCID: PMCPMC108341.

1222. Ijdo JW, Zhang Y, Anderson ML, Goldberg D, Fikrig E. Heat shock protein 70 of the agent of human granulocytic ehrlichiosis binds to Borrelia burgdorferi antibodies. Clin Diagn Lab Immunol. 1998;5(1):118-20. Epub 1998/02/10. doi: 10.1128/cdli.5.1.118-120.1998. PubMed PMID: 9455892; PubMed Central PMCID: PMCPMC121403.

1223. Ijdoen JW. Humane granulocytaire ehrlichiose, een ziekte overgebracht door teken. Nederlands Tijdschrift voor Geneeskunde. 1998;142(31):1778-81.

1224. Jahangir A, Kolbert C, Edwards W, Mitchell P, Dumler JS, Persing DH. Fatal pancarditis associated with human granulocytic Ehrlichiosis in a 44-year-old man. Clin Infect Dis. 1998;27(6):1424-7. Epub 1998/12/30. doi: 10.1086/515014. PubMed PMID: 9868655.

1225. Johnson EM, Ewing SA, Barker RW, Fox JC, Crow DW, Kocan KM. Experimental transmission of Ehrlichia canis (Rickettsiales : Ehrlichieae) by Dermacentor variabilis (Acari : Ixodidae). Veterinary Parasitology. 1998;74(2-4):277-88. doi: 10.1016/s0304-4017(97)00073-3. PubMed PMID: WOS:000072729500017.

1226. Katavolos P, Armstrong PM, Dawson JE, Telford Iii SR. Duration of tick attachment required for transmission of granulocytic ehrlichiosis. Journal of Infectious Diseases. 1998;177(5):1422-5. doi: 10.1086/517829.

1227. Katavolos P, Armstrong PM, Dawson JE, Telford SR, 3rd. Duration of tick attachment required for transmission of granulocytic ehrlichiosis. J Infect Dis. 1998;177(5):1422-5. Epub 1998/05/21. doi: 10.1086/517829. PubMed PMID: 9593039.

1228. Kaye G, Weber P, Evans A, Venezia R. Efficacy of Alkaline Hydrolysis as an Alternative Method for Treatment and Disposal of Infectious Animal Waste. Contemp Top Lab Anim Sci. 1998;37(3):43-6. Epub 2002/11/29. PubMed PMID: 12456159.

1229. Keirans JE, Lacombe EH. First records of Amblyomma americanum, Ixodes (Ixodes) dentatus, and Ixodes (Ceratixodes) uriae (Acari: Ixodidae) from Maine. J Parasitol. 1998;84(3):629-31. Epub 1998/06/30. PubMed PMID: 9645873.

1230. Kelly PJ, Matthewman LA, Brouqui P, Raoult D. Lack of susceptibility of Ehrlichia canis to imidocarb dipropionate in vitro. Journal of the South African Veterinary Association. 1998;69(2):55-6. doi: 10.4102/jsava.v69i2.815.

1231. Kilani M, Dorchies P. The importance and prevalence of diseases according to their geographic origin-North Africa-Mediterranean regions and the Middle East. Point Veterinaire. 1998;29(194):37-43.

1232. Kilani M, Dorchies P. The importance and prevalence of diseases according to their geographic origin - North Africa Mediterranean regions and the Middle East. Point Veterinaire. 1998;29(194):37-43. PubMed PMID: WOS:000077030100009.

1233. Kim HY, Rikihisa Y. Characterization of monoclonal antibodies to the 44-kilodalton major outer membrane protein of the human granulocytic ehrlichiosis agent. J Clin Microbiol. 1998;36(11):3278-84. Epub 1998/10/17. doi: 10.1128/jcm.36.11.3278-3284.1998. PubMed PMID: 9774579; PubMed Central PMCID: PMCPMC105315.

1234. Klein H, Löschner B, Zyto N, Pörtner M, Montag T. Expression, purification, and biochemical characterization of a recombinant lectin of Sarcocystis muris (Apicomplexa) cyst merozoites. Glycoconj J. 1998;15(2):147-53. Epub 1998/04/29. doi: 10.1023/a:1006964105349. PubMed PMID: 9557874.

1235. Klein MB, Hayes SF, Goodman JL. Monocytic differentiation inhibits infection and granulocytic differentiation potentiates infection by the agent of human granulocytic ehrlichiosis. Infect Immun. 1998;66(7):3410-5. Epub 1998/06/25. doi: 10.1128/iai.66.7.3410-3415.1998. PubMed PMID: 9632613; PubMed Central PMCID: PMCPMC108360.

1236. Kobayashi Y, Okuda N, Matsumoto M, Inoue K, Wakita M, Hoshino S. Constitutive expression of a heterologous Eubacterium ruminantium xylanase gene (xynA) in Butyrivibrio fibrisolvens. FEMS Microbiology Letters. 1998;163(1):11-7. doi: 10.1016/S0378-1097(98)00135-9.

1237. Lebech AM, Hansen K, Pancholi P, Sloan LM, Magera JM, Persing DH. Immunoserologic evidence of Human Granulocytic Ehrlichiosis in Danish patients with Lyme neuroborreliosis. Scand J Infect Dis. 1998;30(2):173-6. Epub 1998/09/08. doi: 10.1080/003655498750003582. PubMed PMID: 9730306.

1238. Lee EH, Rikihisa Y. Protein kinase A-mediated inhibition of gamma interferon-induced tyrosine phosphorylation of Janus kinases and latent cytoplasmic transcription factors in human monocytes by Ehrlichia chaffeensis. Infect Immun. 1998;66(6):2514-20. Epub 1998/05/29. doi: 10.1128/iai.66.6.2514-2520.1998. PubMed PMID: 9596710; PubMed Central PMCID: PMCPMC108232.

1239. Lee EH, Rikihisa Y. Protein kinase A mediated inhibition of gamma interferon-induced tyrosine phosphorylation of Janus kinases and latent cytoplasmic transcription factors in human monocytes by Ehrlichia chaffeensis. Infection and Immunity. 1998;66(6):2514-20. doi: 10.1128/iai.66.6.2514-2520.1998. PubMed PMID: WOS:000073781100016.

1240. Lenártová V, Holovská K, Javorský P. The influence of mercury on the antioxidant enzyme activity of rumen bacteria Streptococcus bovis and Selenomonas ruminantium. FEMS Microbiology Ecology. 1998;27(4):319-25. doi: 10.1016/S0168-6496(98)00077-4.

1241. Lillieh, oulm, oulm, k I, Egenvall A, Tvedten HW. Hematopathology in dogs experimentally infected with a Swedish granulocytic Ehrlichia species. Vet Clin Pathol. 1998;27(4):116-22. Epub 2002/06/21. doi: 10.1111/j.1939-165x.1998.tb01030.x. PubMed PMID: 12075539.

1242. Little SE, Stallknecht DE, Lockhart JM, Dawson JE, Davidson WR. Natural coinfection of a white-tailed deer (Odocoileus virginianus) population with three Ehrlichia spp. Journal of Parasitology. 1998;84(5):897-901. doi: 10.2307/3284616.

1243. Lockhart JM, Davidson WR, Stallknecht DE, Dawson JE. Lack of seroreactivity to Ehrlichia chaffeensis among rodent populations. Journal of Wildlife Diseases. 1998;34(2):392-6. doi: 10.7589/0090-3558-34.2.392. PubMed PMID: WOS:000073228900026.

1244. Lotric-Furlan S, Petrovec M, Avsic-Zupanc T, Nicholson WL, Sumner JW, Childs JE, et al. Human ehrlichiosis in central Europe. Wien Klin Wochenschr. 1998;110(24):894-7. Epub 1999/02/27. PubMed PMID: 10048172.

1245. Lotric-Furlan S, Petrovec M, Avsic-Zupanc T, Strle F. Ehrlichia antibodies, leukopenia and thrombocytopenia in initial phase of tick-borne encephalitis. Infection. 1998;26(4):253. Epub 1998/08/26. doi: 10.1007/bf02962378. PubMed PMID: 9717688.

1246. Lotric-Furlan S, Petrovec M, Avsic-Zupanc T, Strle F. Ehrlichia antibodies, leukopenia and thrombocytopenia in initial phase of tick-borne encephalitis [3]. Infection. 1998;26(4):253. doi: 10.1007/BF02962378.

1247. Lotric-Furlan S, Petrovec M, Zupanc TA, Nicholson WL, Sumner JW, Childs JE, et al. Human granulocytic ehrlichiosis in Europe: clinical and laboratory findings for four patients from Slovenia. Clin Infect Dis. 1998;27(3):424-8. Epub 1998/10/14. doi: 10.1086/514683. PubMed PMID: 9770134.

1248. Macechko PT, van Keulen H, Jarroll EL, Mulgrew T, Gurien A, Erlandsen SL. Detection of Giardia Trophozoites in Archival Pathology Specimens of Human Small Intestine. Microsc Microanal. 1998;4(4):397-403. Epub 1999/05/18. doi: 10.1017/s1431927698980370. PubMed PMID: 9882715.

1249. Magnarelli LA, Ijdo JW, Anderson JF, Padula SJ, Flavell RA, Fikrig E. Human exposure to a granulocytic Ehrlichia and other tick-borne agents in Connecticut. J Clin Microbiol. 1998;36(10):2823-7. Epub 1998/09/17. doi: 10.1128/jcm.36.10.2823-2827.1998. PubMed PMID: 9738027; PubMed Central PMCID: PMCPMC105071.

1250. Magnarelli LA, Ijdo JW, Dumler JS, Heimer R, Fikrig E. Reactivity of human sera to different strains of granulocytic ehrlichiae in immunodiagnostic assays. Journal of Infectious Diseases. 1998;178(6):1835-8. doi: 10.1086/314516.

1251. Magnarelli LA, JW IJ, Dumler JS, Heimer R, Fikrig E. Reactivity of human sera to different strains of granulocytic ehrlichiae in immunodiagnostic assays. J Infect Dis. 1998;178(6):1835-8. Epub 1998/11/17. doi: 10.1086/314516. PubMed PMID: 9815246.

1252. Mahbubani MH, Schaefer Iii FW, Jones DD, Bej AK. Detection of Giardia in environmental waters by immuno-PCR amplification methods. Current Microbiology. 1998;36(2):107-13. doi: 10.1007/s002849900288.

1253. Mahfouz NM, Aboul-Fadl T, Diab AK. Metronidazole twin ester prodrugs: Synthesis, physicochemical properties, hydrolysis kinetics and antigiardial activity. European Journal of Medicinal Chemistry. 1998;33(9):675-83. doi: 10.1016/S0223-5234(98)80026-3.

1254. Massung RF, Slater K, Owens JH, Nicholson WL, Mather TN, Solberg VB, et al. Nested PCR assay for detection of granulocytic ehrlichiae. J Clin Microbiol. 1998;36(4):1090-5. Epub 1998/05/23. doi: 10.1128/jcm.36.4.1090-1095.1998. PubMed PMID: 9542943; PubMed Central PMCID: PMCPMC104695.

1255. McGill SL, Regnery RL, Karem KL. Characterization of human immunoglobulin (Ig) isotype and IgG subclass response to Bartonella henselae infection. Infect Immun. 1998;66(12):5915-20. Epub 1998/11/24. doi: 10.1128/iai.66.12.5915-5920.1998. PubMed PMID: 9826373; PubMed Central PMCID: PMCPMC108749.

1256. Meinkoth JH, Ewing SA, Cowell RL, Dawson JE, Warner CK, Mathew JS, et al. Morphologic and molecular evidence of a dual species ehrlichial infection in a dog presenting with inflammatory central nervous system disease. Journal of Veterinary Internal Medicine. 1998;12(5):389-93. doi: 10.1111/j.1939-1676.1998.tb02140.x. PubMed PMID: WOS:000075931300012.

1257. Monis PT, Andrews RH, Mayrhofer G, Mackrill J, Kulda J, Isaac-Renton JL. Novel lineages of Giardia intestinalis identified by genetic analysis of organisms isolated from dogs in Australia. Parasitology. 1998;116(1):7-19. doi: 10.1017/S0031182097002011.

1258. Monis PT, Andrews RH, Mayrhofer G, Mackrill J, Kulda J, Isaac-Renton JL, et al. Novel lineages of Giardia intestinalis identified by genetic analysis of organisms isolated from dogs in Australia. Parasitology. 1998;116 ( Pt 1):7-19. Epub 1998/03/03. doi: 10.1017/s0031182097002011. PubMed PMID: 9481769.

1259. Moody EK, Barker RW, White JL, Crutcher JM. Ticks and tick-borne diseases in Oklahoma. J Okla State Med Assoc. 1998;91(8):438-45. Epub 1998/11/26. PubMed PMID: 9828526.

1260. Morgan UM, Sargent KD, Deplazes P, Forbes DA, Spano F, Hertzberg H, et al. Molecular characterization of Cryptosporidium from various hosts. Parasitology. 1998;117 ( Pt 1):31-7. Epub 1998/08/08. doi: 10.1017/s0031182098002765. PubMed PMID: 9695098.

1261. Morgan UM, Sargent KD, Deplazes P, Forbes DA, Spano F, Hertzberg H, et al. Molecular characterization of Cryptosporidium from various hosts. Parasitology. 1998;117(1):31-7. doi: 10.1017/S0031182098002765.

1262. Murphy CI, Storey JR, Recchia J, Doros-Richert LA, Gingrich-Baker C, Munroe K, et al. Major antigenic proteins of the agent of human granulocytic ehrlichiosis are encoded by members of a multigene family. Infect Immun. 1998;66(8):3711-8. Epub 1998/07/23. doi: 10.1128/iai.66.8.3711-3718.1998. PubMed PMID: 9673253; PubMed Central PMCID: PMCPMC108406.

1263. Murphy GL, Ewing SA, Whitworth LC, Fox JC, Kocan AA. A molecular and serologic survey of Ehrlichia canis, E. chaffeensis, and E. ewingii in dogs and ticks from Oklahoma. Vet Parasitol. 1998;79(4):325-39. Epub 1998/12/01. doi: 10.1016/s0304-4017(98)00179-4. PubMed PMID: 9831955.

1264. Murphy GL, Ewing SA, Whitworth LC, Fox JC, Kocan AA. A molecular and serologic survey of Ehrlichia canis, E-chaffeensis, and E-ewingii in dogs and ticks from Oklahoma. Veterinary Parasitology. 1998;79(4):325-39. doi: 10.1016/s0304-4017(98)00179-4. PubMed PMID: WOS:000076995800006.

1265. Nicholson WL, Muir S, Sumner JW, Childs JE. Serologic evidence of infection with Ehrlichia spp. in wild rodents (Muridae: Sigmodontinae) in the United States. J Clin Microbiol. 1998;36(3):695-700. Epub 1998/03/21. doi: 10.1128/jcm.36.3.695-700.1998. PubMed PMID: 9508298; PubMed Central PMCID: PMCPMC104611.

1266. Noden BH, Radulovic S, Higgins JA, Azad AF. Molecular identification of Rickettsia typhi and R-felis in co-infected Ctenocephalides felis (Siphonaptera : Pulicidae). Journal of Medical Entomology. 1998;35(4):410-4. doi: 10.1093/jmedent/35.4.410. PubMed PMID: WOS:000075274000010.

1267. Nuti M, Serafini DA, Bassetti D, Ghionni A, Russino F, Rombolà P, et al. Ehrlichia infection in Italy. Emerg Infect Dis. 1998;4(4):663-5. Epub 1998/12/29. doi: 10.3201/eid0404.980420. PubMed PMID: 9866746; PubMed Central PMCID: PMCPMC2640256.

1268. Nyika A, Mahan SM, Burridge MJ, McGuire TC, Rurangirwa F, Barbet AF. A DNA vaccine protects mice against the rickettsial agent Cowdria ruminantium. Parasite Immunology. 1998;20(3):111-9. doi: 10.1046/j.1365-3024.1998.00120.x.

1269. Ogden NH, Bown K, Horrocks BK, Woldehiwet Z, Bennett M. Granulocytic Ehrlichia infection in ixodid ticks and mammals in woodlands and uplands of the U.K. Med Vet Entomol. 1998;12(4):423-9. Epub 1998/11/24. doi: 10.1046/j.1365-2915.1998.00133.x. PubMed PMID: 9824827.

1270. Ogden NH, Woldehiwet Z, Hart CA. Granulocytic ehrlichiosis: an emerging or rediscovered tick-borne disease? J Med Microbiol. 1998;47(6):475-82. Epub 1999/01/08. doi: 10.1099/00222615-47-6-475. PubMed PMID: 9879965.

1271. Ohashi N, Unver A, Zhi N, Rikihisa Y. Cloning and characterization of multigenes encoding the immunodominant 30-kilodalton major outer membrane proteins of Ehrlichia canis and application of the recombinant protein for serodiagnosis. J Clin Microbiol. 1998;36(9):2671-80. Epub 1998/08/15. doi: 10.1128/jcm.36.9.2671-2680.1998. PubMed PMID: 9705412; PubMed Central PMCID: PMCPMC105182.

1272. Ohashi N, Zhi N, Zhang Y, Rikihisa Y. Immunodominant major outer membrane proteins of Ehrlichia chaffeensis are encoded by a polymorphic multigene family. Infect Immun. 1998;66(1):132-9. Epub 1998/01/10. doi: 10.1128/iai.66.1.132-139.1998. PubMed PMID: 9423849; PubMed Central PMCID: PMCPMC107868.

1273. Parola P, Beati L, Cambon M, Brouqui P, Raoult D. Ehrlichial DNA amplified from Ixodes ricinus (Acari: Ixodidae) in France. J Med Entomol. 1998;35(2):180-3. Epub 1998/04/16. doi: 10.1093/jmedent/35.2.180. PubMed PMID: 9538582.

1274. Pennock JL, Behnke JM, Bickle QD, Devaney E, Grencis RK, Isaac RE, et al. Rapid purification and characterization of L-dopachrome-methyl ester tautomerase (macrophage-migration-inhibitory factor) from Trichinella spiralis, Trichuris muris and Brugia pahangi. Biochem J. 1998;335 ( Pt 3)(Pt 3):495-8. Epub 1998/10/31. doi: 10.1042/bj3350495. PubMed PMID: 9794786; PubMed Central PMCID: PMCPMC1219807.

1275. Pennock JL, Behnke JM, Bickle QD, Devaney E, Grencis RK, Isaac RE, et al. Rapid purification and characterization of L-dopachrome-methyl ester tautomerase (macrophage-migration-inhibitory factor) from Trichinella spiralis, Trichuris muris and Brugia pahangi. Biochemical Journal. 1998;335(3):495-8. doi: 10.1042/bj3350495.

1276. Pennock JL, Behnke JM, Bickle QD, Devaney E, Grencis RK, Isaac RE, et al. Rapid purification and characterization of L-dopachrome-methyl ester tautomerase (macrophage-migration-inhibitory factor) from Trichinella spiralis, Trichuris muris and Brugia pahangi. Biochemical Journal. 1998;335:495-8. doi: 10.1042/bj3350495. PubMed PMID: WOS:000076980300004.

1277. Platt-Samoraj A, Szweda W, Ciecierski H. Ehrlichiosis in the light of the latest research. Medycyna Weterynaryjna. 1998;54(6):363-7. PubMed PMID: WOS:000073891500001.

1278. Popov VL, Han VC, Chen SM, Dumler JS, Feng HM, Andreadis TG, et al. Ultrastructural differentiation of the genogroups in the genus Ehrlichia. J Med Microbiol. 1998;47(3):235-51. Epub 1998/03/25. doi: 10.1099/00222615-47-3-235. PubMed PMID: 9511829.

1279. Pruitt AA. Infections of the nervous system. Neurologic Clinics. 1998;16(2):419-47. doi: 10.1016/S0733-8619(05)70071-2.

1280. Pusterla N, Huder JB, Feige K, Lutz H. Identification of a granulocytic Ehrlichia strain isolated from a horse in Switzerland and comparison with other rickettsiae of the Ehrlichia phagocytophila genogroup. J Clin Microbiol. 1998;36(7):2035-7. Epub 1998/07/03. doi: 10.1128/jcm.36.7.2035-2037.1998. PubMed PMID: 9650957; PubMed Central PMCID: PMCPMC104973.

1281. Pusterla N, Huder JB, Lutz H, Braun U. Detection of Ehrlichia phagocytophila DNA in Ixodes ricinus ticks from areas in Switzerland where tick-borne fever is endemic. Journal of Clinical Microbiology. 1998;36(9):2735-6. doi: 10.1128/jcm.36.9.2735-2736.1998. PubMed PMID: WOS:000075420800064.

1282. Pusterla N, Weber R, Wolfensberger C, Schär G, Zbinden R, Fierz W, et al. Serological evidence of human granulocytic ehrlichiosis in Switzerland. Eur J Clin Microbiol Infect Dis. 1998;17(3):207-9. Epub 1998/07/17. doi: 10.1007/bf01691120. PubMed PMID: 9665305.

1283. Radetsky M. The emerging spectrum of tickborne infections. Current Opinion in Infectious Diseases. 1998;11(3):313-8. doi: 10.1097/00001432-199806000-00008. PubMed PMID: WOS:000073822700010.

1284. Ravyn MD, Goodman JL, Kodner CB, Westad DK, Coleman LA, Engstrom SM, et al. Immunodiagnosis of human granulocytic ehrlichiosis by using culture-derived human isolates. J Clin Microbiol. 1998;36(6):1480-8. Epub 1998/06/10. doi: 10.1128/jcm.36.6.1480-1488.1998. PubMed PMID: 9620365; PubMed Central PMCID: PMCPMC104863.

1285. Reddy GR, Sulsona CR, Barbet AF, Mahan SM, Burridge MJ, Alleman AR. Molecular characterization of a 28 kDa surface antigen gene family of the tribe Ehrlichiae. Biochem Biophys Res Commun. 1998;247(3):636-43. Epub 1998/07/02. doi: 10.1006/bbrc.1998.8844. PubMed PMID: 9647746.

1286. Reubel GH, Kimsey RB, Barlough JE, Madigan JE. Experimental transmission of Ehrlichia equi to horses through naturally infected ticks (Ixodes pacificus) from Northern California. Journal of Clinical Microbiology. 1998;36(7):2131-4. doi: 10.1128/jcm.36.7.2131-2134.1998.

1287. Roland WE, Everett ED, Cyr TL, Hasan SZ, Dommaraju CB, McDonald GA. Ehrlichia chaffeensis in Missouri ticks. American Journal of Tropical Medicine and Hygiene. 1998;59(4):641-3. doi: 10.4269/ajtmh.1998.59.641.

1288. Sainz A, Amusategui I, Tesouro MA. Canine ehrlichiosis in the Comunidad de Madrid in central Spain. 1998. p. 438-40.

1289. Santino I, Iori A, Sessa R, Sulli C, Favia G, Del Piano M. Borrelia burgdorferi s.l. and Ehrlichia chaffeensis in the National Park of Abruzzo. FEMS Microbiol Lett. 1998;164(1):1-6. Epub 1998/07/24. doi: 10.1111/j.1574-6968.1998.tb13059.x. PubMed PMID: 9675844.

1290. Schauber EM, Gertz SJ, Maple WT, Ostfeld RS. Coinfection of blacklegged ticks (Acari: Ixodidae) in Dutchess County, New York, with the agents of Lyme disease and human granulocytic ehrlichiosis. J Med Entomol. 1998;35(5):901-3. Epub 1998/10/17. doi: 10.1093/jmedent/35.5.901. PubMed PMID: 9775627.

1291. Sexton DJ, Corey GR, Carpenter C, Kong LQ, Gandhi T, Breitschwerdt E, et al. Dual infection with Ehrlichia chaffeensis and a spotted fever group rickettsia: a case report. Emerg Infect Dis. 1998;4(2):311-6. Epub 1998/06/11. doi: 10.3201/eid0402.980222. PubMed PMID: 9621205; PubMed Central PMCID: PMCPMC2640120.

1292. Sigal LH. Musculoskeletal manifestations of Lyme arthritis. Rheumatic Disease Clinics of North America. 1998;24(2):323-+. doi: 10.1016/s0889-857x(05)70012-0. PubMed PMID: WOS:000073676700009.

1293. Smith GE, Anderson EC, Burridge MJ, Peter TF, Mahan SM. Growth of Cowdria ruminantium in tissue culture endothelial cell lines from wild African mammals. J Wildl Dis. 1998;34(2):297-304. Epub 1998/05/13. doi: 10.7589/0090-3558-34.2.297. PubMed PMID: 9577776.

1294. Stafford Iii KC, Ward JS, Magnarelli LA. Impact of Controlled Burns on the Abundance of Ixodes scapularis (Acari: Ixodidae). Journal of Medical Entomology. 1998;35(4):510-3. doi: 10.1093/jmedent/35.4.510.

1295. Stafford KC, Ward JS, Magnarelli LA. Impact of controlled burns on the abundance of Ixodes scapularis (Acari : Ixodidae). Journal of Medical Entomology. 1998;35(4):510-3. doi: 10.1093/jmedent/35.4.510. PubMed PMID: WOS:000075274000027.

1296. Storey JR, Doros-Richert LA, Gingrich-Baker C, Munroe K, Mather TN, Coughlin RT, et al. Molecular cloning and sequencing of three granulocytic Ehrlichia genes encoding high-molecular-weight immunoreactive proteins. Infect Immun. 1998;66(4):1356-63. Epub 1998/04/07. doi: 10.1128/iai.66.4.1356-1363.1998. PubMed PMID: 9529053; PubMed Central PMCID: PMCPMC108060.

1297. Stuen S, Artursson K, Engvall EO. Experimental Infection of Lambs with an Equine Granulocytic Ehrlichia Species Resembling the Agent that Causes Human Granulocytic Ehrlichiosis (HGE). Acta Veterinaria Scandinavica. 1998;39(4):491-7. doi: 10.1186/BF03547775.

1298. Stuen S, Artursson K, Olsson Engvall E. Experimental infection of lambs with an equine granulocytic Ehrlichia species resembling the agent that causes human granulocytic ehrlichiosis (HGE). Acta Vet Scand. 1998;39(4):491-7. Epub 1999/02/02. doi: 10.1186/bf03547775. PubMed PMID: 9926463; PubMed Central PMCID: PMCPMC8050659.

1299. Stuen S, Engvall EO, Artursson K. Persistence of Ehrlichia phagocytophila infection in lambs in relation to clinical parameters and antibody responses. Veterinary Record. 1998;143(20):553-5. doi: 10.1136/vr.143.20.553. PubMed PMID: WOS:000077197200009.

1300. Sulzer AJ. Dual infection with Ehrlichia chaffeensis and a spotted fever group rickettsia: a case report. Emerg Infect Dis. 1998;4(4):705-6. Epub 1998/12/29. doi: 10.3201/eid0404.980430. PubMed PMID: 9866756; PubMed Central PMCID: PMCPMC2640241.

1301. Sulzer AJ, Sexton DJ, Walker DH. Dual infection with Ehrlichia chaffeensis and a spotted fever group rickettsia: A case report [3] (multiple letters). Emerging Infectious Diseases. 1998;4(4):706. doi: 10.3201/eid0404.980431.

1302. Sweeney CJ, Ghassemi M, Agger WA, Persing DH. Coinfection with Babesia microti and Borrelia burgdorferi in a western Wisconsin resident. Mayo Clinic Proceedings. 1998;73(4):338-41. PubMed PMID: WOS:000072992800006.

1303. Tälleklint L, Jaenson TGT. Increasing Geographical Distribution and Density of Ixodes ricinus (Acari: Ixodidae) in Central and Northern Sweden. Journal of Medical Entomology. 1998;35(4):521-6. doi: 10.1093/jmedent/35.4.521.

1304. Thomas DR, Sillis M, Coleman TJ, Kench SM, Ogden NH, Salmon RL, et al. Low rates of ehrlichiosis and Lyme borreliosis in English farmworkers. Epidemiol Infect. 1998;121(3):609-14. Epub 1999/02/25. doi: 10.1017/s0950268898001514. PubMed PMID: 10030710; PubMed Central PMCID: PMCPMC2809568.

1305. Totté P, Nyanjui J, Bensaid A, McKeever D. The use of CD4+ T-cell lines to screen for immunogenic proteins of Cowdria ruminantium. 1998. p. 375-7.

1306. Vachiéry N, Trap I, Totté P, Martinez D, Bensaid A. Inhibition of MHC class I and class II cell surface expression on bovine endothelial cells upon infection with Cowdria ruminantium. Veterinary Immunology and Immunopathology. 1998;61(1):37-48. doi: 10.1016/S0165-2427(97)00129-3.

1307. Van Andel AE, Magnarelli LA, Heimer R, Wilson ML. Development and duration of antibody response against Ehrlichia equi in horses. Journal of the American Veterinary Medical Association. 1998;212(12):1910-+. PubMed PMID: WOS:000074230000029.

1308. van Keulen H, Feely DE, Macechko PT, Jarroll EL, Erlandsen SL. The sequence of Giardia small subunit rRNA shows that voles and muskrats are parasitized by a unique species Giardia microti. Journal of Parasitology. 1998;84(2):294-300. doi: 10.2307/3284485. PubMed PMID: WOS:000073273600013.

1309. Varde S, Beckley J, Schwartz I. Prevalence of tick-borne pathogens in Ixodes scapularis in a rural New Jersey County. Emerg Infect Dis. 1998;4(1):97-9. Epub 1998/03/21. doi: 10.3201/eid0401.980113. PubMed PMID: 9452402; PubMed Central PMCID: PMCPMC2627663.

1310. Vemulapalli R, Biswas B, Dutta SK. Cloning and molecular analysis of genes encoding two immunodominant antigens of Ehrlichia risticii. Microbial Pathogenesis. 1998;24(6):361-72. doi: 10.1006/mpat.1998.0208.

1311. Vesey G, Ashbolt N, Fricker EJ, Deere D, Williams KL, Veal DA, et al. The use of a ribosomal RNA targeted oligonucleotide probe for fluorescent labelling of viable Cryptosporidium parvum oocysts. Journal of Applied Microbiology. 1998;85(3):429-40. doi: 10.1046/j.1365-2672.1998.853496.x.

1312. Walker DH. Tick-transmitted infectious diseases in the United States. Annu Rev Public Health. 1998;19:237-69. Epub 1998/06/05. doi: 10.1146/annurev.publhealth.19.1.237. PubMed PMID: 9611619.

1313. Wallace BJ, Brady G, Ackman DM, Wong SJ, Jacquette G, Lloyd EE, et al. Human granulocytic ehrlichiosis in New York. Arch Intern Med. 1998;158(7):769-73. Epub 1998/04/29. doi: 10.1001/archinte.158.7.769. PubMed PMID: 9554683.

1314. Walls JJ, Asanovich KM, Bakken JS, Dumler JS. Serologic evidence of a natural infection of white-tailed deer with the agent of human granulocytic ehrlichiosis in Wisconsin and Maryland. Clin Diagn Lab Immunol. 1998;5(6):762-5. Epub 1998/11/05. doi: 10.1128/cdli.5.6.762-765.1998. PubMed PMID: 9801331; PubMed Central PMCID: PMCPMC96198.

1315. Walls JJ, Asanovich KM, Bakken JS, Stephen Dumler J. Serologic evidence of a natural infection of white-tailed deer with the agent of human granulocytic ehrlichiosis in Wisconsin and Maryland. Clinical and Diagnostic Laboratory Immunology. 1998;5(6):762-5. doi: 10.1128/cdli.5.6.762-765.1998.

1316. Waner T, Strenger C, Keysary A, Harrus S. Kinetics of serologic cross-reactions between Ehrlichia canis and the Ehrlichia phagocytophila genogroups in experimental E-canis infection in dogs. Veterinary Immunology and Immunopathology. 1998;66(3-4):237-43. doi: 10.1016/s0165-2427(98)00198-6. PubMed PMID: WOS:000077693100003.

1317. Weber R, Pusterla N, Loy M, Lutz H. Fever, leukopenia, and thrombocytopenia in a patient with acute Lyme borreliosis were due to human granulocytic ehrlichiosis. Clin Infect Dis. 1998;26(1):253-4. Epub 1998/02/10. doi: 10.1086/517052. PubMed PMID: 9455582.

1318. Weber R, Pusterla N, Loy M, Lutz H. Fever, leukopenia, and thrombocytopenia in a patient with acute Lyme borreliosis were due to human granulocytic ehrlichiosis [9] (multiple letters). Clinical Infectious Diseases. 1998;26(1):253-4. doi: 10.1086/517052.

1319. Webster P, Ijdo JW, Chicoine LM, Fikrig E. The agent of human granulocytic ehrlichiosis resides in an endosomal compartment. Journal of Clinical Investigation. 1998;101(9):1932-41. doi: 10.1172/JCI1544.

1320. Webster P, JW IJ, Chicoine LM, Fikrig E. The agent of Human Granulocytic Ehrlichiosis resides in an endosomal compartment. J Clin Invest. 1998;101(9):1932-41. Epub 1998/06/13. doi: 10.1172/jci1544. PubMed PMID: 9576758; PubMed Central PMCID: PMCPMC508780.

1321. White DJ, Talarico J, Chang HG, Birkhead GS, Heimberger T, Morse DL. Human babesiosis in New York State: Review of 139 hospitalized cases and analysis of prognostic factors. Arch Intern Med. 1998;158(19):2149-54. Epub 1998/11/04. doi: 10.1001/archinte.158.19.2149. PubMed PMID: 9801183.

1322. White DJ, Talarico J, Chang HG, Birkhead GS, Heimberger T, Morse DL. Human babesiosis in New York State - Review of 139 hospitalized cases and analysis of prognostic factors. Archives of Internal Medicine. 1998;158(19):2149-54. doi: 10.1001/archinte.158.19.2149. PubMed PMID: WOS:000076577500010.

1323. Winslow GM, Yager E, Shilo K, Collins DN, Chu FK. Infection of the laboratory mouse with the intracellular pathogen Ehrlichia chaffeensis. Infect Immun. 1998;66(8):3892-9. Epub 1998/07/23. doi: 10.1128/iai.66.8.3892-3899.1998. PubMed PMID: 9673277; PubMed Central PMCID: PMCPMC108445.

1324. Wong SJ, Thomas JA. Cytoplasmic, nuclear, and platelet autoantibodies in human granulocytic ehrlichiosis patients. J Clin Microbiol. 1998;36(7):1959-63. Epub 1998/07/03. doi: 10.1128/jcm.36.7.1959-1963.1998. PubMed PMID: 9650944; PubMed Central PMCID: PMCPMC104960.

1325. Wormser GP, Nowakowski J, Nadelman RB, Schwartz I, McKenna D, Holmgren D, et al. Efficacy of an OspA vaccine preparation for prevention of Lyme disease in New York state. Infection. 1998;26(4):208-12. doi: 10.1007/bf02962365. PubMed PMID: WOS:000075228400003.

1326. Wu JM, Hsieh TC, DiPietrantonio AD, Kumor A, Horowitz HW, Wormser GP. Attenuated responses to interferon (IFN) in HL-60 leukemia cells infected with the agent of human granulocytic ehrlichiosis (HGE). Faseb Journal. 1998;12(5):A808-A. PubMed PMID: WOS:000076006501067.

1327. Xiao L, Sulaiman I, Fayer R, Lal AA. Species and strain-specific typing of Cryptosporidium parasites in clinical and environmental samples. Mem Inst Oswaldo Cruz. 1998;93(5):687-91. Epub 1998/11/27. doi: 10.1590/s0074-02761998000500022. PubMed PMID: 9830539.

1328. Yanke LJ, Bae HD, Selinger LB, Cheng KJ. Phytase activity of anaerobic ruminal bacteria. Microbiology. 1998;144(6):1565-73. doi: 10.1099/00221287-144-6-1565.

1329. Zhang Y, Ohashi N, Rikihisa Y. Cloning of the heat shock protein 70 (HSP70) gene of Ehrlichia sennetsu and differential expression of HSP70 and HSP60 mRNA after temperature upshift. Infect Immun. 1998;66(7):3106-12. Epub 1998/06/25. doi: 10.1128/iai.66.7.3106-3112.1998. PubMed PMID: 9632573; PubMed Central PMCID: PMCPMC108320.

1330. Zhang YL, Ohashi N, Rikihisa Y. Cloning of the heat shock protein 70 (HSP70) gene of Ehrlichia sennetsu and differential expression of HSP70 and HSP60 mRNA after temperature upshift. Infection and Immunity. 1998;66(7):3106-12. doi: 10.1128/iai.66.7.3106-3112.1998. PubMed PMID: WOS:000074379900012.

1331. Zhi N, Ohashi N, Rikihisa Y, Horowitz HW, Wormser GP, Hechemy K. Cloning and expression of the 44-kilodalton major outer membrane protein gene of the human granulocytic ehrlichiosis agent and application of the recombinant protein to serodiagnosis. J Clin Microbiol. 1998;36(6):1666-73. Epub 1998/06/10. doi: 10.1128/jcm.36.6.1666-1673.1998. PubMed PMID: 9620397; PubMed Central PMCID: PMCPMC104897.

1332. New assay for ehrlichiosis. JAMA. 1999;282(20):1908. doi: 10.1001/jama.282.20.1908.

1333. Human granulocytic ehrlichiosis in western Europe. New England Journal of Medicine; 1999. p. 1214-6.

1334. Al-Khaldi SF, Evans JD, Martin SA. Complete nucleotide sequence of a cryptic plasmid from the ruminal bacterium Selenomonas ruminantium HD4 and identification of two predicted open reading frames. Plasmid. 1999;42(1):45-52. doi: 10.1006/plas.1999.1405.

1335. Alberdi MP, Paxton EA, Clark AM, Watson P, Sumption KJ, Walker AR, editors. Sequence analysis of groEL gene reveals further differences between granulocytic Ehrlichia isolates of Europe and the United States. 3rd International Conference on Ticks and Tick-Borne Pathogens (TTP 3); 1999 Aug 30-Sep 03; High Tatra Mt, Slovakia2000.

1336. Allsopp MT, Hattingh CM, Vogel SW, Allsopp BA. Evaluation of 16S, map1 and pCS20 probes for detection of Cowdria and Ehrlichia species. Epidemiology & Infection. 1999;122(2):323-8. doi: 10.1017/S0950268899002101. PubMed PMID: 104717010. Language: English. Entry Date: 20110610. Revision Date: 20190529. Publication Type: journal article.

1337. Aoki N. [Sennetsu rickettsiosis]. Ryoikibetsu Shokogun Shirizu. 1999;(24 Pt 2):286-7. Epub 1999/04/14. PubMed PMID: 10201200.

1338. Arguin PM, Singleton J, Rotz LD, Marston E, Treadwell TA, Slater K, et al. An investigation into the possibility of transmission of tick-borne pathogens via blood transfusion. Transfusion-Associated Tick-Borne Illness Task Force. Transfusion. 1999;39(8):828-33. Epub 1999/09/30. doi: 10.1046/j.1537-2995.1999.39080828.x. PubMed PMID: 10504117.

1339. Arguin PM, Singleton J, Rotz LD, Marston E, Treadwell TA, Slater K, et al. An investigation into the possibility of transmission of tick-borne pathogens via blood transfusion. Transfusion. 1999;39(8):828-33. doi: 10.1046/j.1537-2995.1999.39080828.x.

1340. Arguin PM, Singleton J, Rotz LD, Marston E, Treadwell TA, Slater K, et al. An investigation into the possibility of transmission of tick-borne pathogens via blood transfusion. Transfusion-Associated Tick-Borne Illness Task Force. Transfusion. 1999;39(8):828-33. PubMed PMID: 107222634. Corporate Author: Transfusion-Associated Tick-Borne Illness Task Force. Language: English. Entry Date: 19991101. Revision Date: 20190819. Publication Type: journal article.

1341. Arguin PM, Singleton J, Rotz LD, Marston E, Treadwell TA, Slater K, et al. An investigation into the possibility of transmission of tick-borne pathogens via blood transfusion. Transfusion. 1999;39(8):828-33. doi: 10.1046/j.1537-2995.1999.39080828.x. PubMed PMID: WOS:000082052700006.

1342. Arraga-Alvarado C, Palmar M, Parra O, Salas P. Fine structural characterisation of a Rickettsia-like organism in human platelets from patients with symptoms of ehrlichiosis. J Med Microbiol. 1999;48(11):991-7. Epub 1999/10/27. doi: 10.1099/00222615-48-11-991. PubMed PMID: 10535642.

1343. Artursson K, Gunnarsson A, Wikstrom UB, Engvall EO. A serological and clinical follow-up in horses with confirmed equine granulocytic ehrlichiosis. Equine Veterinary Journal. 1999;31(6):473-7. doi: 10.1111/j.2042-3306.1999.tb03853.x. PubMed PMID: WOS:000083614600006.

1344. Barnewall RE, Ohashi N, Rikihisa Y. Ehrlichia chaffeensis and E. sennetsu, but not the human granulocytic ehrlichiosis agent, colocalize with transferrin receptor and up-regulate transferrin receptor mRNA by activating iron-responsive protein 1. Infect Immun. 1999;67(5):2258-65. Epub 1999/05/04. doi: 10.1128/iai.67.5.2258-2265.1999. PubMed PMID: 10225882; PubMed Central PMCID: PMCPMC115965.

1345. Barnewall RE, Ohashi N, Rikihisa Y. Ehrlichia chaffeensis and E-sennetsu, but not the human granulocytic ehrlichiosis agent, colocalize with transferrin receptor and up-regulate transferrin receptor mRNA by activating iron-responsive protein 1. Infection and Immunity. 1999;67(5):2258-65. doi: 10.1128/iai.67.5.2258-2265.1999. PubMed PMID: WOS:000079909300029.

1346. Baumgarten BU, Röllinghoff M, Bogdan C. Prevalence of Borrelia burgdorferi and granulocytic and monocytic ehrlichiae in Ixodes ricinus ticks from southern Germany. J Clin Microbiol. 1999;37(11):3448-51. Epub 1999/10/19. doi: 10.1128/jcm.37.11.3448-3451.1999. PubMed PMID: 10523532; PubMed Central PMCID: PMCPMC85664.

1347. Belman AL. Tick-borne diseases. Semin Pediatr Neurol. 1999;6(4):249-66. Epub 2000/01/29. doi: 10.1016/s1071-9091(99)80024-2. PubMed PMID: 10649834.

1348. Belongia EA, Reed KD, Mitchell PD, Chyou PH, Mueller-Rizner N, Finkel MF, et al. Clinical and epidemiological features of early Lyme disease and human granulocytic ehrlichiosis in Wisconsin. Clin Infect Dis. 1999;29(6):1472-7. Epub 1999/12/10. doi: 10.1086/313532. PubMed PMID: 10585798.

1349. Betts CJ, Else KJ. Mast cells, eosinophils and antibody-mediated cellular cytotoxicity are not critical in resistance to Trichuris muris. Parasite Immunology. 1999;21(1):45-52. doi: 10.1046/j.1365-3024.1999.00200.x.

1350. Bjöersdorff A, Berglund J, Kristiansen BE, Söderström C, Eliasson I. [Varying clinical picture and course of human granulocytic ehrlichiosis. Twelve Scandinavian cases of the new tick-borne zoonosis are presented]. Lakartidningen. 1999;96(39):4200-4. Epub 1999/11/02. PubMed PMID: 10544585.

1351. Bjöersdorff A, Berglund J, Kristiansen BE, Söderström C, Eltasson I. Variable presentation and course in human granulocytic ehrlichiosis; 12 case reports of the new tick-borne zoonosis. Lakartidningen. 1999;96(39):4200-4.

1352. Bjöersdorff A, Brouqui P, Eliasson I, Massung RF, Wittesjö B, Berglund J. Serological evidence of Ehrlichia infection in Swedish Lyme borreliosis patients. Scand J Infect Dis. 1999;31(1):51-5. Epub 1999/06/25. doi: 10.1080/00365549950161880. PubMed PMID: 10381218.

1353. Bleck TP. Central nervous system involvement in rickettsial diseases. Neurologic Clinics. 1999;17(4):801-+. doi: 10.1016/s0733-8619(05)70167-5. PubMed PMID: WOS:000083789500008.

1354. Bornay-Llinares FJ, da Silva AJ, Moura INS, Myjak P, Pietkiewicz H, Kruminis-Lozowska W, et al. Identification of Cryptosporidium felis in a cow by morphologic and molecular methods. Applied and Environmental Microbiology. 1999;65(4):1455-8. PubMed PMID: WOS:000079530000014.

1355. Bornay-Llinares FJ, Da Silva AJ, Moura INS, Myjak W, Pietkiewicz H, Kruminis-ŁOzowska W, et al. Identification of Cryptosporidium felis in a cow by morphologic and molecular methods. Applied and Environmental Microbiology. 1999;65(4):1455-8. doi: 10.1128/aem.65.4.1455-1458.1999.

1356. Bowie MV, Reddy GR, Semu SM, Mahan SM, Barbet AF. Potential value of major antigenic protein 2 for serological diagnosis of heartwater and related Ehrlichial infections. Clinical and Diagnostic Laboratory Immunology. 1999;6(2):209-15. doi: 10.1128/cdli.6.2.209-215.1999.

1357. Braclik M, Kucharz EJ. [Human ehrlichiosis]. Pol Arch Med Wewn. 1999;101(3):241-3. Epub 2000/03/04. PubMed PMID: 10697402.

1358. Braclik M, Kucharz EJ. Human ehrlichioses. Polskie Archiwum Medycyny Wewnetrznej. 1999;101(3):241-3.

1359. Brandsma AR, Little SE, Lockhart JM, Davidson WR, Stallknecht DE, Dawson JE. Novel Ehrlichia organism (Rickettsiales : Ehrlichieae) in white-tailed deer associated with lone star tick (Acari : Ixodidae) parasitism. Journal of Medical Entomology. 1999;36(2):190-4. doi: 10.1093/jmedent/36.2.190. PubMed PMID: WOS:000078828300013.

1360. Brouqui P. Adaptation of the agent of the human monocyte ehrlichiosis (Ehrlichia chaffeensis) to HL60. Am J Trop Med Hyg. 1999;60(4):518-9. Epub 1999/05/29. doi: 10.4269/ajtmh.1999.60.518. PubMed PMID: 10348219.

1361. Brown TJ, Donaghy MJ, Keys EA, Ionas G, Learmonth JJ, McLenachan PA, et al. The viability of Giardia intestinalis and Giardia muris cysts in seawater. International Journal of Environmental Health Research. 1999;9(2):157-61. doi: 10.1080/09603129973290.

1362. Buller RS, Arens M, Hmiel SP, Paddock CD, Sumner JW, Rikhisa Y, et al. Ehrlichia ewingii, a newly recognized agent of human ehrlichiosis. N Engl J Med. 1999;341(3):148-55. Epub 1999/07/15. doi: 10.1056/nejm199907153410303. PubMed PMID: 10403852.

1363. Buller RS, Arens M, Hmiel SP, Paddock CD, Sumner JW, Rikihisa Y, et al. Ehrlichia ewingii, A newly recognized agent of human ehrlichiosis. New England Journal of Medicine. 1999;341(3):148-55. doi: 10.1056/nejm199907153410303. PubMed PMID: WOS:000081413800003.

1364. Bunnell JE, Magnarelli LA, Dumler JS. Infection of laboratory mice with the human granulocytic ehrlichiosis agent does not induce antibodies to diagnostically significant Borrelia burgdorferi antigens. J Clin Microbiol. 1999;37(6):2077-9. Epub 1999/05/15. doi: 10.1128/jcm.37.6.2077-2079.1999. PubMed PMID: 10325386; PubMed Central PMCID: PMCPMC85039.

1365. Bunnell JE, Trigiani ER, Srinivas SR, Dumler JS. Development and distribution of pathologic lesions are related to immune status and tissue deposition of human granulocytic ehrlichiosis agent-infected cells in a murine model system. J Infect Dis. 1999;180(2):546-50. Epub 1999/07/09. doi: 10.1086/314902. PubMed PMID: 10395880.

1366. Callaway TR, Adams KA, Russell JB. The ability of 'low G + C Gram-positive' ruminal bacteria to resist monensin and counteract potassium depletion. Current Microbiology. 1999;39(4):226-30. doi: 10.1007/s002849900449.

1367. Childs JE, Ellis BA, Nicholson WL, Kosoy M, Sumner JW. Shared vector-borne zoonoses of the Old World and New World: home grown or translocated? Schweiz Med Wochenschr. 1999;129(31-32):1099-105. Epub 1999/09/07. PubMed PMID: 10476548.

1368. Childs JE, Sumner JW, Nicholson WL, Massung RF, Standaert SM, Paddock CD. Outcome of diagnostic tests using samples from patients with culture-proven human monocytic ehrlichiosis: implications for surveillance. J Clin Microbiol. 1999;37(9):2997-3000. Epub 1999/08/17. doi: 10.1128/jcm.37.9.2997-3000.1999. PubMed PMID: 10449489; PubMed Central PMCID: PMCPMC85432.

1369. Christmann S. The order of the physical world - On the poetry and poetics of Charles H. Sisson. Aaa-Arbeiten Aus Anglistik Und Amerikanistik. 1999;24(2):229-45. PubMed PMID: WOS:000085327300005.

1370. Christova IS, Dumler JS. Human granulocytic ehrlichiosis in Bulgaria. Am J Trop Med Hyg. 1999;60(1):58-61. Epub 1999/02/13. doi: 10.4269/ajtmh.1999.60.58. PubMed PMID: 9988323.

1371. Comer JA, Nicholson WL, Olson JG, Childs JE. Serologic testing for human granulocytic ehrlichiosis at a national referral center. J Clin Microbiol. 1999;37(3):558-64. Epub 1999/02/13. doi: 10.1128/jcm.37.3.558-564.1999. PubMed PMID: 9986812; PubMed Central PMCID: PMCPMC84468.

1372. Comer JA, Nicholson WL, Sumner JW, Olson JG, Childs JE. Diagnosis of human ehrlichiosis by PCR assay of acute-phase serum. J Clin Microbiol. 1999;37(1):31-4. Epub 1998/12/17. doi: 10.1128/jcm.37.1.31-34.1999. PubMed PMID: 9854059; PubMed Central PMCID: PMCPMC84159.

1373. Davidson WR, Lockhart JM, Stallknecht DE, Howerth EW. Susceptibility of red and gray foxes to infection by Ehrlichia chaffeensis. Journal of Wildlife Diseases. 1999;35(4):696-702. doi: 10.7589/0090-3558-35.4.696.

1374. Deng MQ, Cliver DO. Improved immunofluorescence assay for detection of Giardia and Cryptosporidium from asymptomatic adult cervine animals. Parasitology Research. 1999;85(8-9):733-6. doi: 10.1007/s004360050623.

1375. Des Vignes F, Levin ML, Fish D. Comparative vector competence of Dermacentor variabilis and Ixodes scapularis (Acari: Ixodidae) for the agent of human granulocytic ehrlichiosis. J Med Entomol. 1999;36(2):182-5. Epub 1999/03/20. doi: 10.1093/jmedent/36.2.182. PubMed PMID: 10083755.

1376. Dubinina EV, Alekseev AN. [The biodiversity dynamics of the causative agents of diseases transmitted by ticks in the genus Ixodes: an analysis of multiyear data]. Med Parazitol (Mosk). 1999;(2):13-9. Epub 2000/03/07. PubMed PMID: 10703200.

1377. Dumler JS, Valsamakis A. Molecular diagnostics for existing and emerging infections - Complementary tools for a new era of clinical microbiology. American Journal of Clinical Pathology. 1999;112(1):S33-S9. PubMed PMID: WOS:000081173400004.

1378. Dumpis U, Crook D, Oksi J. Tick-borne encephalitis. Clinical Infectious Diseases. 1999;28(4):882-90. doi: 10.1086/515195. PubMed PMID: WOS:000079611600034.

1379. Eastlund T, Persing D, Mathiesen D, Kim D, Bieging J, McCann P, et al. Human granulocytic ehrlichiosis after red cell transfusion. Transfusion. 1999;39(10):117S-S. PubMed PMID: WOS:000083207500555.

1380. Ebisawa I. [Ehrlichiosis]. Ryoikibetsu Shokogun Shirizu. 1999;(23 Pt 1):104-6. Epub 1999/03/24. PubMed PMID: 10088349.

1381. Edlow JA. Lyme disease and related tick-borne illnesses. Annals of Emergency Medicine. 1999;33(6):680-93. doi: 10.1016/s0196-0644(99)80007-9. PubMed PMID: WOS:000080626200007.

1382. Elwood CM, Garden OA. Gastrointestinal immunity in health and disease. Veterinary Clinics of North America-Small Animal Practice. 1999;29(2):471-+. PubMed PMID: WOS:000079767400008.

1383. Estrada-Pena A, Jongejan F. Ticks feeding on humans: a review of records on human-biting Ixodoidea with special reference to pathogen transmission. Experimental and Applied Acarology. 1999;23(9):685-715. doi: 10.1023/a:1006241108739. PubMed PMID: WOS:000082469100001.

1384. Faul JL, Doyle RL, Kao PN, Ruoss SJ. Tick-borne pulmonary disease - Update on diagnosis and management. Chest. 1999;116(1):222-30. doi: 10.1378/chest.116.1.222. PubMed PMID: WOS:000081513200037.

1385. Fehrsen J, Du Plessis DH. Cross-reactive epitope mimics in a fragmented-genome phage display library derived from the rickettsia, Cowdria ruminantium. Immunotechnology. 1999;4(3-4):175-84. doi: 10.1016/S1380-2933(98)00018-9.

1386. Felz MW, Durden LA. Attachment sites of four tick species (Acari: Ixodidae) parasitizing humans in Georgia and South Carolina. J Med Entomol. 1999;36(3):361-4. Epub 1999/05/25. doi: 10.1093/jmedent/36.3.361. PubMed PMID: 10337108.

1387. Fingerle V. Epidemiological aspects of human granulocytic Ehrlichiosis in Southern Germany. Wiener Klinische Wochenschrift. 1999;111(22-23):1000-4.

1388. Fingerle V, Goodman JL, Johnson RC, Kurtti TJ, Munderloh UG, Wilske B. Epidemiological aspects of human granulocytic Ehrlichiosis in southern Germany. Wien Klin Wochenschr. 1999;111(22-23):1000-4. Epub 2000/02/10. PubMed PMID: 10666819.

1389. Fingerle V, Munderloh UG, Liegl G, Wilske B. Coexistence of ehrlichiae of the phagocytophila group with Borrelia burgdorferi in Ixodes ricinus from Southern Germany. Medical Microbiology and Immunology. 1999;188(3):145-9. doi: 10.1007/s004300050117. PubMed PMID: WOS:000085309400006.

1390. Foley JE, Crawford-Miksza L, Dumler JS, Glaser C, Chae JS, Yeh E, et al. Human granulocytic ehrlichiosis in Northern California: two case descriptions with genetic analysis of the Ehrlichiae. Clin Infect Dis. 1999;29(2):388-92. Epub 1999/09/07. doi: 10.1086/520220. PubMed PMID: 10476747.

1391. Foley JE, Foley P, Jecker M, Swift PK, Madigan JE. Granulocytic ehrlichiosis and tick infestation in mountain lions in California. Journal of Wildlife Diseases. 1999;35(4):703-9. doi: 10.7589/0090-3558-35.4.703.

1392. Foley JE, Lerche NW, Dumler JS, Madigan JE. A simian model of human granulocytic ehrlichiosis. Am J Trop Med Hyg. 1999;60(6):987-93. Epub 1999/07/14. doi: 10.4269/ajtmh.1999.60.987. PubMed PMID: 10403332.

1393. French DM, Brown WC, Palmer GH. Emergence of Anaplasma marginale antigenic variants during persistent rickettsemia. Infect Immun. 1999;67(11):5834-40. Epub 1999/10/26. doi: 10.1128/iai.67.11.5834-5840.1999. PubMed PMID: 10531237; PubMed Central PMCID: PMCPMC96963.

1394. Freyer B, Hansner T, Mehlhorn H, Rüger W. Characterization of a genomic region encoding the 32-kDa dense granule antigen of Sarcocystis muris (Apicomplexa). Parasitology Research. 1999;85(11):923-7. doi: 10.1007/s004360050659.

1395. George JC. About a first case of human granulocytic ehrlichiosis in France revealed by a summer flu-like syndrom. Revue du Praticien - Medecine Generale. 1999;13(475):1715-7.

1396. Golightly MG, Benach J. Tick-borne diseases. Reviews and Research in Medical Microbiology. 1999;10(1):1-10. doi: 10.1097/00013542-199901000-00001.

1397. Gongora Biachi RA, Velazquez JZ, Castro Sansores CJ, Martinez PG. First case of human ehrlichiosis in Mexico. Enfermedades Infecciosas y Microbiologia. 1999;19(3):139.

1398. Gongóra-Biachi RA, Zavala-Velázquez J, Castro-Sansores CJ, González-Martínez P. First case of human ehrlichiosis in Mexico. Emerg Infect Dis. 1999;5(3):481. Epub 1999/05/26. doi: 10.3201/eid0503.990327. PubMed PMID: 10341193; PubMed Central PMCID: PMCPMC2640767.

1399. Goodman JL. Ehrlichiosis--ticks, dogs, and doxycycline. N Engl J Med. 1999;341(3):195-7. Epub 1999/07/15. doi: 10.1056/nejm199907153410311. PubMed PMID: 10403860.

1400. Goodman JL. Ehrlichiosis - Ticks, dogs, and doxycycline. New England Journal of Medicine. 1999;341(3):195-7. doi: 10.1056/NEJM199907153410311.

1401. Goodman JL, Nelson CM, Klein MB, Hayes SF, Weston BW. Leukocyte infection by the granulocytic ehrlichiosis agent is linked to expression of a selectin ligand. J Clin Invest. 1999;103(3):407-12. Epub 1999/02/02. doi: 10.1172/jci4230. PubMed PMID: 9927502; PubMed Central PMCID: PMCPMC407896.

1402. Granström M, editor Ehrlichiosis and babesiosis, emerging human tick-borne diseases in Europe? Zentralblatt fur Bakteriologie; 1999.

1403. Granstrom M. New emerging tick-borne diseases. Bulletin De L Academie Nationale De Medecine. 1999;183(7):1391-8. PubMed PMID: WOS:000084662800012.

1404. Granstrom M. Ehrlichiosis and babesiosis, emerging human tick-borne diseases in Europe? Zentralblatt Fur Bakteriologie-International Journal of Medical Microbiology Virology Parasitology and Infectious Diseases. 1999;289(5-7):756-9. PubMed PMID: WOS:000085052700051.

1405. Gratz NG. Emerging and resurging vector-borne diseases. Annual Review of Entomology. 1999;44:51-75. doi: 10.1146/annurev.ento.44.1.51. PubMed PMID: WOS:000078435900004.

1406. Greenberg SB. Serious waterborne and wilderness infections. Critical Care Clinics. 1999;15(2):387-+. doi: 10.1016/s0749-0704(05)70060-9. PubMed PMID: WOS:000080605800010.

1407. Gubbels JM, De Vos AP, Van Der Weide M, Viseras J, Schouls LM, De Vries E, et al. Simultaneous detection of bovine Theileria and Babesia species by reverse line blot hybridization. Journal of Clinical Microbiology. 1999;37(6):1782-9. doi: 10.1128/jcm.37.6.1782-1789.1999.

1408. Gustafson R, Artursson K. [Ehrlichiosis is common among animals but can also occur in humans]. Lakartidningen. 1999;96(37):3884-7. Epub 1999/10/16. PubMed PMID: 10522093.

1409. Gustafson R, Artursson K. Ehrlichiosis, common among animals but also occurs in humans. Lakartidningen. 1999;96(37):3884-7.

1410. Haas CN, Aturaliye DN. Kinetics of electroporation-assisted chlorination of Giardia muris. Water Research. 1999;33(8):1761-6. doi: 10.1016/S0043-1354(98)00406-0.

1411. Hansner T, Freyer B, Mehlhorn H, Rüger W. Isolation and characterization of a cDNA clone encoding a thiol proteinase of Sarcocystis muris cyst mero' zoites (Apicomplexa). Parasitol Res. 1999;85(8-9):749-57. Epub 1999/08/04. doi: 10.1007/s004360050626. PubMed PMID: 10431744.

1412. Harrus S, Waner T, Bark H, Jongejan F, Cornelissen AWCA. Recent advances in determining the pathogenesis of canine monocytic ehrlichiosis. Journal of Clinical Microbiology. 1999;37(9):2745-9. doi: 10.1128/jcm.37.9.2745-2749.1999.

1413. Herron MJ, Nelson CM, Goodman JL. Binding of Human Granulocytic Ehrlichia to HL60 is a lectin-type interaction. Faseb Journal. 1999;13(5):A969-A. PubMed PMID: WOS:000082132901864.

1414. Hilton E, DeVoti J, Benach JL, Halluska ML, White DJ, Paxton H, et al. Seroprevalence and seroconversion for tick-borne diseases in a high-risk population in the northeast United States. Am J Med. 1999;106(4):404-9. Epub 1999/05/04. doi: 10.1016/s0002-9343(99)00046-7. PubMed PMID: 10225242.

1415. Hilton E, DeVoti J, Benach JL, Halluska ML, White DJ, Paxton H, et al. Seroprevalence and seroconversion for tickborne diseases in a high-risk population in the northeast United States. American Journal of Medicine. 1999;106(4):404-9. doi: 10.1016/s0002-9343(99)00046-7. PubMed PMID: WOS:000079840100005.

1416. Hong SJ, Woo HC, Lee SU, Huh S. Infection status of dragonflies with Plagiorchis muris metacercariae in Korea. The Korean journal of parasitology. 1999;37(2):65-70. doi: 10.3347/kjp.1999.37.2.65.

1417. Horowitz HW, Aguero-Rosenfeld ME, Wormser GP. Recurrent human granulocytic ehrlichiosis and Lyme disease - In response. Annals of Internal Medicine. 1999;130(12):1029-30. doi: 10.7326/0003-4819-130-12-199906150-00101. PubMed PMID: WOS:000080894700022.

1418. Hossain D, Aguero-Rosenfeld ME, Horowitz HW, Wu JM, Hsieh TC, Sachdeva N, et al. Clinical and laboratory evolution of a culture-confirmed case of human granulocytic ehrlichiosis. Conn Med. 1999;63(5):265-70. Epub 1999/06/11. PubMed PMID: 10363404.

1419. Hsieh T, DiPietrantonio AM, Horowitz HW, Dumler JS, Aguero-Rosenfeld ME, Wormser GP, et al. Changes in expression of the 44-kilodalton outer surface membrane antigen (p44 kD) for monitoring progression of infection and antimicrobial susceptibility of the human granulocytic ehrlichiosis (HGE) agent in HL-60 cells. Biochem Biophys Res Commun. 1999;257(2):351-5. Epub 1999/04/13. doi: 10.1006/bbrc.1999.0457. PubMed PMID: 10198216.

1420. Hsieh TC, Dipietrantonio AM, Horowitz HW, Dumler JS, Aguero-Rosenfeld ME, Wormser GP, et al. Changes in expression of the 44-kilodalton outer surface membrane antigen (p44 kD) for monitoring progression of infection and antimicrobial susceptibility of the human granulocytic ehrlichiosis (HGE) agent in HL-60 cells. Biochemical and Biophysical Research Communications. 1999;257(2):351-5. doi: 10.1006/bbrc.1999.0457.

1421. Hunfeld KP, Brade V. Prevalence of antibodies against the human granulocytic ehrlichiosis agent in Lyme borreliosis patients from Germany. Eur J Clin Microbiol Infect Dis. 1999;18(3):221-4. Epub 1999/06/05. doi: 10.1007/s100960050264. PubMed PMID: 10357060.

1422. Huygelen C. [Report of the first congress of the "European Society for Emerging Infections". (Budapest, 13-16 September 1998)]. Verh K Acad Geneeskd Belg. 1999;61(6):649-59. Epub 2000/02/03. PubMed PMID: 10655775.

1423. Ijdo JW, Wu C, Magnarelli LA, Fikrig E. Serodiagnosis of human granulocytic ehrlichiosis by a recombinant HGE- 44-based enzyme-linked immunosorbent assay. Journal of Clinical Microbiology. 1999;37(11):3540-4. doi: 10.1128/jcm.37.11.3540-3544.1999.

1424. Ijdo JW, Wu CY, Magnarelli LA, Fikrig E. Serodiagnosis of human granulocytic ehrlichiosis by a recombinant HGE-44-based enzyme-linked immunosorbent assay. Journal of Clinical Microbiology. 1999;37(11):3540-4. doi: 10.1128/jcm.37.11.3540-3544.1999. PubMed PMID: WOS:000083154300020.

1425. Johnson JR. Recurrent human granulocytic ehrlichiosis and Lyme disease. Ann Intern Med. 1999;130(12):1029-30. Epub 1999/06/26. doi: 10.7326/0003-4819-130-12-199906150-00014. PubMed PMID: 10383360.

1426. Johnson JR. Recurrent human granulocytic ehrlichiosis and Lyme disease. Philadelphia, Pennsylvania: American College of Physicians; 1999. p. 1029-30.

1427. Johnson JR, Horowitz HW, Aguero-Rosenfeld ME, Wormser GP. Recurrent human granulocytic ehrlichiosis and Lyme disease [4] (multiple letters). Annals of Internal Medicine. 1999;130(12):1029-30. doi: 10.7326/0003-4819-130-12-199906150-00014.

1428. JW IJ, Wu C, Magnarelli LA, Fikrig E. Serodiagnosis of human granulocytic ehrlichiosis by a recombinant HGE-44-based enzyme-linked immunosorbent assay. J Clin Microbiol. 1999;37(11):3540-4. Epub 1999/10/19. doi: 10.1128/jcm.37.11.3540-3544.1999. PubMed PMID: 10523549; PubMed Central PMCID: PMCPMC85687.

1429. Karp CL, Neva FA. Tropical infectious diseases in human immunodeficiency virus-infected patients. Clinical Infectious Diseases. 1999;28(5):947-63. doi: 10.1086/514745. PubMed PMID: WOS:000079955800001.

1430. Kawahara M, Ito T, Suto C, Shibata S, Rikihisa Y, Hata K, et al. Comparison of Ehrlichia muris strains isolated from wild mice and ticks and serologic survey of humans and animals with E. muris as antigen. J Clin Microbiol. 1999;37(4):1123-9. Epub 1999/03/13. doi: 10.1128/jcm.37.4.1123-1129.1999. PubMed PMID: 10074536; PubMed Central PMCID: PMCPMC88659.

1431. Kawahara M, Ito T, Suto C, Shibata S, Rikihisa Y, Hata K, et al. Comparison of Ehrlichia muris strains isolated from wild mice and ticks and serologic survey of humans and animals with E-muris as antigen. Journal of Clinical Microbiology. 1999;37(4):1123-9. doi: 10.1128/jcm.37.4.1123-1129.1999. PubMed PMID: WOS:000079105500041.

1432. Keysary A, Amram L, Keren G, Sthoeger Z, Potasman I, Jacob A, et al. Serologic evidence of human monocytic and granulocytic ehrlichiosis in Israel. Emerg Infect Dis. 1999;5(6):775-8. Epub 1999/12/22. doi: 10.3201/eid0506.990605. PubMed PMID: 10603210; PubMed Central PMCID: PMCPMC2640796.

1433. Kimbell LM, Miller DL, Chavez W, Altman N. Molecular analysis of the 18S rRNA gene of Cryptosporidium serpentis in a wild-caught corn snake (Elaphe guttata guttata) and a five-species restriction fragment length polymorphism-based assay that can additionally discern C-parvum from C-wrairi. Applied and Environmental Microbiology. 1999;65(12):5345-9. PubMed PMID: WOS:000084036800024.

1434. Kordick SK, Breitschwerdt EB, Hegarty BC, Southwick KL, Colitz CM, Hancock SI, et al. Coinfection with multiple tick-borne pathogens in a Walker Hound kennel in North Carolina. J Clin Microbiol. 1999;37(8):2631-8. Epub 1999/07/16. doi: 10.1128/jcm.37.8.2631-2638.1999. PubMed PMID: 10405413; PubMed Central PMCID: PMCPMC85300.

1435. Korenberg EI. [Ehrlichiosis--new infectious pathology in Russia]. Med Parazitol (Mosk). 1999;(4):10-6. Epub 2001/02/28. PubMed PMID: 11220996.

1436. Kowalska-Duplaga K, Strus M, Heczko P, Krobicka B, Kurowska-Baran D, Mrukowicz JZ. Lactobif, a marketed probiotic product containing Bifidobacterium ruminantium, was not effective in the treatment of acute rotavirus diarrhoea in infants. Gut. 1999;44:17‐25. PubMed PMID: CN-01648066.
[truncated: 1,674,082 more chars]
