# Supplementary material for: Human monocytotropic ehrlichiosis—A systematic review and analysis of the literature
Source: PLoS Negl Trop Dis. 2024 Aug 2;18(8):e0012377. doi: 10.1371/journal.pntd.0012377 (PMC11324158; doi:10.1371/journal.pntd.0012377)
Supplement: S7 Text — (DOCX) [file pntd.0012377.s007.docx]

**Analysis of human ehrlichiosis CRID with coinfection(s)**

***Signs and symptoms***

For all of the 26 human ehrlichiosis CRID with coinfection(s), data on signs and symptoms was available. Figure 12 shows the frequency of the most commonly reported signs and symptoms.

**Fig. 12 Frequency of signs and symptoms in human ehrlichiosis cases with coinfection (n=26 cases, 23 immunocompetent, 3 immunocompromized).**

**
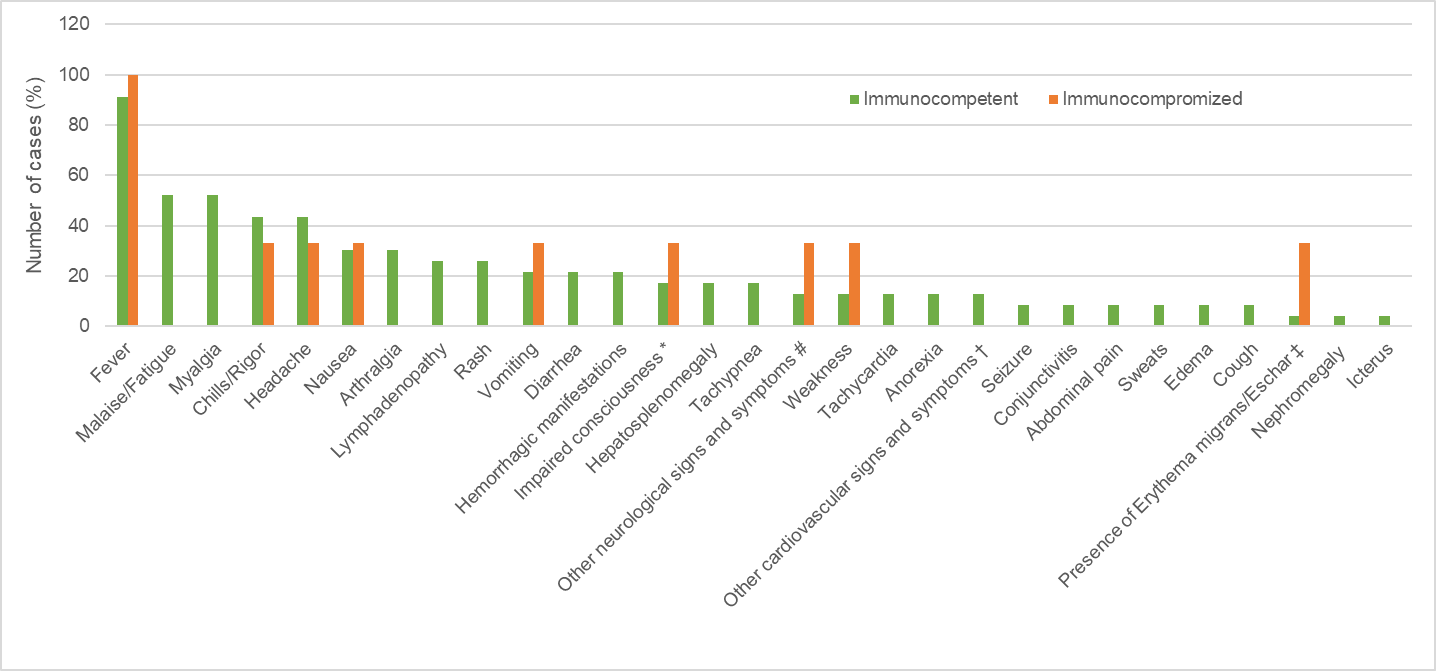
**

* definition: altered mental state, confusion, somnolence, delirium, coma.

^#^ hemorrhagic manifestations: petechiae (40%), epistaxis (20%), melaena (20%), petechiae + subconjunctival hemorrhage (20%).

^†^ other neurological signs and symptoms: ataxia (25%), hyperreflexia (25%), vision problems + ocular pain (25%), tremor + ataxia (25%).

^‡^ other cardiovascular signs and symptoms: palpitations (33%), arrhythmia (33%), atrial fibrillation (33%).

***Laboratory findings***

Of the 26 human ehrlichiosis CRID with coinfection(s), data on laboratory findings was available for 24 cases. Fig. 13 shows the frequency and Table 20 the median values and ranges of the most common abnormal findings in human ehrlichiosis CRID with coinfection(s).

**Fig. 13 Abnormal laboratory findings related to human ehrlichiosis with coinfection (n=24; 21 immunocompetent, 3 immunocompromized).**

**
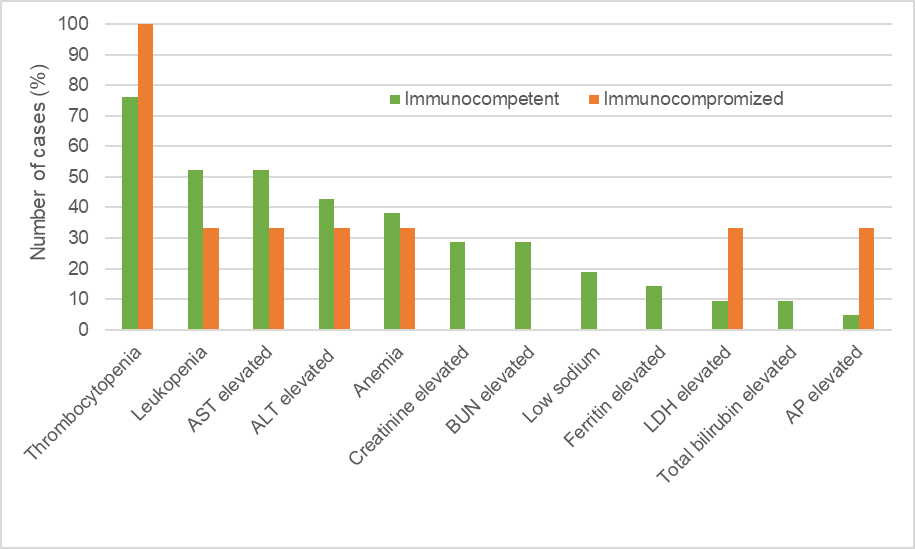
**

AST, aspartate aminotransferase; ALT, alanine aminotransferase; BUN, blood urea nitrogen; LDH, lactate dehydrogenase; AP, alkaline phosphatase.

**Table 18 Abnormal laboratory findings in human ehrlichiosis cases with coinfection (n=24)**

| Laboratory parameters | **Median** | **Range** | **Data available (n)** |
| --- | --- | --- | --- |
| Leukocytes (10^3^/µl) | 4.1 | 0.9-12.9 | 16 |
| Thrombocytes (10^3^/µl) | 68 | 7-181 | 18 |
| Hemoglobin (g/dl) | 11.6 | 2.6-14.6 | 10 |
| Hematocrit (%) | 25.7 | 6-36 | 3 |
| AST (U/l) | 465 | 24-2830 | 13 |
| ALT (U/l) | 179 | 26-725 | 11 |
| AP (U/l) | 138 | 77-295 | 5 |
| Total bilirubin (µmol/l) | 219 | 146-292 | 2 |
| LDH (U/l) | 1072 | 342-2307 | 3 |
| BUN (mg/dl) | 60 | 9-155 | 7 |
| Creatinine (µmol/l) | 2.5 | 0.2-6.6 | 7 |
| Ferritin (µg/l) | 21173 | 714-61437 | 3 |
| Sodium (mmol/l) | 131 | 126-139 | 5 |

AST, aspartate aminotransferase; ALT, alanine aminotransferase; AP, alkaline phosphatase; LDH, lactate dehydrogenase; BUN, blood urea nitrogen.

***Complications***

Complications were reported in 17 (71%) of 24 human ehrlichiosis CRID with coinfection(s), where information on complications were reported (Fig. 14).

**
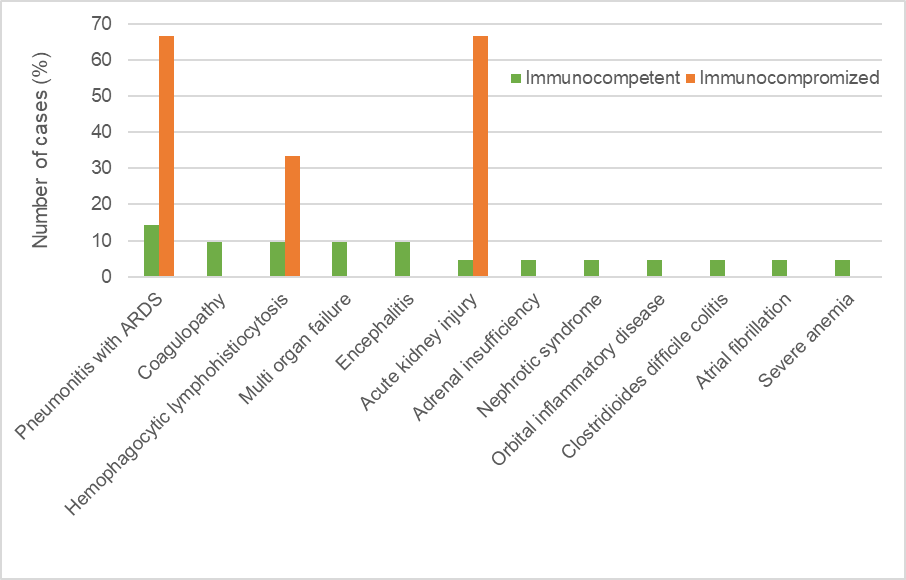
Fig. 14 Frequency of complications in human ehrlichiosis coinfection cases reported with individual data (n=24; 21 immunocompetent, 3 immunocompromized).**

ARDS, acute respiratory distress syndrome.

* as in many cases multiple complications were concomitantly present, the number of complications exceeds the number of cases.

***Outcome***

Data on the outcome was available for 24 of the HE CRID with coinfection(s): three (12.5%) died due to acute complications related to their infections. One of them was immunocompromized due to a splenectomy (Table 21), resulting in a case fatality rate of 9.5% in immunocompetent and 33.3% in immunocompromized patients.

Of the survivors with respectively available data, three, all immunocompetent patients (14.3%), were reported to suffer from sequelae (Table 22).

**Table 19 Reported fatal cases of HE CRID with coinfection(s) (n=3).**

| **No.** | **Year of publication** | **Age of patient(years)** | **Sex of patient** | **Country of infection** | **Pre-existing medical conditions** | **Immunosuppressive therapy /immunocom-promisation** | **Time between first symptoms and medical presentation (days)** | **Level of diagnostic certainty ^#^** | **Coinfection** | **Antimicrobial therapy** | **Time between presentation to hospital and specific therapy (days)** | **Complications/cause of death** | **Time from first symptoms to death** | **Ref.** |
| --- | --- | --- | --- | --- | --- | --- | --- | --- | --- | --- | --- | --- | --- | --- |
| 1 | 1990 | 67 | Male | USA | Diabetes, COPD | Splenectomy | N.r. | A | RMSF and Q fever | Doxycycline, Chloramphenicol | N.r. | AKI, respiratory failure | 25 | [1] |
| 2 | 2001 | 85 | Male | USA | Hypertension | N.r. | N.r. | B+ | *Babesia microti* | N.r. | N.r. | MOF, coagulopathy, respiratory failure | 7 | [2] |
| 3 | 2021 | 81 | Female | USA | Rheumatoid arthritis | N.r. | N.r. | N.r. | EBV | Doxycycline | N.r. | HLH, hemodynamic instability, shock | N.r. | [3] |

USA, United States of America; COPD, chronic obstructive lung disease; A, diagnosed by single IgG IFA serology; B+, diagnosed by microscopy; RMSF, rocky mountain spotted fever; EBV, Epstein-Barr virus; AKI, acute kidney injury; MOF, multi organ failure; HLH, hemophagocytic lymphohistiocytosis; Ref., reference; N.r. none/not reported.

**Table 20 Reported sequelae in HE CRID with coinfection(s) (n=3).**

| **No.** | **Year of publication** | **Age of patient (years)** | **Sex of patient** | **Country of infection** | **Preexisting medical conditions** | **Immunosuppressive treatment** | **Time between first symptoms and medical presentation (days)** | **Level of diagnostic certainty** | **Coinfection(s)** | **Antimicrobial treatment** | **Time between presentation to hospital and specific therapy (days)** | **Complications** | **Sequelae** | **Ref.** |
| --- | --- | --- | --- | --- | --- | --- | --- | --- | --- | --- | --- | --- | --- | --- |
| 1 | 1994 | 38 | Female | USA | N.r. | N.r. | 3 | B | *B.burgdorferi* | Doxycycline | 1 | N.r. | Recurrent symptoms for several months. | [4] |
| 2 | 2007 | 41 | Male | USA | N.r. | N.r. | 7 | A+ | *A.phagocytophilum* | Doxycycline | N.r. | Encephalopathy | Six months after infection he suffered from short-term memory impairment and rare seizures. | [5] |
| 3 | 2019 | 57 | Male | USA | Hepatitis C, alcohol abuse | N.r. | 6 | A+ | *E.coli* | Doxycycline | 2 | ARDS, AKI, DIC | Transferred to a long-term acute care facility because he could not be liberated from the ventilator and dialysis. | [6] |

HE, human ehrlichiosis; CRID, cases reported with individual data; USA, United States of America; A+, diagnosed by PCR; B, diagnosed by single IgG immunofluorescence assay serology; *B. burgdorferi; Borrelia burgdorferi; A. phagocytophilum, Anaplasma phagocytophilum; E. coli, Escherichia coli;* ARDS, acute respiratory distress syndrome; AKI, acute kidney injury; DIC, disseminated intravascular coagulation.

**References**

1. Eng TR, Harkess JR, Fishbein DB, Dawson JE, Greene CN, Redus MA, et al. EPIDEMIOLOGIC, CLINICAL, AND LABORATORY FINDINGS OF HUMAN EHRLICHIOSIS IN THE UNITED-STATES, 1988. Jama-Journal of the American Medical Association. 1990;264(17):2251-8. doi: 10.1001/jama.264.17.2251. PubMed PMID: WOS:A1990EF48800031.

2. Javed MZ, Srivastava M, Zhang S, Kandathil M. Concurrent babesiosis and ehrlichiosis in an elderly host. Mayo Clinic Proceedings. 2001;76(5):563-5. doi: 10.4065/76.5.563.

3. Zoglman J, Kapoor A, Naydenov S. A 13 syllable clinical conundrum. American Journal of Respiratory and Critical Care Medicine. 2021;203(9). doi: 10.1164/ajrccm-conference.2021.203.1_MeetingAbstracts.A2938.

4. Paparone PW, Glenn WB. Lyme disease with concurrent ehrlichiosis. Journal of the American Osteopathic Association. 1994;94(7):568+73+77. doi: 10.7556/jaoa.1994.94.7.568.

5. Young NP, Klein CJ. Encephalopathy with seizures having PCR-positive Anaplasma phagocytophilum and Ehrlichia chaffeensis [2]. European Journal of Neurology. 2007;14(2):e3-e4. doi: 10.1111/j.1468-1331.2006.01582.x.

6. Messana J, Davison D. An unexpected presentation of ticking off the inflammatory cascade. Critical Care Medicine. 2019;47(1).
