# Supplementary material for: Human monocytotropic ehrlichiosis—A systematic review and analysis of the literature
Source: PLoS Negl Trop Dis. 2024 Aug 2;18(8):e0012377. doi: 10.1371/journal.pntd.0012377 (PMC11324158; doi:10.1371/journal.pntd.0012377)
Supplement: S8 Text — (DOCX) [file pntd.0012377.s008.docx]

**Analysis of human ehrlichiosis infection cases reported with non-individual data (CRNID)**

Because of the often cumulative/pooled reported data of CRNID (i.e., case series, cohorts etc.), the analysis was more limited compared with CRID.

For 817 cases, the patient’s sex was reported. 264 (32%) were female and 553 (68%) male. Immunocompromisation was reported for 112 (13%) patients, all of them due to a solid organ transplantation. The suspected vector of disease in cases with respective data available was a tick bite in all cases, with 325 (39%) patients actually remembering a recent tick bite. In 68 cases, an outdoor activity as a risk factor for a tick bite was reported. In 36 (53%) cases the outdoor activity was occupational (military or agriculture) and in 32 (47%) cases recreational (golfing, unspecified activities near wooded area).

Of the 659 HME CRNID, data on whether they were symptomatic or not was available for 640 cases, showing 638 (99.7%) symptomatic and two (0.3%) asymptomatic cases. Hospital admission was reported for 471 (74%) of the symptomatic cases.

***Signs and symptoms***

For 462 of the 844 CRNID, data on signs and symptoms was available. Figure 15 shows the frequency of the most commonly reported symptoms.


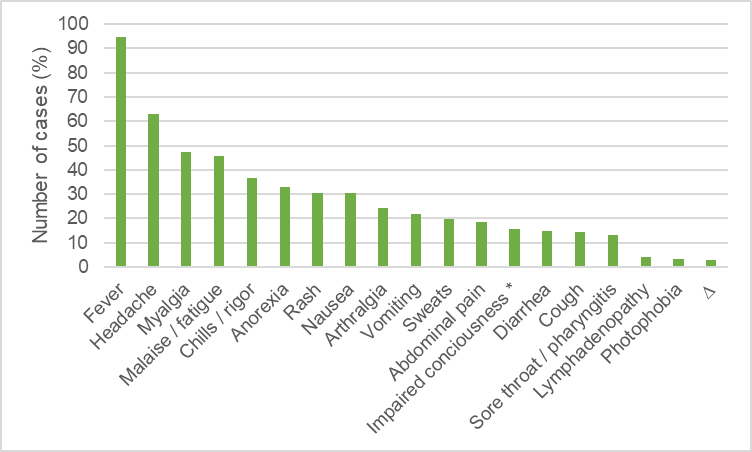
**Fig. 15 Signs and symptoms of cases of human monocytotropic ehrlichiosis reported with non-individual data (n=462).**

* = altered mental state, confusion, somnolence, delirium, coma

∆ more rare signs and symptoms not included in the figure: 2-3%: lymphoadenopathy, photophobia; 1-1.9%: neck stiffness, dyspnea; <1%: dysgeusia, hallucinations, stupor, eye pain, testicular pain, swollen joints, herpes labialis.

***Laboratory findings***

For 458 CRNID cases data on laboratory findings was available. Fig. 16 shows the frequency of the most common abnormal findings in CRNID.

**Fig. 16 Laboratory findings in human monocytotropic ehrlichiosis cases reported with non-individual data**
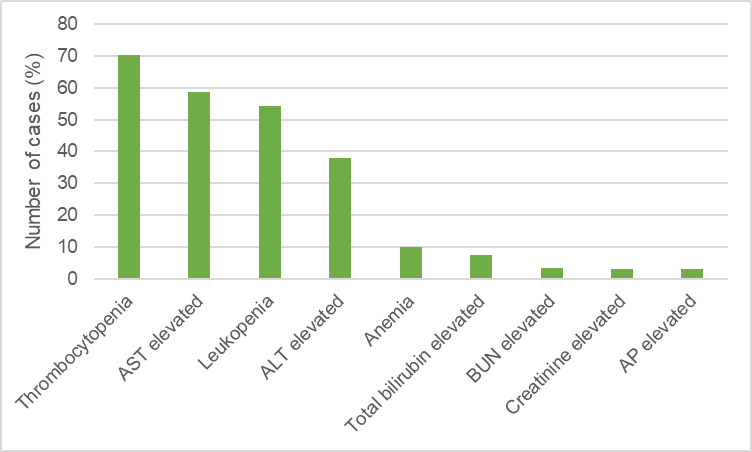
AST, aspartate aminotransferase; ALT, alanine aminotransferase; BUN, blood urea nitrogen; AP, alkaline phosphatase.

***Complications***

Data on complications was available for 328 CRNID. In 57 (17.4%) cases complications were reported. The most common reported complications included respiratory failure, renal failure, disseminated intravascular coagulation, encephalopathy, seizures and coma.

***Treatment***

For 622 of the 844 CRNID data on treatment was available. 564 (91%) cases received antimicrobial treatment, 25 (4%) cases received no antimicrobial treatment and for 33 (5%) it was unclear whether they did receive antimicrobial treatment or not. Of the 564 cases receiving antimicrobial treatment, 449 (80%) received appropriate antimicrobial therapy for human ehrlichiosis, 96 (17%) received no appropriate antimicrobial therapy for human ehrlichiosis and for 19 (3%) it was unclear whether they received appropriate antimicrobial therapy or not. Of the appropriately treated cases, 307 (68%) received doxycycline, 87 (19%) tetracycline, 10 (2%) chloramphenicol and for 45 (10%) the appropriate antimicrobial agent was not specified.

***Outcome***

Data on the outcome was available for 583 of the 844 CRNID. Four (0.7%) died due to HME and 579 (99.3%) survived. Of the survivors with respective data available, three (0.5%) suffered from sequelae.
