# Supplementary material for: Human monocytotropic ehrlichiosis—A systematic review and analysis of the literature
Source: PLoS Negl Trop Dis. 2024 Aug 2;18(8):e0012377. doi: 10.1371/journal.pntd.0012377 (PMC11324158; doi:10.1371/journal.pntd.0012377)
Supplement: Attachment — Submitted filename: Response to reviewers_24-6-24.docx [file pntd.0012377.s010.docx]

We cordially want to thank the two reviewers for taking their time to review our manuscript, their critical eyes, and their constructive remarks!

As mentioned in the manuscript, an in parallel conducted review entitled “Human granulocytotropic anaplasmosis – a systematic review and analysis of the literature” was conducted by our team and submitted to PLoS NTD and reviewed simultaneously. Unlike we suggested and hoped for in our submission letter the two manuscript were unfortunately handled by different editors and different reviewers. Therefore, next to the points raised by the two reviewers of this manuscript we also decided to in parallel implement some changes suggested by the reviewers of our review on anaplasmosis in this review on ehrlichiosis. Since both reviews follow the very same protocol, structure and all figures and tables match/mirror each other, we consider this the best approach in order to improve the manuscripts’ quality and the two works to depict true twin papers.

In the following, we will address the points listed by the two reviewers in chronological order and also address what we adapted in the manuscript following the comments of one of the reviewers (self-identified to be Prof. J. Stephen Dumler) who in parallel reviewed our anaplasmosis manuscript.

Reviewer #1:

1. The Article type should be Review, not Research Article
**RESPONSE:** We thought the same and submitted the manuscript as “review” but were corrected by the PLoS NTD editorial office, that “systematic reviews” fall under the category “research articles”.

2. Line 70, "Ehrlichioses and anaplasmoses" should be "Ehrlichiosis and anaplasmosis"?
**RESPONSE:** The plural is intentionally chosen as the different species do not necessarily cause the same disease pattern. Analogously, “Rickettsiosis is not a distinct single disease and the infections by the different species are referred to as “Rickettsioses”.

3. What does "The different Anaplasmataceae" mean? Do you mean different members of Anaplasmataceae?
**RESPONSE:** Yes, exactly.

4. The Introduction section lacks many references.
**RESPONSE:** We tried to reference the most important aspects, but not every single point. Since the introduction is not supposed to replace a text book and generally should be kept short we consider the 25 references in the introduction part to be more than adequate.

5. Lines 88-93, this sentence is hard to read. Please rephrase it.
**RESPONSE:** The sentence has been divided into to 2 sentences to enhance readability: “The first human infections caused by *Ehrlichia* were reported as early as 1954, when *Rickettsia sennetsu* (later *Ehrlichia sennetsu*, today *Neorickettsia sennetsu*) was identified as the causative agent of Sennetsu fever (a glandular fever-like disease) in Japan [2, 3]. Until then, bacteria of the family Anaplasmataceae (today including the genera *Ehrlichia*, *Anaplasma* and *Neorickettsia*) were primarily known as veterinary pathogens.”

6. Lines 99-102, this sentence is hard to read. Please rephrase it.
**RESPONSE:** The sentence has been divided into 2 sentences to enhance readability: “In 1999, *E. ewingii* (first described in 1971 [6]), a *Ehrlichia* strain closely related to *E. canis* and *E. chaffeensis* and, like *E. canis* a causative agent of canine ehrlichiosis, was found to also cause illness in humans. These *E. ewingii* infections presented with a clinical picture similar to that of HME caused by *E. chaffeensis* [7].”

7. Lines 102-103, "of both, E. chaffeensis and E. ewingii," should be "of both E. chaffeensis and E. ewingii".
**RESPONSE:** The semicolon has been removed as suggested.

8. Lines 108 and 111, "15'527" and "100'000" shoud be "15, 527" and "100, 000". Please revise it throughout the manuscript.
**RESPONSE:** The thousand-separator has been adapted as suggested.

9. Line 114, "which’s enzootic" seem should be "whose enzootic".
**RESPONSE:** The wording has been corrected as suggested.

10. Table 1, Table 6, and Lines 636-644. I would not recommend you cite and describe "Candidatus Ehrlichia erythraense". This is not a formally recognized Ehrlichia species.
**RESPONSE:** We listed this candidatus species for completeness, like we also listed candidatus species isolated from symptomatic patients in our in parallel conducted review on anaplasmosis. The fact that candidatus species are not yet acknowledged is self-explained in their naming, but for the reader we consider it relevant to communicate that emerging species will likely increase the spectrum of pathogens in the future.

11. Lines 131 and 133, "human granulocytotropic ehrlichiosis" and "human granulocytotropic anaplasmosis" should not be italicized.
**RESPONSE:** Corrected as suggested.

12. Line 135, "«ehrlichiosis»" . It is rare to use "«»".
**RESPONSE:** The "«»" has been removed.

13. Line 138, "[add reference later]"?? You forgot to add the reference?
**RESPONSE:** This part refers to our not yet published review on anaplasmosis. Until the proof reads we expect/hope to be able adding this cross-reference.

14. Line 172, "Besides microscopy" should be "Except for microscopy"?
**RESPONSE:** Corrected as suggested.

15. Table 4, Taiwan is a province of China. Please do not list it separately. Also, you can show it as Taiwan (China).
**RESPONSE:** The claim that Taiwan is a province of China is incorrect in our view: as long as (i) Taiwan has a president not identic to the president of China and (ii) citizens of Taiwan carry passports not identic to the passports carried by citizens of China the island does apparently not have the status of a Chinese province like e.g. Yunnan, Gansu, or Yunnan. In addition, the labeling as Chinese province would very likely be adequate in Chinese scientific journals, but considering that PLoS is an US American journal we consider the labeling according to the countries US American recognition to be adequate.

16. Table 14, The "Outcome", "cure" should be "cured"?
**RESPONSE:** Corrected as suggested.

17. Line 607, 615, and elsewhere in the manuscript. Is "HE" the same as "HME"?
**RESPONSE:** this point is addressed in line 298: “It should be noted that throughout the further manuscript we use the term HME when specifically referring to human infections with *E. canis* and *E. chaffeensis*, while we use the term HE when referring to human infections with *Ehrlichia*, regardless of species.”

Reviewer #2:

1. In abstract, the authors described, “Cases of HME are exclusively reported from North America.” (p2 L38) “Human monocytotropic ehrlichiosis (HME) is a bacterial disease caused by Ehrlichia chaffeensis which is transmitted by tick bites and exclusively reported from Northern America.” (p2 L52-54) In fact, cases of HME have been reported in various countries on different continents (p15-16 Table 4,5 Fig 5). The word “exculsively” was confusing.
**RESPONSE: The sentence has been changed to: “**Cases of HME are almost exclusively reported from North America. Human infections due to other (non-*chaffeensis*) *Ehrlichia* spp. are rare.”

2. Table 1 was not mentioned in the context.
**RESPONSE:** The following sentence has been added to the manuscript: “Table 1 shows the currently known human pathogenic *Ehrlichia* species.”

3. Captions for tables are placed above the table, and captions for figures are placed below the figure.
**RESPONSE:** the figures currently placed in the submitted raw-manuscript are only sreenshots of the original figures, submitted as separate files and only placed in the text to facilitate reviewing. The end-formating by the editorial office will resolve the addressed issue.

4. Although I appreciate the authors’ efforts, the Results are tediously long. Certain tables could be combined, e.g. Table 4 and 5; while some figures could be omitted or left in supporting files. In this way, interesting findings, such as rash which appeared much more often in children, and HLH which was associated with human ehrlichiosis at a surprisingly high rate, could be further addressed.
**RESPONSE:** as addressed, our two in parallel conducted reviews follow the same structure and table and figures mirror each other. Overall, it is a bit unfortunate, that not the same reviewers were assigned to the two manuscripts as wished by us. Only then, the full picture and the logic to compile and to dimension the two reviews becomes apparent. The fact that we have not addressed the differences in the incidence of rashes in children and adults, or in more detail ehrlichia associated HLH, is simply because there is currently no plausible models to explain these observations.

5. “…, the low rate of coinfection appears quite plausible when considering that most other tick-borne pathogens in HE endemic areas are transmitted by Ixodes spp. ticks and not by tick species transmitting HE.” Although human ehrlichiosis is transmitted primarily by the lone star tick (Amblyomma americanum) in north America, the blacklegged tick (Ixodes scapularis), which is known able to transmit Lyme disease and babesiosis, is also an important vector of human ehrlichiosis in the area. Moreover, Ehrlichia has been identified in Ixodes and other ticks, e.g. Ixodes ricinus, Ixodes pacificus, Dermacentor variabilis, and Ixodes persulcatus.
**RESPONSE:** We agree and therefore rephrased to: “However, reported data on the rate of co-infection in HE cases should generally be interpreted with caution (limited sample sizes, questionable representativeness, lack of systematic testing for coinfections). In addition, the report of concomitantly diagnosed coinfections should especially interpreted with caution if the causative pathogens are exclusively transmitted by different tick species.”

\# Prof. J Stephen Dumler (reviewer of the anaplasmosis review):

- Figure 1: We removed the anaplasmosis panel to only show ehrlichia morulae in this manuscript. In the anaplasmosis review, the same has been done, now only showing anaplasmosis morulae. We changed the caption of the figure to “Microscopical detection of *Ehrlichia chaffensis* *morulae*.” And the description to “Intracellular Ehrlichia chaffeensis morulae in: (A) light microscopy of the peripheral blood smear preparation (magnification x 1500); (B) leukocyte in the stroma of the ovary (magnification x 1200) [courtesy of Prof. Aileen M. Marty]” after corresponding with Prof. Marty.

- Figues 8,9,10: the three figures have been improved to now also show data according to the fractions of the cases’ diagnostic level of certainty: A+ and A vs. B+-D.

- Table 7: The footer/legend of table 7 has been extended for clarity by “* Note that the list of coinfections is based on the coinfections reported by the authors in the original publication, which, however, often cannot by checked for validity due to a lack of information on the corresponding diagnostics and therefore do not allow a conclusive assessment.”

- Table 15: The footer/legend of table 15 has been extended for clarity by “* tetracyclines (doxycycline, minocycline) and rifampicin were considered effective/appropriate; chloramphenicol was also considered effective/appropriate, although evidence is limited; beta-lactams, quinolons, macrolides, aminoglycosides, glycopeptides, nitroimidazoles, sulphonamides, and lincosamide were considered ineffective/inappropriate.
